# Highly-chemoselective step-down reduction of carboxylic acids to aromatic hydrocarbons *via* palladium catalysis†
†Electronic supplementary information (ESI) available: Experimental details and characterization data. See DOI: 10.1039/c9sc00892f


**DOI:** 10.1039/c9sc00892f

**Published:** 2019-04-29

**Authors:** Chengwei Liu, Zhi-Xin Qin, Chong-Lei Ji, Xin Hong, Michal Szostak

**Affiliations:** a College of Chemistry and Chemical Engineering and Key Laboratory of Auxiliary Chemistry and Technology for Chemical Industry , Ministry of Education , Shaanxi University of Science and Technology , Xi'an 710021 , China; b Department of Chemistry , Rutgers University , 73 Warren Street , Newark , NJ 07102 , USA . Email: michal.szostak@rutgers.edu; c Department of Chemistry , Zhejiang University , Hangzhou 310027 , China . Email: hxchem@zju.edu.cn

## Abstract

Aryl carboxylic acids are among the most abundant substrates in chemical synthesis and represent a perfect example of a traceless directing group that is central to many processes in the preparation of pharmaceuticals, natural products and polymers. Herein, we describe a highly selective method for the direct step-down reduction of carboxylic acids to arenes, proceeding *via* well-defined Pd(0)/(ii) catalytic cycle. The method shows a remarkably broad substrate scope, enabling to direct the classical acyl reduction towards selective decarbonylation by a redox-neutral mechanism. The utility of this reaction is highlighted in the direct defunctionalization of pharmaceuticals and natural products, and further emphasized in a range of traceless processes using removable carboxylic acids under mild, redox-neutral conditions orthogonal to protodecarboxylation. Extensive DFT computations were conducted to demonstrate preferred selectivity for the reversible oxidative addition and indicated that a versatile hydrogen atom transfer (HAT) pathway is operable.

## Abstract 2

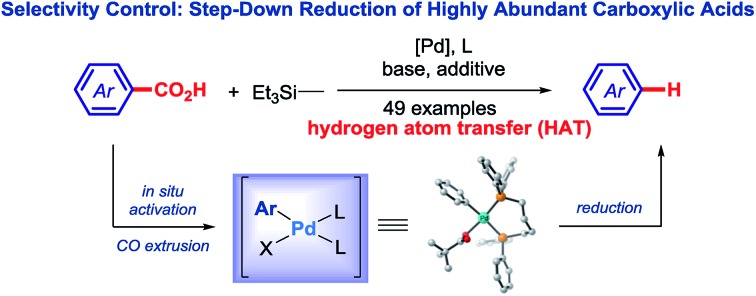
Herein, we describe a highly selective method for the direct decarbonylative step-down reduction of carboxylic acids to arenes, proceeding *via* well defined Pd(0)/(II) catalytic cycle.

## Introduction

**Fig. 1:**
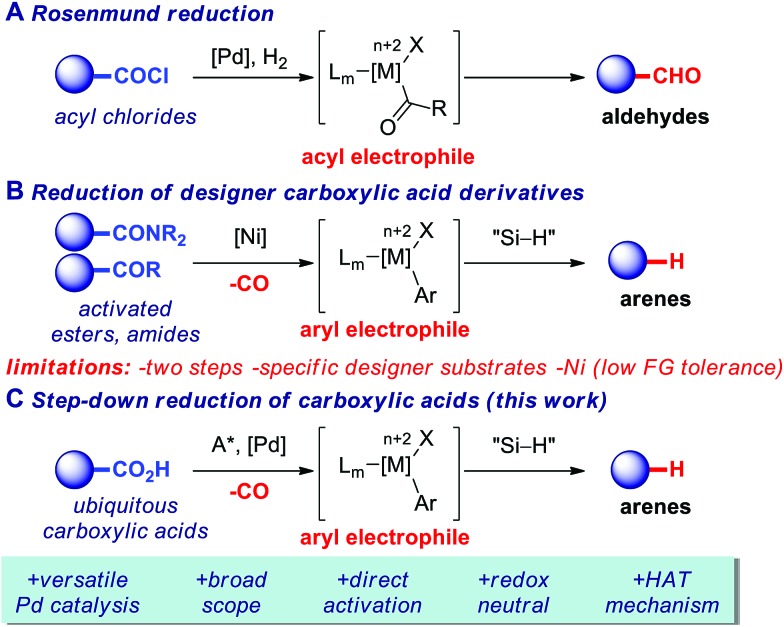
Transition-metal-catalyzed reduction of carboxylic acids.

The reduction of carboxylic acid derivatives represents one of the most fundamental transformations in synthetic chemistry and catalysis.^1^ Reduction reactions of carboxylic acids and derivatives are traditionally performed using stoichiometric metal hydrides.^2^ However, these reagents suffer from major scope limitations and are inherently less safe than milder silane-based hydrides due to their pyrophoric nature.^3^ The reduction of carboxylic acid chlorides to aldehydes (acyl pathway) has been achieved by Rosenmund using Pd-catalysis^4^*via* an oxidative insertion/transmetallation/reductive elimination mechanism,^5^ thus establishing the classic cross-coupling tactics for the synthesis of aldehydes from carboxylic acids (Fig. 1A). More recently, a Ni-catalyzed step-down reduction of N-chelating amides directly to hydrocarbons has been achieved by Maiti and co-workers,^6^ while the Rueping group developed a selective methodology for the Ni-catalyzed step-down reduction of phenolic esters and *N*-acyl-glutarimides (Fig. 1B),^7^ developed earlier by our group.^8^

Herein, we report the first highly selective method for the direct step-down reduction of ubiquitous carboxylic acids to arenes (decarbonylative pathway), proceeding *via* well-defined Pd(0)/(ii) cycle (Fig. 1C). The method supersedes the two-step methods using less general substrates and shows much broader reaction scope owing to the versatility of Pd-catalysis.^5^

The use of preformed carboxylic acid derivatives has been of choice to effect the direct reduction to hydrocarbons (Fig. 1B).^6^,^7^ Recognizing that these specifically-designed and less general N- and O-derivatives (pyrazoles, glutarimides, phenolic esters) are prepared from carboxylic acids in a separate step, we recently questioned whether a more straightforward approach engaging directly simple aromatic carboxylic acids could be realized in this important reaction class using versatile Pd-catalysis (Fig. 1C).

Specifically, we proposed that the direct reduction of carboxylic acids^9^,^10^ to hydrocarbons *via* a heretofore unknown redox-neutral, decarbonylative^11^–^15^ Pd(0)-catalyzed pathway could enable to use ubiquitous carboxylic acids as traceless activating groups, offering high level of predictability5a,b and functional group tolerance5e–g under redox-neutral conditions orthogonal to protodecarboxylation.^16^ Furthermore, the method would allow us for a more convenient approach to hydrocarbons that the reduction of designer N- and O-carboxylic acid derivatives, which are further limited by substrate scope.^6^,^7^ Finally, since aromatic carboxylic acids are commercially synthesized from the corresponding and cheaper toluenes, the method would establish Pd-promoted access to benzenes from feedstock toluenes, thus enabling to valorize crude oil as a mild alternative to toluene hydrodealkylation.^17^

## Results and discussion

We initiated our studies by probing the direct reduction of electronically- and sterically-unbiased 4-phenyl benzoic acid as a model substrate. After extensive optimization (see ESI†), we found that the reduction of 4-Ph-benzoic acid in the presence of Pd(OAc)_2_ (1 mol%), dppb (2 mol%, dppb = 1,4-bis(diphenylphosphino)butane), piv_2_O (1.5 equiv., piv = 2,2-dimethylpropanoyl), Et_3_N (1.5 equiv.) and Et_3_SiH (1.5 equiv.) as a hydride source afforded the desired reduction product in 97% yield on gram scale, attesting to the scalability of the method. Under the optimized conditions reduction to the aldehyde or aldehyde derived products was not observed, consistent with high facility of catalytic system to trigger the reduction (*cf.* acyl pathway) under redox-neutral conditions.

**Fig. 2:**
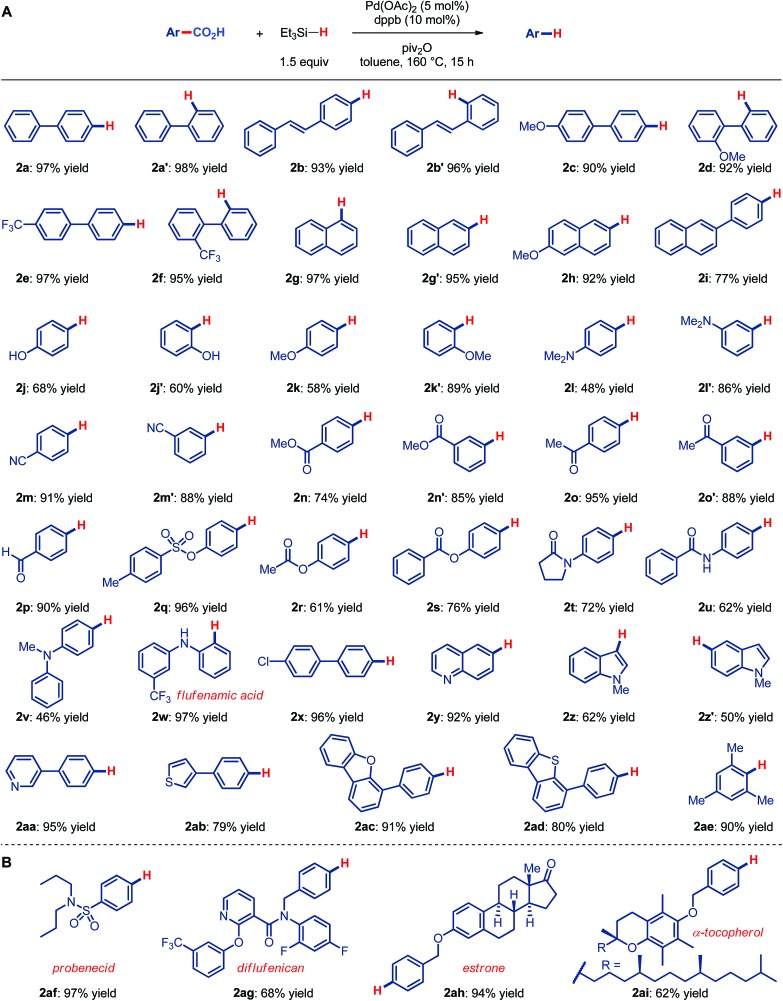
Scope of decarbonylative reduction of carboxylic acids. Conditions: carboxylic acid (1.0 equiv.), Pd(OAc)_2_ (5 mol%), dppb (10 mol%), Et_3_SiH (1.5 equiv.), piv_2_O (1.5 equiv.), toluene, 160 °C, 15 h. Dppb = 1,4-bis(diphenylphosphino)butane; piv = pivaloyl. See ESI for details.†

With optimal conditions in hand, we next focused on examining the scope of the decarbonylative reduction of carboxylic acids (Fig. 2A). We were delighted to find that the scope of the reaction is very broad and compatible with a variety of functional groups. As shown, unbiased as well as sterically-hindered aryl (**2a–a′**, **2c–f**) and alkenyl benzoic (**2b–b′**) acid underwent smooth reduction. Substitution with electron-donating (**2c–d**) or electron-withdrawing (**2e–f**) groups was readily tolerated. Simple (**2g–g′**) and substituted naphthalenes (**2h–i**) were found to be competent substrates. Notably the reduction is not limited to conjugated arenes^6^ and can be applied to a broad array of simple benzoic acids bearing a plethora of functional groups poised for further manipulation, including unprotected hydroxy (**2j–j′**), ethers (**2k–k′**), amines (**2l–l′**), nitriles (**2m–m′**), esters (**2n–n′**), ketones (**2o–o′**), aldehydes (**2p**), sulfonyl (**2q**), acyl groups (**2r–s**), amides (**2t–u**), amines (**2v–w**), and halides (**2x**). It is noteworthy that a range of heterocycles, including quinolines (**2y**), indoles (**2z–z′**), pyridines (**2aa**), thiophenes (**2ab**), benzofurans (**2ac**) and benzothiophenes (**2ad**), as well as extremely sterically-hindered carboxylic acids (**2af**) proceeded in high yields and with exquisite selectivity for decarbonylation. Overall, the scope of the reaction shows a number of clear advantages over other methods.^6^,^7^,^9^–^11^,^16^

To demonstrate the generality and potential impact of this new reduction method, we applied this protocol to late-stage derivatization of bioactive natural products and pharmaceuticals (Fig. 2B). We were delighted to find that decarboxylation of probenecid (**2af**) as well as of carboxylic acids derived from a fluorine-containing^18^ diflufenican (**2ag**), estrone (**2ah**) and tocopherol (**2ai**) afforded the decarbonylation products in high yields, underscoring the mild conditions and high potential impact of the present protocol.

**Fig. 3:**
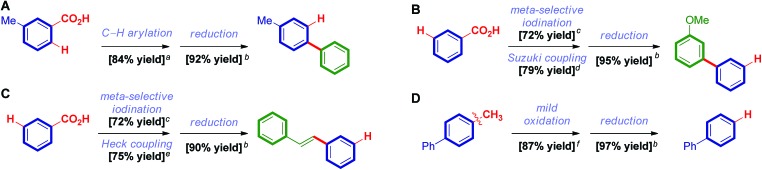
Synthetic applications. Conditions: ^a^PhI, [Ru(*p*-cym)Cl_2_]_2_, PCy_3_HBF_4_, K_2_CO_3_, NMP, 100 °C. ^b^Standard conditions. ^c^I_2_, NaIO_4_, H_2_SO_4_, 23 °C. ^d^3-MeO-C_6_H_4_-B(OH)_2_, Pd_2_(dba)_3_, SPhos, K_3_PO_4_, PhMe, 100 °C. ^e^PhCH=CH_2_, Pd_2_(dba)_3_, P*t*-Bu_3_HBF_4_, Et_3_N, dioxane, 23 °C. ^f^KMnO_4_, Na_2_CO_3_, H_2_O, 120 °C.

As a further illustration of the synthetic utility we conducted a series of metal-catalyzed and metal-free reactions using carboxylic acid as a traceless directing group (Fig. 3A–D). We were pleased to find that Ru-catalyzed *ortho*-arylation directed by a carboxylic acid,^19^ electrophilic meta-iodination/Suzuki cross-coupling^20^ and electrophilic meta-iodination/Heck cross-coupling^20^ provide rapid access to a range of valuable products in high yields, thus signifying a clear appeal of this novel method to organic synthesis. Furthermore, the decarbonylative reduction of carboxylic acids establishes valuable access to benzenes from feedstock toluenes (Fig. 3D). New valorization methods of oil processing products are of high interest from the industrial and sustainability standpoints.17a,b

Altogether, the broad scope of reactivity, tolerance to various sensitive functional groups and the potential to predictably use in functionalization of complex acids provide distinct advantages from other processes for removing carboxylic acid group^6^,^7^,^9^–^11^,^16^ and Bode well for future applications.

Although the scope of the reaction is very broad, several points should be noted: (1) typically, the yield can be improved by using small excess of Et_3_SiH (3.0 equiv.) and/or triethylamine as a base. For example, the yield of **2l** can be improved to 67% under these conditions. (2) In general, the reduction of electron-rich aromatics is less efficient than that of electron-deficient counterparts (*vide infra*). (3) Direct reduction of electro-rich five-membered heterocycles is feasible; for example, the reduction of 5-chlorothiophene-2-carboxylic acid proceeds in 91% yield. (4) Multiple reductions are feasible; for example, reduction of [1,1′-biphenyl]-3,3′,4,4′-tetracarboxylic acid (bis-phthalic acid) proceeds in 81% yield. (5) Halides on the carboxylic acid containing ring are possible. (6) We typically did not observe side reactions (*e.g.* aldehyde formation, reduction to the alcohol, deoxygenation). Studies on further expansion of the substrate scope are underway.

**Fig. 4:**
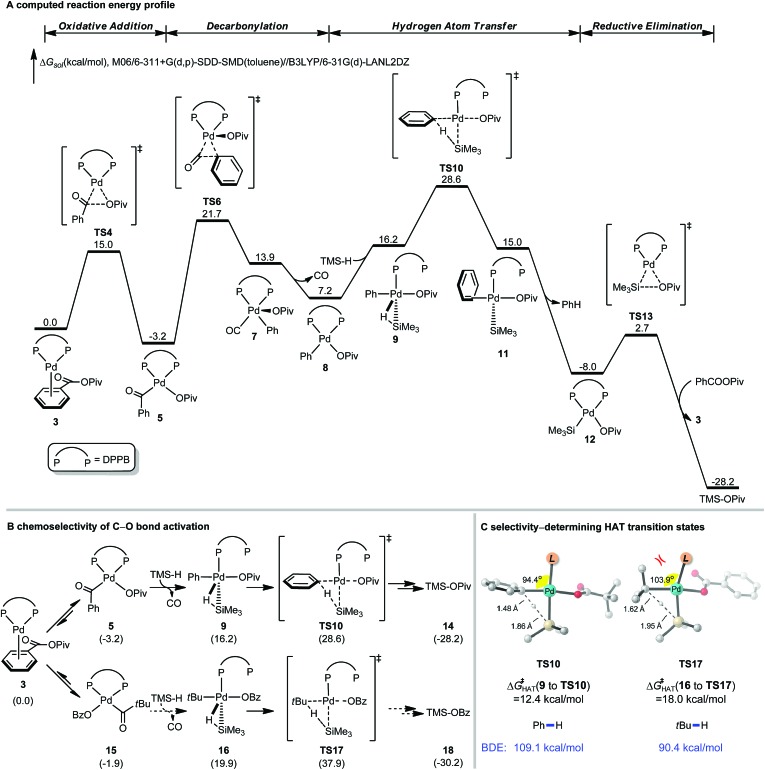
DFT-calculated reaction energy profile and chemoselectivity of C–O bond activation of [Pd(dppb)]-catalyzed decarbonylative reduction of benzoic pivalic anhydride. See ESI for computational details.†

Extensive computational studies were conducted to gain insight into the reaction mechanism and elucidate the controlling factors of selectivity. Recent advances in computational organometallic catalysis make this approach appealing to design more efficient catalytic systems and predict selectivity of bond activation events.^21^–^24^ The computed free energy profile of the catalytic cycle of decarbonylative reduction is shown in Fig. 4A. The C–O bond activation of benzoic pivalic anhydride proceeds *via***TS4**, generating the acylpalladium intermediate **5**. Subsequent decarbonylation through **TS6** leads to the penta-coordinated arylpalladium intermediate **7**, and CO then dissociates to produce the LPd(aryl)(OPiv) intermediate **8**. From **8**, the model silane (TMSH) coordinates, and a subsequent hydrogen atom transfer (HAT) occurs *via***TS10** to produce the reduced arene and the LPd(silyl)(OPiv) intermediate **12**. **12** undergoes a Si–O reductive elimination to regenerate the active Pd(0) catalyst for the next catalytic cycle. We were not able to locate the transition states for palladium-hydride formation despite extensive efforts. Based on the free energy changes of the overall catalytic cycle, the acylpalladium intermediate **5** is the on-cycle resting state, and the HAT step *via***TS10** is the rate-limiting step with an overall barrier of 31.8 kcal mol^–1^.

The chemoselectivity of C–O bond activation is determined by the substituent of anhydride. Fig. 4B shows the free energies of the key intermediates and HAT transition states of the competing C–O bond activation pathways. The HAT step determines the overall catalytic efficiency and differentiates the competing pathways by 9.3 kcal mol^–1^ (**TS10***vs.***TS17**). This computed selectivity is consistent with the experimental observations that the C–O bond activation only occurs on the benzoic acid. Detailed free energy changes of the reduction pathway involving C–O bond activation of pivalic acid are included in the ESI (Fig. S1†). Two factors contribute to this chemoselectivity. First, the steric repulsions between the bulky *t*Bu group and dppb ligand disfavor the pivalic acid C–O bond activation pathway. The highlighted angles in the HAT transition states reflect these steric effects; the phosphine ligand in **TS17** is significantly bent away from *t*Bu group (Fig. 4C). In addition, the phenyl group is intrinsically a better hydrogen atom acceptor comparing with *t*Bu group based on the bond dissociation energies (Ph–H: 109.1 kcal mol^–1^, *t*Bu–H: 90.4 kcal mol^–1^, Fig. 4C). This leads to the differences of intrinsic HAT barriers (12.4 kcal mol^–1^*via***TS10**, 18.0 kcal mol^–1^*via***TS17**, Fig. 4C), which further increases the chemoselectivity.

Additional studies were conducted to gain insight into the reaction mechanism (see ESI, Fig. S2–S7†). (1) To investigate whether benzoic pivalic anhydride was a possible reaction intermediate, 4-Ph-benzoic pivalic anhydride was prepared and subjected to the reaction conditions. Formation of product **2a** was observed (87% yield). Moreover, 4-Ph-benzoic acetic anhydride served as a competent intermediate (83% yield). (2) To investigate electronic effect on the decarbonylative step-down reduction, a Hammett correlation study employing differently substituted 4-Ar-benzoic acids was conducted. The study showed a large positive *ρ*-value of 1.57 (*R*^2^ = 0.99), which can be compared with the *ρ*^+^-value of 0.94 (*R*^2^ = 0.92) using Hammett–Brown σ^+^-constants, suggesting that electron-deficient arenes are inherently more reactive substrates, consistent with facility of metal insertion and decarbonylation. (3) To investigate steric effect on the decarbonylative step-down reduction, intermolecular competition experiments between differently substituted carboxylic acids were conducted, revealing that sterically-hindered carboxylic acids react preferentially, consistent with decarbonylation favored by steric demand of acylpalladium complexes. (4) To gain additional insight, relative reactivity studies regarding the use of biaryls, conjugated arenes and conjugated vinyl-arenes were conducted. The experiments revealed the following order of reactivity: biaryl = Np > vinyl-Ar. (5) To investigate the reduction selectivity, experiments at lower temperatures were conducted. The formation of aldehyde reduction products was not detected. (6) To investigate the effect of low catalytic loading, the reduction was conducted at 0.10 mol% of Pd(OAc)_2_. Formation of product **2a** from **1a** was observed in high yield (82% yield), consistent with the high efficiency of the reduction. Note that the reduction at low loading is also possible using Ac_2_O (51% yield), albeit with a decreased reaction efficiency. The beneficial effect of piv_2_O *vs.* Ac_2_O is consistent with the role of steric repulsion of the *t*-Bu group shutting down the alternative C–O cleavage pathway.12*h* Studies are currently in progress to investigate the mechanistic details of the decarbonylative cross-coupling manifold of carboxylic acids.

Finally, additional points regarding the impact and utility are in order. The facile reduction of carboxylic acids *via* a redox-neutral pathway should be benchmarked against the known methods for the reduction of carboxylic acid derivatives using Ni^6^,^7^ and the known methods *via* protodecarboxylation mechanism.^16^ (1) The benefits of the direct use of carboxylic acids *cf.* designer analogues are clear. (2) Furthermore, the broad scope of the reaction and the orthogonal mechanism for removing the carboxylic acid group expand the utility of carboxylic acids as directing groups in organic synthesis. (3) Perhaps most importantly, the transformation encompasses a general manifold for decarbonylative redox-neutral cross-coupling of ubiquitous carboxylic acids *via* a unified mechanism that provides a range of new compelling methods for manipulation of this privileged functional group.^25^

## Conclusions

In conclusion, we have reported the first method for a direct reduction of carboxylic acids to arenes *via* well-defined redox-neutral decarbonylative Pd(0)/(ii) catalytic cycle. The reaction provides a number of practical advantages for the construction of arenes over recently established two-step methods using designer derivatives of ubiquitous carboxylic acids. The reaction conditions are mild and tolerate a remarkably broad range of functional groups. The practical value of this transformation is evident from the potential to manipulate densely-functionalized substrates, including bioactive natural products and pharmaceuticals as well as in traceless reaction sequences. Detailed DFT study of the reaction mechanism has provided insight into the selectivity of bond activation events and elucidated steric requirements for a direct activation of carboxylic acids *via* a decarbonylative pathway. Studies towards expanding the scope of decarbonylative transformations of carboxylic acids and related substrates are underway and will be reported in due course.

## Conflicts of interest

There are no conflicts to declare.

## Supplementary Material

Supplementary information
