# Supplementary material for: Highly-chemoselective step-down reduction of carboxylic acids to aromatic hydrocarbons via palladium catalysis
Source: Chem Sci. 2019 Apr 29;10(22):5736–42. doi: 10.1039/c9sc00892f (PMC6568276; doi:10.1039/c9sc00892f)
Supplement: Supplementary file 1 [file SC-010-C9SC00892F-s001.pdf]

Electronic Supplementary Material (ESI) for Chemical Science.
This journal is © The Royal Society of Chemistry 2019

Supporting Information for

# Highly-Chemoselective Step-Down Reduction of Carboxylic Acids to Aromatic Hydrocarbons via Palladium Catalysis

Chengwei Liu,[1] Zhi-Xin Qin,[2] Chong-Lei Ji,[2] Xin Hong,[2,*] and Michal Szostak[1,*]

[1]Department of Chemistry, Rutgers University, 73 Warren Street, Newark, New Jersey 07102, United States

[2]Department of Chemistry, Zhejiang University, Hangzhou 310027, China

*Correspondence: hxchem@zju.edu.cn; michal.szostak@rutgers.edu

**Table of Contents** S1

Computational Details S2

Figure S1. Computational Results of the [Pd(dppb)]-Catalyzed Decarbonylative Reduction of Benzoic Pivalic Anhydride S3

Table S1. Energies in Figure 4 and Figure S1 S4

Additional Mechanistic Studies Referred to from the Main Manuscript S5

General Information S9

Experimental Procedures S11

Characterization Data of Reduction Products S12

References S39

Cartesian Coordinates of the Structures S42

$^{1}$H and $^{13}$C NMR Spectra S109

**Computational Details**

All density functional theory (DFT) calculations were performed with Gaussian 09 package.[1] All geometry optimizations were carried out at the B3LYP level of theory[2] using the LANL2DZ basis set[3] for Pd and the 6-31G(d) basis set for the other atoms. The vibrational frequencies were computed at the same level of theory to evaluate its zero-point vibrational energy (ZPVE) and thermal corrections at 298 K, and to check whether each optimized structure is an energy minimum or a transition state. The single point energies were calculated with the M06 functional[4] and a mixed basis set of SDD[5] for Pd and 6-311+G(d,p) for other atoms. Solvation energy corrections were calculated using the SMD model[6] with toluene as the solvent. All the calculated structures were visualized using CYLview.[7]

For the thermal corrections are based on the ideal gas model, this approach ignores the solvent suppression on the rotational and translational freedoms of solutes, resulting in overestimation of entropy contributions to the reaction free energies in solution.[8] To correct the entropy change in solution, An empirical approach proposed by Martin and co-workers was used,[9] because there is currently no widely accepted quantum mechanics-based approach to correct entropy in solution. According to their approach, a correction of 4.3 kcal/mol applies to per component change for a reaction at 298.15 K and 1 atm (i.e., a reaction from m- to n-components has an additional correction of (n−m) * 4.3 kcal/mol).This approach has been validated through a number of computational and experimental studies.[10] In order to adjust the Gibbs free energies from 1 atm to 1 mol/L, a correction of $RT\ln(c_s/c_g)$ (about 1.9 kcal/mol) is added to energies of all species except the carbon monoxide (CO),$c_s$ is the standard molar con-centration in solution (1 mol/L), $c_g$ is the standard molar con-centration in gas phase (0.0446 mol/L), and $R$ is the gas constant.

**Computational Results of the [Pd(dppb)]-Catalyzed Decarbonylative Reduction of Benzoic Pivalic Anhydride.**

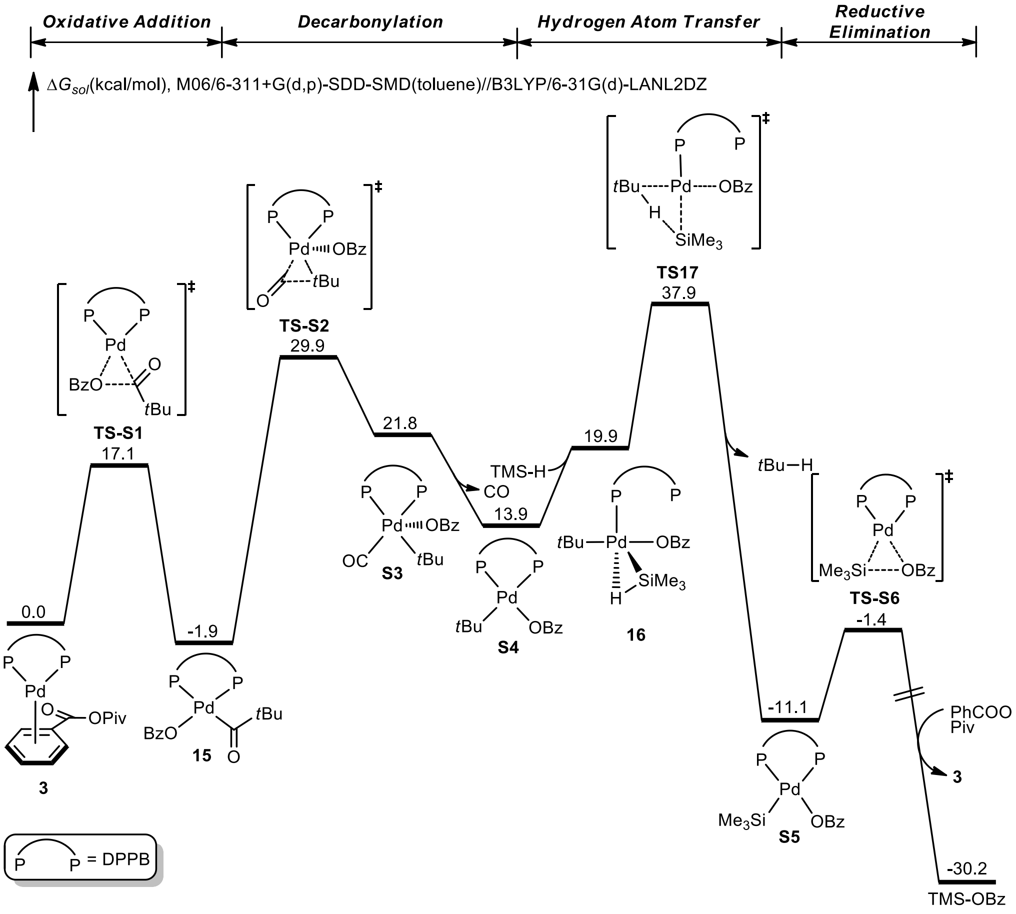


**Figure S1.** DFT-Calculated Reaction Energy Profile and Chemoselectivity of C–O Bond Activation of [Pd(dppb)]-Catalyzed Decarbonylative Reduction of Benzoic Pivalic Anhydride.

**Zero-point correction (*ZPE*), thermal correction to enthalpy (*TCH*), thermal correction to Gibbs free energy (*TCG*), energies (*E*), enthalpies (*H*), and Gibbs free energies (*G*) (in Hartree) of the structures calculated at the M06/6-311+G(d,p)-SDD-SMD(Toluene)//B3LYP/6-31G(d)-LANL2DZ level of theory**

**Table S1. Energies in Figure 4 and Figure S1**

| Structures | *ZPE* | *TCH* | *TCG* | *E* | *H* | *G* | Imaginary Frequency |
|---|---|---|---|---|---|---|---|
| 3 | 0.720759 | 0.768242 | 0.631103 | -2585.092371 | -2584.324129 | -2584.461268 | |
| TS4 | 0.719659 | 0.766234 | 0.634892 | -2585.072334 | -2584.306100 | -2584.437442 | 219.5i |
| 5 | 0.721010 | 0.768049 | 0.634402 | -2585.100817 | -2584.332768 | -2584.466415 | |
| TS6 | 0.718107 | 0.765364 | 0.633830 | -2585.060556 | -2584.295192 | -2584.426726 | 260.8i |
| 7 | 0.718602 | 0.766500 | 0.633911 | -2585.072991 | -2584.306491 | -2584.439080 | |
| 8 | 0.711322 | 0.756264 | 0.629801 | -2471.779777 | -2471.023513 | -2471.149976 | |
| 9 | 0.831046 | 0.886180 | 0.731145 | -2881.551404 | -2880.665224 | -2880.820259 | |
| TS10 | 0.828282 | 0.883128 | 0.729129 | -2881.529706 | -2880.646578 | -2880.800577 | 919.8i |
| 11 | 0.833425 | 0.888712 | 0.734614 | -2881.556798 | -2880.668086 | -2880.822184 | |
| 12 | 0.733844 | 0.781796 | 0.649904 | -2649.471642 | -2648.689846 | -2648.821738 | |
| TS13 | 0.732509 | 0.780056 | 0.648280 | -2649.452972 | -2648.672916 | -2648.804692 | 124.4i |
| 14 | 0.249260 | 0.266352 | 0.206201 | -755.549136 | -755.282784 | -755.342935 | |
| 15 | 0.721399 | 0.768208 | 0.637380 | -2585.101623 | -2584.333415 | -2584.464243 | |
| 16 | 0.830704 | 0.885809 | 0.731071 | -2881.545477 | -2880.659668 | -2880.814406 | |
| TS17 | 0.828893 | 0.883599 | 0.730731 | -2881.516577 | -2880.632978 | -2880.785846 | 973.1i |
| 18 | 0.217793 | 0.233743 | 0.175120 | -829.309232 | -829.075489 | -829.134112 | |
| CO | 0.005036 | 0.008341 | -0.014102 | -113.285648 | -113.277307 | -113.299750 | |
| TMSH | 0.119745 | 0.128198 | 0.089359 | -409.783842 | -409.655644 | -409.694483 | |
| PhH | 0.100796 | 0.106129 | 0.073333 | -232.120317 | -232.014188 | -232.046984 | |
| PhCOOPiv | 0.237677 | 0.253450 | 0.194130 | -691.144382 | -690.890932 | -690.950252 | |
| *t*BuH | 0.132408 | 0.139032 | 0.104796 | -158.363808 | -158.224776 | -158.259012 | |
| TS-S1 | 0.719887 | 0.766362 | 0.635731 | -2585.069734 | -2584.303372 | -2584.434003 | 198.2i |
| TS-S2 | 0.718469 | 0.765678 | 0.633441 | -2585.047117 | -2584.281439 | -2584.413676 | 139.5i |
| S3 | 0.719471 | 0.767050 | 0.635753 | -2585.062328 | -2584.295278 | -2584.426575 | |
| S4 | 0.711941 | 0.756474 | 0.632064 | -2471.771481 | -2471.015007 | -2471.139417 | |
| S5 | 0.702022 | 0.749103 | 0.617400 | -2723.232032 | -2722.482929 | -2722.614632 | |
| TS-S6 | 0.700850 | 0.747420 | 0.616027 | -2723.215181 | -2722.467761 | -2722.599154 | 139.4i |

## Mechanistic Studies Referred to from the Main Manuscript

A series of mechanistic studies were performed to gain insight into the reaction mechanism and investigate factors involved in controlling the Pd-catalyzed decarbonylative step-down reduction of carboxylic acids (Figures S2-S7).

(1) To investigate whether benzoic pivalic anhydride was a possible reaction intermediate, 4-Ph-benzoic pivalic anhydride **1S** was prepared and subjected to the reaction conditions (Figure S2). Formation of product **2a** from **1S** was observed using Pd-catalysis, suggesting that **1S** could be a competent intermediate. Moreover, 4-Ph-benzoic acetic anhydride served as a competent intermediate under Pd-catalyzed conditions (83% yield, not shown).

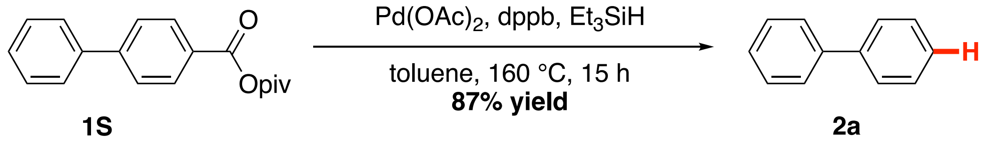


**Figure S2. Decarbonylative step-down reduction of 4-Ph-benzoic pivalic anhydride.** *Conditions: **1S** (1.0 equiv), $Et_3SiH$ (1.5 equiv), $Pd(OAc)_2$ (5 mol%), dppb (10 mol%), toluene (0.20 M), 160 °C, 15 h.*

(2) To investigate electronic effect on the decarbonylative step-down reduction, a Hammett correlation study employing differently substituted 4-Ar-benzoic acids was conducted (Figure S3). The study showed a large positive ρ-value of 1.57 ($R^2$ = 0.99), which can be compared with the $\rho^+$-value of 0.94 ($R^2$ = 0.92) using Hammett-Brown $\sigma^+$ constants, suggesting that electron-deficient arenes are inherently more reactive substrates, consistent with facility of metal insertion and decarbonylation.

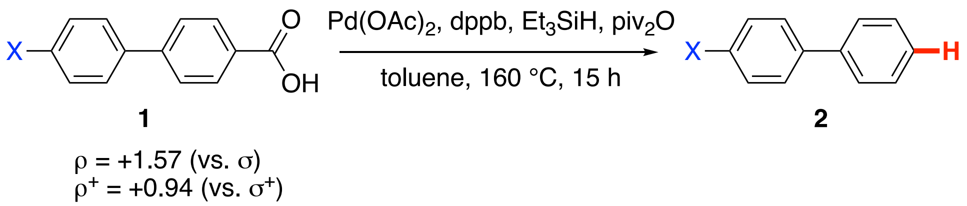

| entry | X | $k_X/k_H$[a] | Hammett σ constant | Hammett σ+ constant |
|---|---|---|---|---|
| 1 | MeO | 0.38 | -0.268 | -0.778 |
| 2 | Me | 0.67 | -0.17 | -0.311 |
| 3 | H | 1.00 | 0 | 0 |
| 4 | F | 1.12 | 0.062 | -0.073 |
| 5 | $CF_3$ | 7.70 | 0.54 | 0.612 |

[a]Relative reactivity values determined from product distribution by $^1$H NMR and/or GC/MS of crude reaction mixtures. The correlation using σ constants (Y = 1.568X + 0.017, $R^2$ = 0.989) can be compared with the correlation obtained using $\sigma^+$ Hammett constants (Y = 0.938 + 0.171, $R^2$ = 0.924), indicating stabilization of the negative charge in the transition state of the reaction.

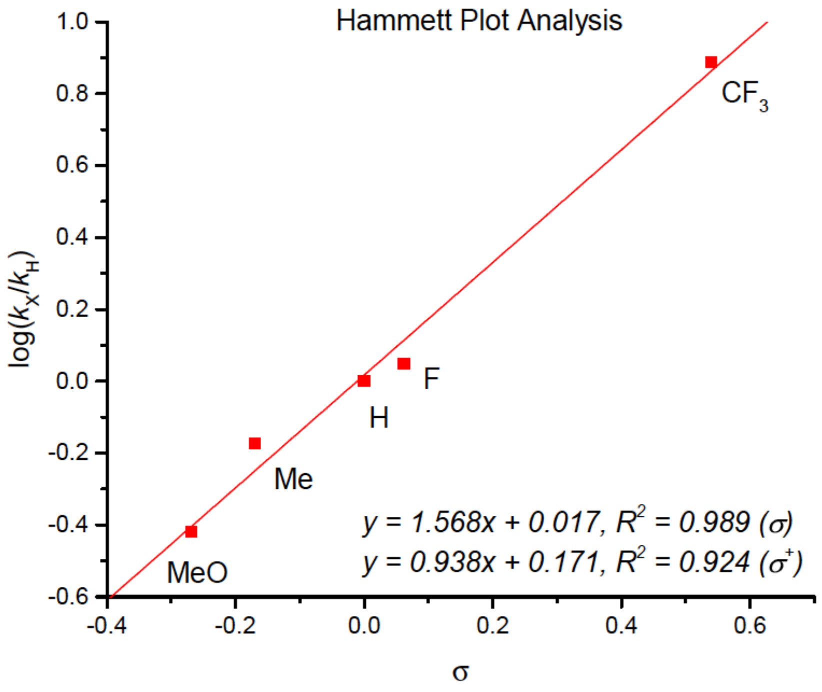


**Figure S3. Hammett study in decarbonylative step-down reduction of carboxylic acids.** *Conditions:* ***1*** *(1.0 equiv each), $Et_3SiH$ (0.5 equiv), $Pd(OAc)_2$ (5 mol%), dppb (10 mol%), $piv_2O$ (1.5 equiv), toluene (0.20 M), 160 °C, 15 h.*

(3) To investigate steric effect on the decarbonylative step-down reduction, intermolecular competition experiments between differently substituted carboxylic acids were conducted

(Figure S4). The experiments revealed that sterically-hindered carboxylic acids react preferentially, consistent with decarbonylation favored by steric demand of acylpalladium complexes.

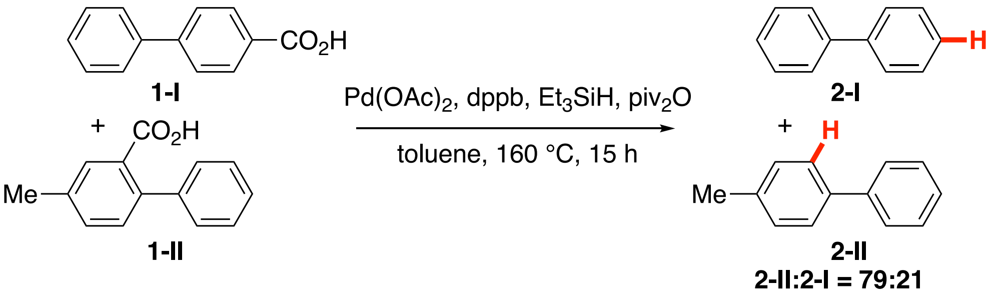


**Figure S4. Intermolecular competition experiments in decarbonylative step-down reduction of carboxylic acids.** *Conditions: **1** (1.0 equiv each), $Et_3SiH$ (0.5 equiv), $Pd(OAc)_2$ (5 mol%), dppb (10 mol%), $piv_2O$ (1.5 equiv), toluene (0.20 M), 160 °C, 15 h.*

(4) To gain additional insight, relative reactivity studies regarding the use of biaryls, conjugated arenes and conjugated vinyl-arenes were conducted (Figure S5). The experiments revealed the following order of reactivity: biaryl = Np > vinyl-Ar.

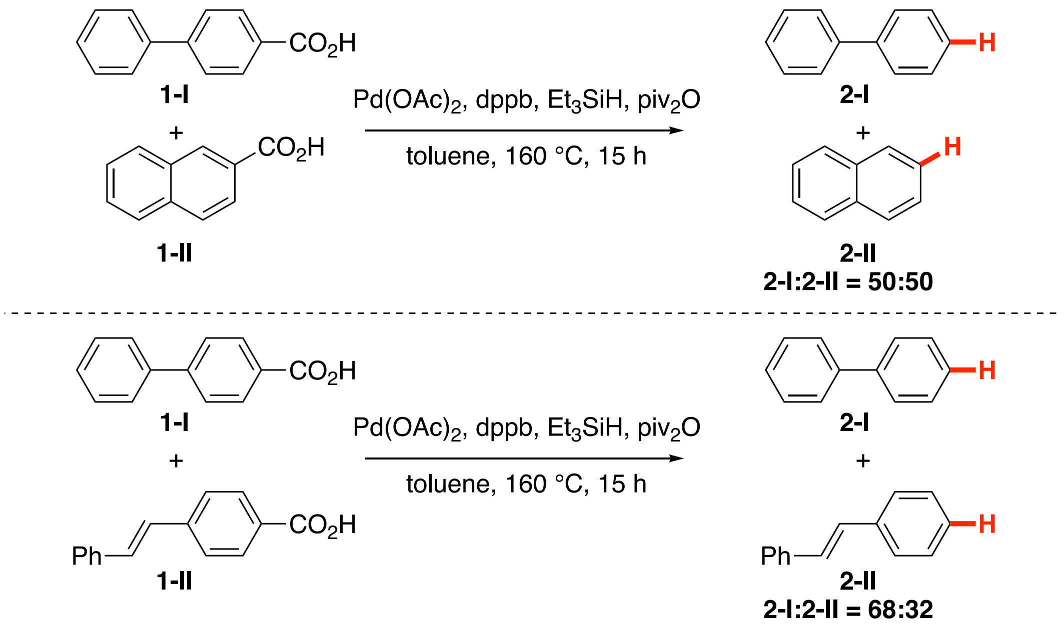

**Figure S5. Intermolecular competition experiments in decarbonylative step-down reduction of carboxylic acids.** *Conditions: **1** (1.0 equiv each), $Et_3SiH$ (0.5 equiv), $Pd(OAc)_2$ (5 mol%), dppb (10 mol%), $piv_2O$ (1.5 equiv), toluene (0.20 M), 160 °C, 15 h.*

(5) To investigate the reduction selectivity in the decarbonylative step-down reduction, experiments at different temperatures were conducted (Figure S6). The formation of aldehyde reduction products was not detected.

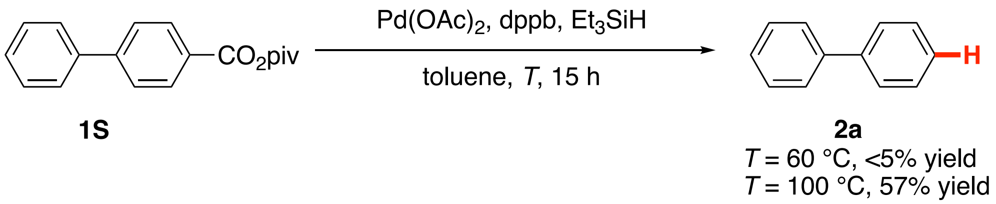


**Figure S6. Decarbonylative step-down reduction of 4-Ph-benzoic pivalic anhydride.** *Conditions: **1S** (1.0 equiv), $Et_3SiH$ (1.5 equiv), $Pd(OAc)_2$ (5 mol%), dppb (10 mol%), toluene (0.20 M), T, 15 h.*

(6) To investigate the effect of low catalytic loading on the decarbonylative step-down reduction, the reduction was conducted at 0.10 mol% of $Pd(OAc)_2$ (Figure S7). Formation of product **2a** from **1a** was observed without a noticeable decrease in yield, consistent with the high efficiency of the reduction. Note that the reduction at low loading is also possible using $Ac_2O$ (51% yield), albeit with a decreased reaction efficiency.

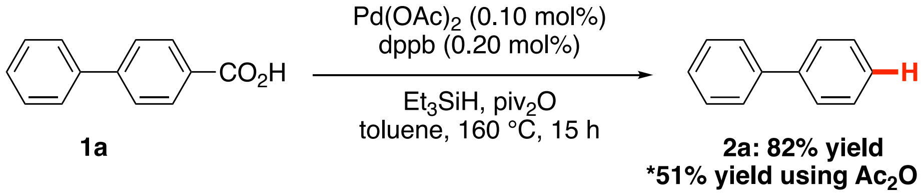


**Figure S7. Decarbonylative step-down reduction of carboxylic acids at low catalyst loading.** *Conditions: **1** (1.0 equiv), $Et_3SiH$ (1.5 equiv), $Pd(OAc)_2$ (0.10 mol%), dppb (0.20 mol%), $piv_2O$ (1.5 equiv), toluene, 160 °C, 15 h.*

## General Information

All starting materials reported in the manuscript are commercially available or have been previously described in literature. All experiments involving palladium were performed using standard Schlenk techniques under argon atmosphere unless stated otherwise. All solvents were purchased at the highest commercial grade and used as received or after purification by passing through activated alumina columns or distillation from sodium/benzophenone under nitrogen. All solvents were deoxygenated prior to use. All other chemicals were purchased at the highest commercial grade and used as received. Reaction glassware was oven-dried at 140 °C for at least 24 h or flame-dried prior to use, allowed to cool under vacuum and purged with argon (three cycles). All products were identified using $^1$H NMR analysis and comparison with authentic samples. GC and/or GC/MS analysis was used for volatile products. All yields refer to yields determined by $^1$H NMR and/or GC or GC/MS using an internal standard (optimization) and isolated yields (preparative runs) unless stated otherwise. $^1$H NMR and $^{13}$C NMR spectra were recorded in $CDCl_3$ on Bruker spectrometers at 500 ($^1$H NMR) and 125 MHz ($^{13}$C NMR). All shifts are reported in parts per million (ppm) relative to residual $CHCl_3$ peak (7.27 and 77.2 ppm, $^1$H NMR and $^{13}$C NMR, respectively). All coupling constants (*J*) are reported in hertz (Hz). Abbreviations are: s, singlet; d, doublet; t, triplet; q, quartet; brs, broad singlet. GC-MS chromatography was performed using Agilent HP6890 GC System and Agilent 5973A inert XL EI/CI MSD using helium as the carrier gas at a flow rate of 1 mL/min and an initial oven temperature of 50 °C. The injector temperature was 250 °C. The detector temperature was 250 °C. For runs with the initial oven temperature of 50 °C, temperature was increased with a 10 °C/min ramp after 50 °C hold for 3 min to a final temperature of 220 °C, then hold at 220 °C for 15 min (splitless mode of injection, total run time of 22.0 min). High-resolution mass spectra (HRMS) were measured on a 7T Bruker Daltonics FT-MS instrument (for HRMS). Melting point was measured on MeltEMP (laboratory devices). All flash chromatography was performed using silica gel, 60 Å, 300 mesh. TLC analysis was carried out on glass plates coated with silica gel 60 F254, 0.2 mm thickness. The plates were visualized using a 254 nm ultraviolet lamp or aqueous potassium permanganate solutions. $^1$H NMR and $^{13}$C NMR data are given for all compounds in Supporting Materials. $^1$H NMR, $^{13}$C NMR and HRMS data are reported for all new compounds.

## Table S2. Optimization of Decarbonylative Reduction

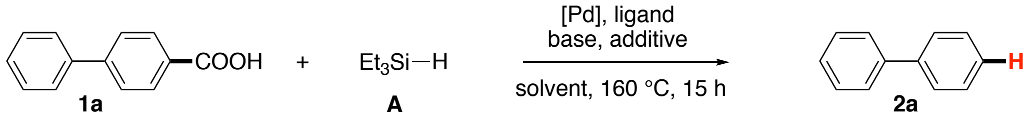


| Entry | [Pd] | ligand | base | additive | solvent | **1a:A:base:additive** | Yield[a] |
|---|---|---|---|---|---|---|---|
| 1 | $Pd(OAc)_2$ | DPPB | $Et_3N$ | $Piv_2O$ | dioxane | 1 : 1.5 : 1.5 : 1.5 | 77% |
| 2 | $Pd(OAc)_2$ | DPPB | $Et_3N$ | $Piv_2O$ | dioxane | 1 : 2.0 : 1.5 : 1.5 | 73% |
| 3 | $Pd(OAc)_2$ | DPPB | $Et_3N$ | $Piv_2O$ | dioxane | 1 : 3.0 : 1.5 : 1.5 | 73% |
| 4 | $Pd(OAc)_2$ | DPPB | $Et_3N$ | $Piv_2O$ | dioxane | 1 : 2.0 : 1.2 : 1.2 | 71% |
| 5 | $Pd(OAc)_2$ | DPPB | $Et_3N$ | $Piv_2O$ | dioxane | 1 : 3.0 : 1.2 : 1.2 | 73% |
| 6[b] | $Pd(OAc)_2$ | DPPB | $Et_3N$ | $Piv_2O$ | dioxane | 1 : 1.5 : 1.5 : 1.5 | 65% |
| 7[c] | $Pd(OAc)_2$ | DPPB | $Et_3N$ | $Piv_2O$ | dioxane | 1 : 1.5 : 1.5 : 1.5 | 45% |
| 8[d] | $Pd(OAc)_2$ | DPPB | $Et_3N$ | $Piv_2O$ | dioxane | 1 : 1.5 : 1.5 : 1.5 | 42% |
| 9 | $Pd(OAc)_2$ | DPPB | $Et_3N$ | $Piv_2O$ | toluene | 1 : 1.5 : 1.5 : 1.5 | >98% |
| 10 | $Pd(OAc)_2$ | DPPB | $Et_3N$ | $Ac_2O$ | toluene | 1 : 1.5 : 1.5 : 1.5 | 89% |
| 11 | -- | -- | -- | -- | toluene | 1 : 1.5 : 0 : 0 | <2% |
| 12 | -- | -- | $Et_3N$ | -- | toluene | 1 : 1.5 : 1.5 : 0 | <2% |
| 13 | -- | -- | -- | $Piv_2O$ | toluene | 1 : 1.5 : 0 : 1.5 | <2% |
| 14 | -- | -- | $Et_3N$ | $Piv_2O$ | toluene | 1 : 1.5 : 1.5 : 1.5 | <2% |
| 15 | $Pd(OAc)_2$ | DPPB | -- | -- | toluene | 1 : 1.5 : 0 : 0 | <2% |
| 16 | $Pd(OAc)_2$ | DPPB | $Et_3N$ | -- | toluene | 1 : 1.5 : 1.5 : 0 | <2% |
| 17 | $Pd(OAc)_2$ | -- | -- | $Piv_2O$ | toluene | 1 : 1.5 : 0 : 1.5 | <2% |
| 18 | $Pd(OAc)_2$ | DPPB | -- | $Piv_2O$ | toluene | 1 : 1.5 : 0 : 1.5 | >98% |
| 19[b] | $Pd(OAc)_2$ | DPPB | -- | $Piv_2O$ | toluene | 1 : 1.5 : 0 : 1.5 | 98% |
| 20[c] | $Pd(OAc)_2$ | DPPB | -- | $Piv_2O$ | toluene | 1 : 1.5 : 0 : 1.5 | 62% |
| 21[d] | $Pd(OAc)_2$ | DPPB | -- | $Piv_2O$ | toluene | 1 : 1.5 : 0 : 1.5 | 20% |
| 22 | $Pd(OAc)_2$ | DPPB | -- | $Ac_2O$ | toluene | 1 : 1.5 : 0 : 1.5 | 90% |
| 23 | $Pd(OAc)_2$ | DPPB | -- | AcCl | toluene | 1 : 1.5 : 0 : 1.5 | <2% |
| 24 | $Pd(OAc)_2$ | DPPB | -- | PivCl | toluene | 1 : 1.5 : 0 : 1.5 | <2% |
| 25 | $Pd(OAc)_2$ | DPPB | -- | $Piv_2O$ | toluene | 1 : 1.2 : 0 : 1.2 | 95% |
| 26[e] | $Pd(OAc)_2$ | DPPB | -- | $Piv_2O$ | toluene | 1 : 1.5 : 0 : 1.5 | 78% |
| 27[f] | $Pd(OAc)_2$ | DPPB | -- | $Piv_2O$ | toluene | 1 : 1.5 : 0 : 1.5 | 98% |
| 28[g] | $Pd(OAc)_2$ | DPPB | -- | $Piv_2O$ | toluene | 1 : 1.5 : 0 : 1.5 | 84% |
| 29[h] | $Pd(OAc)_2$ | DPPB | -- | $Piv_2O$ | toluene | 1 : 1.5 : 0 : 1.5 | 63% |
| 30 | $Pd(OAc)_2$ | DPPM | -- | $Piv_2O$ | toluene | 1 : 1.5 : 0 : 1.5 | 58% |
| 31 | $Pd(OAc)_2$ | DPPE | -- | $Piv_2O$ | toluene | 1 : 1.5 : 0 : 1.5 | 95% |
| 32 | $Pd(OAc)_2$ | DPPP | -- | $Piv_2O$ | toluene | 1 : 1.5 : 0 : 1.5 | 97% |
| 33 | $Pd(OAc)_2$ | DPPPent | -- | $Piv_2O$ | toluene | 1 : 1.5 : 0 : 1.5 | 91% |
| 34 | $Pd(OAc)_2$ | DPPHex | -- | $Piv_2O$ | toluene | 1 : 1.5 : 0 : 1.5 | 48% |
| 35 | $Pd(OAc)_2$ | BINAP | -- | $Piv_2O$ | toluene | 1 : 1.5 : 0 : 1.5 | 83% |
| 36 | $Pd(OAc)_2$ | DPEPhos | -- | $Piv_2O$ | toluene | 1 : 1.5 : 0 : 1.5 | 97% |
| 37 | $Pd(OAc)_2$ | XantPhos | -- | $Piv_2O$ | toluene | 1 : 1.5 : 0 : 1.5 | 80% |
| 38 | $Pd(OAc)_2$ | DPPF | -- | $Piv_2O$ | toluene | 1 : 1.5 : 0 : 1.5 | 85% |
| 39[i] | $Pd(OAc)_2$ | $PCy_3HBF_4$ | -- | $Piv_2O$ | toluene | 1 : 1.5 : 0 : 1.5 | 31% |
| 40[i] | $Pd(OAc)_2$ | $PPh_3$ | -- | $Piv_2O$ | toluene | 1 : 1.5 : 0 : 1.5 | 45% |

Conditions: **1a** (1 equiv), $Pd(OAc)_2$ (5 mol%), ligand (10 mol%), solvent (0.20 M), 160 °C, 15 h. [a]Determined by $^1H$ NMR and/or GC-MS. [b]140 °C. [c]120 °C. [d]100 °C. [e]$Pd(OAc)_2$ (0.25 mol%), DPPB (0.5 mol%). [f]$HMe_2Si\text{-}O\text{-}SiMe_2H$. [g]$Et_2SiH_2$. [h]$Ph_2SiH_2$. [i]ligand (20 mol%).

## Experimental Procedures and Characterization Data

**General Procedure for Decarbonylative Reduction of Carboxylic Acids.** An oven-dried vial equipped with a stir bar was charged with carboxylic acid (neat, 1.0 equiv), $Pd(OAc)_2$ (typically, 5 mol%), ligand (typically, 10 mol%), triethylsilane (typically, 1.5 equiv) and trimethylacetic anhydride (1.5 equiv), placed under a positive pressure of argon, and subjected to three evacuation/backfilling cycles under high vacuum. Toluene (0.20 M) was added with vigorous stirring at room temperature, the reaction mixture was placed in a preheated oil bath at 160 °C, and stirred for the indicated time at 160 °C. After the indicated time, the reaction mixture was cooled down to room temperature, diluted with $CH_2Cl_2$ (10 mL), filtered, and concentrated. Purification by chromatography on silica gel (EtOAc/hexanes) afforded the title product.

**Representative Procedure for Decarbonylative Reduction of Carboxylic Acids. 1.0 g Scale Reaction.** An oven-dried vial equipped with a stir bar was charged with [1,1'-biphenyl]-4-carboxylic acid (neat, 1.0 g, 5.05 mmol), $Pd(OAc)_2$ (0.011 g, 0.0505 mmol, 1 mol%), 1,4-bis(diphenylphosphino)butane (0.043 g, 0.101 mmol, 2 mol%), triethylsilane (0.881 g, 7.58 mmol, 1.5 equiv), triethylamine (0.77 g, 7.58 mmol, 1.5 equiv), and trimethylacetic anhydride (1.41 g, 7.58 mmol, 1.5 equiv), placed under a positive pressure of argon, and subjected to three evacuation/backfilling cycles under high vacuum. Toluene (25 mL, 0.20 M) was added with vigorous stirring at room temperature, the reaction mixture was placed in a preheated oil bath at 160 °C, and stirred for 15 h at 160 °C. After the indicated time, the reaction mixture was cooled down to room temperature, diluted with $CH_2Cl_2$ (50 mL), filtered, and concentrated. Purification by chromatography on silica gel (EtOAc/hexanes) afforded the title product. Yield 97% (0.753 g). White solid. Characterization data are included in the section below.

## Characterization Data of Reduction Products

**[1,1'-Biphenyl]-4-carboxylic acid (1a, Figure 2A, Entry 1)**

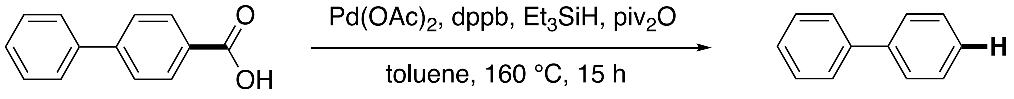


**1a** **2a**

According to the general procedure, the reaction of [1,1'-biphenyl]-4-carboxylic acid (5.05 mmol), $Pd(OAc)_2$ (1 mol%), 1,4-bis(diphenylphosphino)butane (2 mol%), triethylsilane (1.5 equiv), triethylamine (1.5 equiv) and trimethylacetic anhydride (1.5 equiv) in toluene (0.20 M) for 15 h at 160 °C, afforded after work-up and chromatography the title compound in 97% yield (0.753 g). White solid. **$^1$H NMR (500 MHz, $CDCl_3$)** δ 7.64-7.62 (d, *J* = 7.3 Hz, 4 H), 7.49-7.46 (t, *J* = 7.5 Hz, 4 H), 7.40-7.37 (t, *J* = 7.4 Hz, 2 H). **$^{13}$C NMR (125 MHz, $CDCl_3$)** δ 141.26, 128.77, 127.27, 127.19. The spectral data matched those reported in the literature.[11]

**[1,1'-Biphenyl]-2-carboxylic acid (1b, Figure 2A, Entry 2)**

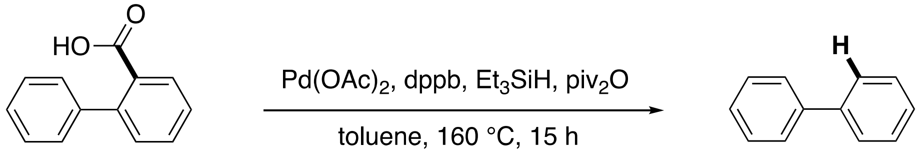


**1b** **2a**

According to the general procedure, the reaction of [1,1'-biphenyl]-2-carboxylic acid (0.20 mmol), $Pd(OAc)_2$ (5 mol%), 1,4-bis(diphenylphosphino)butane (10 mol%), triethylsilane (1.5 equiv) and trimethylacetic anhydride (1.5 equiv) in toluene (0.20 M) for 15 h at 160 °C, afforded after work-up and chromatography the title compound in 98% yield (30.3 mg). White solid. **$^1$H NMR (500 MHz, $CDCl_3$)** δ 7.64-7.62 (d, *J* = 7.3 Hz, 4 H), 7.49-7.46 (t, *J* = 7.5 Hz, 4 H), 7.40-7.37 (t, *J* = 7.4 Hz, 2 H). **$^{13}$C NMR (125 MHz, $CDCl_3$)** δ 141.26, 128.77, 127.27, 127.19. The spectral data matched those reported in the literature.[11]

**(*E*)-4-Styrylbenzoic acid (1c, Figure 2A, Entry 3)**

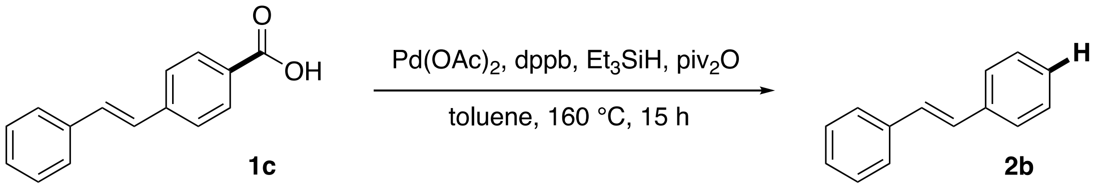


According to the general procedure, the reaction of (*E*)-4-styrylbenzoic acid (0.20 mmol), $Pd(OAc)_2$ (5 mol%), 1,4-bis(diphenylphosphino)butane (10 mol%), triethylsilane (1.5 equiv) and trimethylacetic anhydride (1.5 equiv) in toluene (0.20 M) for 15 h at 160 °C, afforded after work-up and chromatography the title compound in 93% yield (33.6 mg). White solid. **$^1$H NMR (500 MHz, $CDCl_3$)** δ 7.56-7.54 (d, *J* = 7.5 Hz, 4 H), 7.41-7.38 (t, *J* = 7.5 Hz, 4 H), 7.31-7.28 (t, *J* = 7.4 Hz, 2 H), 7.14 (s, 2 H). **$^{13}$C NMR (125 MHz, $CDCl_3$)** δ 137.35, 128.71, 128.70, 127.64, 126.53. The spectral data matched those reported in the literature.[11]

**(*E*)-2-Styrylbenzoic acid (1d, Figure 2A, Entry 4)**

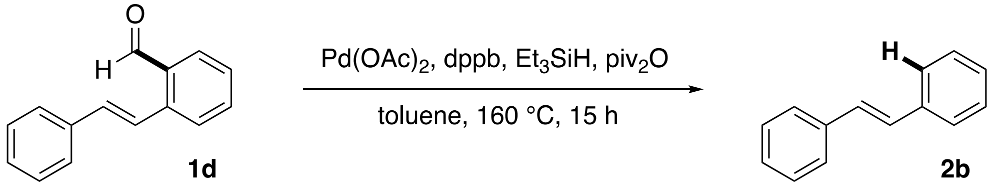


According to the general procedure, the reaction of (*E*)-2-styrylbenzoic acid (0.20 mmol), $Pd(OAc)_2$ (5 mol%), 1,4-bis(diphenylphosphino)butane (10 mol%), triethylsilane (1.5 equiv) and trimethylacetic anhydride (1.5 equiv) in toluene (0.20 M) for 15 h at 160 °C, afforded after work-up and chromatography the title compound in 96% yield (34.6 mg). White solid. **$^1$H NMR (500 MHz, $CDCl_3$)** δ 7.56-7.54 (d, *J* = 7.5 Hz, 4 H), 7.41-7.38 (t, *J* = 7.5 Hz, 4 H), 7.31-7.28 (t, *J* = 7.4 Hz, 2 H), 7.14 (s, 2 H). **$^{13}$C NMR (125 MHz, $CDCl_3$)** δ 137.35, 128.71, 128.70, 127.64, 126.53. The spectral data matched those reported in the literature.[11]

**4'-Methoxy-[1,1'-biphenyl]-4-carboxylic acid (1e, Figure 2A, Entry 5)**

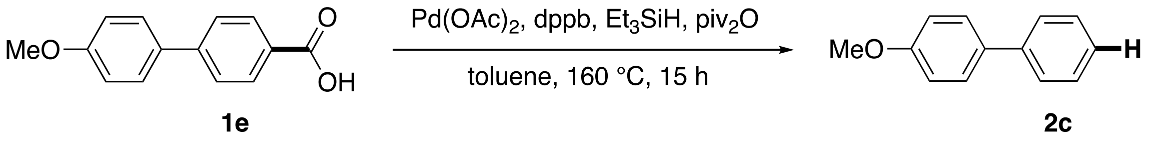


According to the general procedure, the reaction of 4'-methoxy-[1,1'-biphenyl]-4-carboxylic acid (0.20 mmol), $Pd(OAc)_2$ (5 mol%), 1,4-bis(diphenylphosphino)butane (10 mol%), triethylsilane (1.5 equiv) and trimethylacetic anhydride (1.5 equiv) in toluene (0.20 M) for 15 h at 160 °C, afforded after work-up and chromatography the title compound in 90% yield (33.2 mg). White solid. **$^1$H NMR (500 MHz, $CDCl_3$)** δ 7.59-7.55 (m, 4 H), 7.46-7.43 (t, *J* = 7.6 Hz, 2 H), 7.34-7.31 (t, *J* = 7.4 Hz, 1 H), 7.02-7.00 (d, *J* = 8.7 Hz, 2 H), 3.88 (s, 3 H). **$^{13}$C NMR (125 MHz, $CDCl_3$)** δ 159.15, 140.85, 133.80, 128.73, 128.17, 126.76, 126.67, 114.21, 55.37. The spectral data matched those reported in the literature.[12]

**2'-Methoxy-[1,1'-biphenyl]-2-carboxylic acid (1f, Figure 2A, Entry 6)**

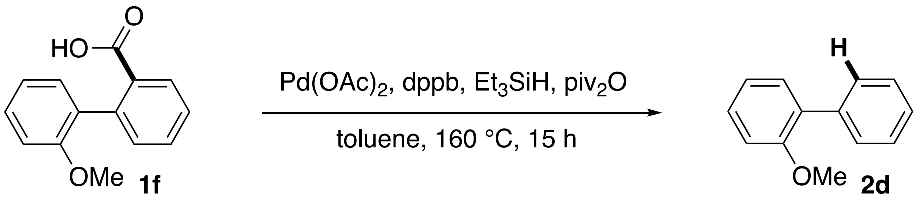


According to the general procedure, the reaction of 2'-methoxy-[1,1'-biphenyl]-2-carboxylic acid (0.20 mmol), $Pd(OAc)_2$ (5 mol%), 1,4-bis(diphenylphosphino)butane (10 mol%), triethylsilane (1.5 equiv) and trimethylacetic anhydride (1.5 equiv) in toluene (0.20 M) for 15 h at 160 °C, afforded after work-up and chromatography the title compound in 92% yield (33.9 mg). White solid. **$^1$H NMR (500 MHz, $CDCl_3$)** δ 7.58-7.57 (d, *J* = 7.6 Hz, 2 H), 7.47-7.44 (t, *J* = 7.5 Hz, 2 H), 7.38-7.35 (t, *J* = 7.1 Hz, 3 H), 7.09-7.06 (t, *J* = 7.5 Hz, 1 H), 7.04-7.02 (d, *J* = 8.6 Hz, 1 H), 3.85 (s, 3 H). **$^{13}$C NMR (125 MHz, $CDCl_3$)** δ 156.49, 138.57, 130.92, 130.75, 129.57, 128.64, 128.01, 126.94, 120.85, 111.25, 55.58. The spectral data matched those reported in the literature.[12]

**4'-(Trifluoromethyl)-[1,1'-biphenyl]-4-carboxylic acid (1g, Figure 2A, Entry 7)**

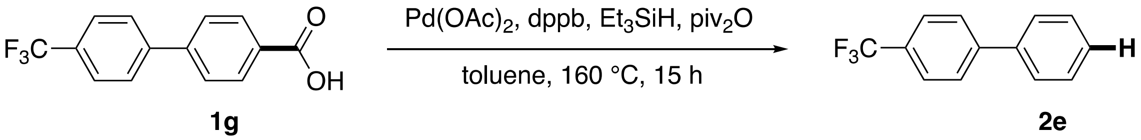


According to the general procedure, the reaction of 4'-(trifluoromethyl)-[1,1'-biphenyl]-4-carboxylic acid (0.20 mmol), $Pd(OAc)_2$ (5 mol%), 1,4-bis(diphenylphosphino)butane (10 mol%), triethylsilane (1.5 equiv) and trimethylacetic anhydride (1.5 equiv) in toluene (0.20 M) for 15 h at 160 °C, afforded after work-up and chromatography the title compound in 97% yield (43.1 mg). White solid. **$^1$H NMR (500 MHz, $CDCl_3$)** δ 7.73 (s, 4 H), 7.64-7.63 (d, $J$ = 7.6 Hz, 2 H), 7.52-7.49 (t, $J$ = 7.5 Hz, 2 H), 7.46-7.43 (t, $J$ = 7.4 Hz, 1 H). **$^{13}$C NMR (125 MHz, $CDCl_3$)** δ 144.75, 139.79, 129.36 (q, $J^F$ = 32.3 Hz), 129.01, 128.21, 127.44, 127.30, 125.73 (q, $J^F$ = 3.7 Hz), 124.34 (q, $J^F$ = 270.2 Hz). **$^{19}$F NMR (471 MHz, $CDCl_3$)** δ -62.39. The spectral data matched those reported in the literature.[13]

**2'-(Trifluoromethyl)-[1,1'-biphenyl]-2-carboxylic acid (1h, Figure 2A, Entry 8)**

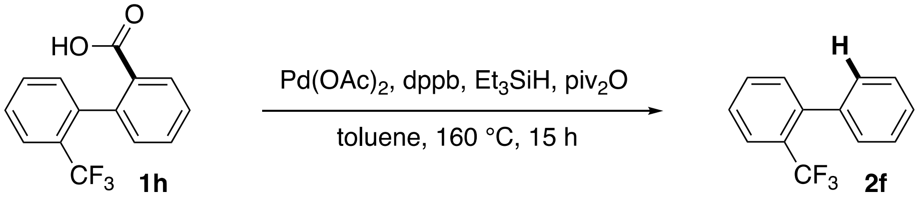


According to the general procedure, the reaction of 2'-(trifluoromethyl)-[1,1'-biphenyl]-2-carboxylic acid (0.20 mmol), $Pd(OAc)_2$ (5 mol%), 1,4-bis(diphenylphosphino)butane (10 mol%), triethylsilane (1.5 equiv) and trimethylacetic anhydride (1.5 equiv) in toluene (0.20 M) for 15 h at 160 °C, afforded after work-up and chromatography the title compound in 95% yield (42.3 mg). White solid. **$^1$H NMR (500 MHz, $CDCl_3$)** δ 7.79-7.78 (d, $J$ = 7.8 Hz, 1 H), 7.60-7.58 (t, $J$ = 7.3 Hz, 1 H), 7.51-7.48 (t, $J$ = 7.6 Hz, 1 H), 7.43 (s, 3 H), 7.37 (s, 3 H). **$^{13}$C NMR (125 MHz, $CDCl_3$)** δ 141.45 (q, $J^F$ = 1.9 Hz), 139.87, 132.04, 131.28, 128.96 (q, $J^F$ = 1.4 Hz), 128.49 (q, $J^F$ = 29.6 Hz), 127.74, 127.61, 127.32, 126.05 (q, $J^F$ = 5.3 Hz), 124.18 (q, $J^F$ = 272.2 Hz). **$^{19}$F NMR (471 MHz, $CDCl_3$)** δ -56.83. The spectral data matched those reported in the literature.[14]

**1-Naphthoic acid (1i, Figure 2A, Entry 9)**

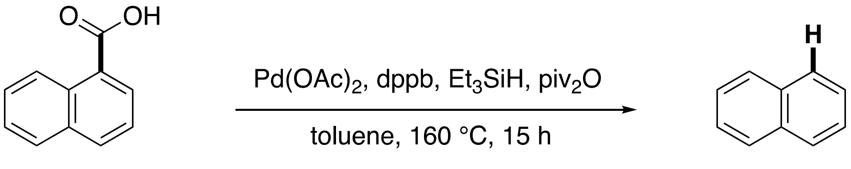


**1i** **2g**

According to the general procedure, the reaction of 1-naphthoic acid (0.20 mmol), $Pd(OAc)_2$ (5 mol%), 1,4-bis(diphenylphosphino)butane (10 mol%), triethylsilane (1.5 equiv) and trimethylacetic anhydride (1.5 equiv) in toluene (0.20 M) for 15 h at 160 °C, afforded after work-up and chromatography the title compound in 97% yield (24.9 mg). White solid. **$^{1}$H NMR (500 MHz, $CDCl_3$)** δ 7.93-7.91 (m, 4 H), 7.56-7.54 (m, 4 H). **$^{13}$C NMR (125 MHz, $CDCl_3$)** δ 133.53, 127.96, 125.89. The spectral data matched those reported in the literature.[11]

**2-Naphthoic acid (1j, Figure 2A, Entry 10)**

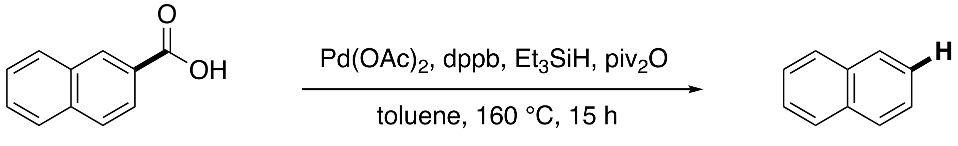


**1j** **2g**

According to the general procedure, the reaction of 2-naphthoic acid (0.20 mmol), $Pd(OAc)_2$ (5 mol%), 1,4-bis(diphenylphosphino)butane (10 mol%), triethylsilane (1.5 equiv) and trimethylacetic anhydride (1.5 equiv) in toluene (0.20 M) for 15 h at 160 °C, afforded after work-up and chromatography the title compound in 95% yield (24.4 mg). White solid. **$^{1}$H NMR (500 MHz, $CDCl_3$)** δ 7.93-7.91 (m, 4 H), 7.56-7.54 (m, 4 H). **$^{13}$C NMR (125 MHz, $CDCl_3$)** δ 133.53, 127.96, 125.89. The spectral data matched those reported in the literature.[11]

**6-Methoxy-2-naphthoic acid (1k, Figure 2A, Entry 11)**

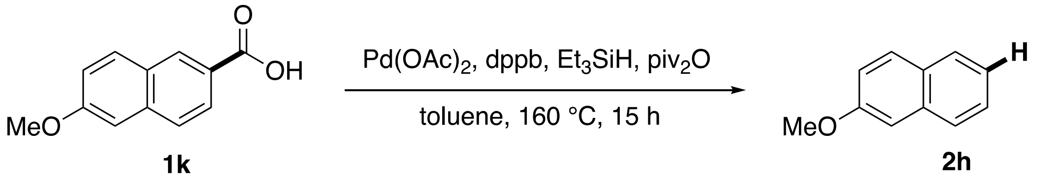


According to the general procedure, the reaction of 6-methoxy-2-naphthoic acid (0.20 mmol), $Pd(OAc)_2$ (5 mol%), 1,4-bis(diphenylphosphino)butane (10 mol%), triethylsilane (1.5 equiv) and trimethylacetic anhydride (1.5 equiv) in toluene (0.20 M) for 15 h at 160 °C, afforded after work-up and chromatography the title compound in 92% yield (29.1 mg). White solid. **$^{1}$H NMR (500 MHz, $CDCl_3$)** δ 7.80-7.76 (m, 3 H), 7.48-7.45 (t, *J* = 7.1 Hz, 1 H), 7.37-7.35 (t, *J* = 7.2 Hz, 1 H), 7.19-7.17 (m, 2 H), 3.95 (s, 3 H). **$^{13}$C NMR (125 MHz, $CDCl_3$)** δ 157.61, 134.58, 129.40, 128.97, 127.67, 126.75, 126.38, 123.60, 118.73, 105.76, 55.31. The spectral data matched those reported in the literature.[11]

**4-(Naphthalen-2-yl)benzoic acid (1l, Figure 2A, Entry 12)**

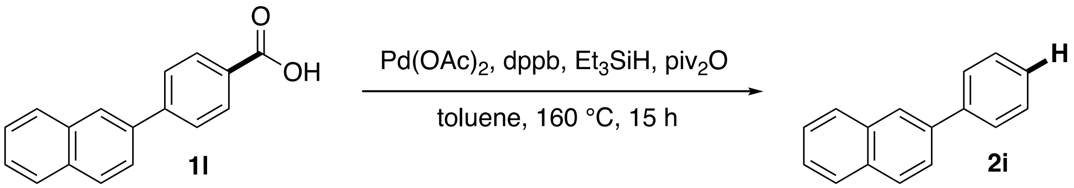


According to the general procedure, the reaction of 4-(naphthalen-2-yl)benzoic acid (0.20 mmol), $Pd(OAc)_2$ (5 mol%), 1,4-bis(diphenylphosphino)butane (10 mol%), triethylsilane (1.5 equiv) and trimethylacetic anhydride (1.5 equiv) in toluene (0.20 M) for 15 h at 160 °C, afforded after work-up and chromatography the title compound in 77% yield (31.5 mg). White solid. **$^{1}$H NMR (500 MHz, $CDCl_3$)** δ 8.08 (s, 1 H), 7.96-7.92 (t, *J* = 8.8 Hz, 2 H), 7.91-7.89 (d, *J* = 7.8 Hz, 1 H), 7.79-7.75 (m, 3 H), 7.55-7.50 (m, 4 H), 7.43-7.40 (t, *J* = 7.3 Hz, 1 H). **$^{13}$C NMR (125 MHz, $CDCl_3$)** δ 141.15, 138.58, 133.69, 132.63, 128.87, 128.42, 128.21, 127.66, 127.45, 127.37, 126.30, 125.94, 125.82, 125.61. The spectral data matched those reported in the literature.[13]

**4-Hydroxybenzoic acid (1m, Figure 2A, Entry 13)**

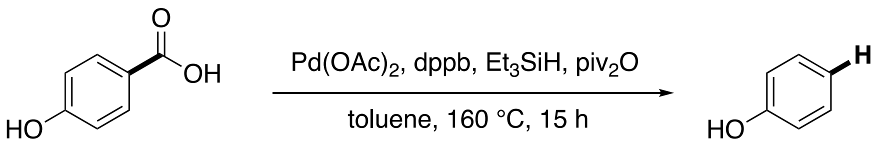


According to the general procedure, the reaction of 4-hydroxybenzoic acid (0.20 mmol), $Pd(OAc)_2$ (5 mol%), 1,4-bis(diphenylphosphino)butane (10 mol%), triethylsilane (1.5 equiv) and trimethylacetic anhydride (1.5 equiv) in toluene (0.20 M) for 15 h at 160 °C, afforded after work-up and chromatography the title compound in 68% yield (12.8 mg). White solid. **$^1$H NMR (500 MHz, $CDCl_3$)** δ 7.30-7.27 (t, *J* = 7.7 Hz, 2 H), 6.99-6.96 (t, *J* = 7.4 Hz, 1 H), 6.88-6.87 (d, *J* = 8.2 Hz, 2 H), 4.99 (s, 1 H). **$^{13}$C NMR (125 MHz, $CDCl_3$)** δ 155.40, 129.73, 120.89, 115.34. The spectral data matched those reported in the literature.[15]

**2-Hydroxybenzoic acid (1n, Figure 2A, Entry 14)**

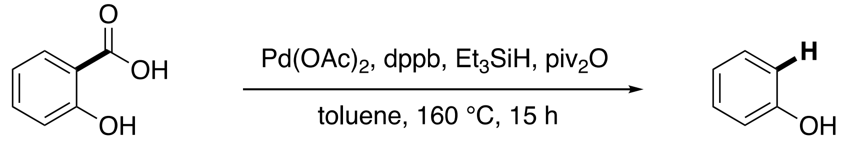


According to the general procedure, the reaction of 2-hydroxybenzoic acid (0.20 mmol), $Pd(OAc)_2$ (5 mol%), 1,4-bis(diphenylphosphino)butane (10 mol%), triethylsilane (1.5 equiv) and trimethylacetic anhydride (1.5 equiv) in toluene (0.20 M) for 15 h at 160 °C, afforded after work-up and chromatography the title compound in 60% yield (11.3 mg). White solid. **$^1$H NMR (500 MHz, $CDCl_3$)** δ 7.30-7.27 (t, *J* = 7.7 Hz, 2 H), 6.99-6.96 (t, *J* = 7.4 Hz, 1 H), 6.88-6.87 (d, *J* = 8.2 Hz, 2 H), 4.99 (s, 1 H). **$^{13}$C NMR (125 MHz, $CDCl_3$)** δ 155.40, 129.73, 120.89, 115.34. The spectral data matched those reported in the literature.[15]

**4-Methoxybenzoic acid (1o, Figure 2A, Entry 15)**

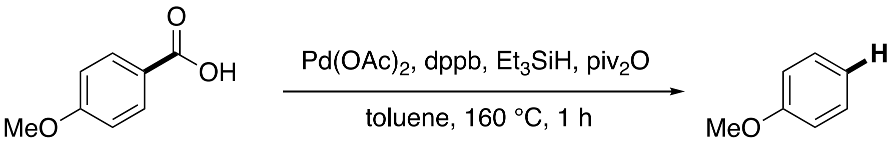


**1o** **2k**

According to the general procedure, the reaction of 4-methoxybenzoic acid (0.20 mmol), $Pd(OAc)_2$ (5 mol%), 1,4-bis(diphenylphosphino)butane (10 mol%), triethylsilane (1.5 equiv) and trimethylacetic anhydride (1.5 equiv) in toluene (0.20 M) for 1 h at 160 °C, afforded after work-up and chromatography the title compound in 58% yield (12.6 mg). White solid. **$^1$H NMR (500 MHz, $CDCl_3$)** δ 7.34-7.31 (t, *J* = 7.6 Hz, 2 H), 6.99-6.97 (t, *J* = 7.3 Hz, 1 H), 6.95-6.93 (d, *J* = 8.3 Hz, 2 H), 3.84 (s, 3 H). **$^{13}$C NMR (125 MHz, $CDCl_3$)** δ 159.56, 129.46, 120.66, 113.90, 55.15. The spectral data matched those reported in the literature.[11]

**2-Methoxybenzoic acid (1p, Figure 2A, Entry 16)**

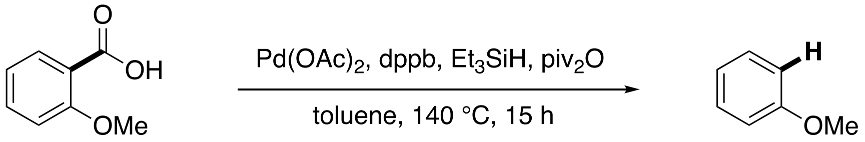


**1p** **2k**

According to the general procedure, the reaction of 2-methoxybenzoic acid (0.20 mmol), $Pd(OAc)_2$ (5 mol%), 1,4-bis(diphenylphosphino)butane (10 mol%), triethylsilane (1.5 equiv) and trimethylacetic anhydride (1.5 equiv) in toluene (0.20 M) for 15 h at 140 °C, afforded after work-up and chromatography the title compound in 89% yield (19.3 mg). White solid. **$^1$H NMR (500 MHz, $CDCl_3$)** δ 7.34-7.31 (t, *J* = 7.6 Hz, 2 H), 6.99-6.97 (t, *J* = 7.3 Hz, 1 H), 6.95-6.93 (d, *J* = 8.3 Hz, 2 H), 3.84 (s, 3 H). **$^{13}$C NMR (125 MHz, $CDCl_3$)** δ 159.56, 129.46, 120.66, 113.90, 55.15. The spectral data matched those reported in the literature.[11]

**4-(Dimethylamino)benzoic acid (1q, Figure 2A, Entry 17)**

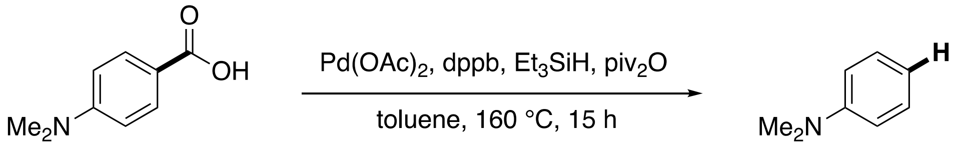


**1q** **2l**

According to the general procedure, the reaction of 4-(dimethylamino)benzoic acid (0.20 mmol), $Pd(OAc)_2$ (5 mol%), 1,4-bis(diphenylphosphino)butane (10 mol%), triethylsilane (1.5 equiv) and trimethylacetic anhydride (1.5 equiv) in toluene (0.20 M) for 15 h at 160 °C, afforded after work-up and chromatography the title compound in 48% yield (11.7 mg). White solid. **$^{1}$H NMR (500 MHz, $CDCl_3$)** δ 7.31-7.28 (d, $J$ = 8.1 Hz, 2 H), 6.81-6.76 (m, 3 H), 2.99 (s, 6 H). **$^{13}$C NMR (125 MHz, $CDCl_3$)** δ 150.69, 129.10, 116.65, 112.69, 40.65. The spectral data matched those reported in the literature.[16]

**3-(Dimethylamino)benzoic acid (1r, Figure 2A, Entry 18)**

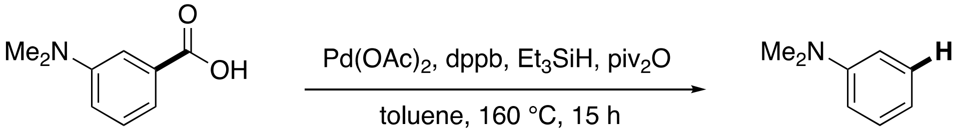


**1r** **2l**

According to the general procedure, the reaction of 3-(dimethylamino)benzoic acid (0.20 mmol), $Pd(OAc)_2$ (5 mol%), 1,4-bis(diphenylphosphino)butane (10 mol%), triethylsilane (1.5 equiv) and trimethylacetic anhydride (1.5 equiv) in toluene (0.20 M) for 15 h at 160 °C, afforded after work-up and chromatography the title compound in 86% yield (20.9 mg). White solid. **$^{1}$H NMR (500 MHz, $CDCl_3$)** δ 7.31-7.28 (d, $J$ = 8.1 Hz, 2 H), 6.81-6.76 (m, 3 H), 2.99 (s, 6 H). **$^{13}$C NMR (125 MHz, $CDCl_3$)** δ 150.69, 129.10, 116.65, 112.69, 40.65. The spectral data matched those reported in the literature.[16]

**4-Cyanobenzoic acid (1s, Figure 2A, Entry 19)**

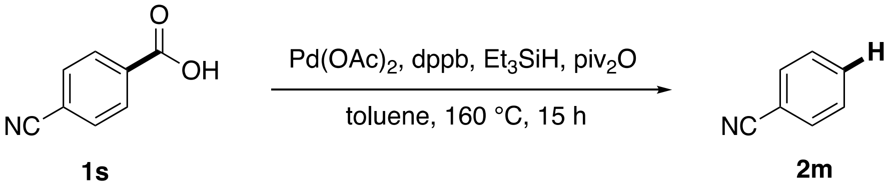

According to the general procedure, the reaction of 4-cyanobenzoic acid (0.20 mmol), $Pd(OAc)_2$ (5 mol%), 1,4-bis(diphenylphosphino)butane (10 mol%), triethylsilane (1.5 equiv) and trimethylacetic anhydride (1.5 equiv) in toluene (0.20 M) for 15 h at 160 °C, afforded after work-up and chromatography the title compound in 91% yield (18.8 mg). White solid. **$^1$H NMR (500 MHz, $CDCl_3$)** δ 7.68-7.67 (d, $J$ = 8.3 Hz, 2 H), 7.64-7.61 (t, $J$ = 7.6 Hz, 1 H), 7.51-7.48 (t, $J$ = 7.9 Hz, 2 H). **$^{13}$C NMR (125 MHz, $CDCl_3$)** δ 132.80, 132.17, 129.15, 118.87, 112.46. The spectral data matched those reported in the literature.[17]

**3-Cyanobenzoic acid (1t, Figure 2A, Entry 20)**

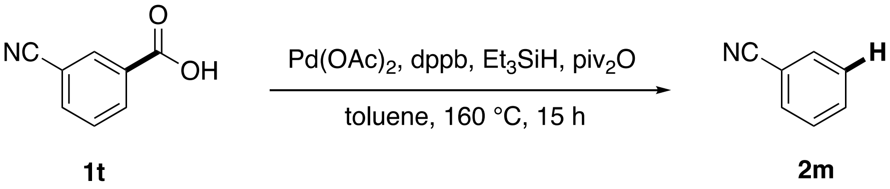

According to the general procedure, the reaction of 3-cyanobenzoic acid (0.20 mmol), $Pd(OAc)_2$ (5 mol%), 1,4-bis(diphenylphosphino)butane (10 mol%), triethylsilane (1.5 equiv) and trimethylacetic anhydride (1.5 equiv) in toluene (0.20 M) for 15 h at 160 °C, afforded after work-up and chromatography the title compound in 88% yield (18.2 mg). White solid. **$^1$H NMR (500 MHz, $CDCl_3$)** δ 7.68-7.67 (d, $J$ = 8.3 Hz, 2 H), 7.64-7.61 (t, $J$ = 7.6 Hz, 1 H), 7.51-7.48 (t, $J$ = 7.9 Hz, 2 H). **$^{13}$C NMR (125 MHz, $CDCl_3$)** δ 132.80, 132.17, 129.15, 118.87, 112.46. The spectral data matched those reported in the literature.[17]

**4-(Methoxycarbonyl)benzoic acid (1u, Figure 2A, Entry 21)**

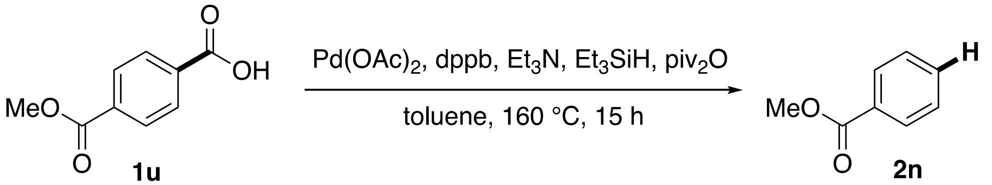


According to the general procedure, the reaction of 4-(methoxycarbonyl)benzoic acid (0.20 mmol), $Pd(OAc)_2$ (5 mol%), 1,4-bis(diphenylphosphino)butane (10 mol%), triethylamine (1.5 equiv), triethylsilane (1.5 equiv) and trimethylacetic anhydride (1.5 equiv) in toluene (0.20 M) for 15 h at 160 °C, afforded after work-up and chromatography the title compound in 74% yield (20.2 mg). White solid. **$^1$H NMR (500 MHz, $CDCl_3$)** δ 8.08-8.06 (d, *J* = 7.6 Hz, 2 H), 7.59-7.57 (t, *J* = 7.4 Hz, 1 H), 7.48-7.45 (t, *J* = 7.7 Hz, 2 H), 3.94 (s, 3 H). **$^{13}$C NMR (125 MHz, $CDCl_3$)** δ 167.14, 132.92, 130.18, 129.58, 128.37, 52.11. The spectral data matched those reported in the literature.[11]

**3-(Methoxycarbonyl)benzoic acid (1v, Figure 2A, Entry 22)**

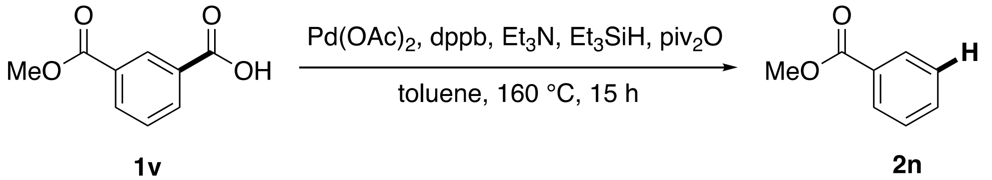


According to the general procedure, the reaction of 3-(methoxycarbonyl)benzoic acid (0.20 mmol), $Pd(OAc)_2$ (5 mol%), 1,4-bis(diphenylphosphino)butane (10 mol%), triethylamine (1.5 equiv), triethylsilane (1.5 equiv) and trimethylacetic anhydride (1.5 equiv) in toluene (0.20 M) for 15 h at 160 °C, afforded after work-up and chromatography the title compound in 85% yield (23.2 mg). White solid. **$^1$H NMR (500 MHz, $CDCl_3$)** δ 8.08-8.06 (d, *J* = 7.6 Hz, 2 H), 7.59-7.57 (t, *J* = 7.4 Hz, 1 H), 7.48-7.45 (t, *J* = 7.7 Hz, 2 H), 3.94 (s, 3 H). **$^{13}$C NMR (125 MHz, $CDCl_3$)** δ 167.14, 132.92, 130.18, 129.58, 128.37, 52.11. The spectral data matched those reported in the literature.[11]

**4-Acetylbenzoic acid (1w, Figure 2A, Entry 23)**

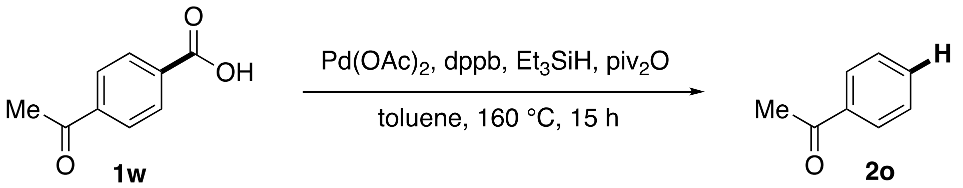


According to the general procedure, the reaction of 4-acetylbenzoic acid (0.20 mmol), $Pd(OAc)_2$ (5 mol%), 1,4-bis(diphenylphosphino)butane (10 mol%), triethylsilane (1.5 equiv) and trimethylacetic anhydride (1.5 equiv) in toluene (0.20 M) for 15 h at 160 °C, afforded after work-up and chromatography the title compound in 95% yield (22.9 mg). White solid. **$^1$H NMR (500 MHz, $CDCl_3$)** δ 7.99-7.97 (d, *J* = 7.4 Hz, 2 H), 7.60-7.57 (t, *J* = 7.4 Hz, 1 H), 7.50-7.47 (t, *J* = 7.8 Hz, 2 H), 2.63 (s, 3 H). **$^{13}$C NMR (125 MHz, $CDCl_3$)** δ 198.18, 137.14, 133.12, 128.59, 128.32, 26.63. The spectral data matched those reported in the literature.[18]

**3-Acetylbenzoic acid (1x, Figure 2A, Entry 24)**

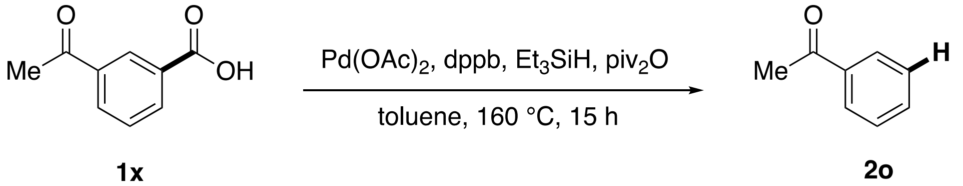


According to the general procedure, the reaction of 3-acetylbenzoic acid (0.20 mmol), $Pd(OAc)_2$ (5 mol%), 1,4-bis(diphenylphosphino)butane (10 mol%), triethylsilane (1.5 equiv) and trimethylacetic anhydride (1.5 equiv) in toluene (0.20 M) for 15 h at 160 °C, afforded after work-up and chromatography the title compound in 88% yield (21.2 mg). White solid. **$^1$H NMR (500 MHz, $CDCl_3$)** δ 7.99-7.97 (d, *J* = 7.4 Hz, 2 H), 7.60-7.57 (t, *J* = 7.4 Hz, 1 H), 7.50-7.47 (t, *J* = 7.8 Hz, 2 H), 2.63 (s, 3 H). **$^{13}$C NMR (125 MHz, $CDCl_3$)** δ 198.18, 137.14, 133.12, 128.59, 128.32, 26.63. The spectral data matched those reported in the literature.[18]

**4-Formylbenzoic acid (1y, Figure 2A, Entry 25)**

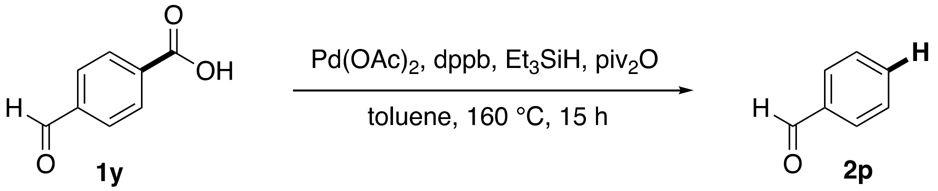

According to the general procedure, the reaction of 4-formylbenzoic acid (0.20 mmol), $Pd(OAc)_2$ (5 mol%), 1,4-bis(diphenylphosphino)butane (10 mol%), triethylsilane (1.5 equiv) and trimethylacetic anhydride (1.5 equiv) in toluene (0.20 M) for 15 h at 160 °C, afforded after work-up and chromatography the title compound in 90% yield (19.1 mg). White solid. **$^1$H NMR (500 MHz, $CDCl_3$)** δ 10.05 (s, 1 H), 7.92-7.90 (d, *J* = 8.3 Hz, 2 H), 7.68-7.65 (t, *J* = 7.3 Hz, 1 H), 7.58-7.55 (t, *J* = 7.7 Hz, 2 H). **$^{13}$C NMR (125 MHz, $CDCl_3$)** δ 192.42, 136.42, 134.48, 129.77, 129.02. The spectral data matched those reported in the literature.[19]

**4-(Tosyloxy)benzoic acid (1z, Figure 2A, Entry 26)**

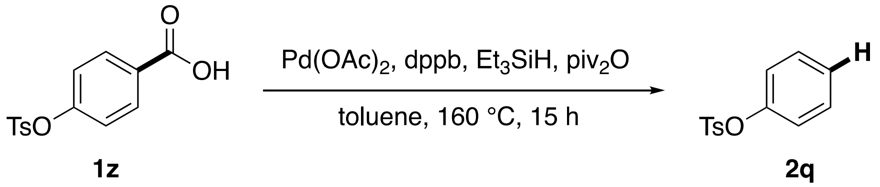

According to the general procedure, the reaction of 4-(tosyloxy)benzoic acid (0.20 mmol), $Pd(OAc)_2$ (5 mol%), 1,4-bis(diphenylphosphino)butane (10 mol%), triethylsilane (1.5 equiv) and trimethylacetic anhydride (1.5 equiv) in toluene (0.20 M) for 15 h at 160 °C, afforded after work-up and chromatography the title compound in 96% yield (47.7 mg). White solid. **$^1$H NMR (500 MHz, $CDCl_3$)** δ 7.73-7.72 (d, *J* = 7.6 Hz, 2 H), 7.33-7.25 (m, 5 H), 7.01-7.00 (d, *J* = 7.8 Hz, 2 H), 2.47 (s, 3 H). **$^{13}$C NMR (125 MHz, $CDCl_3$)** δ 149.67, 145.33, 132.44, 129.74, 129.61, 128.54, 127.09, 122.41, 21.73. The spectral data matched those reported in the literature.[20]

**4-Acetoxybenzoic acid (1aa, Figure 2A, Entry 27)**

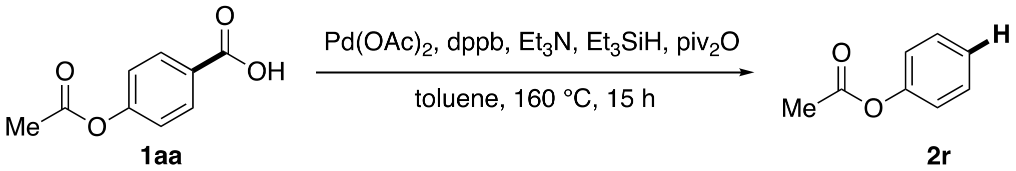


According to the general procedure, the reaction of 4-acetoxybenzoic acid (0.20 mmol), $Pd(OAc)_2$ (5 mol%), 1,4-bis(diphenylphosphino)butane (10 mol%), triethylamine (1.5 equiv), triethylsilane (1.5 equiv) and trimethylacetic anhydride (1.5 equiv) in toluene (0.20 M) for 15 h at 160 °C, afforded after work-up and chromatography the title compound in 61% yield (16.7 mg). White solid. **$^1$H NMR (500 MHz, $CDCl_3$)** δ 7.42-7.39 (t, *J* = 7.7 Hz, 2 H), 7.27-7.24 (t, *J* = 7.4 Hz, 1 H), 7.12-7.11 (d, *J* = 8.6 Hz, 2 H), 2.33 (s, 3 H). **$^{13}$C NMR (125 MHz, $CDCl_3$)** δ 169.49, 150.72, 129.44, 125.84, 121.59, 21.15. The spectral data matched those reported in the literature.[21]

**4-(Benzoyloxy)benzoic acid (1ab, Figure 2A, Entry 28)**

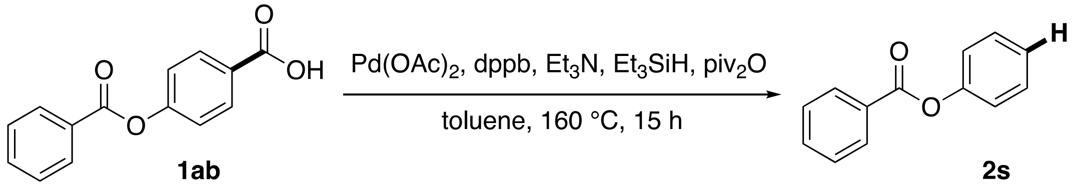


According to the general procedure, the reaction of 4-(benzoyloxy)benzoic acid (0.20 mmol), $Pd(OAc)_2$ (5 mol%), 1,4-bis(diphenylphosphino)butane (10 mol%), triethylamine (1.5 equiv), triethylsilane (1.5 equiv) and trimethylacetic anhydride (1.5 equiv) in toluene (0.20 M) for 15 h at 160 °C, afforded after work-up and chromatography the title compound in 76% yield (30.2 mg). White solid. **$^1$H NMR (500 MHz, $CDCl_3$)** δ 8.25-8.24 (d, *J* = 8.0 Hz, 2 H), 7.69-7.66 (t, *J* = 7.3 Hz, 1 H), 7.56-7.53 (t, *J* = 7.7 Hz, 2 H), 7.48-7.45 (t, *J* = 7.8 Hz, 2 H), 7.32-7.29 (t, *J* = 7.4 Hz, 1 H), 7.26-7.25 (d, *J* = 8.2 Hz, 2 H). **$^{13}$C NMR (125 MHz, $CDCl_3$)** δ 165.21, 150.99, 133.60, 130.20, 129.62, 129.52, 128.59, 125.91, 121.74. The spectral data matched those reported in the literature.[22]

**4-(2-Oxopyrrolidin-1-yl)benzoic acid (1ac, Figure 2A, Entry 29)**

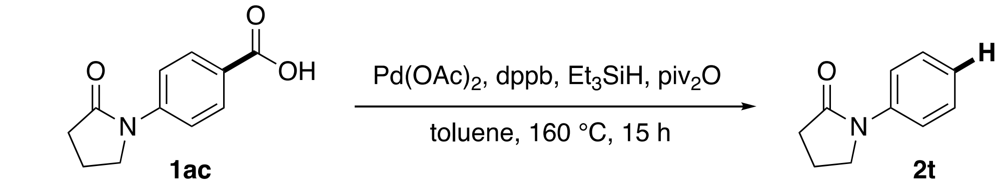


According to the general procedure, the reaction of 4-(2-oxopyrrolidin-1-yl)benzoic acid (0.20 mmol), $Pd(OAc)_2$ (5 mol%), 1,4-bis(diphenylphosphino)butane (10 mol%), triethylsilane (1.5 equiv) and trimethylacetic anhydride (1.5 equiv) in toluene (0.20 M) for 15 h at 160 °C, afforded after work-up and chromatography the title compound in 72% yield (23.3 mg). White solid. **$^1$H NMR (500 MHz, $CDCl_3$)** δ 7.64-7.62 (d, *J* = 8.0 Hz, 2 H), 7.41-7.38 (t, *J* = 7.9 Hz, 2 H), 7.18-7.15 (t, *J* = 7.4 Hz, 1 H), 3.91-3.88 (t, *J* = 7.0 Hz, 2 H), 2.65-2.62 (t, *J* = 8.1 Hz, 2 H), 2.22-2.16 (m, 2 H). **$^{13}$C NMR (125 MHz, $CDCl_3$)** δ 174.23, 139.42, 128.84, 124.52, 119.98, 48.80, 32.78, 18.06. The spectral data matched those reported in the literature.[23]

**4-Benzamidobenzoic acid (1ad, Figure 2A, Entry 30)**

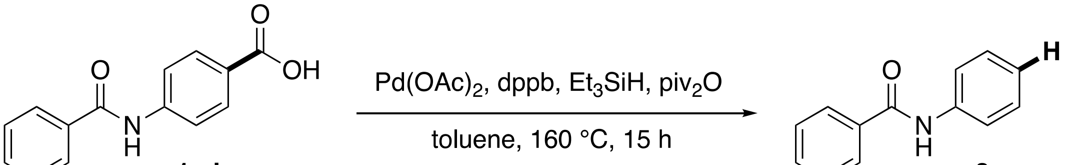


According to the general procedure, the reaction of 4-benzamidobenzoic acid (0.20 mmol), $Pd(OAc)_2$ (5 mol%), 1,4-bis(diphenylphosphino)butane (10 mol%), triethylsilane (1.5 equiv) and trimethylacetic anhydride (1.5 equiv) in toluene (0.20 M) for 15 h at 160 °C, afforded after work-up and chromatography the title compound in 62% yield (24.5 mg). White solid. **$^1$H NMR (500 MHz, $CDCl_3$)** δ 7.90-7.89 (d, *J* = 7.6 Hz, 3 H), 7.68-7.66 (d, *J* = 8.1 Hz, 2 H), 7.59-7.56 (t, *J* = 7.3 Hz, 1 H), 7.52-7.49 (t, *J* = 7.7 Hz, 2 H), 7.41-7.38 (t, *J* = 7.7 Hz, 2 H), 7.20-7.17 (t, *J* = 7.4 Hz, 1 H). **$^{13}$C NMR (125 MHz, $CDCl_3$)** δ 165.75, 137.94, 135.03, 131.87, 129.13, 128.82, 127.03, 124.60, 120.21. The spectral data matched those reported in the literature.[24]

**4-(Methyl(phenyl)amino)benzoic acid (1ae, Figure 2A, Entry 31)**

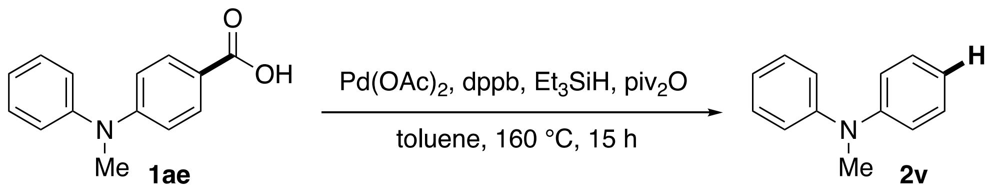


According to the general procedure, the reaction of 4-(methyl(phenyl)amino)benzoic acid (0.20 mmol), $Pd(OAc)_2$ (5 mol%), 1,4-bis(diphenylphosphino)butane (10 mol%), triethylsilane (1.5 equiv) and trimethylacetic anhydride (1.5 equiv) in toluene (0.20 M) for 15 h at 160 °C, afforded after work-up and chromatography the title compound in 46% yield (16.9 mg). White solid. **$^1$H NMR (500 MHz, $CDCl_3$)** δ 7.32-7.29 (t, *J* = 8.1 Hz, 4 H), 7.06-7.04 (d, *J* = 8.1 Hz, 4 H), 7.00-6.97 (t, *J* = 7.3 Hz, 2 H), 3.35 (s, 3 H). **$^{13}$C NMR (125 MHz, $CDCl_3$)** δ 149.05, 129.20, 121.26, 120.45, 40.25. The spectral data matched those reported in the literature.[25]

**Flufenamic acid (1af, Figure 2A, Entry 32)**

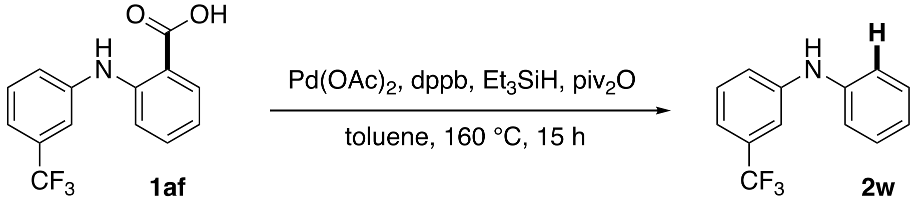


According to the general procedure, the reaction of flufenamic acid (0.20 mmol), $Pd(OAc)_2$ (5 mol%), 1,4-bis(diphenylphosphino)butane (10 mol%), triethylsilane (1.5 equiv) and trimethylacetic anhydride (1.5 equiv) in toluene (0.20 M) for 15 h at 160 °C, afforded after work-up and chromatography the title compound in 97% yield (46.1 mg). White solid. **$^1$H NMR (500 MHz, $CDCl_3$)** δ 7.38-7.33 (m, 3 H), 7.29 (s, 1 H), 7.22-7.21 (d, *J* = 8.4 Hz, 1 H), 7.16-7.13 (t, *J* = 8.2 Hz, 3 H), 7.06-7.03 (t, *J* = 7.4 Hz, 1 H), 5.85 (s, 1 H). **$^{13}$C NMR (125 MHz, $CDCl_3$)** δ 144.06, 141.78, 131.78 (q, $J^F$ = 31.8 Hz), 129.84, 129.59, 124.11 (q, $J^F$ = 270.7 Hz), 122.37, 119.74, 119.06, 116.94 (q, $J^F$ = 3.8 Hz), 113.23 (q, $J^F$ = 3.9 Hz). **$^{19}$F NMR (471 MHz, $CDCl_3$)** δ -62.87. The spectral data matched those reported in the literature.[26]

**4'-Chloro-[1,1'-biphenyl]-4-carboxylic acid (1ag, Figure 2A, Entry 33)**

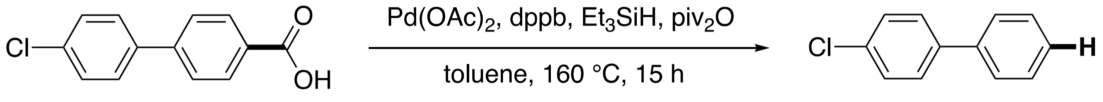


According to the general procedure, the reaction of 4'-chloro-[1,1'-biphenyl]-4-carboxylic acid (0.20 mmol), $Pd(OAc)_2$ (5 mol%), 1,4-bis(diphenylphosphino)butane (10 mol%), triethylsilane (1.5 equiv) and trimethylacetic anhydride (1.5 equiv) in toluene (0.20 M) for 15 h at 160 °C, afforded after work-up and chromatography the title compound in 96% yield (36.3 mg). White solid. **$^1$H NMR (500 MHz, $CDCl_3$)** δ 7.60-7.58 (d, *J* = 7.5 Hz, 2 H), 7.56-7.55 (d, *J* = 8.4 Hz, 2 H), 7.50-7.44 (m, 4 H), 7.42-7.39 (t, *J* = 7.3 Hz, 1 H). **$^{13}$C NMR (125 MHz, $CDCl_3$)** δ 140.03, 139.70, 133.41, 128.94, 128.91, 128.42, 127.62, 127.02. The spectral data matched those reported in the literature.[27]

**Quinoline-6-carboxylic acid (1ah, Figure 2A, Entry 34)**

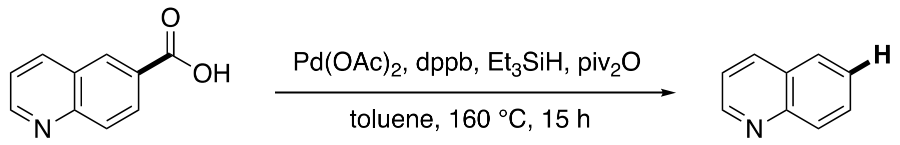


According to the general procedure, the reaction of quinoline-6-carboxylic acid (0.20 mmol), $Pd(OAc)_2$ (5 mol%), 1,4-bis(diphenylphosphino)butane (10 mol%), triethylsilane (1.5 equiv) and trimethylacetic anhydride (1.5 equiv) in toluene (0.20 M) for 15 h at 160 °C, afforded after work-up and chromatography the title compound in 92% yield (23.8 mg). White solid. **$^1$H NMR (500 MHz, $CDCl_3$)** δ 10.24 (s, 1 H), 9.09 (s, 1 H), 8.39-8.36 (m, 2 H), 8.26-8.22 (m, 2 H), 7.57-7.55 (m, 1 H). **$^{13}$C NMR (125 MHz, $CDCl_3$)** δ 153.15, 150.94, 137.50, 134.37, 133.64, 130.87, 127.75, 126.80, 122.25. The spectral data matched those reported in the literature.[28]

**1-Methyl-1*H*-indole-3-carboxylic acid (1ai, Figure 2A, Entry 35)**

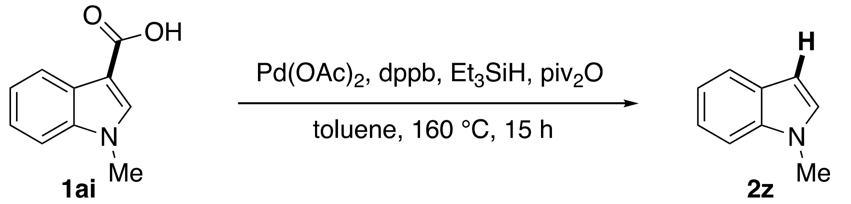


According to the general procedure, the reaction of 1-methyl-1*H*-indole-3-carboxylic acid (0.20 mmol), $Pd(OAc)_2$ (5 mol%), 1,4-bis(diphenylphosphino)butane (10 mol%), triethylsilane (1.5 equiv) and trimethylacetic anhydride (1.5 equiv) in toluene (0.20 M) for 15 h at 160 °C, afforded after work-up and chromatography the title compound in 62% yield (16.3 mg). White solid. **$^1$H NMR (500 MHz, $CDCl_3$)** δ 10.06 (s, 1 H), 8.18 (s, 1 H), 7.84-7.82 (d, *J* = 8.6 Hz, 1 H), 7.44-7.43 (d, *J* = 8.5 Hz, 1 H), 7.18-7.18 (d, *J* = 3.0 Hz, 1 H), 6.68-6.68 (d, *J* = 2.9 Hz, 1 H), 3.88 (s, 3 H). **$^{13}$C NMR (125 MHz, $CDCl_3$)** δ 139.96, 130.73, 129.32, 128.23, 126.47, 121.85, 109.79, 103.27, 33.16. The spectral data matched those reported in the literature.[29]

**1-Methyl-1*H*-indole-5-carboxylic acid (1aj, Figure 2A, Entry 36)**

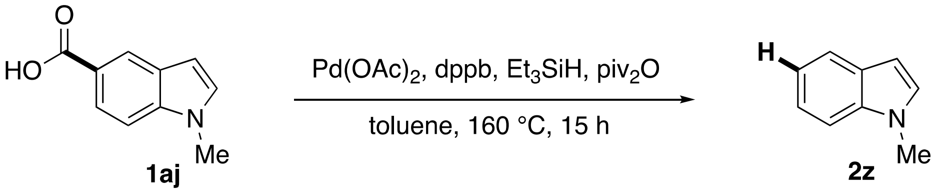


According to the general procedure, the reaction of 1-methyl-1*H*-indole-5-carboxylic acid (0.20 mmol), $Pd(OAc)_2$ (5 mol%), 1,4-bis(diphenylphosphino)butane (10 mol%), triethylsilane (1.5 equiv) and trimethylacetic anhydride (1.5 equiv) in toluene (0.20 M) for 15 h at 160 °C, afforded after work-up and chromatography the title compound in 50% yield (13.2 mg). White solid. **$^1$H NMR (500 MHz, $CDCl_3$)** δ 10.06 (s, 1 H), 8.18 (s, 1 H), 7.84-7.82 (d, *J* = 8.6 Hz, 1 H), 7.44-7.43 (d, *J* = 8.5 Hz, 1 H), 7.18-7.18 (d, *J* = 3.0 Hz, 1 H), 6.68-6.68 (d, *J* = 2.9 Hz, 1 H), 3.88 (s, 3 H). **$^{13}$C NMR (125 MHz, $CDCl_3$)** δ 139.96, 130.73, 129.32, 128.23, 126.47, 121.85, 109.79, 103.27, 33.16. The spectral data matched those reported in the literature.[29]

**4-(Pyridin-3-yl)benzoic acid (1ak, Figure 2A, Entry 37)**

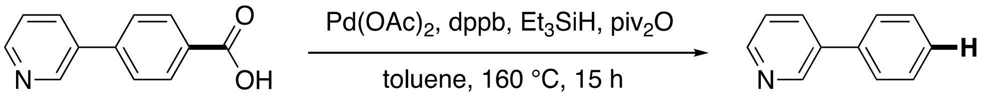


**1ak** **2aa**

According to the general procedure, the reaction of 4-(pyridin-3-yl)benzoic acid (0.20 mmol), $Pd(OAc)_2$ (5 mol%), 1,4-bis(diphenylphosphino)butane (10 mol%), triethylsilane (1.5 equiv) and trimethylacetic anhydride (1.5 equiv) in toluene (0.20 M) for 15 h at 160 °C, afforded after work-up and chromatography the title compound in 95% yield (29.5 mg). White solid. **$^1$H NMR (500 MHz, $CDCl_3$)** δ 8.88 (s, 1 H), 8.63-8.62 (d, *J* = 4.5 Hz, 1 H), 7.91-7.90 (d, *J* = 7.9 Hz, 1 H), 7.62-7.61 (d, *J* = 7.5 Hz, 2 H), 7.53-7.50 (t, *J* = 7.5 Hz, 2 H), 7.45-7.42 (t, *J* = 7.4 Hz, 1 H), 7.41-7.38 (m, 1 H). **$^{13}$C NMR (125 MHz, $CDCl_3$)** δ 148.49, 148.37, 137.87, 136.67, 134.39, 129.10, 128.12, 127.18, 123.56. The spectral data matched those reported in the literature.[30]

**4-(Thiophen-3-yl)benzoic acid (1al, Figure 2A, Entry 38)**

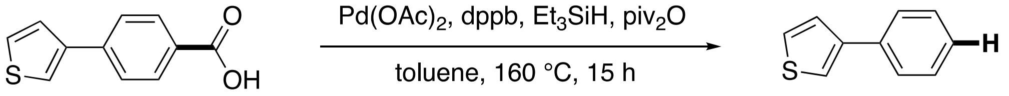


**1al** **2ab**

According to the general procedure, the reaction of 4-(thiophen-3-yl)benzoic acid (0.20 mmol), $Pd(OAc)_2$ (5 mol%), 1,4-bis(diphenylphosphino)butane (10 mol%), triethylsilane (1.5 equiv) and trimethylacetic anhydride (1.5 equiv) in toluene (0.20 M) for 15 h at 160 °C, afforded after work-up and chromatography the title compound in 79% yield (25.4 mg). White solid. **$^1$H NMR (500 MHz, $CDCl_3$)** δ 7.64-7.62 (d, *J* = 7.7 Hz, 1 H), 7.48-7.40 (m, 4 H), 7.38-7.36 (m, 2 H), 7.33-7.30 (t, *J* = 7.4 Hz, 1 H). **$^{13}$C NMR (125 MHz, $CDCl_3$)** δ 142.40, 135.89, 128.81, 127.14, 126.47, 126.37, 126.20, 120.18. The spectral data matched those reported in the literature.[23]

**4-(Dibenzo[*b,d*]furan-4-yl)benzoic acid (1am, Figure 2A, Entry 39)**

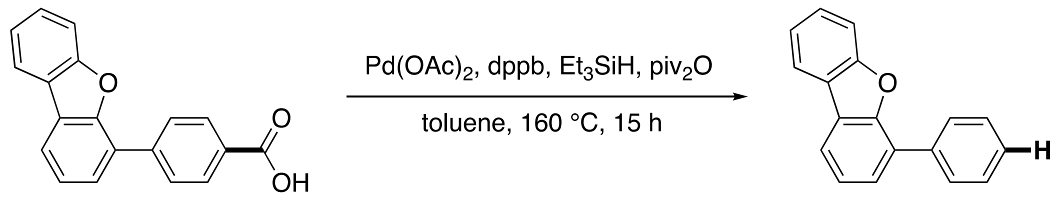


**1am** **2ac**

According to the general procedure, the reaction of 4-(dibenzo[*b,d*]furan-4-yl)benzoic acid (0.20 mmol), $Pd(OAc)_2$ (5 mol%), 1,4-bis(diphenylphosphino)butane (10 mol%), triethylsilane (1.5 equiv) and trimethylacetic anhydride (1.5 equiv) in toluene (0.20 M) for 15 h at 160 °C, afforded after work-up and chromatography the title compound in 91% yield (44.5 mg). White solid. **$^1$H NMR (500 MHz, $CDCl_3$)** δ 8.04-8.03 (d, *J* = 7.7 Hz, 1 H), 7.99-7.96 (t, *J* = 7.4 Hz, 3 H), 7.66-7.64 (d, *J* = 7.9 Hz, 2 H), 7.61-7.58 (t, *J* = 7.6 Hz, 2 H), 7.53-7.46 (m, 3 H), 7.42-7.39 (t, *J* = 7.5 Hz, 1 H). **$^{13}$C NMR (125 MHz, $CDCl_3$)** δ 156.22, 153.41, 136.48, 128.86, 128.70, 127.83, 127.26, 126.91, 125.93, 124.95, 124.26, 123.24, 122.80, 120.71, 119.70, 111.90. The spectral data matched those reported in the literature.[31]

**4-(Dibenzo[*b,d*]thiophen-4-yl)benzoic acid (1an, Figure 2A, Entry 40)**

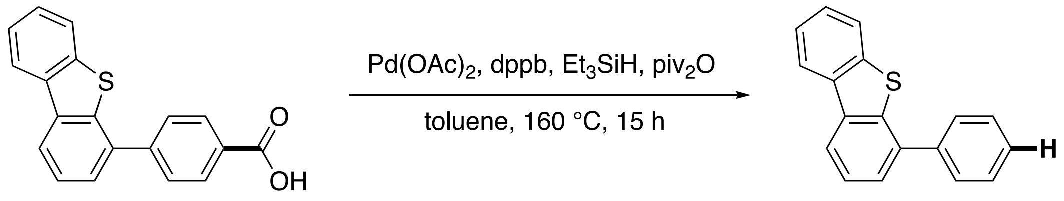


**1an** **2ad**

According to the general procedure, the reaction of 4-(dibenzo[*b,d*]thiophen-4-yl)benzoic acid (0.20 mmol), $Pd(OAc)_2$ (5 mol%), 1,4-bis(diphenylphosphino)butane (10 mol%), triethylsilane (1.5 equiv) and trimethylacetic anhydride (1.5 equiv) in toluene (0.20 M) for 15 h at 160 °C, afforded after work-up and chromatography the title compound in 80% yield (41.7 mg). White solid. **$^1$H NMR (500 MHz, $CDCl_3$)** δ 8.23-8.18 (m, 2 H), 7.87-7.85 (d, *J* = 8.4 Hz, 1 H), 7.78-7.77 (d, *J* = 7.8 Hz, 2 H), 7.61-7.46 (m, 7 H). **$^{13}$C NMR (125 MHz, $CDCl_3$)** δ 140.63, 139.61, 138.63, 137.06, 136.23, 135.82, 128.82, 128.29, 128.03, 126.92, 126.81, 125.12, 124.39, 122.63, 121.75, 120.46. The spectral data matched those reported in the literature.[32]

**2,4,6-Trimethylbenzoic acid (1ao, Figure 2A, Entry 41)**

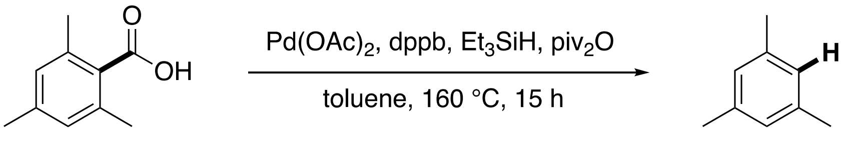


**1ao** **2ae**

According to the general procedure, the reaction of 2,4,6-trimethylbenzoic acid (0.20 mmol), $Pd(OAc)_2$ (5 mol%), 1,4-bis(diphenylphosphino)butane (10 mol%), triethylsilane (1.5 equiv) and trimethylacetic anhydride (1.5 equiv) in toluene (0.20 M) for 15 h at 160 °C, afforded the title compound in 90% yield which was determined by $^1H$ NMR and GC-MS owing to the volatility of the product.[11]

**Probenecid (1ap, Figure 2B, Entry 42)**

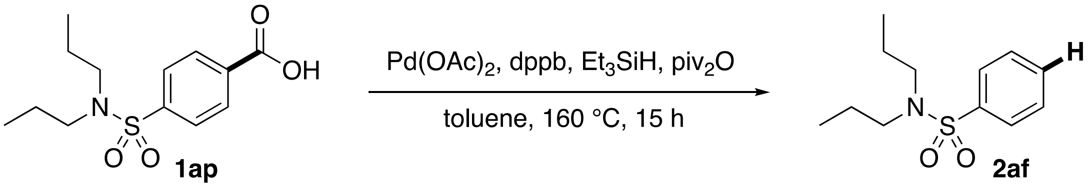


According to the general procedure, the reaction of probenecid (0.20 mmol), $Pd(OAc)_2$ (5 mol%), 1,4-bis(diphenylphosphino)butane (10 mol%), triethylsilane (1.5 equiv) and trimethylacetic anhydride (1.5 equiv) in toluene (0.20 M) for 15 h at 160 °C, afforded after work-up and chromatography the title compound in 97% yield (46.9 mg). White solid. **$^1$H NMR (500 MHz, $CDCl_3$)** δ 7.83-7.82 (d, *J* = 7.8 Hz, 2 H), 7.58-7.56 (t, *J* = 7.4 Hz, 1 H), 7.53-7.50 (t, *J* = 7.6 Hz, 2 H), 3.11-3.08 (t, *J* = 7.6 Hz, 4 H), 1.61-1.53 (m, 4 H), 0.90-0.87 (t, *J* = 7.4 Hz, 6 H). **$^{13}$C NMR (125 MHz, $CDCl_3$)** δ 140.18, 132.22, 128.96, 127.03, 50.02, 22.02, 11.19. The spectral data matched those reported in the literature.[33]

**4-((*N*-(2,4-Difluorophenyl)-2-(3-(trifluoromethyl)phenoxy)nicotinamido)methyl)benzoic acid (1aq, Figure 2B, Entry 43)**

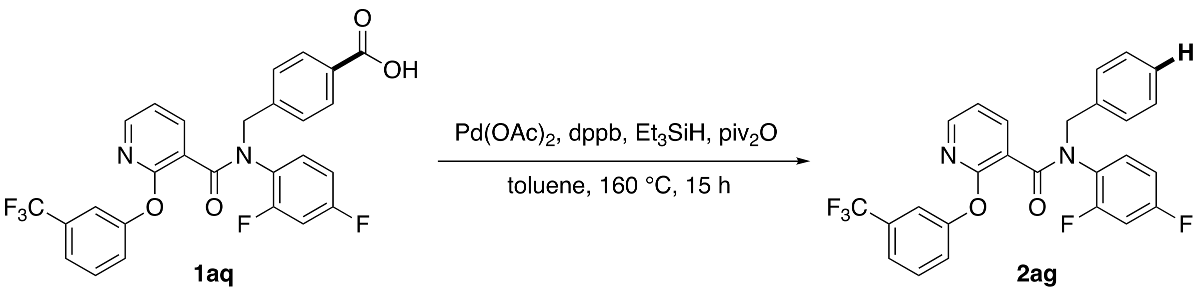


According to the general procedure, the reaction of 4-((*N*-(2,4-difluorophenyl)-2-(3-(trifluoromethyl)phenoxy)nicotinamido)methyl)benzoic acid (0.20 mmol), $Pd(OAc)_2$ (5 mol%), 1,4-bis(diphenylphosphino)butane (10 mol%), triethylsilane (1.5 equiv) and trimethylacetic anhydride (1.5 equiv) in toluene (0.20 M) for 15 h at 160 °C, afforded after work-up and chromatography the title compound in 68% yield (65.9 mg). *New compound.* White solid. **Mp** = 109-110 °C. **$^1$H NMR (500 MHz, $CDCl_3$)** δ 8.04-8.03 (d, $J$ = 4.3 Hz, 1 H), 7.84-7.83 (d, $J$ = 7.2 Hz, 1 H), 7.53-7.47 (m, 2 H), 7.31-7.29 (m, 5 H), 7.17-7.16 (m, 2 H), 7.01-6.98 (t, $J$ = 5.5 Hz, 1 H), 6.91-6.90 (d, $J$ = 6.5 Hz, 1 H), 6.69-6.66 (t, $J$ = 9.2 Hz, 1 H), 6.63-6.60 (t, $J$ = 7.9 Hz, 1 H), 5.48-5.45 (d, $J$ = 14.3 Hz, 1 H), 4.73-4.70 (d, $J$ = 14.4 Hz, 1 H). **$^{13}$C NMR (125 MHz, $CDCl_3$)** δ 167.12, 160.02 (q, $J^F$ = 250.5 Hz), 159.9 (q, $J^F$ = 250.5 Hz), 157.37, 153.19, 148.45, 138.69, 136.23, 131.91 (q, $J^F$ = 32.5 Hz), 131.30 (d, $J^F$ = 9.7 Hz), 130.02, 128.74, 128.58, 127.85, 125.37 (dd, $J^F$ = 4.1 Hz), 124.60, 123.63 (q, $J^F$ = 270.7 Hz), 121.62 (q, $J^F$ = 3.7 Hz), 121.21, 118.78, 118.34 (q, $J^F$ = 3.7 Hz), 111.42 (dd, $J^F$ = 3.7 Hz), 104.82 (dd, $J^F$ = 24.3 Hz), 52.37. **$^{19}$F NMR (471 MHz, $CDCl_3$)** δ -62.68, -107.69, -114.19. **HRMS** calcd for $C_{26}H_{17}O_2F_5N_2Na$ ($M^+$ + Na) 507.1102, found 507.1104.

**4-((((13*S*)-13-Methyl-17-oxo-7,8,9,11,12,13,14,15,16,17-decahydro-6*H*-cyclopenta[*a*]phenanthren-3-yl)oxy)methyl)benzoic acid (1ar, Figure 2B, Entry 44)**

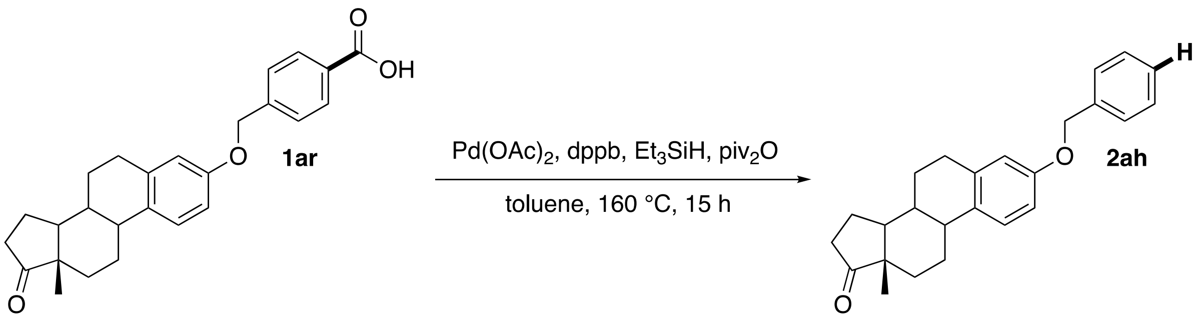


According to the general procedure, the reaction of 4-((((13*S*)-13-methyl-17-oxo-7,8,9,11,12,13,14,15,16,17-decahydro-6*H*-cyclopenta[*a*]phenanthren-3-yl)oxy)methyl)benzoic acid (0.20 mmol), $Pd(OAc)_2$ (5 mol%), 1,4-bis(diphenylphosphino)butane (10 mol%), triethylsilane (1.5 equiv) and trimethylacetic anhydride (1.5 equiv) in toluene (0.20 M) for 15 h at 160 °C, afforded after work-up and chromatography the title compound in 94% yield (67.8 mg). White solid. **$^1$H NMR (500 MHz, CDCl$_3$)** δ 7.75-7.74 (d, *J* = 7.6 Hz, 2 H), 7.63-7.60 (t, *J* = 7.6 Hz, 2 H), 7.49-7.46 (t, *J* = 8.1 Hz, 1 H), 7.24-7.22 (d, *J* = 8.6 Hz, 1 H), 6.81-6.79 (m, 1 H), 6.74 (s, 1 H), 5.12 (s, 2 H), 2.93-2.90 (m, 2 H), 2.55-2.50 (m, 1 H), 2.43-2.40 (m, 1 H), 2.29-2.25 (m, 1 H), 2.20-2.13 (m, 1 H), 2.10-1.97 (m, 3 H), 1.69-1.55 (m, 3 H), 1.53-1.42 (m, 3 H), 0.93 (s, 3 H). **$^{13}$C NMR (125 MHz, CDCl$_3$)** δ 166.88, 156.55, 142.55, 137.94, 132.66, 129.88, 129.61, 126.93, 126.45, 114.95, 112.37, 69.32, 50.43, 48.02, 44.00, 38.34, 35.89, 31.59, 29.67, 26.53, 25.92, 21.61, 13.87. The spectral data matched those reported in the literature.[34]

**4-((((*R*)-2,5,7,8-tetramethyl-2-((4*S*,8*S*)-4,8,12-trimethyltridecyl)chroman-6-yl)oxy)methyl) benzoic acid (1as, Figure 2B, Entry 45)**

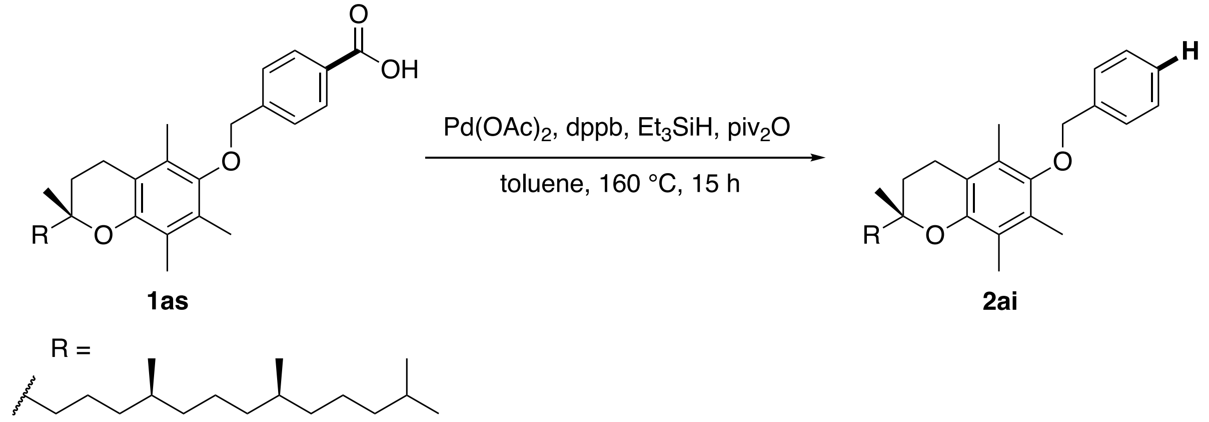


**1as** **2ai**

According to the general procedure, the reaction of 4-((((*R*)-2,5,7,8-tetramethyl-2-((4*S*,8*S*)-4,8,12-trimethyltridecyl)chroman-6-yl)oxy)methyl)benzoic acid (0.20 mmol), Pd(OAc)$_2$ (5 mol%), 1,4-bis(diphenylphosphino)butane (10 mol%), triethylsilane (1.5 equiv) and trimethylacetic anhydride (1.5 equiv) in toluene (0.20 M) for 15 h at 160 °C, afforded after work-up and chromatography the title compound in 62% yield (64.6 mg). White solid. **$^1$H NMR (500 MHz, CDCl$_3$)** δ 7.54-7.53 (d, *J* = 7.5 Hz, 2 H), 7.45-7.42 (t, *J* = 7.4 Hz, 2 H), 7.38-7.35 (t, *J* = 7.2 Hz, 1 H), 4.73 (s, 2 H), 2.64-2.61 (t, *J* = 6.7 Hz, 2 H), 2.25 (s, 3 H), 2.20 (s, 3 H), 2.14 (s, 3 H), 1.89-1.77 (m, 2 H), 1.61-1.53 (m, 3 H), 1.45-1.40 (m, 3 H), 1.35-1.24 (m, 12 H), 1.19-1.09 (m, 6 H), 0.91-0.87 (m, 12 H). **$^{13}$C NMR (125 MHz, CDCl$_3$)** δ 148.14, 147.94, 138.08, 128.47, 127.96, 127.78, 127.72, 125.98, 122.95, 117.62, 74.85, 74.72, 40.10, 39.40, 37.62, 37.45, 37.42, 37.32, 32.81, 32.73, 31.29, 28.01, 24.83, 24.47, 23.92, 22.75, 22.66, 21.08, 20.71, 19.72, 12.89, 12.02, 11.85. The spectral data matched those reported in the literature.[35]

**4-Methyl-1,1'-biphenyl via C-H activation and reduction (1at, Figure 3A, Entry 46)**

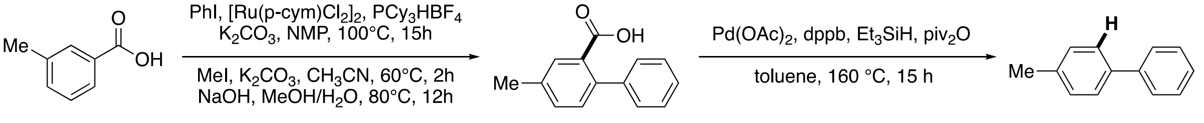


**1at** **2aj**

According to the general procedure,[36] *m*-toluic acid (1.0 mmol) was reacted with iodobenzene (1.5 equiv), [Ru(*p*-cym)Cl$_2$]$_2$ (4 mol%), tricyclohexylphosphine tetrafluoroborate (8 mol%) and $K_2CO_3$ (1.0 equiv) in *N*-methyl-2-pyrrolidone (0.20 M) for 15 h at 100 °C. The reaction mixture was charged with iodomethane (3.0 equiv), $K_2CO_3$ (2.5 equiv) and acetonitrile (1.6 mL) and

heated for 2 h at 60 °C. Methyl 4-methyl-[1,1'-biphenyl]-2-carboxylate was obtained in 93% yield (210.5 mg) after standard work-up and chromatography. A 20 mL vial was charged with methyl 4-methyl-[1,1'-biphenyl]-2-carboxylate (0.93 mmol), NaOH (10.0 equiv), methanol (1 mL) and water (10 mL), and heated for 12 h at 80 °C. The organic solvent was removed under vacuum, the reaction mixture was acidified with HCl (6.0 *N*, 5 mL), filtered and washed with water (3 × 10 mL) to afford **1as** in 90% yield (177.7 mg). According to the general procedure for decarbonylative reduction, the reaction of 4-methyl-[1,1'-biphenyl]-2-carboxylic acid (0.20 mmol), $Pd(OAc)_2$ (5 mol%), 1,4-bis(diphenylphosphino)butane (10 mol%), triethylsilane (1.5 equiv) and trimethylacetic anhydride (1.5 equiv) in toluene (0.20 M) for 15 h at 160 °C, afforded after work-up and chromatography compound **2ai** in 92% yield (31.0 mg). White solid. **$^{1}$H NMR (500 MHz, $CDCl_3$)** δ 7.62-7.60 (d, *J* = 7.7 Hz, 2 H), 7.53-7.52 (d, *J* = 7.9 Hz, 2 H), 7.47-7.44 (t, *J* = 7.6 Hz, 2 H), 7.37-7.34 (t, *J* = 7.3 Hz, 1 H), 7.28-7.28 (d, *J* = 4.8 Hz, 2 H), 2.43 (s, 3 H). **$^{13}$C NMR (125 MHz, $CDCl_3$)** δ 141.18, 138.38, 137.04, 129.49, 128.72, 127.01, 126.99, 21.12. The spectral data matched those reported in the literature.[37]

**3-Methoxy-1,1'-biphenyl via iodination, arylation and reduction (1au, Figure 3B, Entry 47)**

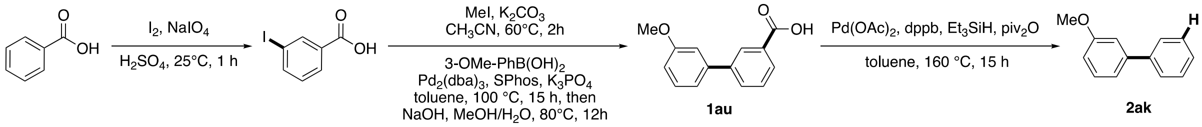


According to the general procedure,[38] iodine (3.0 mmol) and $NaIO_4$ (1.0 mmol) were dropwise added to $H_2SO_4$. Stirring was continued for 30 min at 25 °C to give iodinating solution containing ca. 7.0 mmol (1.17 equiv) of the $I^+$ intermediate. Benzoic acid (6.0 mmol) was added in one portion to the iodinating solution containing the $I^+$ intermediate (1.17 equiv) and the resulting solution was stirred for 1 h at 25 °C. Then the reaction mixture was slowly poured into stirred ice water (50 g). The crude solid product was filtered and washed with water (3 × 10 mL) to afford 3-iodobenzoic acid in 72% yield (1.072 g). White solid. 3-Iodobenzoic acid (4.32 mmol) was reacted with iodomethane (3.0 equiv), $K_2CO_3$ (2.5 equiv) and acetonitrile (10.0 mL) for 2 h at 60 °C to afford after work-up and chromatography methyl 3-iodobenzoate in 91% yield (1.031 g). According to the general procedure,[39] the reaction of 3-iodobenzoate (1.0 mmol), (3-methoxyphenyl)boronic acid (1.5 equiv), $Pd_2(dba)_3$ (1 mol%), SPhos (4 mol%) and $K_3PO_4$ (2.0 equiv) in toluene (0.25 M) for 15 h at 100 °C, afforded after work-up and chromatography

methyl 3'-methoxy-[1,1'-biphenyl]-3-carboxylate in 98% yield (237.4 mg). White solid. A 20 mL vial was charged with methyl 3'-methoxy-[1,1'-biphenyl]-3-carboxylate (0.98 mmol), NaOH (10.0 equiv), methanol (1 mL) and water (10 mL), and heated for 12 h at 80 °C. The organic solvent was removed under vacuum, the reaction mixture was acidified with HCl (6.0 *N*, 5 mL), filtered and washed with water (3 × 10 mL) to afford **1at** in 89% yield (199.1 mg). White solid. According to the general procedure for decarbonylative reduction, the reaction of 3'-methoxy-[1,1'-biphenyl]-3-carboxylic acid (0.20 mmol), $Pd(OAc)_2$ (5 mol%), 1,4-bis(diphenylphosphino)butane (10 mol%), triethylsilane (2.0 equiv) and pivalic anhydride (2.0 equiv) in toluene (0.20 M) for 15 h at 160 °C, afforded after work-up and chromatography compound **2aj** in 95% yield (35.0 mg). White solid. **$^1$H NMR (500 MHz, $CDCl_3$)** δ 7.63-7.62 (d, *J* = 7.7 Hz, 2 H), 7.49-7.46 (t, *J* = 7.5 Hz, 2 H), 7.41-7.38 (m, 2 H), 7.23-7.21 (d, *J* = 7.6 Hz, 1 H), 7.17 (s, 1 H), 6.95-6.93 (d, *J* = 8.1 Hz, 1 H), 3.90 (s, 3 H). **$^{13}$C NMR (125 MHz, $CDCl_3$)** δ 159.97, 142.81, 141.14, 129.76, 128.75, 127.43, 127.22, 119.71, 112.94, 112.71, 55.32. The spectral data matched those reported in the literature.[40]

**(*E*)-Stilbene via iodination, olefination and reduction (1av, Figure 3C, Entry 48)**

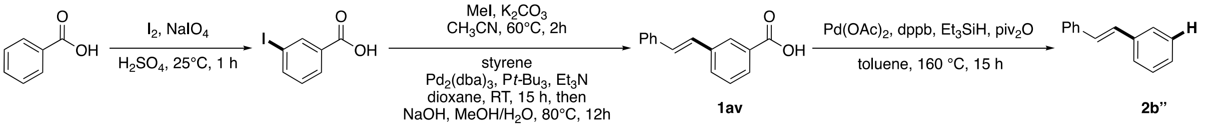


According to the general procedure,[38] iodine (3.0 mmol) and $NaIO_4$ (1.0 mmol) were dropwise added to $H_2SO_4$. Stirring was continued for 30 min at 25 °C to give iodinating solution containing ca. 7.0 mmol (1.17 equiv) of the $I^+$ intermediate. Benzoic acid (6.0 mmol) was added in one portion to the iodinating solution containing the $I^+$ intermediate (1.17 equiv) and the resulting solution was stirred for 1 h at 25 °C. Then the reaction mixture was slowly poured into stirred ice water (50 g). The crude solid product was filtered and washed with water (3 × 10 mL) to afford 3-iodobenzoic acid in 72% yield (1.072 g). White solid. 3-Iodobenzoic acid (4.32 mmol) was reacted with iodomethane (3.0 equiv), $K_2CO_3$ (2.5 equiv) and acetonitrile (10.0 mL) for 2 h at 60 °C to afford after work-up and chromatography methyl 3-iodobenzoate in 91% yield (1.031 g). According to the general procedure,[41] the reaction of 3-iodobenzoate (1.0 mmol), $Pd_2(dba)_3$ (1.5 mol%), $P(tBu)_3HBF_4$ (3 mol%), $Et_3N$ (1.1 equiv) and styrene (2.0 equiv) in 1,4-dioxane (0.2 M) for 15 h at room temperature, afforded methyl (*E*)-3-styrylbenzoate in 96%

yield (228.8 mg). White solid. A 20 mL vial was charged with methyl (*E*)-3-styrylbenzoate (0.96 mmol), NaOH (10.0 equiv), methanol (1 mL) and water (10 mL), and heated for 12 h at 80 °C. The organic solvent was removed under vacuum, the reaction mixture was acidified with HCl (6.0 *N*, 5 mL), filtered and washed with water (3 × 10 mL) to afford **1au** in 86% yield (185.2 mg). White solid. According to the general procedure for decarbonylative reduction, the reaction of (*E*)-3-styrylbenzoic acid (0.20 mmol), $Pd(OAc)_2$ (5 mol%), 1,4-bis(diphenylphosphino)butane (10 mol%), triethylsilane (2.0 equiv) and pivalic anhydride (2.0 equiv) in toluene (0.20 M) for 15 h at 160 °C, afforded after work-up and chromatography compound **2a** in 90% yield (32.5 mg). White solid. **$^1$H NMR (500 MHz, $CDCl_3$)** δ 7.56-7.54 (d, *J* = 7.5 Hz, 4 H), 7.41-7.38 (t, *J* = 7.5 Hz, 4 H), 7.31-7.28 (t, *J* = 7.4 Hz, 2 H), 7.14 (s, 2 H). **$^{13}$C NMR (125 MHz, $CDCl_3$)** δ 137.35, 128.71, 128.70, 127.64, 126.53. The spectral data matched those reported in the literature.[11]

**1,1’-Biphenyl via oxidation and reduction (1a, Figure 3D, Entry 49)**

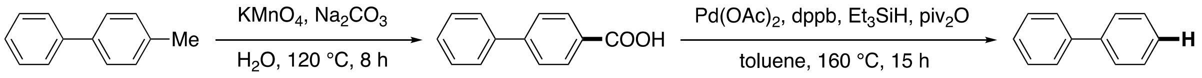


**1a** **2a**

According to the general procedure,[42] methyl 4-methyl-1,1’-biphenyl (2.0 mmol) was reacted with $KMnO_4$ (2.5 equiv) and $Na_2CO_3$ (1.0 equiv) in $H_2O$ (0.2 M) for 8 h at 120 °C. The reaction mixture was filtered through a pad of celite, the filtrate was acidified with HCl (6.0 *N*, 5 mL), filtered washed with water (3 × 10 mL) to afford [1,1’-biphenyl]-4-carboxylic acid in 87% yield (344.9 mg). White solid. According to the general procedure for decarbonylative reduction, the reaction of [1,1’-biphenyl]-4-carboxylic acid (0.20 mmol), $Pd(OAc)_2$ (5 mol%), 1,4-bis(diphenylphosphino)butane (10 mol%), triethylsilane (2.0 equiv) and pivalic anhydride (2.0 equiv) in toluene (0.20 M) for 15 h at 160 °C, afforded after work-up and chromatography compound **2a** in 97% yield (30.0 mg). White solid. **$^1$H NMR (500 MHz, $CDCl_3$)** δ 7.64-7.62 (d, *J* = 7.3 Hz, 4 H), 7.49-7.46 (t, *J* = 7.5 Hz, 4 H), 7.40-7.37 (t, *J* = 7.4 Hz, 2 H). **$^{13}$C NMR (125 MHz, $CDCl_3$)** δ 141.26, 128.77, 127.27, 127.19. The spectral data matched those reported in the literature.[11]

## References


1. Frisch, M. J.; Trucks, G. W.; Schlegel, H. B.; Scuseria, G. E.; Robb, M. A.; Cheeseman, J. R.; Scalmani, G.; Barone, V.; Mennucci, B.; Petersson, G. A.; Nakatsuji, H.; Caricato, M.; Li, X.; Hratchian, H. P.; Izmaylov, A. F.; Bloino, J.; Zheng, G.; Sonnenberg, J. L.; Hada, M.; Ehara, M.; Toyota, K.; Fukuda, R.; Hasegawa, J.; Ishida, M.; Nakajima, T.; Honda, Y.; Kitao, O.; Nakai, H.; Vreven, T.; Montgomery, J. A., Jr.; Peralta, J. E.; Ogliaro, F.; Bearpark, M.; Heyd, J. J.; Brothers, E.; Kudin, K. N.; Staroverov, V. N.; Kobayashi, R.; Normand, J.; Raghavachari, K.; Rendell, A.; Burant, J. C.; Iyengar, S. S.; Tomasi, J.; Cossi, M.; Rega, N.; Millam, J. M.; Klene, M.; Knox, J. E.; Cross, J. B.; Bakken, V.; Adamo, C.; Jaramillo, J.; Gomperts, R.; Stratmann, R. E.; Yazyev, O.; Austin, A. J.; Cammi, R.; Pomelli, C.; Ochterski, J. W.; Martin, R. L.; Morokuma, K.; Zakrzewski, V. G.; Voth, G. A.; Salvador, P.; Dannenberg, J. J.; Dapprich, S.; Daniels, A. D.; Farkas, O.; Foresman, J. B.; Ortiz, J. V.; Cioslowski, J.; Fox, D. J., *Gaussian 09*, Revision D, 01 Gaussian, Inc., Wallingford CT, 2010.
2. (a) Becke, A. D. *J. Chem. Phys.* **1993**, *98*, 5648. (b) Lee, C.; Yang, W.; Parr, R. G. *Phys. Rev. B*: *Condens. Matter Mater. Phys.* **1988**, *37*, 785.
3. (a) Hay, P. J.; Wadt, W. R. *J. Chem. Phys.* **1985**, *82*, 270. (b) Wadt, W. R.; Hay, P. J. *J. Chem. Phys.* **1985**, *82*, 284. (c) Hay, P. J.; Wadt, W. R. *J. Chem. Phys.* **1985**, *82*, 299.
4. (a) Zhao, Y.; Truhlar, D. G. *Theor. Chem. Acc.* **2008**, *120*, 215. (b) Zhao, Y.; Truhlar, D. G. *Acc. Chem. Res.* **2008**, *41*, 157.
5. (a) von Szentpaly, L.; Fuentealba, P.; Preuss, H.; Stoll, H. *Chem. Phys. Lett.* **1982**, *93*, 555. (b) Dolg, M.; Wedig, U.; Stoll, H.; Preuss, H. *J. Chem. Phys.* **1987**, *86*, 866. (c) Schwerdtfeger, P.; Dolg, M.; Schwarz, W. H. E.; Bowmaker, G. A.; Boyd, P. D. W. *J. Chem. Phys.* **1989**, *91*, 1762.
6. Marenich, A. V.; Cramer, C. J.; Truhlar, D. G. *J. Phys. Chem. B* **2009**, *113*, 6378.
7. CYLview, 1.0b, C. Y. Legault, Université de Sherbrooke, 2009 (http://www.cylview.org).
8. (a) Wertz, D. H. *J. Am. Chem. Soc.* **1980**, *102*, 5316. (b) Leung, B. O.; Reid, D. L.; Armstrong, D. A.; Rauk, A. *J. Phys. Chem. A* **2004**, *108*, 2720.
9. Martin, R. L.; Hay, P. J.; Pratt, L. R. *J. Phys. Chem. A* **1998**, *102*, 3565.
10. (a) Yu, Z. X.; Houk, K. N. *J. Am. Chem. Soc.* **2003**, *125*, 13825. (b) Chen, Y.; Ye, S.; Jiao, L.; Liang, Y.; Sinha-Mahapatra, D. K.; Herndon, J. W.; Yu, Z.-X. *J. Am. Chem. Soc.*

**2007**, *129*, 10773. (c) Liang, Y.; Liu, S.; Xia, Y.; Li, Y.; Yu, Z.-X. *Chem. - Eur. J.* **2008**, *14*, 4361. (d) Li, H.; Jiang, J.; Lu, G.; Huang, F.; Wang, Z.-X. *Organometallics* **2011**, *30*, 3131. (e) Li, H.; Wen, M.; Wang, Z.-X. *Inorg. Chem*. **2012**, *51*, 5716. (f) Wen, M.; Huang, F.; Lu, G.; Wang, Z.-X. *Inorg. Chem.* **2013**, *52*, 12098. (g) Qu, S.; Dang, Y.; Song, C.; Wen, M.; Huang, K. − W.; Wang, Z.-X. *J. Am. Chem. Soc.* **2014**, *136*, 4974. (h) Yu, J.-L.; Zhang, S.-Q.; Hong, X. *J. Am. Chem. Soc.* **2017**, *139*, 7224.

11. Yue, H.; Guo, L.; Lee, S.-C.; Liu, X.; Rueping, M. *Angew. Chem. Int. Ed.* **2017**, *56*, 3972.
12. Afsaneh, P.; Abolfazl, H.; Marina, S.; Heike, H.; Jonathan, B.; Kevin, T. *J. Org. Chem.* **2018**, *83*, 10070.
13. Shi, S.; Meng, G.; Szostak, M. *Angew. Chem. Int. Ed.* **2016**, *55*, 6959.
14. Li, Y.; Dong, Y.; Wei, Y.-L.; Jv, J.-J.; Chen, Y.-Q.; Ma, J.-P.; Yao, J.-J.; Dong, Y.-B. *Organometallics*. **2018**, *37*, 1645.
15. Giri, R.; Brusoe, A.; Troshin, K.; Wang, J. Y.; Font, M.; Hartwig, J. F. *J. Am. Chem. Soc.* **2018**, *140*, 793.
16. Yilmaz, O.; Oderinde, M. S.; Emmert, M. H. *J. Org. Chem.* **2018**, *83*, 11089.
17. Low, C. H.; Rosenberg, J. N.; Lopez, M. A.; Agapie, T. *J. Am. Chem. Soc.* **2018**, *140*, 11906.
18. Suga, T.; Shimazu, S.; Ukaji, Y. *Org. Lett.* **2018**, *20*, 5389.
19. Ho, Y. A.; Leiendecker, M.; Liu, X.; Wang, C.; Alandini, N.; Rueping, M. *Org. Lett.* **2018**, *20*, 5644.
20. Correa, A.; Martin, R. *J. Am. Chem. Soc.* **2014**, *136*, 7253.
21. Downey, C. W.; Glist, H. M.; Takashima, A.; Bottum, S. R.; Dixon, G. J. *Tetrahedron Lett.* **2018**, *59*, 3080.
22. Lei, P.; Meng, G.; Shi, S.; Ling, Y.; An, J.; Szostak, R.; Szostak, M. *Chem. Sci.* **2017**, *8*, 6525.
23. Tan, G.; You, Q.; You J. *ACS Catal.* **2018**, *8*, 8709.
24. Liu, C.; Li, G.; Shi, S.; Meng, G.; Lalancette, R.; Szostak, R.; Szostak, M. *ACS Catal.* **2018**, *8*, 9131.
25. Yu, C.; Patureau, F. W. *Angew. Chem. Int. Ed.* **2018**, *57*, 11807.
26. Yoshida, Y.; Otsuka, S.; Nogi, K.; Yorimitsu, H. *Org. Lett.* **2018**, *20*, 1134.

27. Qiao, Y.; Yang, Q.; Schelter, E. J. *Angew. Chem. Int. Ed.* **2018**, *57*, 10999.
28. Miyamura, H.; Suzuki, A.; Yasukawa, T.; Kobayashi, S. *J. Am. Chem. Soc.* **2018**, *140*, 11325.
29. Han, Y.; Zhang, S.; He, J.; Zhang, Y. *ACS Catal.* **2018**, *8*, 8765.
30. Gribble, M. W.; Guo, S.; Buchwald, S. L. *J. Am. Chem. Soc.* **2018**, *140*, 5057.
31. Maetani, S.; Fukuyama, T.; Ryu, I. *Org. Lett.* **2013**, *15*, 2754.
32. Wang, M.; Fan, Q.; Jiang, X. *Org. Lett.* **2016**, *18*, 5756.
33. Liu, T.; Zheng, D.; Li, Z.; Wu, J. *Adv. Synth. Catal.* **2017**, *359*, 2653.
34. Niu, D.; Buchwald, S. L. *J. Am. Chem. Soc.* **2015**, *137*, 9716.
35. Cohen, N.; Schaer, B.; Scalone, M. *J. Org. Chem.* **1992**, *57*, 5783.
36. Huang, L.; Weix, D. J. *Org. Lett.* **2016**, *18*, 5432.
37. Ahmed, J.; Chakraborty, S.; Jose, A.; P. S.; Mandal, S. K. *J. Am. Chem. Soc.* **2018**, *140*, 8330.
38. Kraszkiewicz, L.; Sosnowski, M.; Skulski, L. *Synthesis* **2006**, *2006*, 1195.
39. Barder, T. E.; Walker, S. D.; Martinelli, J. R.; Buchwald, S. L. *J. Am. Chem. Soc.* **2005**, *127*, 4685.
40. Igarashi, T.; Haito, A.; Chatani, N.; Tobisu, M. *ACS Catal.* **2018**, *8*, 7475.
41. Littke, A. F.; Fu, G. C. *J. Am. Chem. Soc.* **2001**, *123*, 6989.
42. Ni, J.; Li, J.; Fan, Z.; Zhang, A. *Org. Lett.* **2016**, *18*, 5960.

**Cartesian coordinates of the structures**

**3**

| | | | |
|---|---|---|---|
| Pd | -0.890884 | 0.150910 | 0.501604 |
| P | -2.470741 | -1.258008 | -0.510997 |
| P | -0.321048 | 2.107193 | -0.669333 |
| C | -3.036873 | -0.443565 | -2.106013 |
| C | -2.026361 | -2.948707 | -1.122758 |
| C | -4.061414 | -1.591627 | 0.379489 |
| C | -0.847743 | 1.910488 | -2.466125 |
| C | 1.459827 | 2.579171 | -0.872914 |
| C | -1.098069 | 3.732893 | -0.232411 |
| C | -3.310102 | 1.076310 | -1.967442 |
| H | -2.221933 | -0.630208 | -2.814983 |
| H | -3.911167 | -0.971591 | -2.505831 |
| C | -2.978251 | -3.839909 | -1.646853 |
| C | -0.681924 | -3.345127 | -1.076870 |
| C | -4.026767 | -2.450201 | 1.494078 |
| C | -5.281563 | -0.980902 | 0.052711 |
| C | -2.360540 | 2.002727 | -2.752429 |
| H | -0.465588 | 0.931516 | -2.778917 |
| H | -0.336576 | 2.662892 | -3.080667 |
| C | 2.085975 | 3.374537 | 0.103616 |
| C | 2.248434 | 2.074104 | -1.920283 |
| C | -0.816011 | 4.922181 | -0.926000 |
| C | -2.030867 | 3.769469 | 0.813893 |
| H | -3.289547 | 1.365764 | -0.910330 |
| H | -4.327895 | 1.290986 | -2.319097 |
| C | -2.590287 | -5.093741 | -2.118227 |
| H | -4.027744 | -3.558108 | -1.674014 |

| | | | |
|---|---|---|---|
| C | -0.293183 | -4.602182 | -1.547903 |
| H | 0.065115 | -2.676843 | -0.658594 |
| C | -5.173117 | -2.700564 | 2.244636 |
| H | -3.093110 | -2.936753 | 1.767223 |
| C | -6.430287 | -1.222138 | 0.813257 |
| H | -5.349531 | -0.310230 | -0.797823 |
| H | -2.684292 | 3.037077 | -2.581005 |
| H | -2.495435 | 1.816024 | -3.827761 |
| C | 3.446687 | 3.668934 | 0.025604 |
| H | 1.500375 | 3.775883 | 0.926095 |
| C | 3.612741 | 2.365929 | -1.996355 |
| H | 1.803303 | 1.449865 | -2.689252 |
| C | -1.454068 | 6.114001 | -0.582313 |
| H | -0.085311 | 4.919445 | -1.730697 |
| C | -2.672534 | 4.962367 | 1.158342 |
| H | -2.247568 | 2.852189 | 1.356626 |
| C | -1.246154 | -5.477097 | -2.069898 |
| H | -3.336853 | -5.774978 | -2.519157 |
| H | 0.752224 | -4.892365 | -1.490468 |
| C | -6.382045 | -2.082878 | 1.908637 |
| H | -5.123948 | -3.377067 | 3.094171 |
| H | -7.364626 | -0.737108 | 0.541513 |
| C | 4.218004 | 3.166135 | -1.025938 |
| H | 3.905169 | 4.297096 | 0.785465 |
| H | 4.199644 | 1.972762 | -2.822754 |
| C | -2.385422 | 6.135779 | 0.460282 |
| H | -1.224506 | 7.026773 | -1.126338 |
| H | -3.392968 | 4.973376 | 1.972220 |
| H | -0.947442 | -6.457684 | -2.431782 |
| H | -7.275799 | -2.272108 | 2.497172 |

| | | | |
|---|---|---|---|
| H | 5.277458 | 3.399511 | -1.089819 |
| H | -2.881390 | 7.065327 | 0.727474 |
| C | -0.192050 | -0.358497 | 2.963451 |
| C | 0.360864 | 0.822348 | 3.482303 |
| C | 0.597026 | -1.208922 | 2.175983 |
| H | -1.208682 | -0.643776 | 3.217180 |
| C | 1.701072 | 1.141194 | 3.242999 |
| H | -0.252492 | 1.476918 | 4.095670 |
| C | 1.938426 | -0.864411 | 1.905666 |
| H | 0.220442 | -2.163841 | 1.829620 |
| C | 2.486999 | 0.306184 | 2.453138 |
| H | 2.127928 | 2.047332 | 3.663179 |
| C | 2.721843 | -1.785410 | 1.053431 |
| H | 3.520812 | 0.558371 | 2.249141 |
| O | 4.008150 | -1.306596 | 0.805761 |
| O | 2.313750 | -2.808522 | 0.558164 |
| C | 5.062114 | -2.192333 | 0.585257 |
| C | 6.127533 | -1.563544 | -0.313126 |
| O | 5.101241 | -3.283354 | 1.085462 |
| C | 6.576032 | -0.206774 | 0.274809 |
| C | 7.321180 | -2.527593 | -0.401523 |
| C | 5.508228 | -1.344927 | -1.713435 |
| H | 6.979136 | -0.324729 | 1.287427 |
| H | 7.368671 | 0.216295 | -0.353333 |
| H | 5.749555 | 0.508427 | 0.309620 |
| H | 7.018515 | -3.497322 | -0.807334 |
| H | 8.092733 | -2.102764 | -1.053462 |
| H | 7.761536 | -2.704871 | 0.584833 |
| H | 5.160127 | -2.289939 | -2.145666 |
| H | 6.265983 | -0.925103 | -2.385400 |

| | | | |
|---|---|---|---|
| H | 4.663946 | -0.650531 | -1.670655 |

**TS4**

| | | | |
|---|---|---|---|
| Pd | 0.143364 | -0.357904 | -0.093653 |
| P | -1.868444 | 0.605320 | -0.786814 |
| C | -0.347341 | -2.455673 | 0.270716 |
| C | -1.516578 | 2.059903 | -1.909877 |
| C | -2.902292 | 1.361668 | 0.548375 |
| C | -3.114190 | -0.324972 | -1.787978 |
| C | -1.032038 | -2.542077 | 1.601677 |
| O | -0.626468 | -3.136335 | -0.699777 |
| C | -0.798721 | 1.733802 | -3.234520 |
| H | -0.928195 | 2.776108 | -1.325003 |
| H | -2.469394 | 2.548327 | -2.150660 |
| C | -3.413972 | 0.506377 | 1.540748 |
| C | -3.169965 | 2.735918 | 0.647662 |
| C | -4.428577 | 0.142343 | -1.959439 |
| C | -2.717657 | -1.503737 | -2.437367 |
| C | -2.285892 | -3.173267 | 1.624433 |
| C | -0.505902 | -2.012561 | 2.790796 |
| C | 0.607520 | 1.109030 | -3.177698 |
| H | -1.439721 | 1.071794 | -3.830297 |
| H | -0.734829 | 2.676850 | -3.796876 |
| C | -4.181248 | 1.008909 | 2.589708 |
| H | -3.208499 | -0.558723 | 1.494910 |
| C | -3.934153 | 3.239363 | 1.704980 |
| H | -2.785743 | 3.428574 | -0.093036 |
| C | -5.325606 | -0.553036 | -2.769315 |
| H | -4.754759 | 1.046068 | -1.451680 |
| C | -3.619658 | -2.195517 | -3.251367 |

| | | | |
|---|---|---|---|
| H | -1.719176 | -1.897706 | -2.279790 |
| C | -3.007160 | -3.263128 | 2.815040 |
| H | -2.676020 | -3.589938 | 0.701763 |
| C | -1.231554 | -2.106613 | 3.977601 |
| H | 0.471680 | -1.544950 | 2.781906 |
| C | 1.681647 | 1.896198 | -2.397801 |
| H | 0.558982 | 0.086461 | -2.785049 |
| H | 0.950822 | 1.018095 | -4.217353 |
| C | -4.443819 | 2.379062 | 2.676872 |
| H | -4.566195 | 0.327813 | 3.343876 |
| H | -4.130910 | 4.306999 | 1.762531 |
| C | -4.921188 | -1.722806 | -3.419498 |
| H | -6.340733 | -0.183710 | -2.891852 |
| H | -3.301971 | -3.110628 | -3.743967 |
| C | -2.484017 | -2.727860 | 3.994937 |
| H | -3.974505 | -3.758930 | 2.822491 |
| H | -0.814321 | -1.698736 | 4.894620 |
| P | 1.713277 | 1.551953 | -0.539364 |
| H | 2.670176 | 1.613529 | -2.773539 |
| H | 1.578930 | 2.974636 | -2.569816 |
| H | -5.038338 | 2.771571 | 3.497716 |
| H | -5.622232 | -2.265040 | -4.048968 |
| H | -3.043322 | -2.802056 | 4.923943 |
| C | 3.526549 | 1.366526 | -0.205938 |
| C | 1.332478 | 3.186001 | 0.238645 |
| C | 4.188126 | 2.086090 | 0.801588 |
| C | 4.252898 | 0.401455 | -0.927539 |
| C | 0.464297 | 3.212875 | 1.340386 |
| C | 1.882968 | 4.398090 | -0.214589 |
| C | 5.542560 | 1.865601 | 1.063517 |

| | | | |
|---|---|---|---|
| H | 3.651359 | 2.829520 | 1.381923 |
| C | 5.607678 | 0.190886 | -0.669471 |
| H | 3.756079 | -0.208882 | -1.675726 |
| C | 0.146488 | 4.419354 | 1.970920 |
| H | 0.028533 | 2.282805 | 1.696287 |
| C | 1.563676 | 5.602661 | 0.411431 |
| H | 2.575485 | 4.401658 | -1.052166 |
| C | 6.259632 | 0.923519 | 0.325850 |
| H | 6.036515 | 2.438673 | 1.844165 |
| H | 6.152833 | -0.552075 | -1.246222 |
| C | 0.693019 | 5.615382 | 1.505525 |
| H | -0.534064 | 4.419844 | 2.817836 |
| H | 1.996499 | 6.531387 | 0.048575 |
| H | 7.314401 | 0.756599 | 0.527234 |
| H | 0.444742 | 6.555165 | 1.991805 |
| C | 2.133374 | -2.648485 | -0.347676 |
| C | 3.068401 | -3.786196 | 0.087763 |
| O | 1.253069 | -2.277284 | 0.617979 |
| O | 2.181010 | -2.100823 | -1.431939 |
| C | 3.994425 | -4.158904 | -1.080252 |
| C | 2.215055 | -5.004415 | 0.504054 |
| C | 3.904307 | -3.297874 | 1.293632 |
| H | 4.597748 | -3.302578 | -1.396962 |
| H | 4.672243 | -4.965037 | -0.775391 |
| H | 3.420290 | -4.499129 | -1.947620 |
| H | 1.576801 | -5.343077 | -0.319277 |
| H | 2.872832 | -5.833595 | 0.790871 |
| H | 1.572679 | -4.764568 | 1.356155 |
| H | 4.511325 | -2.424430 | 1.028497 |
| H | 4.581668 | -4.096155 | 1.620327 |

| | | | |
|---|---|---|---|
| H | 3.260737 | -3.025882 | 2.135252 |

**5**

| | | | |
|---|---|---|---|
| Pd | 0.107743 | 0.054059 | -0.215075 |
| P | 1.811581 | -1.308793 | 0.628443 |
| P | -1.935752 | -1.247913 | 0.524638 |
| C | 1.363303 | -2.473188 | 2.036751 |
| C | 3.260392 | -0.398813 | 1.315417 |
| C | 2.550363 | -2.479676 | -0.587020 |
| C | 1.509935 | 1.008844 | -1.358587 |
| O | -1.200230 | 1.449283 | -1.191703 |
| C | -1.617077 | -2.933098 | 1.278353 |
| C | -3.234320 | -0.588881 | 1.657623 |
| C | -2.842304 | -1.688714 | -1.018863 |
| C | 0.182798 | -2.041634 | 2.936865 |
| H | 1.150784 | -3.437882 | 1.563899 |
| H | 2.274038 | -2.619576 | 2.626369 |
| C | 4.328750 | -0.025780 | 0.485339 |
| C | 3.275861 | 0.002517 | 2.660635 |
| C | 3.633454 | -3.303782 | -0.233477 |
| C | 1.985038 | -2.603485 | -1.864524 |
| C | 2.108179 | 2.317771 | -0.945664 |
| O | 1.763041 | 0.498206 | -2.429014 |
| C | -1.566893 | 2.473182 | -0.497634 |
| C | -1.089877 | -2.881028 | 2.723158 |
| H | -0.896826 | -3.440106 | 0.625739 |
| H | -2.537215 | -3.527328 | 1.244037 |
| C | -4.375965 | -1.348591 | 1.975000 |
| C | -3.060437 | 0.666603 | 2.257903 |
| C | -4.020694 | -1.026463 | -1.393477 |

| | | | |
|---|---|---|---|
| C | -2.296374 | -2.640695 | -1.896201 |
| H | -0.050190 | -0.980947 | 2.783242 |
| H | 0.477895 | -2.137018 | 3.989078 |
| C | 5.394492 | 0.716604 | 0.994677 |
| H | 4.330509 | -0.315610 | -0.560941 |
| C | 4.341849 | 0.747457 | 3.166523 |
| H | 2.453438 | -0.256698 | 3.321812 |
| C | 4.132156 | -4.240322 | -1.137496 |
| H | 4.095373 | -3.207537 | 0.745489 |
| C | 2.489264 | -3.543068 | -2.768221 |
| H | 1.177398 | -1.942748 | -2.161093 |
| C | 1.789308 | 2.933738 | 0.270233 |
| C | 3.035254 | 2.923355 | -1.813323 |
| C | -2.447026 | 3.515495 | -1.242881 |
| O | -1.267099 | 2.681913 | 0.692193 |
| H | -1.878963 | -2.495548 | 3.379278 |
| H | -0.893860 | -3.912705 | 3.047752 |
| C | -5.318926 | -0.865358 | 2.880128 |
| H | -4.537835 | -2.315224 | 1.505156 |
| C | -4.011561 | 1.146714 | 3.164917 |
| H | -2.208721 | 1.285976 | 1.988223 |
| C | -4.648133 | -1.324988 | -2.604424 |
| H | -4.452226 | -0.276701 | -0.738466 |
| C | -2.927565 | -2.940884 | -3.103245 |
| H | -1.367723 | -3.147449 | -1.646973 |
| C | 5.405347 | 1.104107 | 2.335595 |
| H | 6.214945 | 0.995080 | 0.339110 |
| H | 4.338949 | 1.049046 | 4.210574 |
| C | 3.557965 | -4.362949 | -2.406398 |
| H | 4.969986 | -4.871799 | -0.853914 |

| | | | |
|---|---|---|---|
| H | 2.049490 | -3.623408 | -3.758501 |
| C | 2.393977 | 4.143540 | 0.615452 |
| H | 1.034024 | 2.491502 | 0.911020 |
| C | 3.638458 | 4.126549 | -1.462860 |
| H | 3.264429 | 2.431648 | -2.753663 |
| C | -2.787781 | 3.072318 | -2.673769 |
| C | -3.740456 | 3.713776 | -0.426690 |
| C | -1.656329 | 4.840983 | -1.274919 |
| C | -5.135232 | 0.384541 | 3.480582 |
| H | -6.197236 | -1.461090 | 3.115199 |
| H | -3.869579 | 2.125064 | 3.615926 |
| C | -4.107233 | -2.284831 | -3.460700 |
| H | -5.561350 | -0.802904 | -2.877445 |
| H | -2.493852 | -3.684742 | -3.766507 |
| H | 6.234892 | 1.684666 | 2.729744 |
| H | 3.949732 | -5.091347 | -3.111482 |
| C | 3.319349 | 4.737535 | -0.244432 |
| H | 2.133923 | 4.625420 | 1.553898 |
| H | 4.353702 | 4.593054 | -2.135681 |
| H | -3.334295 | 2.123705 | -2.677922 |
| H | -3.410511 | 3.831981 | -3.165116 |
| H | -1.882325 | 2.929347 | -3.271006 |
| H | -3.504827 | 4.002875 | 0.601265 |
| H | -4.364012 | 4.495237 | -0.879574 |
| H | -4.333356 | 2.790957 | -0.391323 |
| H | -1.391887 | 5.154489 | -0.260875 |
| H | -2.254837 | 5.632467 | -1.744166 |
| H | -0.729208 | 4.733067 | -1.850592 |
| H | -5.871522 | 0.761337 | 4.185951 |
| H | -4.596943 | -2.515909 | -4.402774 |

| | | | |
|---|---|---|---|
| H | 3.788610 | 5.679623 | 0.028632 |

**TS6**

| | | | |
|---|---|---|---|
| Pd | 0.014638 | 0.067259 | 0.644811 |
| P | 2.099680 | -0.567197 | -0.372521 |
| P | -1.406716 | -0.877925 | -1.128591 |
| C | 1.966211 | -1.428901 | -2.020806 |
| C | 2.992231 | -1.800066 | 0.673545 |
| C | 3.359004 | 0.748713 | -0.630418 |
| C | 0.544192 | 0.612108 | 2.387734 |
| C | -1.001163 | -0.259406 | -2.860198 |
| C | -1.367126 | -2.722073 | -1.258049 |
| C | -3.203895 | -0.482449 | -0.983859 |
| C | 1.599242 | -0.525820 | -3.213455 |
| H | 1.214930 | -2.215128 | -1.894155 |
| H | 2.915682 | -1.936359 | -2.231250 |
| C | 3.714427 | -1.346423 | 1.790668 |
| C | 2.920171 | -3.180775 | 0.432093 |
| C | 4.572548 | 0.461824 | -1.280853 |
| C | 3.119979 | 2.046032 | -0.155458 |
| O | 1.113208 | 0.898631 | 3.355246 |
| C | 0.377200 | 0.394563 | -3.071702 |
| H | -1.761917 | 0.501516 | -3.057045 |
| H | -1.169686 | -1.071986 | -3.578019 |
| C | -1.905168 | -3.423403 | -2.351354 |
| C | -0.786666 | -3.456234 | -0.212133 |
| C | -3.572516 | 0.874845 | -1.007645 |
| C | -4.196430 | -1.458158 | -0.815120 |
| H | 2.463293 | 0.108942 | -3.444932 |
| H | 1.463548 | -1.184092 | -4.083880 |

| | | | |
|---|---|---|---|
| C | 4.351843 | -2.250665 | 2.638654 |
| H | 3.783660 | -0.282139 | 1.996507 |
| C | 3.560749 | -4.084303 | 1.284132 |
| H | 2.363718 | -3.564678 | -0.416840 |
| C | 5.526016 | 1.460954 | -1.465278 |
| H | 4.780849 | -0.545131 | -1.633345 |
| C | 4.085607 | 3.042001 | -0.337907 |
| H | 2.190495 | 2.287892 | 0.355944 |
| H | 0.525125 | 1.135020 | -2.279915 |
| H | 0.309857 | 0.967655 | -4.006870 |
| C | -1.857093 | -4.817014 | -2.397527 |
| H | -2.378547 | -2.883430 | -3.166723 |
| C | -0.740198 | -4.852179 | -0.255831 |
| H | -0.359135 | -2.922975 | 0.633184 |
| C | -4.914998 | 1.236537 | -0.900615 |
| H | -2.799247 | 1.635671 | -1.083675 |
| C | -5.538475 | -1.086700 | -0.700646 |
| H | -3.931969 | -2.509559 | -0.775753 |
| C | 4.277798 | -3.623351 | 2.388187 |
| H | 4.908401 | -1.881032 | 3.495571 |
| H | 3.499880 | -5.149984 | 1.078745 |
| C | 5.282033 | 2.755033 | -0.993912 |
| H | 6.460037 | 1.230174 | -1.970869 |
| H | 3.892035 | 4.043001 | 0.037492 |
| C | -1.273986 | -5.534686 | -1.349607 |
| H | -2.277674 | -5.342970 | -3.250508 |
| H | -0.285177 | -5.401903 | 0.563758 |
| C | -5.901964 | 0.259002 | -0.750776 |
| H | -5.188366 | 2.288292 | -0.925557 |
| H | -6.298060 | -1.854010 | -0.574443 |

| | | | |
|---|---|---|---|
| H | 4.776216 | -4.327133 | 3.049213 |
| H | 6.027335 | 3.533334 | -1.135978 |
| H | -1.237788 | -6.620150 | -1.387174 |
| H | -6.946969 | 0.545701 | -0.665479 |
| C | -1.414263 | 0.278207 | 2.309456 |
| C | -2.167241 | 1.451582 | 2.426237 |
| C | -1.838196 | -0.901800 | 2.936361 |
| C | -3.369536 | 1.427380 | 3.137068 |
| H | -1.791770 | 2.370064 | 1.990338 |
| C | -3.046518 | -0.918999 | 3.636167 |
| H | -1.231264 | -1.801133 | 2.883318 |
| C | -3.813922 | 0.244818 | 3.733045 |
| H | -3.957896 | 2.337033 | 3.228000 |
| H | -3.383073 | -1.837263 | 4.111227 |
| H | -4.750385 | 0.232518 | 4.284803 |
| C | -0.280342 | 3.183359 | -0.026014 |
| C | -0.502825 | 4.602266 | -0.641699 |
| O | -0.694997 | 2.197501 | -0.718277 |
| O | 0.290888 | 3.098429 | 1.090635 |
| C | 0.052716 | 5.694146 | 0.284773 |
| C | -2.014536 | 4.820406 | -0.854239 |
| C | 0.213754 | 4.655714 | -2.006469 |
| H | 1.125886 | 5.560331 | 0.453128 |
| H | -0.107920 | 6.687883 | -0.154657 |
| H | -0.432948 | 5.665231 | 1.264897 |
| H | -2.560228 | 4.769333 | 0.096684 |
| H | -2.204682 | 5.808686 | -1.293233 |
| H | -2.427299 | 4.061632 | -1.526201 |
| H | 1.294020 | 4.499907 | -1.892353 |
| H | 0.065295 | 5.632701 | -2.485300 |

| | | | |
|---|---|---|---|
| H | -0.170856 | 3.878460 | -2.673395 |

**7**

| | | | |
|---|---|---|---|
| Pd | -0.020588 | 0.043459 | 0.693065 |
| P | 2.136949 | -0.563342 | -0.393704 |
| C | -1.708825 | 0.013084 | 1.921966 |
| C | 1.975886 | -1.392160 | -2.055185 |
| C | 2.951968 | -1.854695 | 0.645916 |
| C | 3.489079 | 0.661269 | -0.648699 |
| C | 0.822602 | 0.800427 | 2.306534 |
| C | -1.958249 | -1.099472 | 2.733474 |
| C | -2.559244 | 1.119254 | 1.987458 |
| C | 1.640342 | -0.425286 | -3.203508 |
| H | 1.193706 | -2.152197 | -1.955274 |
| H | 2.906664 | -1.925623 | -2.282858 |
| C | 2.816439 | -3.226925 | 0.384473 |
| C | 3.685799 | -1.453592 | 1.775714 |
| C | 4.712170 | 0.263942 | -1.220077 |
| C | 3.306824 | 1.998338 | -0.269320 |
| O | 1.294344 | 1.181083 | 3.275007 |
| C | -3.071353 | -1.116004 | 3.583026 |
| H | -1.293782 | -1.959465 | 2.716415 |
| C | -3.666780 | 1.100699 | 2.841809 |
| H | -2.368633 | 1.983125 | 1.360370 |
| C | 0.431094 | 0.499429 | -3.001706 |
| H | 2.517233 | 0.203263 | -3.399954 |
| H | 1.488819 | -1.030708 | -4.108698 |
| C | 3.403308 | -4.172336 | 1.229887 |
| H | 2.253098 | -3.571977 | -0.476344 |
| C | 4.269319 | -2.398870 | 2.617711 |

| | | | |
|---|---|---|---|
| H | 3.812668 | -0.396695 | 1.993306 |
| C | 5.731134 | 1.192647 | -1.418974 |
| H | 4.875865 | -0.773887 | -1.499017 |
| C | 4.337796 | 2.923786 | -0.467624 |
| H | 2.372656 | 2.323831 | 0.185609 |
| C | -3.927827 | -0.015871 | 3.638745 |
| H | -3.259979 | -1.989083 | 4.204255 |
| H | -4.326214 | 1.964799 | 2.882011 |
| C | -0.935881 | -0.176570 | -2.798859 |
| H | 0.592223 | 1.206134 | -2.181879 |
| H | 0.346400 | 1.113527 | -3.908324 |
| C | 4.129714 | -3.762740 | 2.347870 |
| H | 3.293728 | -5.230768 | 1.007935 |
| H | 4.836258 | -2.068501 | 3.483803 |
| C | 5.543649 | 2.527200 | -1.043271 |
| H | 6.672132 | 0.876258 | -1.861406 |
| H | 4.187747 | 3.956905 | -0.166277 |
| H | -4.789541 | -0.026608 | 4.301577 |
| P | -1.404544 | -0.788223 | -1.076757 |
| H | -1.711421 | 0.554846 | -3.038232 |
| H | -1.066587 | -1.014262 | -3.495317 |
| H | 4.585985 | -4.499155 | 3.003673 |
| H | 6.340260 | 3.250666 | -1.196601 |
| C | -1.409347 | -2.629743 | -1.213776 |
| C | -3.192248 | -0.341837 | -1.017743 |
| C | -0.813540 | -3.393940 | -0.199928 |
| C | -2.003331 | -3.295675 | -2.300547 |
| C | -4.188748 | -1.282601 | -0.724283 |
| C | -3.554759 | 0.998444 | -1.239700 |
| C | -0.802003 | -4.789744 | -0.271825 |

| | | | |
|---|---|---|---|
| H | -0.352468 | -2.886755 | 0.643093 |
| C | -1.990011 | -4.688249 | -2.373445 |
| H | -2.494238 | -2.727937 | -3.086191 |
| C | -5.530615 | -0.897543 | -0.684007 |
| H | -3.926920 | -2.316318 | -0.525777 |
| C | -4.897553 | 1.372819 | -1.204508 |
| H | -2.781536 | 1.747444 | -1.390896 |
| C | -1.387699 | -5.438406 | -1.359224 |
| H | -0.334998 | -5.365103 | 0.522893 |
| H | -2.452890 | -5.188210 | -3.220007 |
| C | -5.889127 | 0.427431 | -0.932002 |
| H | -6.293699 | -1.637738 | -0.458240 |
| H | -5.167227 | 2.411195 | -1.378883 |
| H | -1.379154 | -6.523500 | -1.417306 |
| H | -6.934295 | 0.724250 | -0.903620 |
| C | -0.169874 | 3.224771 | 0.081473 |
| C | -0.450740 | 4.709814 | -0.320031 |
| O | 0.609764 | 3.014801 | 1.050129 |
| O | -0.759313 | 2.333124 | -0.604530 |
| C | 0.432022 | 5.676660 | 0.483093 |
| C | -0.177277 | 4.874782 | -1.827651 |
| C | -1.937515 | 5.006860 | -0.033169 |
| H | 1.494872 | 5.487127 | 0.298251 |
| H | 0.215638 | 6.716174 | 0.202035 |
| H | 0.264147 | 5.563390 | 1.557766 |
| H | 0.878560 | 4.684840 | -2.059810 |
| H | -0.412515 | 5.895833 | -2.156032 |
| H | -0.781334 | 4.171582 | -2.407670 |
| H | -2.162146 | 4.895147 | 1.034979 |
| H | -2.189772 | 6.035829 | -0.321515 |

| | | | |
|---|---|---|---|
| H | -2.586270 | 4.324224 | -0.591297 |

**8**

| | | | |
|---|---|---|---|
| Pd | -0.149280 | 0.503587 | 0.031683 |
| P | 1.533019 | -1.278609 | -0.440105 |
| C | -1.424889 | 1.931185 | 0.738832 |
| C | 0.942042 | -2.715628 | -1.470588 |
| C | 2.008929 | -2.036047 | 1.172083 |
| C | 3.144331 | -0.885189 | -1.248378 |
| C | -2.146088 | 1.707941 | 1.918097 |
| C | -1.499322 | 3.187131 | 0.123859 |
| C | 0.650448 | -2.342275 | -2.936865 |
| H | 0.044587 | -3.112918 | -0.983563 |
| H | 1.693660 | -3.513901 | -1.460614 |
| C | 2.051341 | -3.418800 | 1.407812 |
| C | 2.319791 | -1.154642 | 2.224992 |
| C | 4.218314 | -1.790068 | -1.190652 |
| C | 3.286486 | 0.304955 | -1.976101 |
| C | -2.929897 | 2.724682 | 2.477014 |
| H | -2.104518 | 0.742387 | 2.417628 |
| C | -2.288421 | 4.199712 | 0.681940 |
| H | -0.912090 | 3.390089 | -0.766153 |
| C | -0.265886 | -1.134262 | -3.192320 |
| H | 1.603372 | -2.149426 | -3.444200 |
| H | 0.217709 | -3.227022 | -3.425061 |
| C | 2.408893 | -3.913182 | 2.665164 |
| H | 1.802134 | -4.122740 | 0.620238 |
| C | 2.681647 | -1.654771 | 3.475982 |
| H | 2.263138 | -0.081311 | 2.060064 |
| C | 5.409808 | -1.514436 | -1.859583 |

| | | | |
|---|---|---|---|
| H | 4.130520 | -2.704684 | -0.609908 |
| C | 4.484005 | 0.574677 | -2.646638 |
| H | 2.484660 | 1.038608 | -1.987293 |
| C | -3.006943 | 3.972808 | 1.857751 |
| H | -3.480353 | 2.535654 | 3.396554 |
| H | -2.332358 | 5.172950 | 0.196877 |
| C | -1.694282 | -1.210725 | -2.621292 |
| H | 0.212401 | -0.211243 | -2.843911 |
| H | -0.358792 | -1.030126 | -4.281543 |
| C | 2.727854 | -3.033350 | 3.700015 |
| H | 2.437507 | -4.986982 | 2.832077 |
| H | 2.921681 | -0.963275 | 4.279226 |
| C | 5.542892 | -0.331455 | -2.592882 |
| H | 6.235804 | -2.218868 | -1.804147 |
| H | 4.586103 | 1.503957 | -3.200314 |
| H | -3.617781 | 4.762174 | 2.289181 |
| P | -1.948665 | -0.689031 | -0.826531 |
| H | -2.332841 | -0.530093 | -3.192652 |
| H | -2.115712 | -2.213842 | -2.758734 |
| H | 3.006932 | -3.419253 | 4.677032 |
| H | 6.473464 | -0.115599 | -3.111504 |
| C | -2.277093 | -2.247000 | 0.106729 |
| C | -3.608662 | 0.113739 | -0.938718 |
| C | -1.710978 | -2.407614 | 1.380452 |
| C | -3.091221 | -3.272642 | -0.405874 |
| C | -4.723406 | -0.336161 | -0.219443 |
| C | -3.746627 | 1.239934 | -1.767760 |
| C | -1.944972 | -3.568082 | 2.122583 |
| H | -1.073229 | -1.625245 | 1.782233 |
| C | -3.322725 | -4.431482 | 0.334797 |

| | | | |
|---|---|---|---|
| H | -3.561728 | -3.165073 | -1.379268 |
| C | -5.955451 | 0.310930 | -0.345755 |
| H | -4.639354 | -1.191361 | 0.442096 |
| C | -4.977404 | 1.879793 | -1.896196 |
| H | -2.885685 | 1.634953 | -2.300246 |
| C | -2.748653 | -4.581636 | 1.600508 |
| H | -1.490611 | -3.677902 | 3.103012 |
| H | -3.953383 | -5.215877 | -0.074988 |
| C | -6.087680 | 1.414549 | -1.187076 |
| H | -6.810637 | -0.051934 | 0.218189 |
| H | -5.065507 | 2.750165 | -2.540466 |
| H | -2.929116 | -5.486050 | 2.175242 |
| H | -7.046419 | 1.916649 | -1.283983 |
| C | 1.826498 | 2.627676 | 0.138836 |
| C | 2.791067 | 3.652445 | 0.795550 |
| O | 1.409732 | 1.670352 | 0.903684 |
| O | 1.492444 | 2.783122 | -1.048146 |
| C | 3.261414 | 3.203425 | 2.187533 |
| C | 4.003479 | 3.846930 | -0.135919 |
| C | 2.013851 | 4.982853 | 0.909179 |
| H | 3.812221 | 2.257482 | 2.135221 |
| H | 3.929049 | 3.959372 | 2.621585 |
| H | 2.414772 | 3.060514 | 2.865161 |
| H | 4.584700 | 2.921165 | -0.228894 |
| H | 4.669252 | 4.624655 | 0.259651 |
| H | 3.672915 | 4.141025 | -1.135760 |
| H | 1.128801 | 4.870370 | 1.546219 |
| H | 2.652801 | 5.761345 | 1.345684 |
| H | 1.681146 | 5.317474 | -0.078145 |

**9**

| | | | |
|---|---|---|---|
| Pd | d -2.474078 | 0.573214 | -0.510002 |
| C | -3.904382 | -0.639035 | -1.275872 |
| C | -4.080382 | -0.720036 | -2.666983 |
| C | -4.858720 | -1.244293 | -0.446616 |
| C | -5.180536 | -1.388864 | -3.213236 |
| H | -3.368557 | -0.244078 | -3.338544 |
| C | -5.964005 | -1.908872 | -0.993550 |
| H | -4.750680 | -1.201981 | 0.633712 |
| C | -6.126703 | -1.986924 | -2.377279 |
| H | -5.299116 | -1.436792 | -4.293631 |
| H | -6.694607 | -2.368441 | -0.331437 |
| H | -6.981953 | -2.506887 | -2.801309 |
| C | -1.086336 | 2.389463 | 1.423501 |
| C | -0.254565 | 3.638704 | 1.835962 |
| O | -1.106240 | 2.098807 | 0.166728 |
| O | -1.683497 | 1.756786 | 2.315546 |
| C | 0.497756 | 4.260097 | 0.649777 |
| C | 0.744531 | 3.210498 | 2.930427 |
| C | -1.235743 | 4.668740 | 2.437632 |
| H | -0.189455 | 4.571807 | -0.142747 |
| H | 1.064012 | 5.140335 | 0.980759 |
| H | 1.203568 | 3.548757 | 0.208065 |
| H | 1.485969 | 2.503373 | 2.538061 |
| H | 1.290269 | 4.081603 | 3.313990 |
| H | 0.219669 | 2.729838 | 3.760479 |
| H | -1.958743 | 5.017860 | 1.689965 |
| H | -0.690028 | 5.546189 | 2.806888 |
| H | -1.792095 | 4.226704 | 3.269281 |
| Si | -4.508767 | 2.963575 | -0.807028 |

| | | | |
|---|---|---|---|
| C | -5.973204 | 2.646015 | -1.952054 |
| C | -3.710635 | 4.642361 | -1.119932 |
| C | -4.942678 | 2.677383 | 1.001011 |
| H | -6.804500 | 3.320641 | -1.710086 |
| H | -5.703844 | 2.809205 | -3.001751 |
| H | -6.331710 | 1.615877 | -1.852605 |
| H | -3.491854 | 4.784293 | -2.184510 |
| H | -4.371317 | 5.457720 | -0.799561 |
| H | -2.768539 | 4.729707 | -0.569324 |
| H | -5.484803 | 1.732659 | 1.121441 |
| H | -4.046498 | 2.629585 | 1.628758 |
| H | -5.587139 | 3.484991 | 1.372097 |
| H | -3.443228 | 1.957163 | -1.263964 |
| P | -1.332314 | -1.263343 | 0.307368 |
| C | 0.282405 | -0.799878 | 1.108296 |
| C | -0.817251 | -2.586079 | -0.862829 |
| C | -2.263701 | -2.085790 | 1.662037 |
| C | 1.322882 | -0.235190 | 0.129370 |
| H | 0.662376 | -1.704326 | 1.601541 |
| H | 0.040901 | -0.071102 | 1.887607 |
| C | -1.008966 | -2.431256 | -2.241701 |
| C | -0.132987 | -3.723509 | -0.396211 |
| C | -2.534363 | -1.330911 | 2.818452 |
| C | -2.751898 | -3.397226 | 1.564520 |
| C | 2.689373 | -0.025484 | 0.802557 |
| H | 0.950318 | 0.713774 | -0.271601 |
| H | 1.449810 | -0.920344 | -0.719101 |
| C | -0.540020 | -3.396095 | -3.136532 |
| H | -1.530151 | -1.556776 | -2.612128 |
| C | 0.331018 | -4.687302 | -1.290363 |

| | | | |
|---|---|---|---|
| H | 0.033223 | -3.863387 | 0.668142 |
| C | -3.252084 | -1.903486 | 3.869236 |
| H | -2.212161 | -0.293836 | 2.878100 |
| C | -3.472471 | -3.959300 | 2.620583 |
| H | -2.578220 | -3.981771 | 0.667506 |
| C | 3.724764 | 0.573651 | -0.161101 |
| H | 3.056016 | -0.986682 | 1.187230 |
| H | 2.575398 | 0.633889 | 1.673190 |
| C | 0.127384 | -4.525490 | -2.663439 |
| H | -0.699441 | -3.262019 | -4.202890 |
| H | 0.854116 | -5.562543 | -0.914808 |
| C | -3.718458 | -3.217309 | 3.776510 |
| H | -3.453091 | -1.314693 | 4.760220 |
| H | -3.844289 | -4.976846 | 2.533951 |
| P | 5.394452 | 0.826913 | 0.659022 |
| H | 3.378222 | 1.559720 | -0.497317 |
| H | 3.823378 | -0.060963 | -1.051973 |
| H | 0.490615 | -5.276774 | -3.359493 |
| H | -4.278875 | -3.657030 | 4.597409 |
| C | 6.438185 | 1.429243 | -0.748034 |
| C | 5.965478 | -0.935009 | 0.807885 |
| C | 7.812343 | 1.606785 | -0.501329 |
| C | 5.940734 | 1.791681 | -2.009260 |
| C | 6.330208 | -1.730230 | -0.291400 |
| C | 6.015613 | -1.502084 | 2.090714 |
| C | 8.661304 | 2.108962 | -1.485282 |
| H | 8.220068 | 1.345820 | 0.472820 |
| C | 6.789889 | 2.306900 | -2.993593 |
| H | 4.886079 | 1.675878 | -2.237964 |
| C | 6.727067 | -3.055293 | -0.111678 |

| | | | |
|---|---|---|---|
| H | 6.314728 | -1.305550 | -1.291692 |
| C | 6.407061 | -2.831361 | 2.272478 |
| H | 5.751229 | -0.894737 | 2.953091 |
| C | 8.151420 | 2.463840 | -2.737586 |
| H | 9.720450 | 2.230779 | -1.273078 |
| H | 6.382041 | 2.582079 | -3.963078 |
| C | 6.764319 | -3.609764 | 1.171151 |
| H | 7.009973 | -3.656242 | -0.972452 |
| H | 6.439941 | -3.253674 | 3.273587 |
| H | 8.810476 | 2.863266 | -3.503849 |
| H | 7.076343 | -4.641702 | 1.310029 |

**TS10**

| | | | |
|---|---|---|---|
| Pd | 2.689923 | -0.791848 | -0.411871 |
| C | 4.308526 | 0.362342 | -1.172318 |
| C | 4.400739 | 0.704793 | -2.530454 |
| C | 5.151307 | 1.001272 | -0.249986 |
| C | 5.276291 | 1.708998 | -2.950122 |
| H | 3.792958 | 0.181569 | -3.264415 |
| C | 6.026415 | 2.004648 | -0.673052 |
| H | 5.113413 | 0.727567 | 0.800460 |
| C | 6.087230 | 2.365027 | -2.021396 |
| H | 5.329545 | 1.972372 | -4.003660 |
| H | 6.660391 | 2.504805 | 0.054667 |
| H | 6.773889 | 3.141233 | -2.348691 |
| C | 1.070792 | -2.361092 | 1.507626 |
| C | 0.091360 | -3.482966 | 1.962065 |
| O | 1.185208 | -2.186856 | 0.231289 |
| O | 1.705651 | -1.730588 | 2.370453 |
| C | -0.876918 | -3.907497 | 0.846645 |

| | | | |
|---|---|---|---|
| C | -0.697516 | -2.986789 | 3.188784 |
| C | 0.958012 | -4.692856 | 2.380568 |
| H | -0.341656 | -4.289806 | -0.026946 |
| H | -1.547649 | -4.695994 | 1.211386 |
| H | -1.497083 | -3.067927 | 0.513651 |
| H | -1.356881 | -2.150072 | 2.926698 |
| H | -1.326911 | -3.790829 | 3.589945 |
| H | -0.015299 | -2.645688 | 3.971225 |
| H | 1.536154 | -5.080019 | 1.533109 |
| H | 0.321019 | -5.505766 | 2.751265 |
| H | 1.656808 | -4.412506 | 3.175351 |
| Si | 4.044718 | -2.960600 | -0.911050 |
| C | 5.645114 | -2.955022 | -1.940526 |
| C | 2.741891 | -3.950433 | -1.851704 |
| C | 4.450517 | -3.594483 | 0.820692 |
| H | 5.997639 | -3.987303 | -2.069798 |
| H | 5.489390 | -2.529838 | -2.938672 |
| H | 6.445941 | -2.386155 | -1.455073 |
| H | 2.647048 | -3.589116 | -2.882428 |
| H | 3.034571 | -5.008401 | -1.893641 |
| H | 1.770496 | -3.862804 | -1.360276 |
| H | 5.350187 | -3.095141 | 1.199615 |
| H | 3.642657 | -3.407876 | 1.531906 |
| H | 4.655368 | -4.673230 | 0.791533 |
| H | 4.209923 | -1.112550 | -1.036783 |
| P | 1.383675 | 1.159033 | 0.298542 |
| C | -0.266781 | 0.834577 | 1.107243 |
| C | 0.930436 | 2.423419 | -0.964427 |
| C | 2.313317 | 2.059152 | 1.611200 |
| C | -1.313454 | 0.255125 | 0.143448 |

| | | | |
|---|---|---|---|
| H | -0.620399 | 1.778695 | 1.541329 |
| H | -0.073558 | 0.142703 | 1.932840 |
| C | 1.128309 | 2.136707 | -2.322115 |
| C | 0.302310 | 3.632204 | -0.614621 |
| C | 2.539059 | 1.386164 | 2.826908 |
| C | 2.862651 | 3.336728 | 1.426618 |
| C | -2.688979 | 0.093577 | 0.811429 |
| H | -0.958626 | -0.713143 | -0.228287 |
| H | -1.420113 | 0.913570 | -0.728554 |
| C | 0.720369 | 3.038005 | -3.308686 |
| H | 1.601116 | 1.201649 | -2.603526 |
| C | -0.101546 | 4.533609 | -1.598955 |
| H | 0.131718 | 3.874365 | 0.430680 |
| C | 3.266787 | 2.004554 | 3.844235 |
| H | 2.173516 | 0.371404 | 2.961072 |
| C | 3.594504 | 3.946382 | 2.448711 |
| H | 2.724900 | 3.861151 | 0.487032 |
| C | -3.738928 | -0.486688 | -0.148291 |
| H | -3.028540 | 1.070095 | 1.181881 |
| H | -2.598761 | -0.557218 | 1.691413 |
| C | 0.108057 | 4.238355 | -2.948953 |
| H | 0.881025 | 2.799511 | -4.356693 |
| H | -0.583649 | 5.464518 | -1.312497 |
| C | 3.792276 | 3.286336 | 3.661818 |
| H | 3.429258 | 1.476502 | 4.780233 |
| H | 4.010221 | 4.938450 | 2.291652 |
| P | -5.413179 | -0.696957 | 0.674379 |
| H | -3.417001 | -1.482944 | -0.479118 |
| H | -3.822768 | 0.144936 | -1.042768 |
| H | -0.208936 | 4.940845 | -3.715165 |

| | | | |
|---|---|---|---|
| H | 4.359614 | 3.763128 | 4.456974 |
| C | -6.471623 | -1.285603 | -0.727586 |
| C | -5.945720 | 1.077673 | 0.813179 |
| C | -7.847714 | -1.439199 | -0.475592 |
| C | -5.985054 | -1.659684 | -1.989664 |
| C | -6.298583 | 1.872970 | -0.289882 |
| C | -5.977836 | 1.654363 | 2.092303 |
| C | -8.708667 | -1.929443 | -1.455145 |
| H | -8.247313 | -1.169027 | 0.499420 |
| C | -6.846351 | -2.163039 | -2.969584 |
| H | -4.929414 | -1.562201 | -2.222361 |
| C | -6.666356 | 3.207373 | -0.117455 |
| H | -6.296622 | 1.441347 | -1.287312 |
| C | -6.339898 | 2.992887 | 2.266665 |
| H | -5.722310 | 1.047459 | 2.957651 |
| C | -8.209418 | -2.296224 | -2.708308 |
| H | -9.768948 | -2.032784 | -1.238813 |
| H | -6.446776 | -2.447601 | -3.939826 |
| C | -6.685702 | 3.771228 | 1.161645 |
| H | -6.940305 | 3.808405 | -0.981067 |
| H | -6.358735 | 3.422676 | 3.264943 |
| H | -8.877925 | -2.686461 | -3.471124 |
| H | -6.974766 | 4.810573 | 1.294815 |

**11**

| | | | |
|---|---|---|---|
| Pd | 2.701396 | -1.063935 | -0.513183 |
| C | 5.085552 | -0.937338 | -0.734436 |
| C | 4.606557 | -0.315975 | -1.910613 |
| C | 5.592371 | -0.138706 | 0.318660 |
| C | 4.703909 | 1.090624 | -2.043877 |

| | | | |
|---|---|---|---|
| H | 4.345509 | -0.914046 | -2.776293 |
| C | 5.663618 | 1.235804 | 0.174726 |
| H | 5.941530 | -0.617016 | 1.228415 |
| C | 5.237057 | 1.852361 | -1.019499 |
| H | 4.372153 | 1.563810 | -2.963180 |
| H | 6.054111 | 1.847204 | 0.982747 |
| H | 5.318359 | 2.929748 | -1.124864 |
| C | 1.417378 | -1.891694 | 1.925512 |
| C | 0.492910 | -2.687972 | 2.891185 |
| O | 1.213726 | -2.079104 | 0.654872 |
| O | 2.288585 | -1.149092 | 2.399472 |
| C | -0.614539 | -3.458354 | 2.156734 |
| C | -0.128303 | -1.693855 | 3.893450 |
| C | 1.393071 | -3.675813 | 3.667369 |
| H | -0.198517 | -4.186603 | 1.454053 |
| H | -1.238406 | -3.999024 | 2.879841 |
| H | -1.264150 | -2.783479 | 1.589413 |
| H | -0.804987 | -0.991394 | 3.391835 |
| H | -0.711942 | -2.231228 | 4.651024 |
| H | 0.652969 | -1.116763 | 4.395418 |
| H | 1.843696 | -4.418704 | 2.998284 |
| H | 0.802121 | -4.217397 | 4.416614 |
| H | 2.200038 | -3.140969 | 4.176489 |
| Si | 2.730779 | -3.283308 | -1.346468 |
| C | 3.887034 | -3.508769 | -2.853728 |
| C | 0.975575 | -3.646789 | -1.962632 |
| C | 3.287426 | -4.541043 | -0.041352 |
| H | 3.823112 | -4.559064 | -3.171407 |
| H | 3.579453 | -2.895398 | -3.708937 |
| H | 4.942237 | -3.300346 | -2.643896 |

| | | | |
|---|---|---|---|
| H | 0.712832 | -3.006201 | -2.812757 |
| H | 0.901213 | -4.691589 | -2.295195 |
| H | 0.249455 | -3.478168 | -1.164228 |
| H | 4.318616 | -4.360192 | 0.284462 |
| H | 2.643725 | -4.497834 | 0.840610 |
| H | 3.241823 | -5.558988 | -0.452271 |
| H | 5.235772 | -2.009830 | -0.695696 |
| P | 1.610154 | 1.363095 | -0.001568 |
| C | -0.030664 | 1.221421 | 0.896768 |
| C | 1.086546 | 2.351355 | -1.479374 |
| C | 2.494201 | 2.558109 | 1.100960 |
| C | -1.090453 | 0.423137 | 0.122834 |
| H | -0.395916 | 2.232001 | 1.119850 |
| H | 0.186678 | 0.738829 | 1.855477 |
| C | 1.302030 | 1.811148 | -2.756388 |
| C | 0.399249 | 3.574433 | -1.381483 |
| C | 2.820530 | 2.107719 | 2.394339 |
| C | 2.912391 | 3.838522 | 0.707977 |
| C | -2.449309 | 0.412430 | 0.842784 |
| H | -0.732737 | -0.603456 | -0.017366 |
| H | -1.224702 | 0.856564 | -0.877090 |
| C | 0.861254 | 2.476435 | -3.903635 |
| H | 1.808931 | 0.854491 | -2.847464 |
| C | -0.037469 | 4.243327 | -2.525325 |
| H | 0.204128 | 4.011141 | -0.405982 |
| C | 3.517563 | 2.935487 | 3.274786 |
| H | 2.554589 | 1.098690 | 2.699337 |
| C | 3.619314 | 4.659805 | 1.591065 |
| H | 2.690762 | 4.203092 | -0.289696 |
| C | -3.507514 | -0.402581 | 0.083656 |

| | | | |
|---|---|---|---|
| H | -2.800931 | 1.445069 | 0.970315 |
| H | -2.328906 | -0.000149 | 1.853631 |
| C | 0.194998 | 3.696760 | -3.790279 |
| H | 1.034940 | 2.038071 | -4.883038 |
| H | -0.564586 | 5.188976 | -2.428804 |
| C | 3.918742 | 4.214476 | 2.878825 |
| H | 3.755457 | 2.574249 | 4.272138 |
| H | 3.932160 | 5.650084 | 1.268844 |
| P | -5.157131 | -0.430434 | 0.978747 |
| H | -3.170867 | -1.444377 | -0.000624 |
| H | -3.625410 | -0.012409 | -0.936119 |
| H | -0.148382 | 4.217072 | -4.680499 |
| H | 4.464332 | 4.855368 | 3.566654 |
| C | -6.233331 | -1.370983 | -0.200052 |
| C | -5.730720 | 1.312283 | 0.684866 |
| C | -7.597615 | -1.487036 | 0.125477 |
| C | -5.768364 | -2.035305 | -1.345493 |
| C | -6.126811 | 1.799823 | -0.571766 |
| C | -5.749943 | 2.189756 | 1.780022 |
| C | -8.468711 | -2.223050 | -0.674889 |
| H | -7.979860 | -0.992667 | 1.015891 |
| C | -6.639404 | -2.784181 | -2.142951 |
| H | -4.722184 | -1.975449 | -1.628314 |
| C | -6.524121 | 3.127757 | -0.727811 |
| H | -6.134945 | 1.132824 | -1.429803 |
| C | -6.141527 | 3.522284 | 1.624715 |
| H | -5.460683 | 1.823393 | 2.762142 |
| C | -7.991025 | -2.877944 | -1.813803 |
| H | -9.519763 | -2.292513 | -0.406243 |
| H | -6.256539 | -3.291479 | -3.025040 |

| | | | |
|---|---|---|---|
| C | -6.530276 | 3.993008 | 0.370227 |
| H | -6.831258 | 3.488558 | -1.706388 |
| H | -6.149621 | 4.187590 | 2.484347 |
| H | -8.667154 | -3.459359 | -2.435017 |
| H | -6.842290 | 5.027017 | 0.247645 |

**12**

| | | | |
|---|---|---|---|
| Pd | -0.292903 | -0.876750 | 0.063244 |
| P | 1.308409 | 1.174353 | 0.721927 |
| P | -2.183296 | 0.367969 | 0.621419 |
| C | 0.610005 | 2.338975 | 2.011060 |
| C | 1.845484 | 2.338943 | -0.613084 |
| C | 2.888052 | 0.679564 | 1.548628 |
| C | -2.131742 | 0.865511 | 2.445009 |
| C | -2.212258 | 1.947700 | -0.334734 |
| C | -3.959665 | -0.164039 | 0.489586 |
| C | 0.184342 | 1.664655 | 3.325634 |
| H | -0.241767 | 2.850051 | 1.545981 |
| H | 1.354452 | 3.107437 | 2.248236 |
| C | 2.160065 | 1.792223 | -1.870176 |
| C | 1.938025 | 3.730423 | -0.440610 |
| C | 4.000524 | 1.534626 | 1.609544 |
| C | 2.953456 | -0.566796 | 2.191207 |
| C | -0.840257 | 0.524309 | 3.217873 |
| H | -2.974261 | 0.346582 | 2.907902 |
| H | -2.361187 | 1.934781 | 2.520274 |
| C | -2.948408 | 3.070265 | 0.083773 |
| C | -1.512371 | 2.010632 | -1.548715 |
| C | -4.489647 | -1.084917 | 1.408773 |
| C | -4.791692 | 0.301356 | -0.540416 |

| | | | |
|---|---|---|---|
| H | 1.075042 | 1.281648 | 3.838628 |
| H | -0.228683 | 2.448583 | 3.976546 |
| C | 2.573767 | 2.620133 | -2.915582 |
| H | 2.066789 | 0.721139 | -2.038275 |
| C | 2.344781 | 4.554329 | -1.492509 |
| H | 1.690025 | 4.190542 | 0.509966 |
| C | 5.147283 | 1.156910 | 2.308744 |
| H | 3.976314 | 2.493998 | 1.100583 |
| C | 4.099248 | -0.937320 | 2.900441 |
| H | 2.124384 | -1.260455 | 2.102230 |
| H | -0.376156 | -0.363636 | 2.770866 |
| H | -1.119243 | 0.237782 | 4.240178 |
| C | -2.968144 | 4.231688 | -0.687578 |
| H | -3.524973 | 3.037241 | 1.004285 |
| C | -1.538277 | 3.173329 | -2.323837 |
| H | -0.937138 | 1.149934 | -1.879345 |
| C | -5.811946 | -1.518033 | 1.309715 |
| H | -3.869985 | -1.480432 | 2.208318 |
| C | -6.115294 | -0.132607 | -0.639769 |
| H | -4.409866 | 1.004617 | -1.272276 |
| C | 2.667599 | 4.001337 | -2.732424 |
| H | 2.816533 | 2.178624 | -3.878686 |
| H | 2.408475 | 5.628720 | -1.339017 |
| C | 5.196907 | -0.077747 | 2.960884 |
| H | 6.003012 | 1.826390 | 2.342098 |
| H | 4.136388 | -1.905877 | 3.392027 |
| C | -2.261671 | 4.284928 | -1.893061 |
| H | -3.538682 | 5.093209 | -0.350714 |
| H | -0.977756 | 3.208883 | -3.253189 |
| C | -6.631036 | -1.041545 | 0.284470 |

| | | | |
|---|---|---|---|
| H | -6.199063 | -2.230496 | 2.033107 |
| H | -6.742065 | 0.243201 | -1.444042 |
| H | 2.986036 | 4.643040 | -3.550125 |
| H | 6.090969 | -0.371216 | 3.504775 |
| H | -2.276965 | 5.191459 | -2.492123 |
| H | -7.660969 | -1.378434 | 0.205834 |
| C | 2.066981 | -2.001490 | -1.250629 |
| C | 3.404977 | -2.786397 | -1.371703 |
| O | 1.433639 | -2.114696 | -0.129770 |
| O | 1.692293 | -1.303003 | -2.208155 |
| C | 3.388620 | -3.571013 | -2.699532 |
| C | 3.633252 | -3.748216 | -0.195523 |
| C | 4.542495 | -1.741668 | -1.419621 |
| H | 3.187317 | -2.899003 | -3.537941 |
| H | 4.356822 | -4.061573 | -2.862595 |
| H | 2.614915 | -4.347527 | -2.693100 |
| H | 2.832462 | -4.491992 | -0.125593 |
| H | 4.582729 | -4.283674 | -0.327972 |
| H | 3.673318 | -3.210835 | 0.755967 |
| H | 4.387676 | -1.042688 | -2.247415 |
| H | 5.509676 | -2.240266 | -1.563182 |
| H | 4.595622 | -1.166256 | -0.488252 |
| Si | -1.443850 | -2.884423 | -0.614439 |
| C | -0.327262 | -4.084072 | -1.571628 |
| H | 0.473426 | -4.483130 | -0.944048 |
| H | -0.942737 | -4.923573 | -1.924826 |
| H | 0.125717 | -3.602292 | -2.442915 |
| C | -2.017790 | -3.837231 | 0.934915 |
| H | -2.442060 | -4.806253 | 0.636525 |
| H | -1.163906 | -4.036689 | 1.593069 |

| | | | |
|---|---|---|---|
| H | -2.778058 | -3.306920 | 1.515390 |
| C | -2.906264 | -2.588392 | -1.799362 |
| H | -2.569905 | -2.031184 | -2.682328 |
| H | -3.273521 | -3.563771 | -2.148381 |
| H | -3.748693 | -2.052615 | -1.358913 |

**TS13**

| | | | |
|---|---|---|---|
| Pd | -0.173709 | 0.615427 | -0.036573 |
| P | 1.380494 | -1.393361 | -0.509249 |
| P | -2.178923 | -0.303701 | -0.773560 |
| C | 0.520545 | -2.628249 | -1.626599 |
| C | 1.681922 | -2.375317 | 1.027943 |
| C | 3.051927 | -1.331452 | -1.307171 |
| C | -2.013944 | -0.847146 | -2.584114 |
| C | -2.735527 | -1.846435 | 0.086542 |
| C | -3.749144 | 0.685487 | -0.888186 |
| C | 0.233898 | -2.112253 | -3.048561 |
| H | -0.414683 | -2.903590 | -1.124851 |
| H | 1.124307 | -3.540473 | -1.708858 |
| C | 2.181964 | -1.679812 | 2.145178 |
| C | 1.409376 | -3.746975 | 1.154531 |
| C | 4.045199 | -2.298381 | -1.084491 |
| C | 3.319163 | -0.287387 | -2.205853 |
| C | -0.596261 | -0.824471 | -3.191147 |
| H | -2.648675 | -0.158898 | -3.150089 |
| H | -2.464736 | -1.840901 | -2.693409 |
| C | -3.757551 | -2.669500 | -0.420304 |
| C | -2.103180 | -2.220108 | 1.280833 |
| C | -3.778013 | 1.788861 | -1.760105 |
| C | -4.882097 | 0.432215 | -0.100820 |

| | | | |
|---|---|---|---|
| H | 1.188343 | -1.958387 | -3.567926 |
| H | -0.279330 | -2.918226 | -3.592917 |
| C | 2.416729 | -2.348982 | 3.346836 |
| H | 2.380485 | -0.614637 | 2.066048 |
| C | 1.636781 | -4.410319 | 2.363754 |
| H | 1.014962 | -4.312060 | 0.316563 |
| C | 5.269547 | -2.224804 | -1.750009 |
| H | 3.865157 | -3.106782 | -0.381682 |
| C | 4.539972 | -0.219807 | -2.880998 |
| H | 2.570410 | 0.484005 | -2.367473 |
| H | -0.044961 | 0.031783 | -2.782555 |
| H | -0.699641 | -0.632940 | -4.267974 |
| C | -4.130829 | -3.834490 | 0.249169 |
| H | -4.276364 | -2.394387 | -1.334882 |
| C | -2.475744 | -3.388199 | 1.952158 |
| H | -1.305667 | -1.596716 | 1.674545 |
| C | -4.912698 | 2.592229 | -1.864686 |
| H | -2.901102 | 2.032721 | -2.355763 |
| C | -6.016100 | 1.243784 | -0.198460 |
| H | -4.887950 | -0.403619 | 0.590869 |
| C | 2.143406 | -3.714473 | 3.461851 |
| H | 2.808376 | -1.797875 | 4.197987 |
| H | 1.419230 | -5.472671 | 2.442538 |
| C | 5.518712 | -1.188259 | -2.652826 |
| H | 6.030776 | -2.977813 | -1.561983 |
| H | 4.729437 | 0.596030 | -3.573753 |
| C | -3.489006 | -4.196608 | 1.437477 |
| H | -4.922979 | -4.459329 | -0.155252 |
| H | -1.965457 | -3.664808 | 2.870383 |
| C | -6.039085 | 2.321234 | -1.083204 |

| | | | |
|---|---|---|---|
| H | -4.913384 | 3.435503 | -2.550389 |
| H | -6.883312 | 1.026962 | 0.419957 |
| H | 2.321555 | -4.231611 | 4.401266 |
| H | 6.473232 | -1.132315 | -3.169565 |
| H | -3.779523 | -5.105950 | 1.957051 |
| H | -6.922370 | 2.949360 | -1.160066 |
| C | 2.181898 | 2.418870 | 0.770358 |
| C | 3.094153 | 3.610947 | 0.381659 |
| O | 0.924673 | 2.511319 | 0.349119 |
| O | 2.633150 | 1.460063 | 1.381486 |
| C | 2.299504 | 4.862269 | -0.024420 |
| C | 3.955391 | 3.140246 | -0.813300 |
| C | 4.014975 | 3.928697 | 1.576671 |
| H | 1.670433 | 5.225678 | 0.795202 |
| H | 2.992642 | 5.668219 | -0.296489 |
| H | 1.650401 | 4.662458 | -0.881458 |
| H | 3.331477 | 2.929053 | -1.689628 |
| H | 4.670563 | 3.924769 | -1.089985 |
| H | 4.513099 | 2.233191 | -0.562342 |
| H | 3.438041 | 4.282175 | 2.439232 |
| H | 4.728944 | 4.715034 | 1.302879 |
| H | 4.569998 | 3.039040 | 1.884215 |
| Si | -0.785280 | 2.511697 | 1.479708 |
| C | -2.145205 | 1.531201 | 2.429840 |
| H | -2.964943 | 1.200485 | 1.788336 |
| H | -1.729011 | 0.655425 | 2.940453 |
| H | -2.562207 | 2.194401 | 3.203320 |
| C | -1.623672 | 3.928344 | 0.550785 |
| H | -2.399220 | 3.562037 | -0.128599 |
| H | -2.106453 | 4.598812 | 1.275291 |

| | | | |
|---|---|---|---|
| H | -0.900500 | 4.512637 | -0.025206 |
| C | 0.219763 | 3.169980 | 2.960266 |
| H | 0.891886 | 2.403556 | 3.362141 |
| H | 0.830621 | 4.041065 | 2.699291 |
| H | -0.464146 | 3.465391 | 3.765662 |

**14**

| | | | |
|---|---|---|---|
| Si | 1.878787 | 0.065298 | 0.000001 |
| C | 2.291454 | -0.891378 | -1.565687 |
| H | 2.030625 | -0.315028 | -2.461299 |
| H | 3.365616 | -1.110364 | -1.614139 |
| H | 1.746184 | -1.839391 | -1.598991 |
| C | 2.291500 | -0.891336 | 1.565701 |
| H | 3.365662 | -1.110325 | 1.614126 |
| H | 2.030701 | -0.314960 | 2.461306 |
| H | 1.746228 | -1.839346 | 1.599049 |
| C | 2.705217 | 1.756008 | -0.000034 |
| H | 2.422212 | 2.337142 | -0.885307 |
| H | 2.422234 | 2.337167 | 0.885229 |
| H | 3.798108 | 1.660067 | -0.000047 |
| O | 0.199520 | 0.484211 | 0.000020 |
| C | -0.777957 | -0.447391 | 0.000043 |
| C | -2.185795 | 0.162158 | 0.000002 |
| O | -0.543738 | -1.641110 | 0.000007 |
| C | -2.350348 | 1.037507 | -1.262691 |
| C | -3.224588 | -0.969837 | -0.000052 |
| C | -2.350435 | 1.037467 | 1.262712 |
| H | -2.228875 | 0.443113 | -2.175986 |
| H | -3.354686 | 1.476885 | -1.280020 |
| H | -1.618161 | 1.849745 | -1.281208 |

| | | | |
|---|---|---|---|
| H | -3.115662 | -1.607555 | 0.882432 |
| H | -4.236102 | -0.547379 | -0.000081 |
| H | -3.115600 | -1.607529 | -0.882547 |
| H | -2.229021 | 0.443044 | 2.175996 |
| H | -3.354776 | 1.476840 | 1.279987 |
| H | -1.618252 | 1.849707 | 1.281304 |

**15**

| | | | |
|---|---|---|---|
| Pd | 0.092952 | -0.334298 | -0.279501 |
| P | 2.237151 | -0.164149 | 0.603274 |
| C | 0.647909 | -1.976882 | -1.368197 |
| C | 2.505106 | 1.318613 | 1.714511 |
| C | 3.665130 | -0.053288 | -0.556997 |
| C | 2.637017 | -1.576552 | 1.712451 |
| O | -1.905767 | -0.943873 | -0.882913 |
| C | 0.429976 | -1.898899 | -2.917084 |
| O | 1.039771 | -2.998326 | -0.862155 |
| C | 1.873655 | 1.229366 | 3.119012 |
| H | 2.130727 | 2.195021 | 1.176691 |
| H | 3.587561 | 1.454699 | 1.827456 |
| C | 4.096637 | 1.193452 | -1.039947 |
| C | 4.293346 | -1.216643 | -1.031393 |
| C | 3.929969 | -1.747128 | 2.236718 |
| C | 1.614197 | -2.453122 | 2.104819 |
| C | -2.492254 | -1.451552 | 0.150634 |
| C | 0.072284 | -0.489944 | -3.409599 |
| C | 1.737349 | -2.375536 | -3.585131 |
| C | -0.711900 | -2.886895 | -3.244839 |
| C | 0.369892 | 0.928542 | 3.233594 |
| H | 2.410044 | 0.461501 | 3.688284 |

| | | | |
|---|---|---|---|
| H | 2.083176 | 2.184188 | 3.622813 |
| C | 5.139921 | 1.274368 | -1.963828 |
| H | 3.618877 | 2.109142 | -0.703949 |
| C | 5.337989 | -1.130858 | -1.953095 |
| H | 3.965463 | -2.190385 | -0.681282 |
| C | 4.194840 | -2.777187 | 3.138663 |
| H | 4.734357 | -1.081553 | 1.934677 |
| C | 1.885136 | -3.481413 | 3.011021 |
| H | 0.612656 | -2.334829 | 1.702339 |
| C | -3.873412 | -2.013969 | -0.092377 |
| O | -2.005746 | -1.491089 | 1.298896 |
| H | -0.049553 | -0.502557 | -4.500381 |
| H | -0.863260 | -0.138953 | -2.965732 |
| H | 0.868485 | 0.226577 | -3.174266 |
| H | 2.013150 | -3.372647 | -3.229828 |
| H | 1.599437 | -2.418678 | -4.672533 |
| H | 2.568540 | -1.692930 | -3.376655 |
| H | -0.880746 | -2.904953 | -4.329154 |
| H | -1.636689 | -2.584688 | -2.747866 |
| H | -0.452404 | -3.898598 | -2.917635 |
| C | -0.597684 | 1.955551 | 2.619685 |
| H | 0.140467 | -0.061404 | 2.824722 |
| H | 0.144081 | 0.867904 | 4.306953 |
| C | 5.765792 | 0.113381 | -2.420929 |
| H | 5.464418 | 2.247382 | -2.323347 |
| H | 5.816735 | -2.040503 | -2.305531 |
| C | 3.170876 | -3.646031 | 3.526695 |
| H | 5.198433 | -2.901710 | 3.536866 |
| H | 1.086718 | -4.156707 | 3.305315 |
| C | -4.512715 | -1.875771 | -1.331633 |

| | | | |
|---|---|---|---|
| C | -4.530032 | -2.687837 | 0.946016 |
| P | -0.808286 | 1.832866 | 0.755247 |
| H | -1.595834 | 1.780412 | 3.032974 |
| H | -0.317109 | 2.977080 | 2.902872 |
| H | 6.579829 | 0.177409 | -3.137802 |
| H | 3.378176 | -4.450083 | 4.228087 |
| C | -5.788480 | -2.404837 | -1.528896 |
| H | -4.000918 | -1.346074 | -2.127744 |
| C | -5.803574 | -3.220300 | 0.747954 |
| H | -4.020194 | -2.784897 | 1.898934 |
| C | -2.602934 | 2.216119 | 0.536640 |
| C | 0.010753 | 3.348474 | 0.076996 |
| C | -6.435948 | -3.079423 | -0.490552 |
| H | -6.278643 | -2.291405 | -2.492772 |
| H | -6.303503 | -3.746715 | 1.557426 |
| C | -3.056378 | 3.222369 | -0.329963 |
| C | -3.551005 | 1.429126 | 1.213667 |
| C | 0.701457 | 3.242756 | -1.141064 |
| C | -0.051544 | 4.604091 | 0.705234 |
| H | -7.429015 | -3.494160 | -0.645982 |
| C | -4.423881 | 3.451120 | -0.497446 |
| H | -2.346942 | 3.836932 | -0.874131 |
| C | -4.915427 | 1.663277 | 1.045089 |
| H | -3.226723 | 0.603798 | 1.840598 |
| C | 1.322790 | 4.357384 | -1.711161 |
| H | 0.749531 | 2.278976 | -1.641767 |
| C | 0.573603 | 5.716114 | 0.140232 |
| H | -0.603406 | 4.723606 | 1.633304 |
| C | -5.357126 | 2.677472 | 0.192909 |
| H | -4.756372 | 4.238438 | -1.169190 |

| | | | |
|---|---|---|---|
| H | -5.632680 | 1.038034 | 1.569485 |
| C | 1.264258 | 5.594754 | -1.068521 |
| H | 1.849967 | 4.256023 | -2.656090 |
| H | 0.516571 | 6.679057 | 0.640931 |
| H | -6.420926 | 2.855675 | 0.060348 |
| H | 1.749136 | 6.462290 | -1.508130 |

**16**

| | | | |
|---|---|---|---|
| Pd | 2.921105 | -0.484579 | -0.437493 |
| C | 4.748846 | 0.297141 | -1.153026 |
| C | 1.246472 | -1.833272 | 1.624244 |
| O | 1.261504 | -1.724725 | 0.337425 |
| O | 2.003876 | -1.227553 | 2.406251 |
| Si | 3.150300 | -3.651405 | -1.488067 |
| C | 4.591085 | -4.213041 | -2.581887 |
| C | 1.544215 | -3.550538 | -2.465229 |
| C | 3.025302 | -4.740465 | 0.041795 |
| H | 4.425873 | -5.242952 | -2.924139 |
| H | 4.701127 | -3.584103 | -3.472731 |
| H | 5.542656 | -4.195406 | -2.037712 |
| H | 1.668212 | -2.962148 | -3.381956 |
| H | 1.191318 | -4.549189 | -2.751504 |
| H | 0.779803 | -3.070780 | -1.847009 |
| H | 3.933515 | -4.676558 | 0.652118 |
| H | 2.175596 | -4.449055 | 0.665341 |
| H | 2.892129 | -5.790530 | -0.248728 |
| H | 3.524091 | -2.241266 | -1.043137 |
| P | 1.885868 | 1.528717 | 0.093627 |
| C | 0.205433 | 1.270499 | 0.864020 |
| C | 1.482644 | 2.676161 | -1.292425 |

| | | | |
|---|---|---|---|
| C | 2.736360 | 2.513097 | 1.399999 |
| C | -0.851139 | 0.665547 | -0.070450 |
| H | -0.121021 | 2.251276 | 1.234516 |
| H | 0.369394 | 0.630853 | 1.735855 |
| C | 1.719451 | 2.268615 | -2.612749 |
| C | 0.833946 | 3.904089 | -1.070994 |
| C | 2.960707 | 1.869203 | 2.631266 |
| C | 3.181100 | 3.832059 | 1.225002 |
| C | -2.197887 | 0.470659 | 0.646472 |
| H | -0.487715 | -0.299678 | -0.436810 |
| H | -0.997171 | 1.319184 | -0.940370 |
| C | 1.338874 | 3.074805 | -3.687973 |
| H | 2.195977 | 1.309248 | -2.790821 |
| C | 0.456972 | 4.710607 | -2.145021 |
| H | 0.619684 | 4.234701 | -0.058842 |
| C | 3.593946 | 2.552470 | 3.669976 |
| H | 2.657479 | 0.833016 | 2.764755 |
| C | 3.818287 | 4.506115 | 2.269410 |
| H | 3.044340 | 4.336275 | 0.274280 |
| C | -3.259083 | -0.146302 | -0.277260 |
| H | -2.554895 | 1.437617 | 1.026246 |
| H | -2.054602 | -0.178248 | 1.520155 |
| C | 0.711666 | 4.299147 | -3.455783 |
| H | 1.531007 | 2.743018 | -4.704689 |
| H | -0.041004 | 5.658079 | -1.957508 |
| C | 4.021430 | 3.870891 | 3.494969 |
| H | 3.759165 | 2.046465 | 4.617437 |
| H | 4.158476 | 5.527450 | 2.119388 |
| P | -4.909344 | -0.376403 | 0.586893 |
| H | -2.922724 | -1.141554 | -0.595847 |

| | | | |
|---|---|---|---|
| H | -3.378532 | 0.466544 | -1.180881 |
| H | 0.415750 | 4.928265 | -4.290905 |
| H | 4.517051 | 4.397248 | 4.306468 |
| C | -5.982251 | -1.022864 | -0.778904 |
| C | -5.481512 | 1.388573 | 0.692619 |
| C | -7.347327 | -1.210251 | -0.492015 |
| C | -5.514761 | -1.407980 | -2.044947 |
| C | -5.880336 | 2.146612 | -0.420951 |
| C | -5.496559 | 1.997446 | 1.957098 |
| C | -8.216353 | -1.743636 | -1.441466 |
| H | -7.731759 | -0.932463 | 0.486957 |
| C | -6.383583 | -1.954625 | -2.994620 |
| H | -4.468041 | -1.284696 | -2.304295 |
| C | -6.276493 | 3.475893 | -0.273268 |
| H | -5.891850 | 1.689684 | -1.406997 |
| C | -5.886736 | 3.331093 | 2.106484 |
| H | -5.205170 | 1.419381 | 2.830805 |
| C | -7.735935 | -2.120959 | -2.698877 |
| H | -9.268020 | -1.872362 | -1.198280 |
| H | -5.998616 | -2.246566 | -3.968630 |
| C | -6.278483 | 4.072149 | 0.991189 |
| H | -6.586251 | 4.047574 | -1.144720 |
| H | -5.891603 | 3.786012 | 3.093751 |
| H | -8.410471 | -2.544672 | -3.438191 |
| H | -6.589744 | 5.107373 | 1.105227 |
| C | 0.197756 | -2.765316 | 2.196879 |
| C | 0.149756 | -2.968925 | 3.582901 |
| C | -0.732684 | -3.420891 | 1.378293 |
| C | -0.808726 | -3.813707 | 4.140681 |
| H | 0.875716 | -2.451586 | 4.201307 |

| | | | |
|---|---|---|---|
| C | -1.694868 | -4.264028 | 1.936080 |
| H | -0.692380 | -3.259687 | 0.306313 |
| C | -1.734142 | -4.462666 | 3.318321 |
| H | -0.836866 | -3.966200 | 5.216764 |
| H | -2.415115 | -4.764818 | 1.293968 |
| H | -2.484174 | -5.118708 | 3.752939 |
| C | 5.016679 | 1.797339 | -1.062335 |
| H | 4.969665 | 2.165087 | -0.034593 |
| H | 6.033108 | 2.008340 | -1.434808 |
| H | 4.325697 | 2.384849 | -1.675122 |
| C | 5.725403 | -0.445172 | -0.230449 |
| H | 5.583632 | -0.164665 | 0.819709 |
| H | 5.642013 | -1.534404 | -0.307103 |
| H | 6.762373 | -0.181233 | -0.501067 |
| C | 4.892465 | -0.155133 | -2.610798 |
| H | 4.170296 | 0.341069 | -3.268725 |
| H | 5.897590 | 0.111285 | -2.981013 |
| H | 4.779427 | -1.235176 | -2.734354 |

**TS17**

| | | | |
|---|---|---|---|
| Pd | 2.962124 | -0.761333 | -0.495858 |
| C | 5.106576 | -0.072322 | -1.176918 |
| C | 1.368698 | -1.773603 | 1.687562 |
| O | 1.256016 | -1.782138 | 0.397401 |
| O | 2.282553 | -1.206327 | 2.313576 |
| Si | 3.179541 | -3.041777 | -1.479502 |
| C | 4.495543 | -3.539530 | -2.768984 |
| C | 1.512995 | -3.178472 | -2.362776 |
| C | 3.370827 | -4.230847 | -0.023048 |
| H | 4.300997 | -4.582615 | -3.055271 |

| | | | |
|---|---|---|---|
| H | 4.436821 | -2.936153 | -3.681690 |
| H | 5.523253 | -3.493824 | -2.392743 |
| H | 1.481167 | -2.539351 | -3.252711 |
| H | 1.353967 | -4.215401 | -2.689715 |
| H | 0.701239 | -2.889521 | -1.692180 |
| H | 4.307297 | -4.055247 | 0.517761 |
| H | 2.544646 | -4.128112 | 0.683615 |
| H | 3.386912 | -5.264589 | -0.394541 |
| H | 4.137545 | -1.346586 | -1.417817 |
| P | 1.775634 | 1.442222 | 0.137000 |
| C | 0.066882 | 1.240121 | 0.875057 |
| C | 1.425181 | 2.536913 | -1.311646 |
| C | 2.572026 | 2.531589 | 1.402582 |
| C | -0.956013 | 0.580813 | -0.060652 |
| H | -0.282722 | 2.230246 | 1.194030 |
| H | 0.197105 | 0.642014 | 1.782954 |
| C | 1.839321 | 2.119190 | -2.585061 |
| C | 0.695655 | 3.734409 | -1.203537 |
| C | 2.798096 | 1.974720 | 2.675567 |
| C | 3.002226 | 3.843077 | 1.150643 |
| C | -2.324465 | 0.402508 | 0.617992 |
| H | -0.572016 | -0.395352 | -0.374688 |
| H | -1.078162 | 1.192485 | -0.964224 |
| C | 1.548759 | 2.880797 | -3.720032 |
| H | 2.380420 | 1.181185 | -2.681465 |
| C | 0.405998 | 4.496613 | -2.335311 |
| H | 0.353194 | 4.077280 | -0.231152 |
| C | 3.409654 | 2.731802 | 3.675478 |
| H | 2.520224 | 0.942281 | 2.872161 |
| C | 3.620984 | 4.592414 | 2.154450 |

| | | | |
|---|---|---|---|
| H | 2.860445 | 4.287223 | 0.170895 |
| C | -3.354606 | -0.246083 | -0.319280 |
| H | -2.696796 | 1.379079 | 0.955932 |
| H | -2.206674 | -0.218327 | 1.515632 |
| C | 0.834489 | 4.072485 | -3.596321 |
| H | 1.876977 | 2.539497 | -4.698270 |
| H | -0.157983 | 5.420058 | -2.233495 |
| C | 3.820469 | 4.042509 | 3.420925 |
| H | 3.573617 | 2.289417 | 4.654680 |
| H | 3.947014 | 5.607434 | 1.941689 |
| P | -5.020525 | -0.489337 | 0.510313 |
| H | -2.996266 | -1.241031 | -0.614673 |
| H | -3.462186 | 0.349887 | -1.235540 |
| H | 0.606361 | 4.666774 | -4.477108 |
| H | 4.300404 | 4.627205 | 4.201382 |
| C | -6.056104 | -1.160043 | -0.872397 |
| C | -5.617358 | 1.268468 | 0.590960 |
| C | -7.426969 | -1.351442 | -0.617404 |
| C | -5.554335 | -1.561085 | -2.120240 |
| C | -6.000381 | 2.013444 | -0.536881 |
| C | -5.668505 | 1.885933 | 1.850293 |
| C | -8.268444 | -1.904275 | -1.580421 |
| H | -7.837963 | -1.060910 | 0.346973 |
| C | -6.395611 | -2.127075 | -3.083300 |
| H | -4.502128 | -1.435073 | -2.354914 |
| C | -6.416446 | 3.338567 | -0.407859 |
| H | -5.983655 | 1.549535 | -1.519568 |
| C | -6.078808 | 3.215454 | 1.981092 |
| H | -5.389109 | 1.317978 | 2.734473 |
| C | -7.754046 | -2.297341 | -2.819449 |

| | | | |
|---|---|---|---|
| H | -9.325245 | -2.035604 | -1.362125 |
| H | -5.984304 | -2.430954 | -4.042769 |
| C | -6.454564 | 3.943565 | 0.851852 |
| H | -6.713327 | 3.900201 | -1.290242 |
| H | -6.111448 | 3.677321 | 2.964590 |
| H | -8.407125 | -2.736066 | -3.569195 |
| H | -6.781105 | 4.975577 | 0.951261 |
| C | 0.282401 | -2.503161 | 2.443369 |
| C | 0.315859 | -2.509039 | 3.844532 |
| C | -0.756603 | -3.170762 | 1.779862 |
| C | -0.675061 | -3.167575 | 4.571067 |
| H | 1.130337 | -1.992029 | 4.340954 |
| C | -1.749372 | -3.828522 | 2.506976 |
| H | -0.774215 | -3.168063 | 0.695338 |
| C | -1.710745 | -3.827405 | 3.903677 |
| H | -0.641399 | -3.167011 | 5.657657 |
| H | -2.553762 | -4.340803 | 1.985515 |
| H | -2.485304 | -4.338806 | 4.469545 |
| C | 5.102154 | 1.332104 | -0.570345 |
| H | 4.873469 | 1.329187 | 0.497032 |
| H | 6.115889 | 1.752666 | -0.689603 |
| H | 4.410419 | 2.006306 | -1.080522 |
| C | 6.130420 | -0.932780 | -0.426844 |
| H | 5.851402 | -1.052776 | 0.625072 |
| H | 6.248490 | -1.928971 | -0.864588 |
| H | 7.112891 | -0.437377 | -0.459117 |
| C | 5.409652 | 0.023624 | -2.675286 |
| H | 4.637172 | 0.590493 | -3.204834 |
| H | 6.363002 | 0.556378 | -2.815581 |
| H | 5.505162 | -0.955076 | -3.153486 |

**18**

| | | | |
|---|---|---|---|
| C | 0.148070 | -0.605185 | -0.000231 |
| O | -0.726030 | 0.421325 | -0.000261 |
| O | -0.202580 | -1.773703 | -0.000261 |
| C | 1.577236 | -0.178338 | -0.000132 |
| C | 1.951909 | 1.172496 | -0.000087 |
| C | 2.564060 | -1.174592 | -0.000083 |
| C | 3.302193 | 1.520336 | 0.000009 |
| H | 1.183745 | 1.937639 | -0.000133 |
| C | 3.911745 | -0.823627 | 0.000012 |
| H | 2.252024 | -2.213733 | -0.000122 |
| C | 4.282289 | 0.524476 | 0.000061 |
| H | 3.590549 | 2.567964 | 0.000043 |
| H | 4.673752 | -1.598149 | 0.000048 |
| H | 5.334108 | 0.798409 | 0.000136 |
| Si | -2.439805 | 0.175599 | 0.000097 |
| C | -2.946164 | -0.734530 | -1.565239 |
| H | -2.505380 | -1.735372 | -1.593825 |
| H | -4.037455 | -0.837095 | -1.617715 |
| H | -2.620846 | -0.192250 | -2.460967 |
| C | -2.945009 | -0.733767 | 1.566276 |
| H | -2.619874 | -0.190544 | 2.461497 |
| H | -4.036140 | -0.837564 | 1.619306 |
| H | -2.503084 | -1.734106 | 1.595302 |
| C | -3.087411 | 1.942046 | -0.000432 |
| H | -2.747610 | 2.490683 | -0.886441 |
| H | -4.184352 | 1.957276 | 0.000062 |
| H | -2.746797 | 2.491594 | 0.884697 |

**CO**

| | | | |
|---|---|---|---|
| C | 0.000000 | 0.000000 | -0.650130 |
| O | 0.000000 | 0.000000 | 0.487597 |

**TMSH**

| | | | |
|---|---|---|---|
| Si | 0.000034 | -0.000312 | 0.375634 |
| H | 0.000028 | -0.000500 | 1.871266 |
| C | -1.520626 | -0.957462 | -0.222232 |
| H | -2.447957 | -0.494257 | 0.135048 |
| H | -1.566879 | -0.989260 | -1.317798 |
| H | -1.507190 | -1.992590 | 0.138983 |
| C | 1.589945 | -0.837400 | -0.222239 |
| H | 1.638530 | -0.865362 | -1.317799 |
| H | 2.478857 | -0.304198 | 0.135116 |
| H | 1.656324 | -1.870541 | 0.138805 |
| C | -0.069357 | 1.795238 | -0.222014 |
| H | -0.974954 | 2.299391 | 0.135640 |
| H | 0.792906 | 2.368694 | 0.138300 |
| H | -0.069908 | 1.850735 | -1.317517 |

**PhH**

| | | | |
|---|---|---|---|
| C | 1.064021 | -0.905077 | -0.000005 |
| C | -0.251950 | -1.373873 | -0.000069 |
| C | -1.315873 | -0.468904 | 0.000056 |
| C | -1.063945 | 0.905165 | -0.000008 |
| C | 0.251834 | 1.373892 | -0.000062 |
| C | 1.315914 | 0.468799 | 0.000058 |
| H | 1.891597 | -1.609336 | 0.000066 |
| H | -0.447753 | -2.442784 | -0.000078 |
| H | -2.339531 | -0.833555 | 0.000155 |

| | | | |
|---|---|---|---|
| H | -1.891692 | 1.609211 | -0.000007 |
| H | 0.447884 | 2.442751 | -0.000018 |
| H | 2.339489 | 0.833705 | 0.000064 |

**PhCOOPiv**

| | | | |
|---|---|---|---|
| C | 4.256923 | -0.489165 | 0.162949 |
| C | 4.396378 | 0.881550 | -0.075756 |
| C | 2.988280 | -1.058210 | 0.225931 |
| H | 5.137117 | -1.111171 | 0.298626 |
| C | 3.264798 | 1.681822 | -0.251995 |
| H | 5.387109 | 1.325421 | -0.125346 |
| C | 1.849016 | -0.257629 | 0.050783 |
| H | 2.855494 | -2.119627 | 0.407424 |
| C | 1.991976 | 1.116773 | -0.188740 |
| H | 3.374336 | 2.746240 | -0.439565 |
| C | 0.524393 | -0.925257 | 0.121353 |
| H | 1.110456 | 1.732378 | -0.326767 |
| O | -0.525764 | -0.011869 | 0.057979 |
| O | 0.345418 | -2.106628 | 0.275441 |
| C | -1.758000 | -0.391682 | -0.484722 |
| C | -2.902293 | 0.401063 | 0.146006 |
| O | -1.847107 | -1.210378 | -1.357215 |
| C | -2.631591 | 1.915013 | -0.005739 |
| C | -4.209725 | 0.020829 | -0.567016 |
| C | -2.981276 | 0.030923 | 1.645327 |
| H | -2.545334 | 2.200507 | -1.060564 |
| H | -3.465492 | 2.479181 | 0.427224 |
| H | -1.714030 | 2.212708 | 0.509507 |
| H | -4.412950 | -1.050253 | -0.476676 |
| H | -5.046594 | 0.572320 | -0.124170 |

| | | | |
|---|---|---|---|
| H | -4.163852 | 0.260282 | -1.633972 |
| H | -3.156833 | -1.042174 | 1.780473 |
| H | -3.812813 | 0.571409 | 2.111829 |
| H | -2.060273 | 0.297596 | 2.172049 |

***t*BuH**

| | | | |
|---|---|---|---|
| C | 0.000008 | -0.000007 | -0.373044 |
| H | -0.000011 | -0.000005 | -1.473623 |
| C | -0.849360 | -1.190352 | 0.095874 |
| H | -1.882104 | -1.111300 | -0.264873 |
| H | -0.439249 | -2.141208 | -0.265567 |
| H | -0.882844 | -1.238008 | 1.192520 |
| C | 1.455579 | -0.140372 | 0.095869 |
| H | 1.903628 | -1.074094 | -0.265183 |
| H | 2.073853 | 0.690443 | -0.265286 |
| H | 1.513615 | -0.145915 | 1.192519 |
| C | -0.606218 | 1.330727 | 0.095871 |
| H | -0.021866 | 2.185651 | -0.265493 |
| H | -1.635004 | 1.450572 | -0.264951 |
| H | -0.630076 | 1.383891 | 1.192513 |

**TS-S1**

| | | | |
|---|---|---|---|
| Pd | 0.087343 | -0.234511 | -0.105274 |
| P | 2.344849 | -0.242682 | 0.467085 |
| C | 2.704725 | 1.302722 | 1.479173 |
| C | 3.651432 | -0.094954 | -0.838785 |
| C | 3.000893 | -1.562573 | 1.582745 |
| C | 2.142604 | 1.317434 | 2.913863 |
| H | 2.311922 | 2.148102 | 0.904646 |
| H | 3.792154 | 1.438321 | 1.531164 |

| | | | |
|---|---|---|---|
| C | 3.804977 | 1.115078 | -1.538400 |
| C | 4.452407 | -1.186418 | -1.213048 |
| C | 4.310998 | -1.525574 | 2.094627 |
| C | 2.144974 | -2.600776 | 1.978274 |
| C | 0.628790 | 1.133287 | 3.118319 |
| H | 2.654314 | 0.539347 | 3.493058 |
| H | 2.440493 | 2.275229 | 3.365945 |
| C | 4.740213 | 1.232442 | -2.567090 |
| H | 3.191388 | 1.974961 | -1.285311 |
| C | 5.384441 | -1.068404 | -2.246313 |
| H | 4.354241 | -2.132958 | -0.690861 |
| C | 4.753082 | -2.505096 | 2.982509 |
| H | 4.992529 | -0.734530 | 1.792457 |
| C | 2.592654 | -3.582211 | 2.868779 |
| H | 1.136617 | -2.650952 | 1.579937 |
| C | -0.289559 | 2.219223 | 2.524085 |
| H | 0.308269 | 0.150789 | 2.750988 |
| H | 0.459341 | 1.121621 | 4.203982 |
| C | 5.534562 | 0.141120 | -2.925908 |
| H | 4.848199 | 2.180276 | -3.088606 |
| H | 5.995647 | -1.925824 | -2.516330 |
| C | 3.891949 | -3.535998 | 3.372544 |
| H | 5.767853 | -2.465105 | 3.370473 |
| H | 1.919563 | -4.382993 | 3.162939 |
| P | -0.700574 | 2.010081 | 0.692966 |
| H | -1.253168 | 2.185520 | 3.042982 |
| H | 0.126704 | 3.217404 | 2.705927 |
| H | 6.261936 | 0.232877 | -3.728066 |
| H | 4.237402 | -4.299549 | 4.064938 |
| C | -2.498846 | 2.461700 | 0.667733 |

| | | | |
|---|---|---|---|
| C | 0.084177 | 3.467031 | -0.139936 |
| C | -3.021197 | 3.516997 | -0.095496 |
| C | -3.392740 | 1.649365 | 1.390296 |
| C | 0.605196 | 3.281490 | -1.430743 |
| C | 0.164275 | 4.747386 | 0.434023 |
| C | -4.395702 | 3.767298 | -0.117764 |
| H | -2.357824 | 4.153186 | -0.672171 |
| C | -4.762995 | 1.906664 | 1.371400 |
| H | -3.021211 | 0.796831 | 1.952777 |
| C | 1.192893 | 4.340840 | -2.127439 |
| H | 0.551877 | 2.295416 | -1.885590 |
| C | 0.759082 | 5.804398 | -0.256454 |
| H | -0.250856 | 4.930971 | 1.421140 |
| C | -5.270576 | 2.968545 | 0.618431 |
| H | -4.779438 | 4.593057 | -0.711816 |
| H | -5.435690 | 1.266395 | 1.935917 |
| C | 1.275768 | 5.603565 | -1.539049 |
| H | 1.587262 | 4.176085 | -3.126781 |
| H | 0.814161 | 6.786764 | 0.205485 |
| H | -6.339282 | 3.164912 | 0.599762 |
| H | 1.737387 | 6.427938 | -2.076053 |
| C | -0.398571 | -2.326434 | -0.912464 |
| C | -0.066465 | -2.440825 | -2.422322 |
| O | -0.234314 | -3.221270 | -0.112894 |
| C | -0.212556 | -1.118616 | -3.189623 |
| C | 1.377548 | -2.960620 | -2.537316 |
| C | -1.028141 | -3.505153 | -3.004838 |
| H | 0.454325 | -0.352826 | -2.775549 |
| H | 0.059116 | -1.273010 | -4.241407 |
| H | -1.237435 | -0.739360 | -3.152588 |

| | | | |
|---|---|---|---|
| H | 1.506221 | -3.886358 | -1.969176 |
| H | 1.612513 | -3.160654 | -3.589464 |
| H | 2.094006 | -2.223445 | -2.166200 |
| H | -0.930299 | -4.451597 | -2.463853 |
| H | -0.785270 | -3.682101 | -4.059571 |
| H | -2.070710 | -3.180645 | -2.943334 |
| C | -2.568802 | -1.709601 | 0.254526 |
| C | -3.971922 | -2.097246 | -0.080085 |
| O | -1.776403 | -1.505523 | -0.831975 |
| O | -2.179230 | -1.561299 | 1.398193 |
| C | -4.462982 | -2.079171 | -1.393149 |
| C | -4.821438 | -2.474054 | 0.971299 |
| C | -5.785596 | -2.439178 | -1.649999 |
| H | -3.809919 | -1.770651 | -2.201208 |
| C | -6.138945 | -2.841702 | 0.710788 |
| H | -4.425385 | -2.477741 | 1.981373 |
| C | -6.623380 | -2.824409 | -0.600826 |
| H | -6.162620 | -2.418991 | -2.668899 |
| H | -6.789535 | -3.141661 | 1.527889 |
| H | -7.652489 | -3.109238 | -0.803900 |

**TS-S2**

| | | | |
|---|---|---|---|
| Pd | d -0.205799 | -0.037126 | 0.888548 |
| C | 0.657154 | 0.691084 | 3.271349 |
| P | -1.687278 | -1.237924 | -0.626196 |
| C | -0.382272 | 1.480930 | 4.047759 |
| C | 1.705905 | 1.590679 | 2.642550 |
| C | 1.303346 | -0.448298 | 4.042462 |
| C | -0.900089 | -0.788966 | 2.441858 |
| P | 0.103698 | 1.949428 | -0.766553 |

| | | | |
|---|---|---|---|
| C | -2.025672 | -0.455878 | -2.288974 |
| C | -1.202305 | -2.967006 | -1.019620 |
| C | -3.385226 | -1.421509 | 0.072275 |
| H | -1.144382 | 0.846041 | 4.508012 |
| H | -0.875545 | 2.230461 | 3.421178 |
| H | 0.132750 | 2.019538 | 4.861284 |
| H | 2.346093 | 1.966411 | 3.461729 |
| H | 1.271318 | 2.459680 | 2.146412 |
| H | 2.345042 | 1.061786 | 1.934503 |
| H | 1.879726 | -1.097043 | 3.376880 |
| H | 0.579512 | -1.054522 | 4.593584 |
| H | 1.993155 | -0.003994 | 4.779425 |
| O | -1.529093 | -1.400323 | 3.200629 |
| C | 0.614343 | 1.430446 | -2.510736 |
| C | 1.386126 | 3.240355 | -0.424574 |
| C | -1.436373 | 2.951781 | -1.006889 |
| C | -0.881148 | -0.565734 | -3.310394 |
| H | -2.268470 | 0.594953 | -2.098240 |
| H | -2.931586 | -0.914619 | -2.703480 |
| C | -1.957169 | -3.738615 | -1.922505 |
| C | -0.072429 | -3.527662 | -0.408354 |
| C | -3.691203 | -2.517071 | 0.896028 |
| C | -4.369933 | -0.442661 | -0.141152 |
| C | 0.500927 | -0.061627 | -2.872597 |
| H | 1.667928 | 1.715923 | -2.576817 |
| H | 0.076931 | 2.050516 | -3.239020 |
| C | 2.731443 | 2.828302 | -0.388432 |
| C | 1.080448 | 4.580238 | -0.144427 |
| C | -1.610644 | 3.860213 | -2.066033 |
| C | -2.472629 | 2.813052 | -0.069798 |

| | | | |
|---|---|---|---|
| H | -0.778121 | -1.615582 | -3.609587 |
| H | -1.198236 | -0.020275 | -4.211213 |
| C | -1.579632 | -5.047345 | -2.215193 |
| H | -2.846401 | -3.323438 | -2.390174 |
| C | 0.297688 | -4.843925 | -0.704981 |
| H | 0.528120 | -2.950259 | 0.292384 |
| C | -4.951809 | -2.634293 | 1.482034 |
| H | -2.945473 | -3.285086 | 1.076002 |
| C | -5.630939 | -0.564610 | 0.444893 |
| H | -4.160858 | 0.422848 | -0.761929 |
| H | 0.896919 | -0.653237 | -2.041237 |
| H | 1.181905 | -0.237362 | -3.716672 |
| C | 3.743305 | 3.746360 | -0.111223 |
| H | 2.971818 | 1.778777 | -0.537515 |
| C | 2.098827 | 5.494749 | 0.139454 |
| H | 0.050181 | 4.920519 | -0.153170 |
| C | -2.787732 | 4.599404 | -2.186714 |
| H | -0.818128 | 4.007020 | -2.794573 |
| C | -3.650556 | 3.556744 | -0.186270 |
| H | -2.354855 | 2.108225 | 0.749918 |
| C | -0.448544 | -5.601693 | -1.606130 |
| H | -2.167450 | -5.635603 | -2.915073 |
| H | 1.177950 | -5.264420 | -0.227169 |
| C | -5.926403 | -1.660244 | 1.257871 |
| H | -5.170438 | -3.490477 | 2.114414 |
| H | -6.383463 | 0.198129 | 0.261785 |
| C | 3.431582 | 5.083504 | 0.150273 |
| H | 4.777299 | 3.412229 | -0.088504 |
| H | 1.845128 | 6.530885 | 0.348735 |
| C | -3.811048 | 4.449281 | -1.246509 |

| | | | |
|---|---|---|---|
| H | -2.904515 | 5.296378 | -3.012439 |
| H | -4.439716 | 3.434406 | 0.550862 |
| H | -0.155064 | -6.622749 | -1.836055 |
| H | -6.908421 | -1.754049 | 1.713487 |
| H | 4.221984 | 5.796677 | 0.368910 |
| H | -4.726555 | 5.027134 | -1.341012 |
| C | 2.667271 | -1.377856 | 0.445577 |
| C | 4.069411 | -1.658440 | -0.072723 |
| O | 2.052809 | -0.384038 | -0.081990 |
| O | 2.210892 | -2.129415 | 1.335946 |
| C | 4.861097 | -2.623387 | 0.563908 |
| C | 4.588166 | -0.980833 | -1.183432 |
| C | 6.150006 | -2.897457 | 0.106844 |
| H | 4.440701 | -3.146708 | 1.416428 |
| C | 5.874232 | -1.258579 | -1.648560 |
| H | 3.965356 | -0.243962 | -1.679785 |
| C | 6.660086 | -2.215816 | -1.001722 |
| H | 6.757736 | -3.644455 | 0.612117 |
| H | 6.264468 | -0.729792 | -2.515186 |
| H | 7.663593 | -2.431155 | -1.361011 |

**S3**

| | | | |
|---|---|---|---|
| Pd | -0.182221 | 0.073526 | 0.981971 |
| C | 0.463815 | 1.224728 | 2.782795 |
| P | -0.001958 | 1.891697 | -0.656105 |
| C | 1.985762 | 1.186067 | 2.803924 |
| C | -0.082203 | 2.648782 | 2.714686 |
| C | -0.115971 | 0.567498 | 4.044520 |
| C | -0.053443 | -1.528364 | 2.107399 |
| C | 0.526379 | 1.302840 | -2.367631 |

| | | | |
|---|---|---|---|
| C | -1.594727 | 2.797735 | -0.927462 |
| C | 1.241108 | 3.251246 | -0.445831 |
| H | 2.423392 | 1.691781 | 1.940877 |
| H | 2.362792 | 0.159638 | 2.821491 |
| H | 2.350215 | 1.701098 | 3.710923 |
| H | -1.175763 | 2.670359 | 2.650571 |
| H | 0.192869 | 3.176469 | 3.644853 |
| H | 0.330901 | 3.230958 | 1.891782 |
| H | 0.345307 | -0.397061 | 4.272695 |
| H | 0.081269 | 1.226523 | 4.906989 |
| H | -1.201624 | 0.429488 | 3.986006 |
| O | -0.103484 | -2.507045 | 2.699018 |
| C | 0.288969 | -0.146554 | -2.815733 |
| H | 1.607796 | 1.450168 | -2.347739 |
| H | 0.118319 | 2.012912 | -3.097870 |
| C | -1.803537 | 3.663643 | -2.015754 |
| C | -2.640939 | 2.614037 | -0.010146 |
| C | 2.602917 | 2.897053 | -0.407487 |
| C | 0.885844 | 4.602153 | -0.314002 |
| C | -1.124097 | -0.550933 | -3.255165 |
| H | 0.670150 | -0.807688 | -2.032878 |
| H | 0.949303 | -0.307071 | -3.678937 |
| C | -3.023035 | 4.320660 | -2.181236 |
| H | -1.007838 | 3.841260 | -2.733527 |
| C | -3.861523 | 3.275074 | -0.172393 |
| H | -2.497895 | 1.938845 | 0.829458 |
| C | 3.579725 | 3.882732 | -0.261736 |
| H | 2.886283 | 1.848284 | -0.472900 |
| C | 1.870326 | 5.582434 | -0.166637 |
| H | -0.157314 | 4.899506 | -0.323632 |

| | | | |
|---|---|---|---|
| C | -2.220138 | -0.504822 | -2.177346 |
| H | -1.067993 | -1.572507 | -3.649583 |
| H | -1.449535 | 0.083335 | -4.092163 |
| C | -4.055425 | 4.128255 | -1.259120 |
| H | -3.166019 | 4.985123 | -3.029193 |
| H | -4.657435 | 3.119280 | 0.550844 |
| C | 3.218984 | 5.226961 | -0.143884 |
| H | 4.627145 | 3.594182 | -0.233852 |
| H | 1.577323 | 6.624636 | -0.069787 |
| P | -1.711308 | -1.298658 | -0.567547 |
| H | -2.486790 | 0.531283 | -1.947953 |
| H | -3.128896 | -0.994235 | -2.547978 |
| H | -5.004126 | 4.642108 | -1.388621 |
| H | 3.983500 | 5.990894 | -0.029651 |
| C | -3.319858 | -1.517886 | 0.312503 |
| C | -1.270783 | -3.020027 | -1.061428 |
| C | -4.400726 | -0.643908 | 0.112762 |
| C | -3.452762 | -2.543536 | 1.265076 |
| C | 0.001584 | -3.530401 | -0.765660 |
| C | -2.194845 | -3.826039 | -1.751480 |
| C | -5.583216 | -0.796188 | 0.840963 |
| H | -4.331104 | 0.162412 | -0.609991 |
| C | -4.633880 | -2.692125 | 1.990976 |
| H | -2.635253 | -3.238219 | 1.433231 |
| C | 0.341063 | -4.828627 | -1.161665 |
| H | 0.729636 | -2.932791 | -0.220386 |
| C | -1.848290 | -5.116786 | -2.145370 |
| H | -3.191152 | -3.450205 | -1.970536 |
| C | -5.704031 | -1.818645 | 1.782112 |
| H | -6.411600 | -0.114337 | 0.666405 |

| | | | |
|---|---|---|---|
| H | -4.718137 | -3.494861 | 2.718561 |
| C | -0.576536 | -5.619686 | -1.851147 |
| H | 1.328653 | -5.213632 | -0.923590 |
| H | -2.569635 | -5.731530 | -2.677525 |
| H | -6.624588 | -1.936558 | 2.347180 |
| H | -0.307715 | -6.627478 | -2.156987 |
| C | 2.806755 | -1.212058 | 0.234601 |
| C | 4.265899 | -1.557219 | -0.034100 |
| O | 2.318580 | -0.230810 | -0.399515 |
| O | 2.198204 | -1.944809 | 1.066913 |
| C | 4.984499 | -0.895339 | -1.037603 |
| C | 4.907383 | -2.548889 | 0.719404 |
| C | 6.320372 | -1.214626 | -1.282810 |
| H | 4.475607 | -0.135423 | -1.621830 |
| C | 6.244857 | -2.865996 | 0.480928 |
| H | 4.336198 | -3.057392 | 1.489016 |
| C | 6.955170 | -2.199642 | -0.521526 |
| H | 6.867840 | -0.696434 | -2.066932 |
| H | 6.734795 | -3.633532 | 1.075627 |
| H | 7.997179 | -2.447660 | -0.709431 |

**S4**

| | | | |
|---|---|---|---|
| Pd | 0.108099 | -0.407215 | -0.612139 |
| C | 0.513088 | -1.755257 | -2.260632 |
| P | 2.253374 | -0.412414 | 0.332485 |
| C | -0.252301 | -3.062043 | -2.063871 |
| C | 1.977781 | -2.060354 | -2.545422 |
| C | -0.076431 | -0.924332 | -3.403367 |
| C | 2.434057 | 0.742702 | 1.805791 |
| C | 3.765880 | 0.090189 | -0.615835 |

| | | | |
|---|---|---|---|
| C | 2.666119 | -2.033899 | 1.112059 |
| H | 0.143083 | -3.646658 | -1.226040 |
| H | -0.138499 | -3.685170 | -2.968532 |
| H | -1.317906 | -2.894106 | -1.907926 |
| H | 2.048502 | -2.678483 | -3.457298 |
| H | 2.568232 | -1.158253 | -2.724844 |
| H | 2.445362 | -2.635018 | -1.738818 |
| H | 0.488006 | 0.002588 | -3.567147 |
| H | -0.021417 | -1.503621 | -4.342389 |
| H | -1.125240 | -0.675622 | -3.226151 |
| C | 1.739546 | 0.322119 | 3.113501 |
| H | 2.089839 | 1.730231 | 1.481505 |
| H | 3.509327 | 0.837665 | 1.998270 |
| C | 3.935731 | 1.452817 | -0.916634 |
| C | 4.733133 | -0.818120 | -1.073337 |
| C | 3.920970 | -2.292288 | 1.691618 |
| C | 1.651998 | -2.996324 | 1.233611 |
| C | 0.216873 | 0.126032 | 3.093924 |
| H | 2.201413 | -0.604338 | 3.474165 |
| H | 1.985483 | 1.092844 | 3.858987 |
| C | 5.043516 | 1.892962 | -1.641351 |
| H | 3.200255 | 2.181618 | -0.590727 |
| C | 5.841577 | -0.375895 | -1.798593 |
| H | 4.625246 | -1.877473 | -0.869091 |
| C | 4.161741 | -3.498262 | 2.350146 |
| H | 4.715571 | -1.554427 | 1.627121 |
| C | 1.894844 | -4.201145 | 1.898321 |
| H | 0.667002 | -2.789943 | 0.823664 |
| C | -0.604267 | 1.355452 | 2.666020 |
| H | -0.068482 | -0.723789 | 2.464998 |

| | | | |
|---|---|---|---|
| H | -0.083966 | -0.139681 | 4.116365 |
| C | 6.002509 | 0.980095 | -2.084214 |
| H | 5.154971 | 2.951971 | -1.859015 |
| H | 6.577998 | -1.097716 | -2.141749 |
| C | 3.149963 | -4.457169 | 2.450685 |
| H | 5.138515 | -3.687350 | 2.787691 |
| H | 1.098282 | -4.934959 | 1.983892 |
| P | -0.765377 | 1.558522 | 0.804298 |
| H | -1.624186 | 1.253032 | 3.045626 |
| H | -0.195796 | 2.271015 | 3.110285 |
| H | 6.865326 | 1.322513 | -2.649001 |
| H | 3.339706 | -5.395986 | 2.964195 |
| C | -2.537296 | 2.061192 | 0.599705 |
| C | 0.093022 | 3.154091 | 0.408560 |
| C | -2.899816 | 3.112947 | -0.257329 |
| C | -3.555061 | 1.353870 | 1.261278 |
| C | 0.719180 | 3.272508 | -0.842852 |
| C | 0.093285 | 4.269093 | 1.263631 |
| C | -4.240462 | 3.460034 | -0.431366 |
| H | -2.137589 | 3.673629 | -0.788214 |
| C | -4.893758 | 1.706224 | 1.087308 |
| H | -3.310574 | 0.496314 | 1.879085 |
| C | 1.332545 | 4.468259 | -1.228588 |
| H | 0.720896 | 2.419282 | -1.517265 |
| C | 0.716777 | 5.458722 | 0.885836 |
| H | -0.408306 | 4.220716 | 2.226011 |
| C | -5.242184 | 2.762743 | 0.244837 |
| H | -4.498574 | 4.280757 | -1.095775 |
| H | -5.664828 | 1.139340 | 1.601682 |
| C | 1.337811 | 5.561777 | -0.361753 |

| | | | |
|---|---|---|---|
| H | 1.805500 | 4.540961 | -2.204476 |
| H | 0.709245 | 6.309324 | 1.562388 |
| H | -6.285795 | 3.033706 | 0.108976 |
| H | 1.817448 | 6.491198 | -0.656842 |
| C | -2.560936 | -1.356514 | -0.070146 |
| C | -3.968955 | -1.836389 | -0.343479 |
| O | -1.958891 | -0.808242 | -1.071882 |
| O | -2.072295 | -1.512691 | 1.067889 |
| C | -4.572133 | -1.662251 | -1.596059 |
| C | -4.682517 | -2.479125 | 0.676979 |
| C | -5.870145 | -2.120836 | -1.822525 |
| H | -4.012323 | -1.164156 | -2.380077 |
| C | -5.979579 | -2.938363 | 0.450995 |
| H | -4.196872 | -2.610639 | 1.638442 |
| C | -6.576697 | -2.759649 | -0.800141 |
| H | -6.331594 | -1.980542 | -2.797000 |
| H | -6.524986 | -3.438512 | 1.247765 |
| H | -7.587746 | -3.118193 | -0.977976 |

**S5**

| | | | |
|---|---|---|---|
| Pd | -0.222108 | -0.752599 | -0.015826 |
| P | 0.704940 | 1.653677 | 0.655789 |
| P | -2.375340 | -0.208209 | 0.639637 |
| C | -0.293623 | 2.515248 | 1.982606 |
| C | 0.725013 | 2.911888 | -0.700458 |
| C | 2.399111 | 1.759388 | 1.386721 |
| C | -2.374824 | 0.218795 | 2.487396 |
| C | -3.066480 | 1.272082 | -0.216768 |
| C | -3.804167 | -1.391532 | 0.625951 |
| C | -0.410786 | 1.726015 | 3.296232 |

| | | | |
|---|---|---|---|
| H | -1.288823 | 2.703499 | 1.561374 |
| H | 0.152706 | 3.492544 | 2.201151 |
| C | 1.292612 | 2.536710 | -1.932150 |
| C | 0.182942 | 4.201294 | -0.570811 |
| C | 3.104846 | 2.972282 | 1.448041 |
| C | 2.974574 | 0.607621 | 1.944006 |
| C | -1.005742 | 0.312464 | 3.193693 |
| H | -2.963267 | -0.575127 | 2.954091 |
| H | -2.948182 | 1.142835 | 2.627240 |
| C | -4.139701 | 2.017958 | 0.302866 |
| C | -2.514567 | 1.649632 | -1.449558 |
| C | -3.724268 | -2.544478 | 1.426163 |
| C | -4.945410 | -1.202444 | -0.164732 |
| H | 0.579126 | 1.652801 | 3.763224 |
| H | -1.030075 | 2.319334 | 3.984181 |
| C | 1.324380 | 3.439311 | -2.996219 |
| H | 1.706598 | 1.538655 | -2.047546 |
| C | 0.212665 | 5.097764 | -1.641981 |
| H | -0.272156 | 4.522098 | 0.360008 |
| C | 4.354794 | 3.032517 | 2.063186 |
| H | 2.682703 | 3.869205 | 1.003007 |
| C | 4.226538 | 0.672069 | 2.561690 |
| H | 2.461397 | -0.344882 | 1.857666 |
| H | -0.296081 | -0.365419 | 2.703606 |
| H | -1.126918 | -0.066609 | 4.216816 |
| C | -4.639398 | 3.118598 | -0.392515 |
| H | -4.601655 | 1.732241 | 1.244053 |
| C | -3.018477 | 2.750220 | -2.147529 |
| H | -1.677014 | 1.086914 | -1.852049 |
| C | -4.762086 | -3.474849 | 1.445079 |

| | | | |
|---|---|---|---|
| H | -2.838369 | -2.727875 | 2.029240 |
| C | -5.984593 | -2.136869 | -0.147162 |
| H | -5.029155 | -0.328854 | -0.801785 |
| C | 0.784782 | 4.720372 | -2.857303 |
| H | 1.771532 | 3.135157 | -3.939153 |
| H | -0.211712 | 6.091489 | -1.521849 |
| C | 4.916817 | 1.882435 | 2.624387 |
| H | 4.893164 | 3.976241 | 2.099404 |
| H | 4.666835 | -0.230804 | 2.975925 |
| C | -4.078801 | 3.486315 | -1.619498 |
| H | -5.468275 | 3.686967 | 0.021095 |
| H | -2.568677 | 3.036340 | -3.093588 |
| C | -5.898360 | -3.272929 | 0.656980 |
| H | -4.680243 | -4.360067 | 2.069973 |
| H | -6.861869 | -1.972770 | -0.767256 |
| H | 0.809432 | 5.419242 | -3.689694 |
| H | 5.893903 | 1.929672 | 3.098153 |
| H | -4.468369 | 4.345547 | -2.158836 |
| H | -6.706862 | -3.998665 | 0.668118 |
| C | 2.600974 | -1.236158 | -0.890278 |
| O | 1.703893 | -1.658571 | -0.055474 |
| O | 2.432251 | -0.361093 | -1.751093 |
| Si | -0.703248 | -2.829700 | -1.140552 |
| C | 0.560445 | -3.051399 | -2.545700 |
| H | 1.507713 | -3.445374 | -2.166685 |
| H | 0.160222 | -3.763495 | -3.280127 |
| H | 0.779723 | -2.111636 | -3.060679 |
| C | -0.512592 | -4.265119 | 0.090429 |
| H | -0.529508 | -5.226375 | -0.441816 |
| H | 0.447040 | -4.179800 | 0.610688 |

| | | | |
|---|---|---|---|
| H | -1.312779 | -4.283575 | 0.838242 |
| C | -2.392848 | -2.973176 | -2.014561 |
| H | -2.588952 | -2.108814 | -2.660754 |
| H | -2.355834 | -3.860260 | -2.662662 |
| H | -3.240948 | -3.092184 | -1.338343 |
| C | 3.950743 | -1.907201 | -0.761765 |
| C | 5.000352 | -1.488456 | -1.589872 |
| C | 4.176020 | -2.936774 | 0.162078 |
| C | 6.255945 | -2.086955 | -1.495522 |
| H | 4.806097 | -0.691801 | -2.300223 |
| C | 5.432192 | -3.537034 | 0.256005 |
| H | 3.357496 | -3.257210 | 0.797694 |
| C | 6.474801 | -3.113098 | -0.572043 |
| H | 7.064777 | -1.755144 | -2.141896 |
| H | 5.598146 | -4.337143 | 0.973418 |
| H | 7.453462 | -3.581308 | -0.499063 |

**TS-S6**

| | | | |
|---|---|---|---|
| Pd | -0.231829 | -0.581659 | -0.042580 |
| P | 0.676192 | 1.752796 | 0.590664 |
| P | -2.412479 | -0.275183 | 0.694947 |
| C | -0.389807 | 2.593143 | 1.878911 |
| C | 0.725743 | 3.002620 | -0.771551 |
| C | 2.352079 | 1.899651 | 1.362611 |
| C | -2.405455 | 0.192369 | 2.534232 |
| C | -3.297834 | 1.137312 | -0.110367 |
| C | -3.716881 | -1.600424 | 0.707555 |
| C | -0.494801 | 1.841952 | 3.218184 |
| H | -1.383566 | 2.712783 | 1.429844 |
| H | -0.004981 | 3.600372 | 2.081006 |

| | | | |
|---|---|---|---|
| C | 1.365969 | 2.625038 | -1.966917 |
| C | 0.134302 | 4.273956 | -0.697179 |
| C | 3.029299 | 3.124275 | 1.481220 |
| C | 2.956978 | 0.740213 | 1.870252 |
| C | -1.019791 | 0.395851 | 3.184355 |
| H | -2.924088 | -0.623080 | 3.046203 |
| H | -3.030612 | 1.083266 | 2.668045 |
| C | -4.482690 | 1.686284 | 0.412389 |
| C | -2.763056 | 1.687045 | -1.284326 |
| C | -3.549132 | -2.705062 | 1.561488 |
| C | -4.825680 | -1.588983 | -0.152219 |
| H | 0.490864 | 1.836020 | 3.700587 |
| H | -1.149524 | 2.434522 | 3.873497 |
| C | 1.428401 | 3.508278 | -3.044846 |
| H | 1.806661 | 1.635612 | -2.050991 |
| C | 0.190611 | 5.151276 | -1.783816 |
| H | -0.379909 | 4.593720 | 0.203036 |
| C | 4.277045 | 3.186558 | 2.101250 |
| H | 2.586060 | 4.029817 | 1.075290 |
| C | 4.205812 | 0.803140 | 2.493701 |
| H | 2.454547 | -0.217324 | 1.761555 |
| H | -0.290736 | -0.260516 | 2.692637 |
| H | -1.081020 | 0.055125 | 4.226671 |
| C | -5.108334 | 2.760097 | -0.220215 |
| H | -4.929448 | 1.265621 | 1.309537 |
| C | -3.389839 | 2.763137 | -1.919340 |
| H | -1.843154 | 1.274956 | -1.690155 |
| C | -4.472907 | -3.749600 | 1.575338 |
| H | -2.683812 | -2.757619 | 2.218155 |
| C | -5.747460 | -2.639369 | -0.143638 |

| | | | |
|---|---|---|---|
| H | -4.978045 | -0.756581 | -0.831314 |
| C | 0.840715 | 4.773149 | -2.958724 |
| H | 1.931507 | 3.201663 | -3.958303 |
| H | -0.274068 | 6.131211 | -1.706978 |
| C | 4.866753 | 2.026097 | 2.610863 |
| H | 4.792463 | 4.140294 | 2.182639 |
| H | 4.664743 | -0.106200 | 2.872195 |
| C | -4.561509 | 3.301477 | -1.387789 |
| H | -6.023522 | 3.173303 | 0.195976 |
| H | -2.953828 | 3.181594 | -2.821987 |
| C | -5.578599 | -3.719933 | 0.721830 |
| H | -4.324795 | -4.589741 | 2.248802 |
| H | -6.600796 | -2.607248 | -0.816152 |
| H | 0.885386 | 5.456765 | -3.802730 |
| H | 5.841555 | 2.075883 | 3.089079 |
| H | -5.049213 | 4.139608 | -1.878651 |
| H | -6.297436 | -4.534711 | 0.728847 |
| C | 2.529884 | -1.374955 | -1.040677 |
| O | 1.486575 | -1.911642 | -0.417669 |
| O | 2.454411 | -0.445885 | -1.837689 |
| Si | -0.173225 | -2.713728 | -1.369571 |
| C | -1.761916 | -2.347949 | -2.387209 |
| H | -2.648857 | -2.215060 | -1.764502 |
| H | -1.631885 | -1.447464 | -2.999061 |
| H | -1.942197 | -3.188468 | -3.074306 |
| C | -0.463342 | -4.145932 | -0.166547 |
| H | -1.389844 | -4.017736 | 0.400554 |
| H | -0.538214 | -5.088073 | -0.726840 |
| H | 0.368078 | -4.239919 | 0.539412 |
| C | 0.986056 | -3.295526 | -2.765903 |

| | | | |
|---|---|---|---|
| H | 1.303863 | -2.464439 | -3.405158 |
| H | 1.887304 | -3.787832 | -2.384909 |
| H | 0.448198 | -4.011445 | -3.400904 |
| C | 3.849220 | -1.998120 | -0.684492 |
| C | 3.948366 | -3.052566 | 0.234403 |
| C | 5.011488 | -1.498215 | -1.288946 |
| C | 5.194252 | -3.600452 | 0.540584 |
| H | 3.046167 | -3.432921 | 0.700580 |
| C | 6.254673 | -2.047271 | -0.982602 |
| H | 4.915076 | -0.680505 | -1.995233 |
| C | 6.348401 | -3.099724 | -0.067177 |
| H | 5.264952 | -4.419170 | 1.252033 |
| H | 7.151289 | -1.656124 | -1.456207 |
| H | 7.318458 | -3.528482 | 0.171463 |

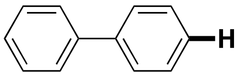

**2a**

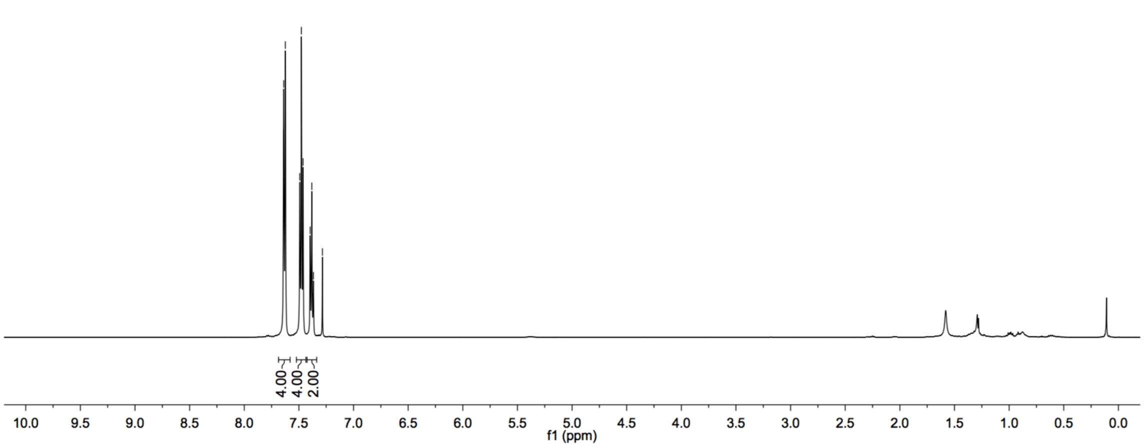

7.64
7.62
7.49
7.48
7.46
7.40
7.38
7.37
7.28
4.00
4.00
2.00
10.0 9.5 9.0 8.5 8.0 7.5 7.0 6.5 6.0 5.5 5.0 4.5 4.0 3.5 3.0 2.5 2.0 1.5 1.0 0.5 0.0
f1 (ppm)


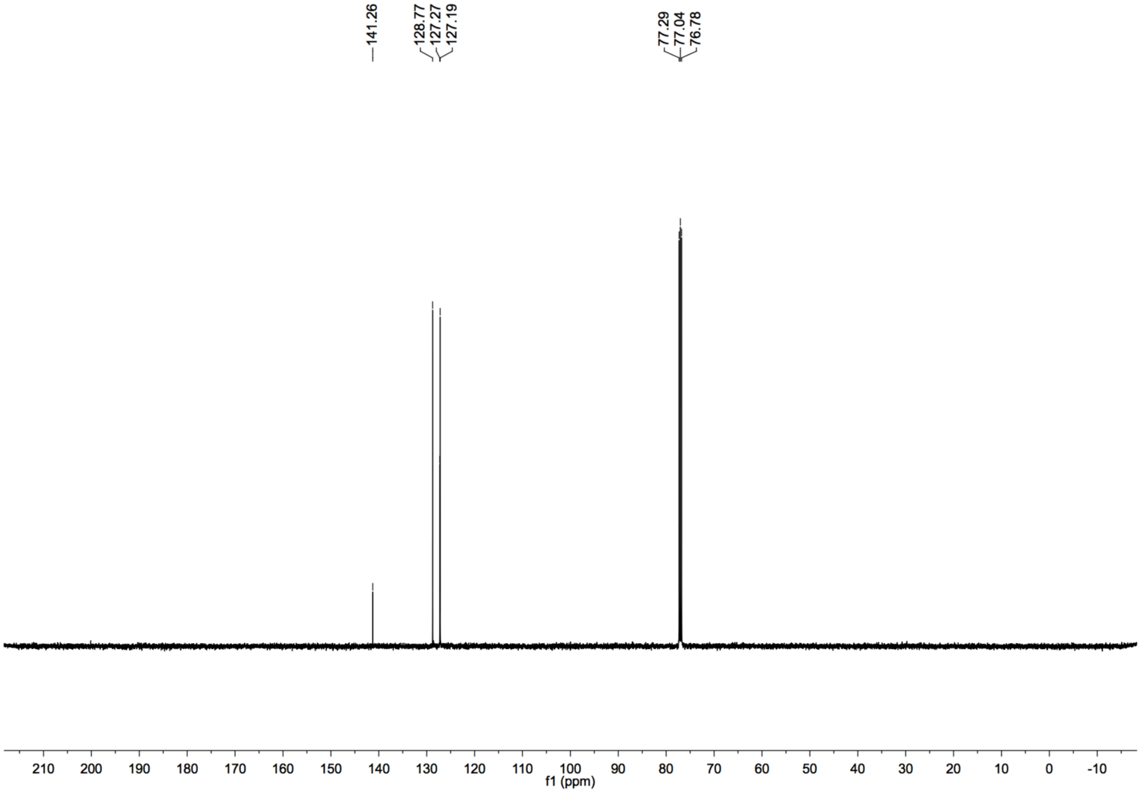

141.26
128.77
127.27
127.19
77.29
77.04
76.78
210 200 190 180 170 160 150 140 130 120 110 100 90 80 70 60 50 40 30 20 10 0 -10
f1 (ppm)

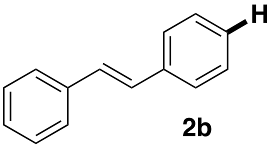
H

**2b**

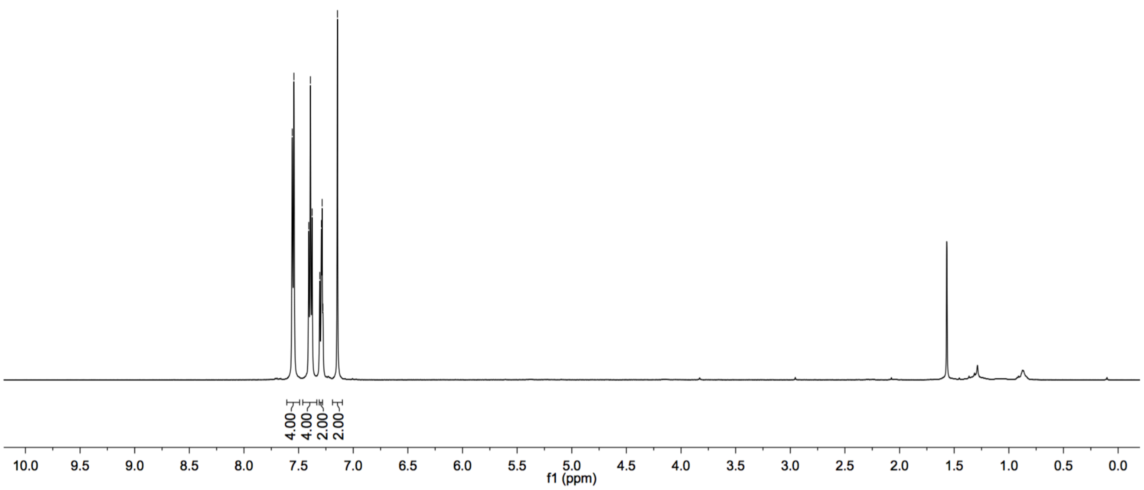
7.56
7.54
7.41
7.39
7.38
7.31
7.29
7.28
7.28
7.14
4.00
4.00
2.00
2.00
10.0 9.5 9.0 8.5 8.0 7.5 7.0 6.5 6.0 5.5 5.0 4.5 4.0 3.5 3.0 2.5 2.0 1.5 1.0 0.5 0.0
f1 (ppm)

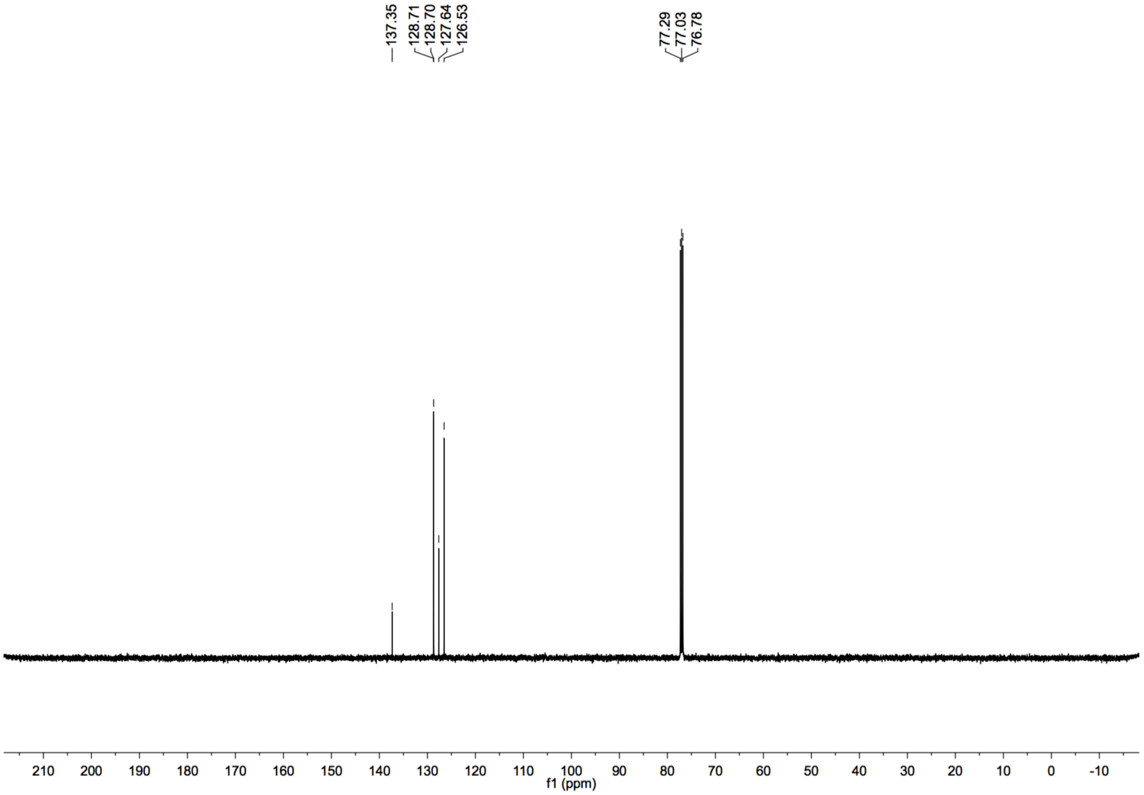
137.35
128.71
128.70
127.64
126.53
77.29
77.03
76.78
210 200 190 180 170 160 150 140 130 120 110 100 90 80 70 60 50 40 30 20 10 0 -10
f1 (ppm)

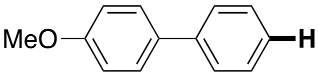
MeO
H

**2c**

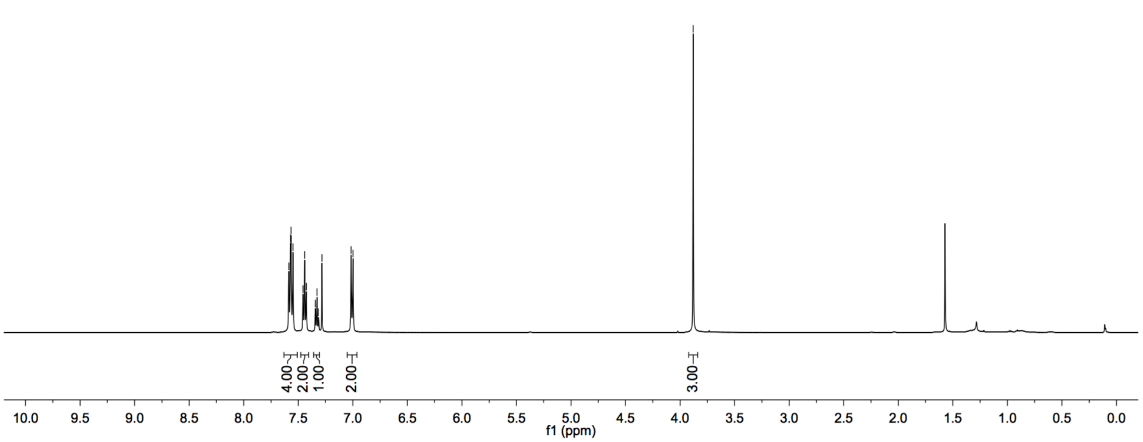
7.59
7.57
7.57
7.55
7.46
7.44
7.43
7.34
7.33
7.31
7.28
7.02
7.00
3.88
4.00
2.00
1.00
2.00
3.00
f1 (ppm)

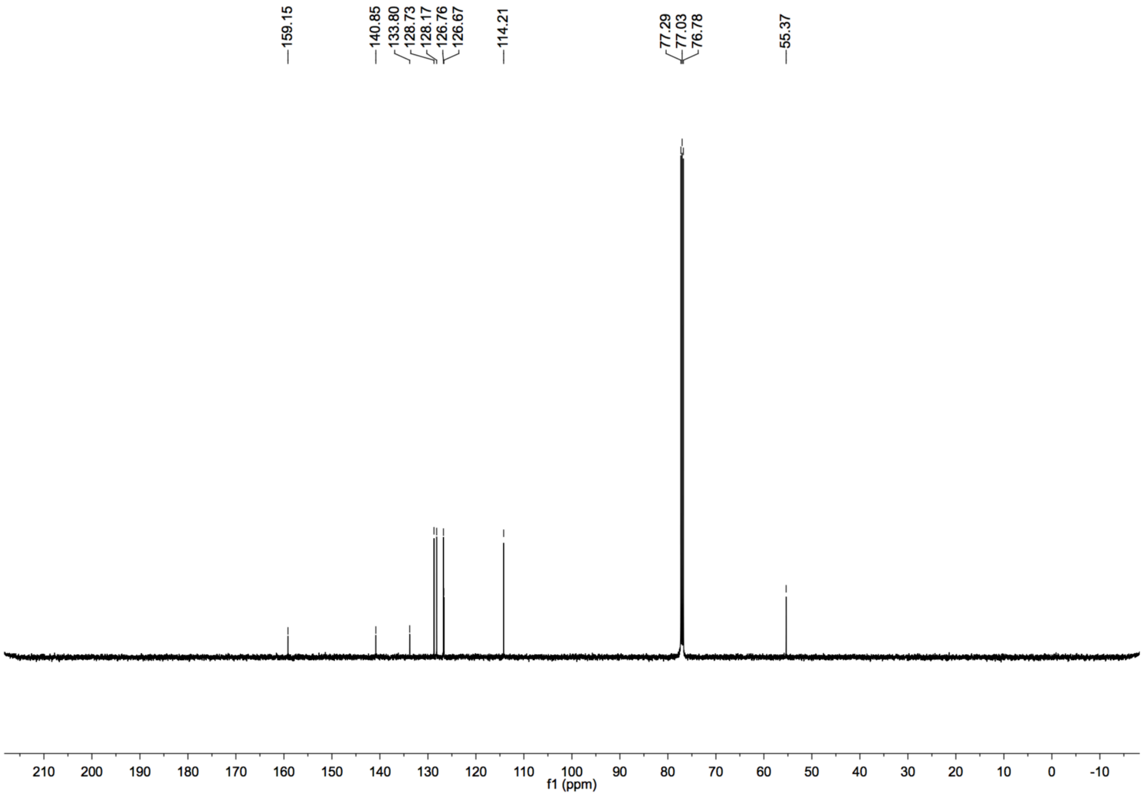
159.15
140.85
133.80
128.73
128.17
126.76
126.67
114.21
77.29
77.03
76.78
55.37
f1 (ppm)

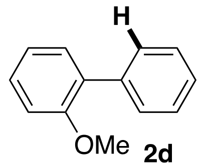
H
OMe

**2d**

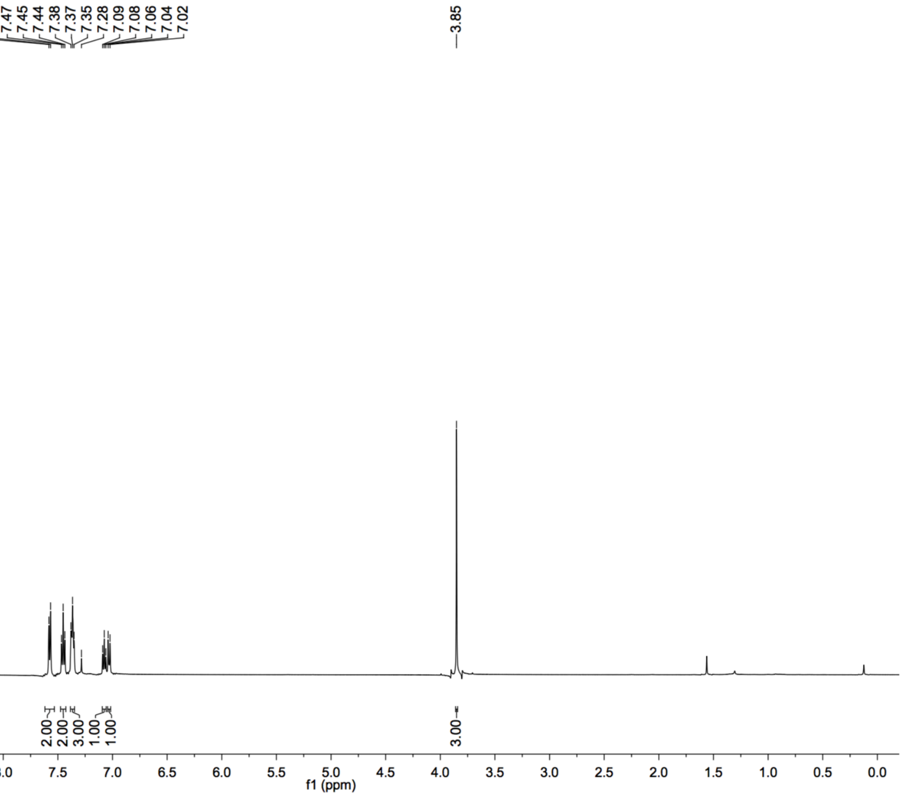
7.58
7.57
7.47
7.45
7.44
7.38
7.37
7.35
7.28
7.09
7.08
7.06
7.04
7.02
3.85
2.00
2.00
3.00
1.00
1.00
3.00
10.0 9.5 9.0 8.5 8.0 7.5 7.0 6.5 6.0 5.5 5.0 4.5 4.0 3.5 3.0 2.5 2.0 1.5 1.0 0.5 0.0
f1 (ppm)

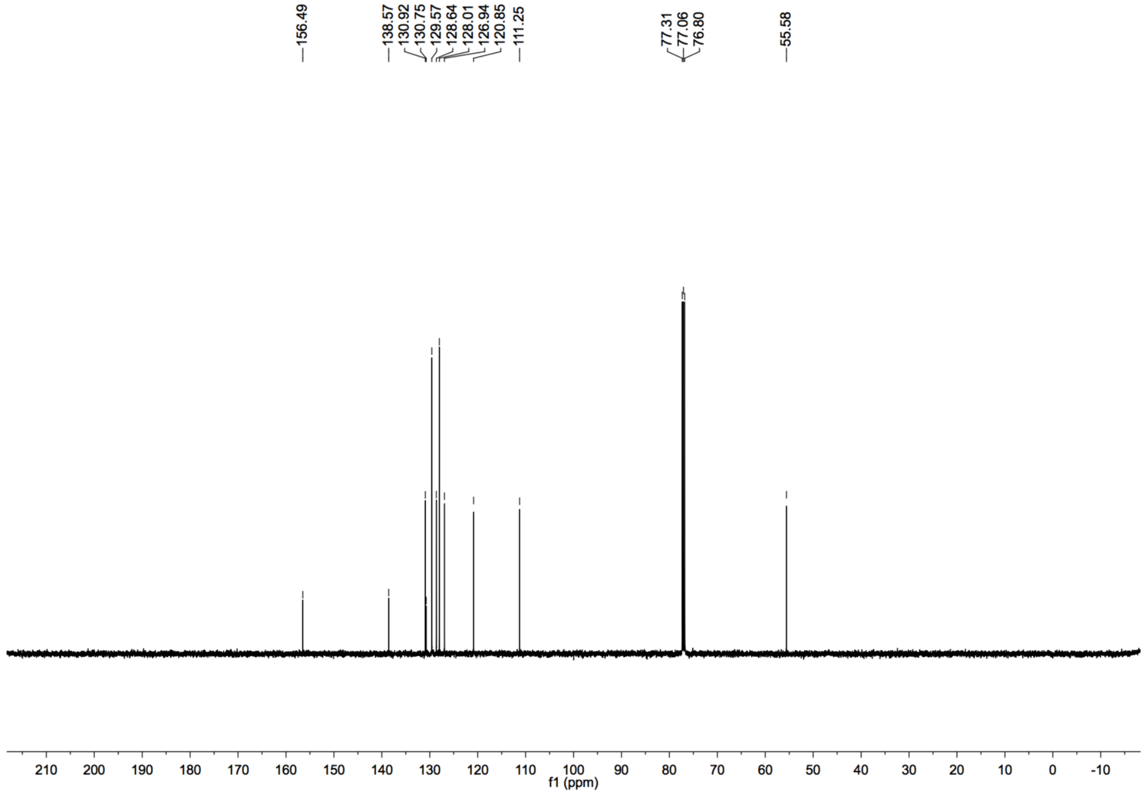
156.49
138.57
130.92
130.75
129.57
128.64
128.01
126.94
120.85
111.25
77.31
77.06
76.80
55.58
210 200 190 180 170 160 150 140 130 120 110 100 90 80 70 60 50 40 30 20 10 0 -10
f1 (ppm)

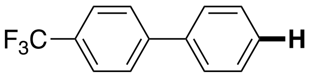
F3C
H

**2e**

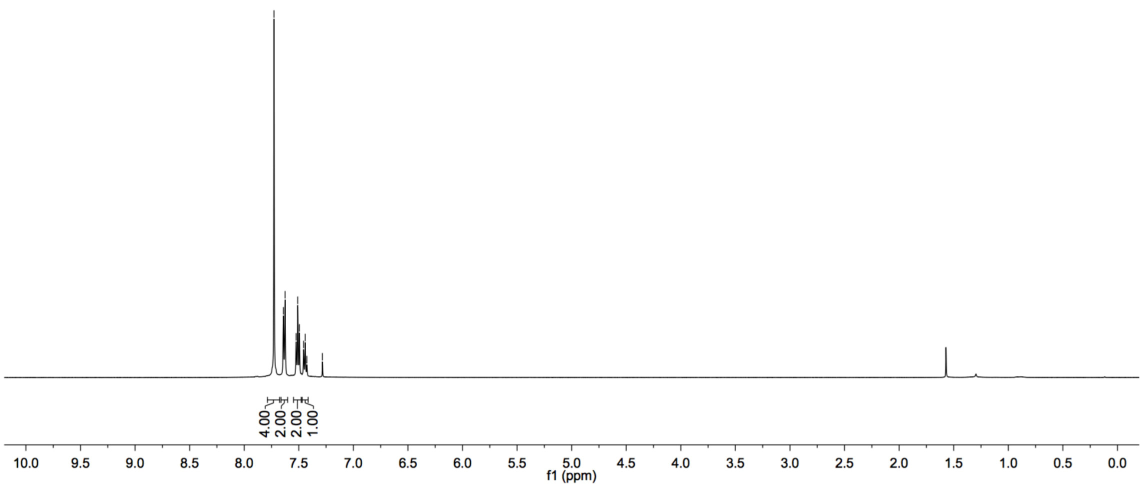
7.73
7.64
7.63
7.52
7.51
7.49
7.46
7.44
7.43
7.28
4.00
2.00
2.00
1.00
10.0 9.5 9.0 8.5 8.0 7.5 7.0 6.5 6.0 5.5 5.0 4.5 4.0 3.5 3.0 2.5 2.0 1.5 1.0 0.5 0.0
f1 (ppm)

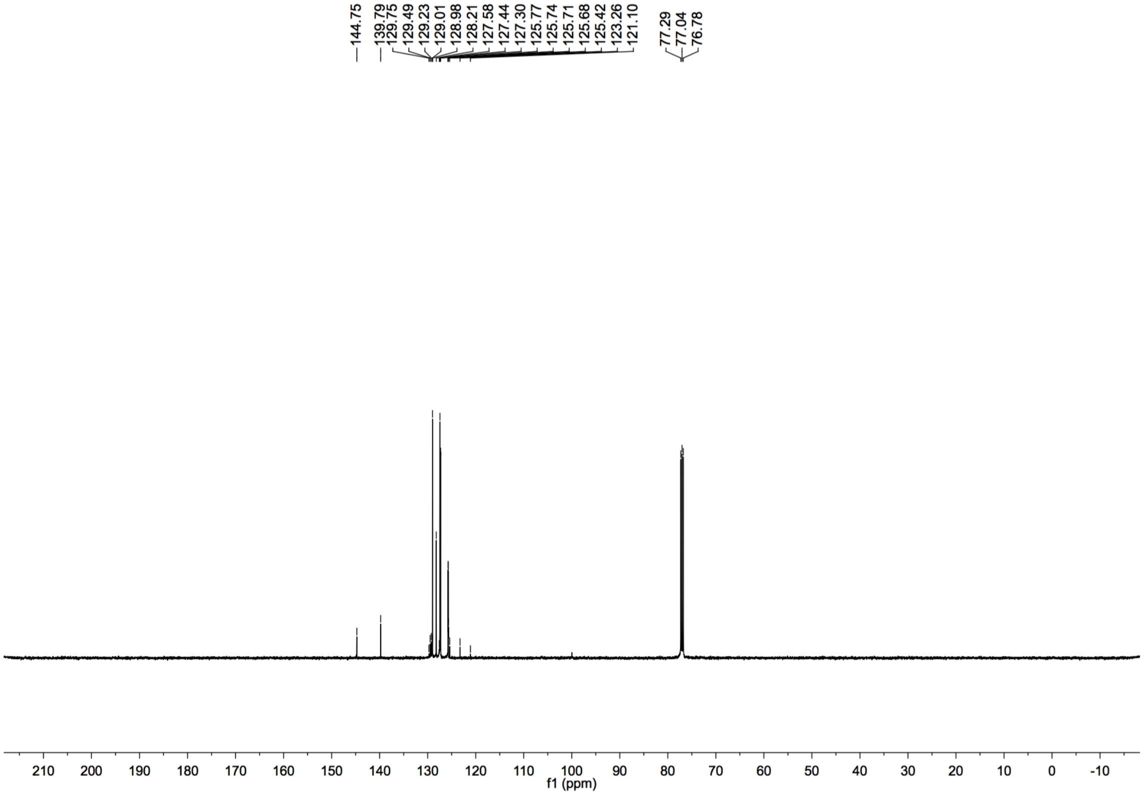
144.75
139.79
129.75
129.49
129.23
129.01
128.98
128.21
127.58
127.44
127.30
125.77
125.74
125.71
125.68
125.42
123.26
121.10
77.29
77.04
76.78
210 200 190 180 170 160 150 140 130 120 110 100 90 80 70 60 50 40 30 20 10 0 -10
f1 (ppm)

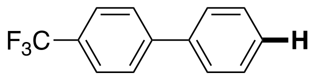
F3C
H

**2e**

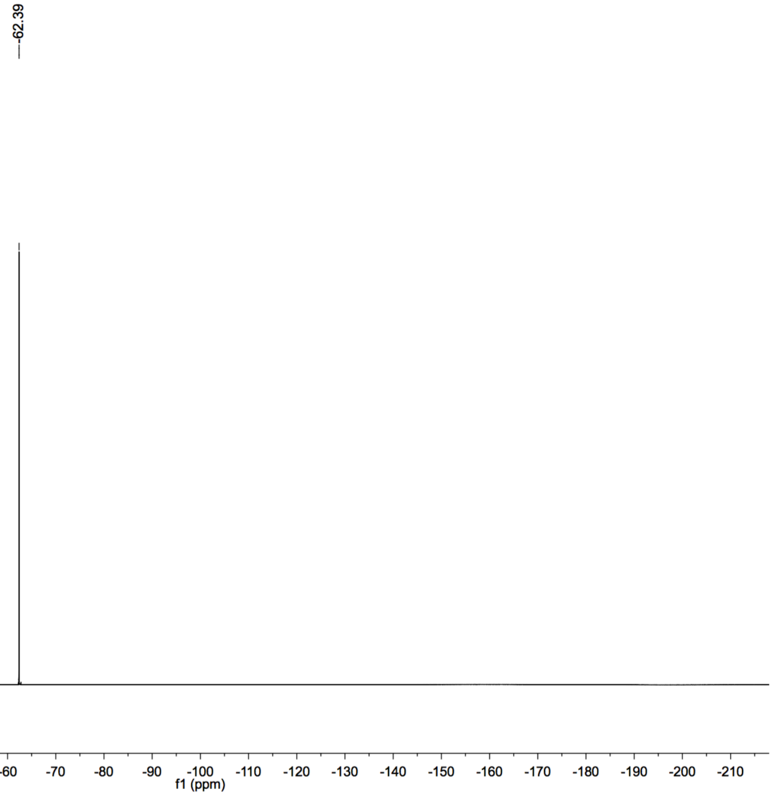
-62.39
10
0
-10
-20
-30
-40
-50
-60
-70
-80
-90
-100
-110
-120
-130
-140
-150
-160
-170
-180
-190
-200
-210
f1 (ppm)

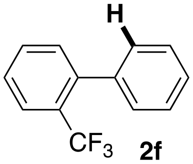
H
CF3

**2f**

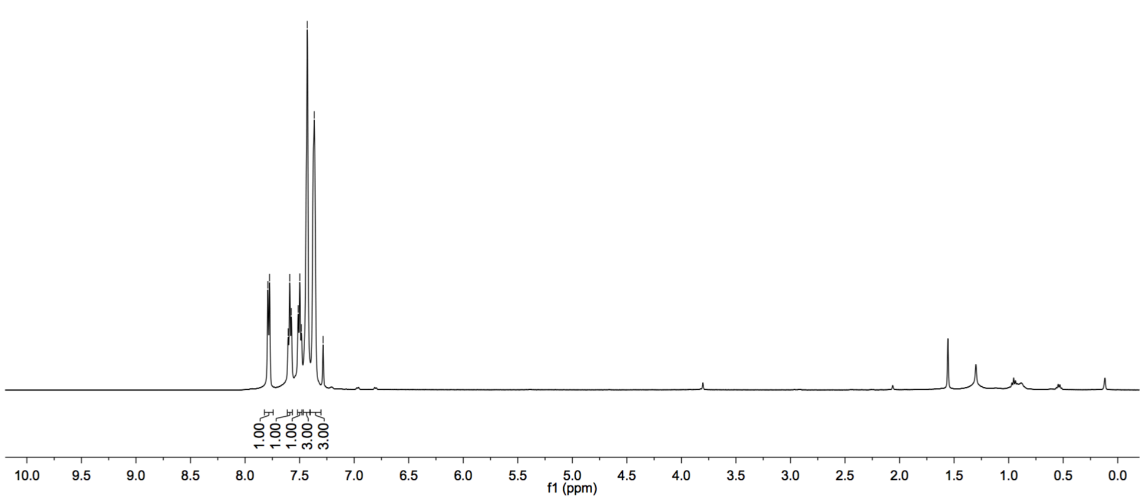
7.79
7.78
7.60
7.59
7.58
7.51
7.50
7.48
7.43
7.37
7.28
1.00
1.00
1.00
3.00
3.00
10.0 9.5 9.0 8.5 8.0 7.5 7.0 6.5 6.0 5.5 5.0 4.5 4.0 3.5 3.0 2.5 2.0 1.5 1.0 0.5 0.0
f1 (ppm)

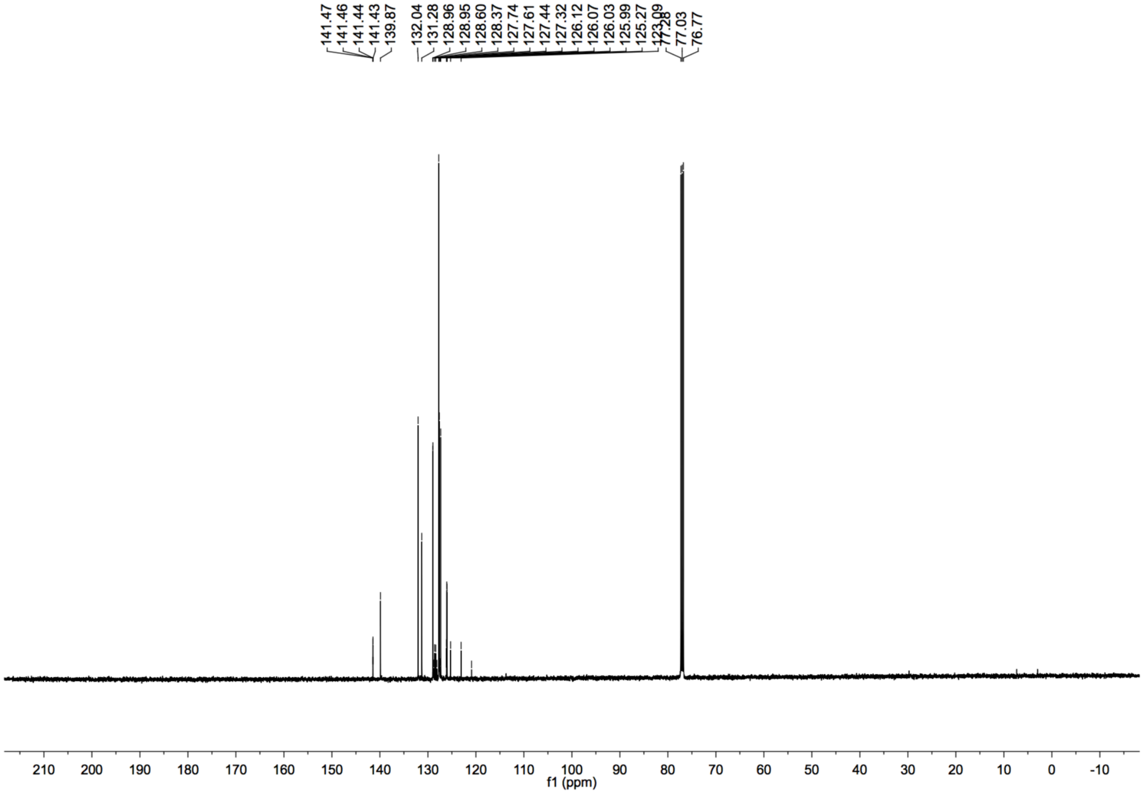
141.47
141.46
141.44
141.43
139.87
132.04
131.28
128.96
128.95
128.60
128.37
127.74
127.61
127.44
127.32
126.12
126.07
126.03
125.99
125.27
123.09
77.28
77.03
76.77
210 200 190 180 170 160 150 140 130 120 110 100 90 80 70 60 50 40 30 20 10 0 -10
f1 (ppm)

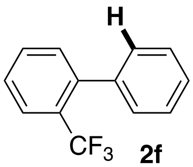

**2f**

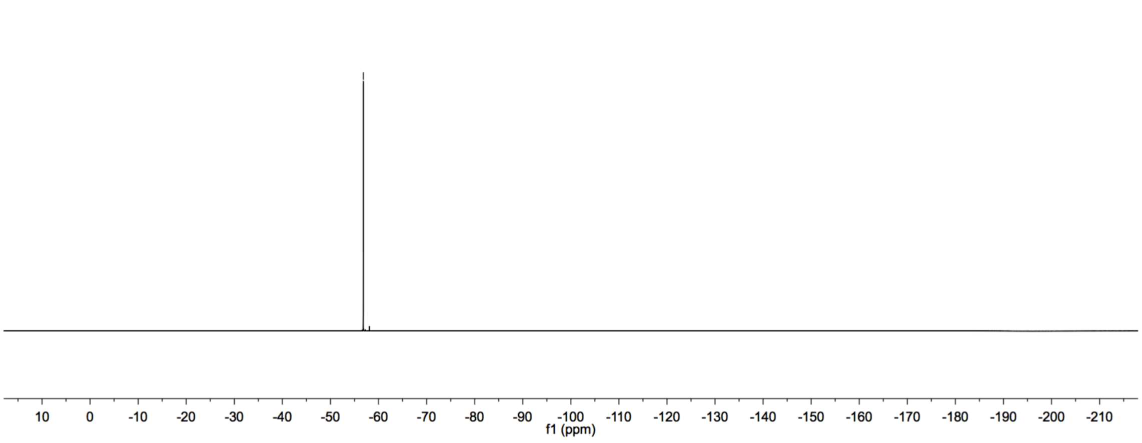

-56.83
10 0 -10 -20 -30 -40 -50 -60 -70 -80 -90 -100 -110 -120 -130 -140 -150 -160 -170 -180 -190 -200 -210
f1 (ppm)

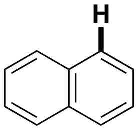
H

2g

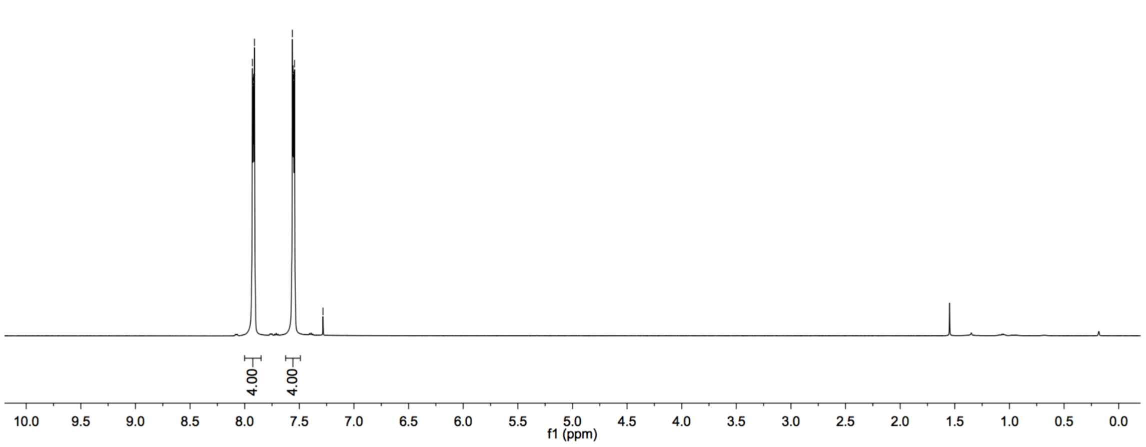
7.93
7.92
7.92
7.91
7.56
7.56
7.55
7.54
7.28
4.00
4.00
10.0 9.5 9.0 8.5 8.0 7.5 7.0 6.5 6.0 5.5 5.0 4.5 4.0 3.5 3.0 2.5 2.0 1.5 1.0 0.5 0.0
f1 (ppm)

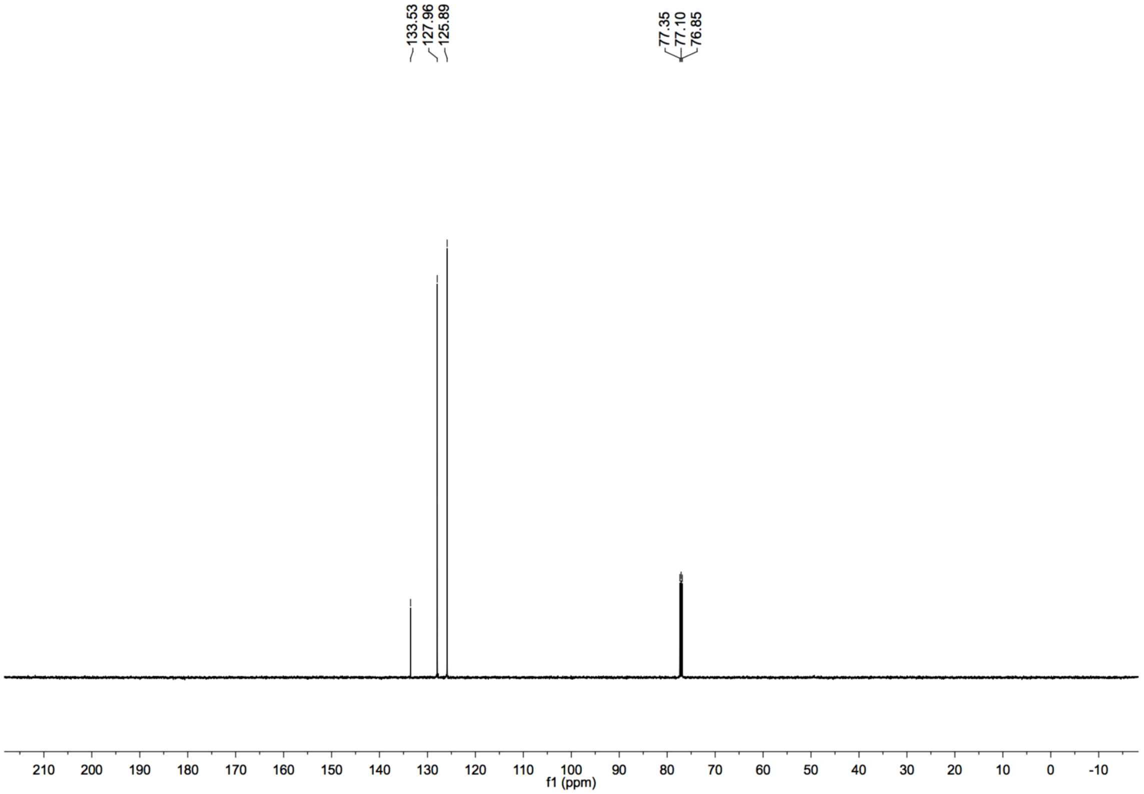
133.53
127.96
125.89
77.35
77.10
76.85
210 200 190 180 170 160 150 140 130 120 110 100 90 80 70 60 50 40 30 20 10 0 -10
f1 (ppm)

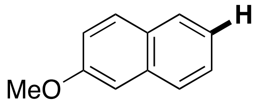
MeO
H

**2h**

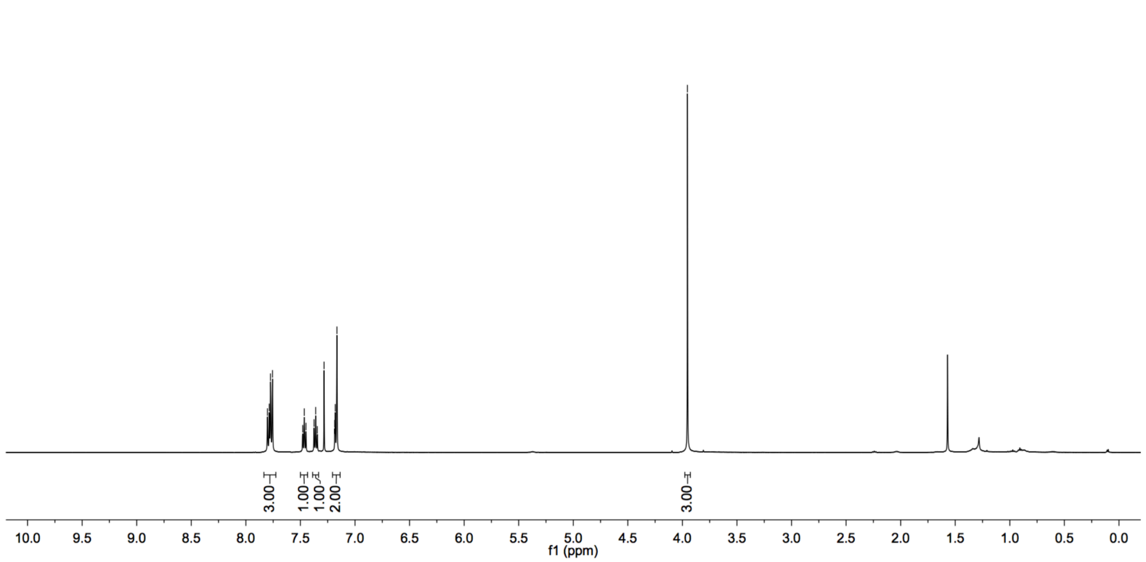
7.80
7.79
7.77
7.76
7.48
7.46
7.45
7.37
7.36
7.35
7.28
7.19
7.18
7.17
3.95
3.00
1.00
1.00
2.00
3.00
10.0 9.5 9.0 8.5 8.0 7.5 7.0 6.5 6.0 5.5 5.0 4.5 4.0 3.5 3.0 2.5 2.0 1.5 1.0 0.5 0.0
f1 (ppm)

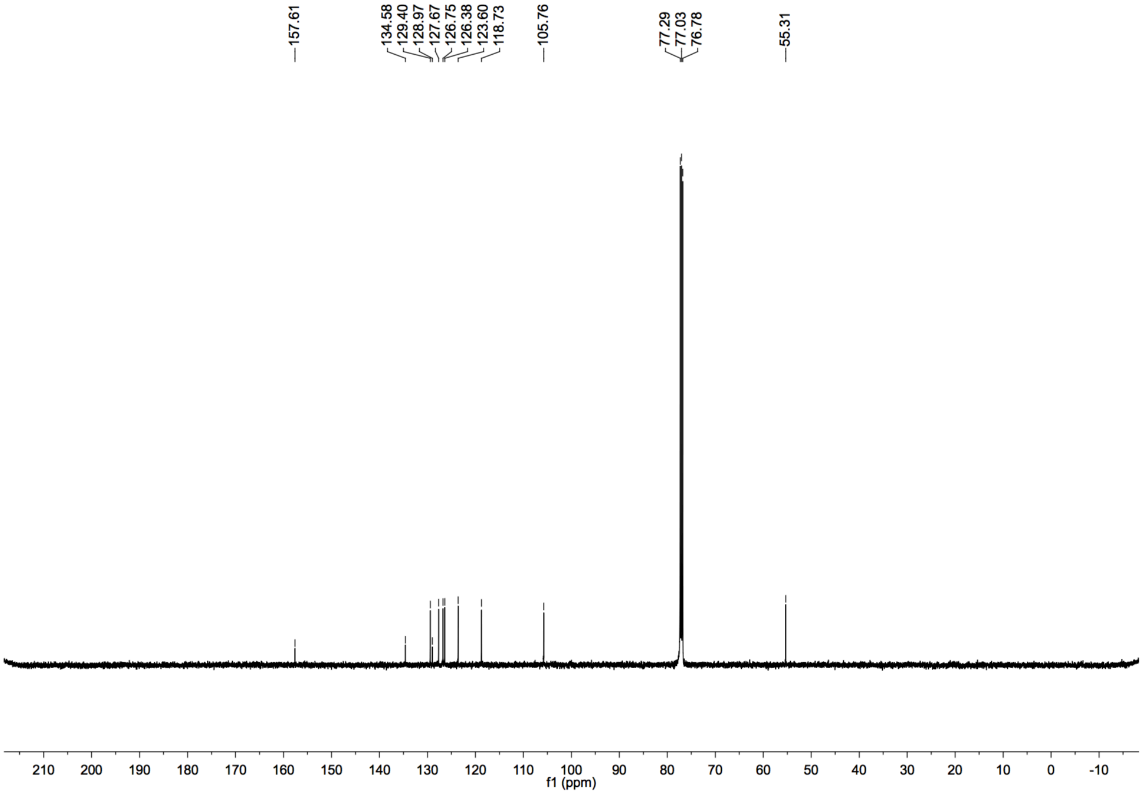
157.61
134.58
129.40
128.97
127.67
126.75
126.38
123.60
118.73
105.76
77.29
77.03
76.78
55.31
210 200 190 180 170 160 150 140 130 120 110 100 90 80 70 60 50 40 30 20 10 0 -10
f1 (ppm)

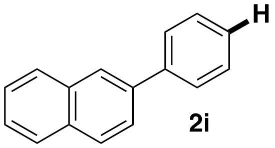

2i

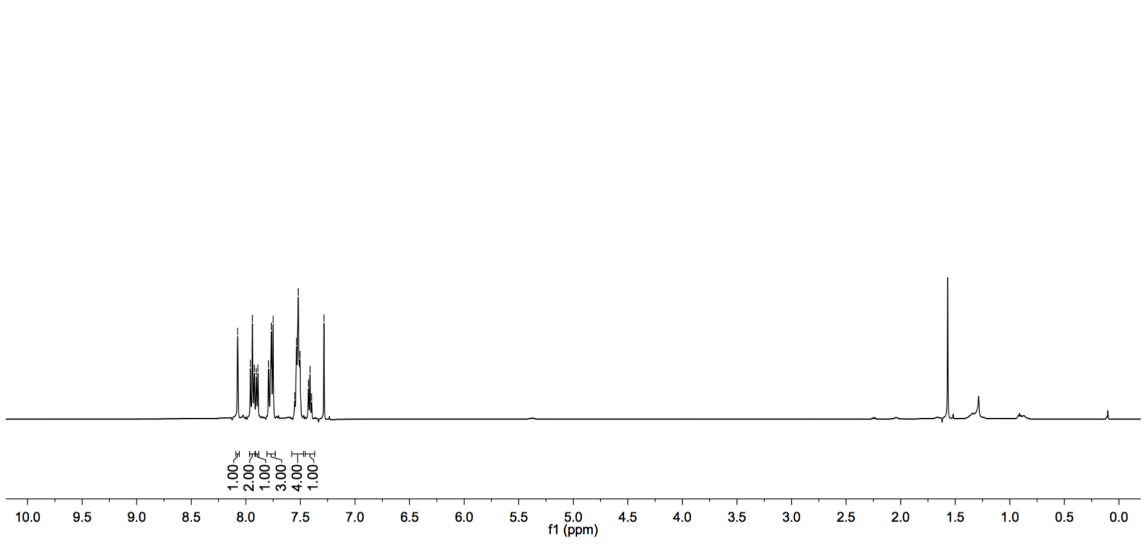

8.08
7.96
7.94
7.92
7.91
7.89
7.79
7.77
7.75
7.55
7.54
7.52
7.50
7.43
7.41
7.40
7.28
1.00
2.00
1.00
3.00
4.00
1.00
10.0 9.5 9.0 8.5 8.0 7.5 7.0 6.5 6.0 5.5 5.0 4.5 4.0 3.5 3.0 2.5 2.0 1.5 1.0 0.5 0.0
f1 (ppm)


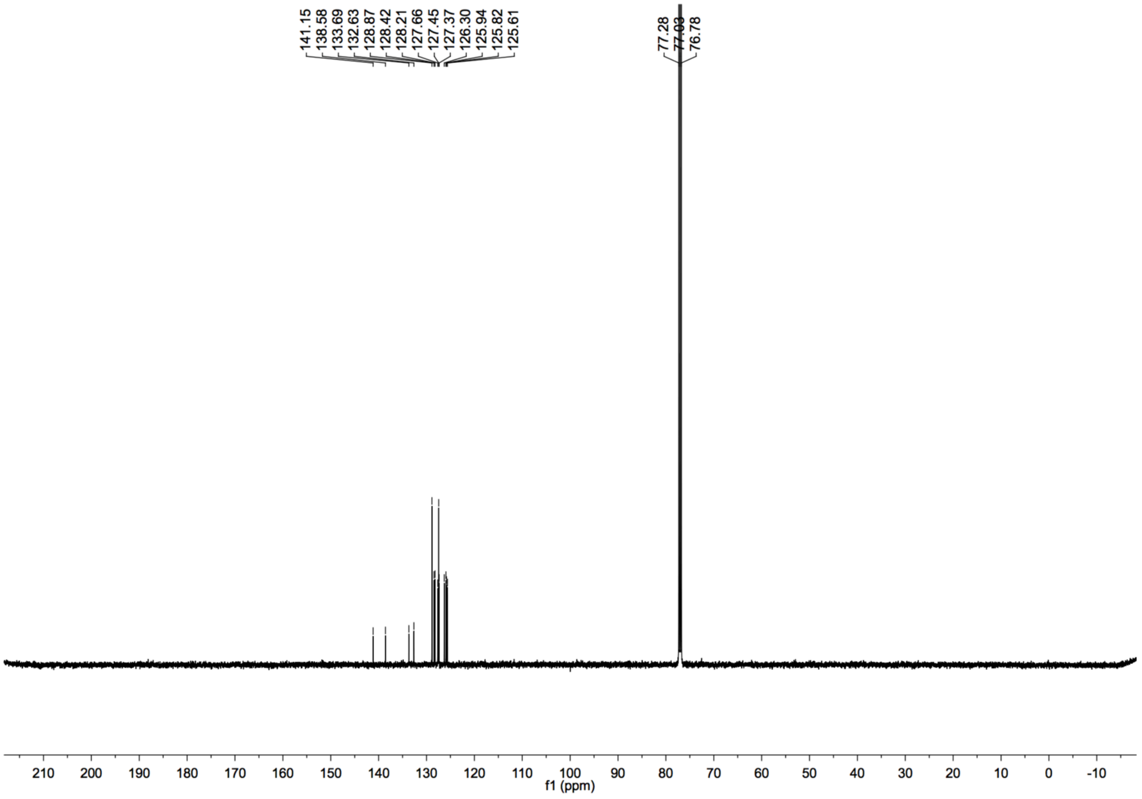

141.15
138.58
133.69
132.63
128.87
128.42
128.21
127.66
127.45
127.37
126.30
125.94
125.82
125.61
77.28
77.03
76.78
210 200 190 180 170 160 150 140 130 120 110 100 90 80 70 60 50 40 30 20 10 0 -10
f1 (ppm)

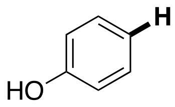

H
HO


**2j**

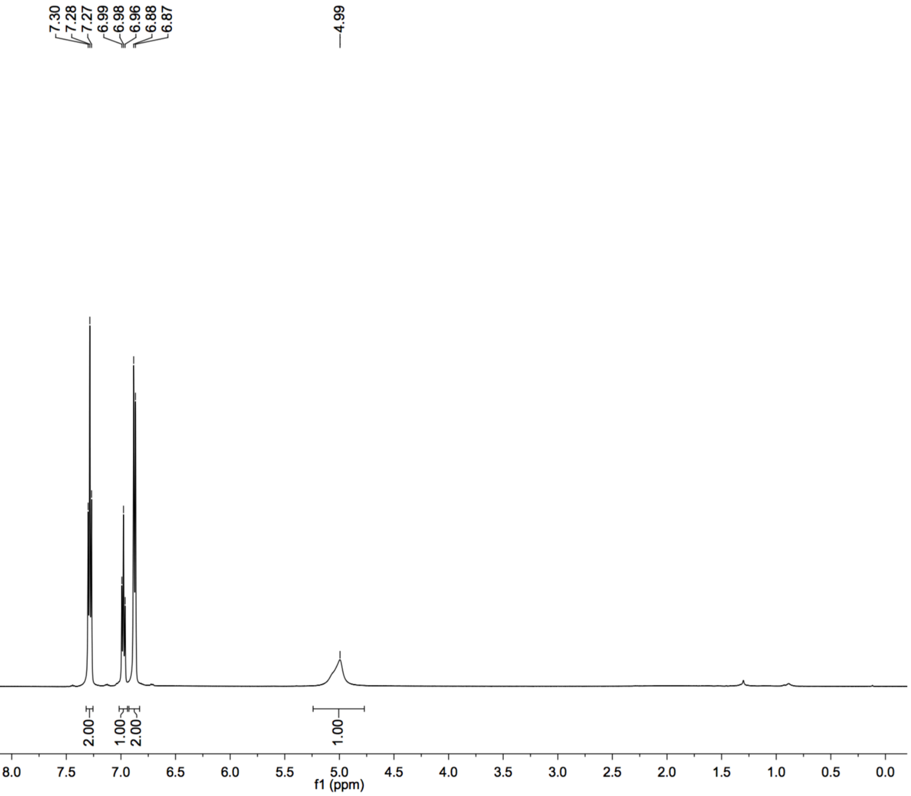

7.30
7.28
7.27
6.99
6.98
6.96
6.88
6.87
4.99
2.00
1.00
2.00
1.00
f1 (ppm)


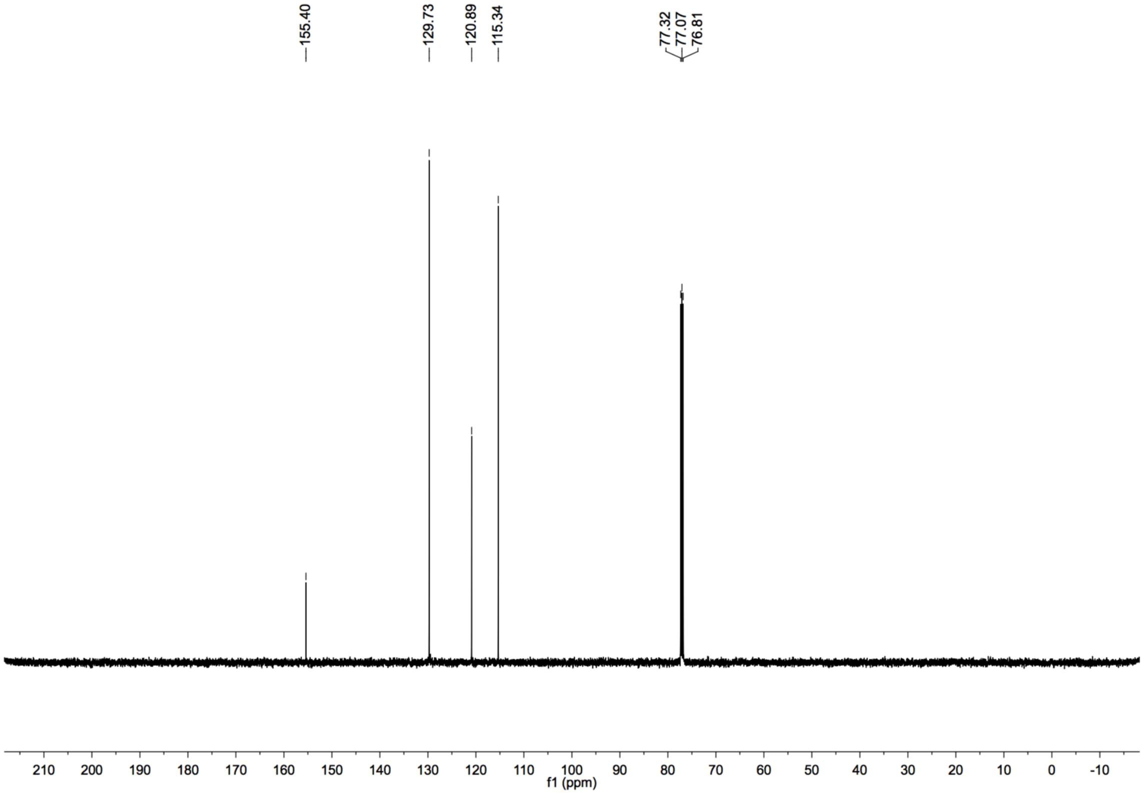

155.40
129.73
120.89
115.34
77.32
77.07
76.81
f1 (ppm)

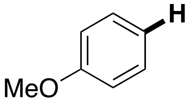
MeO
H

**2k**

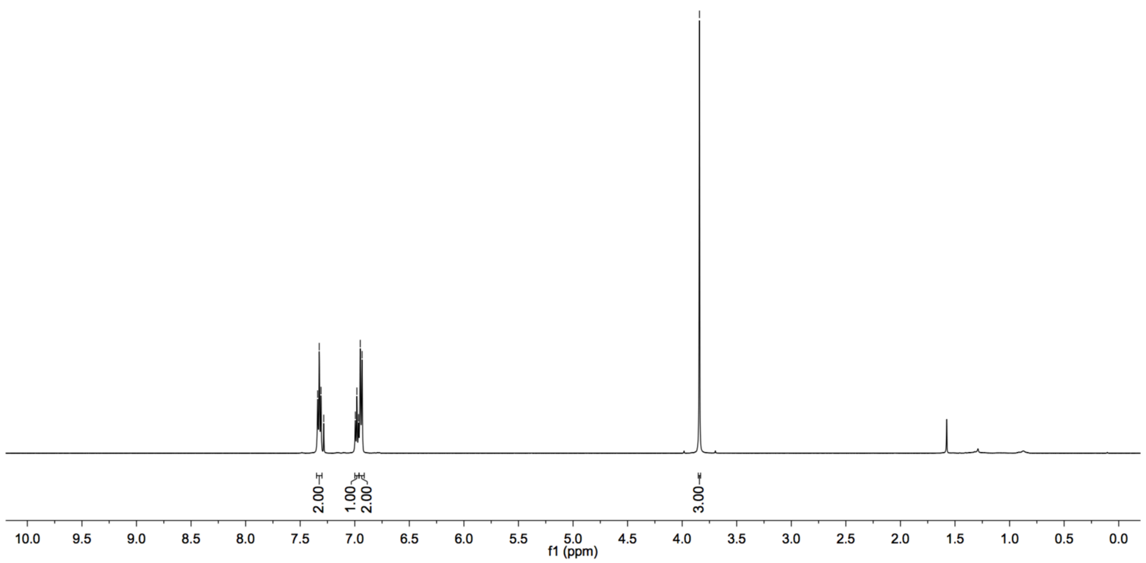
7.34
7.33
7.31
7.28
6.99
6.98
6.97
6.95
6.93
3.84
2.00
1.00
2.00
3.00
f1 (ppm)

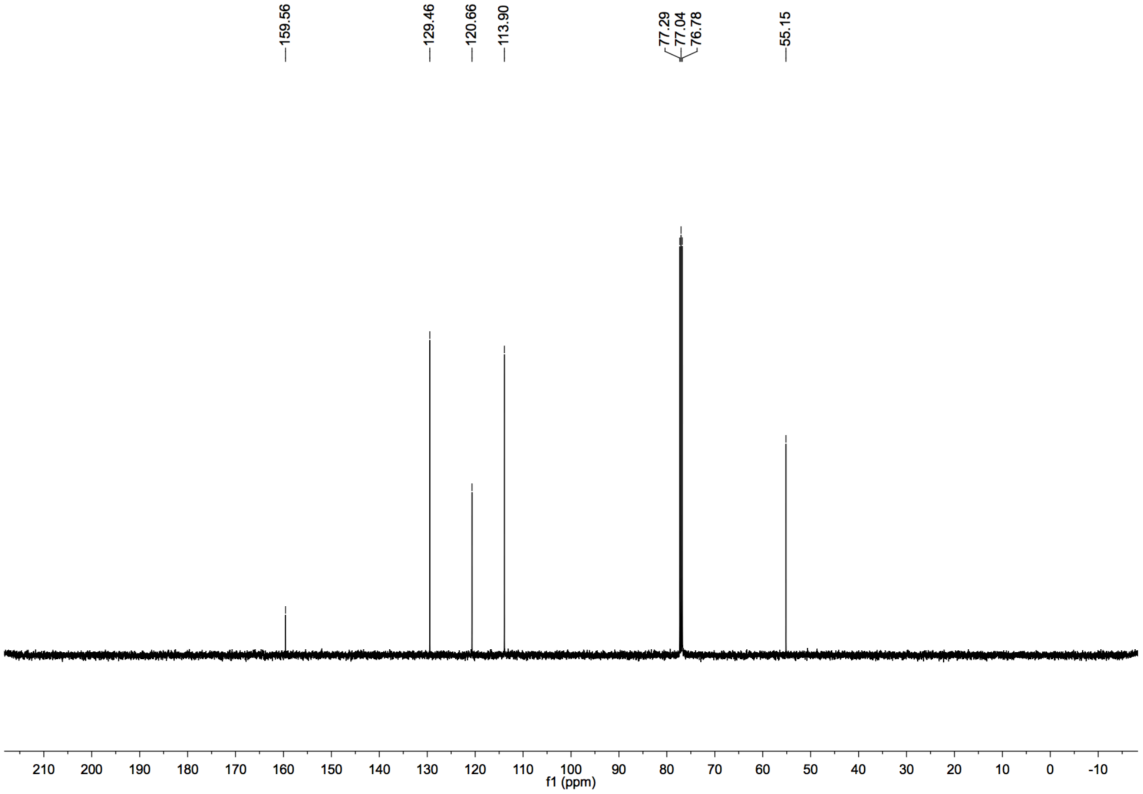
159.56
129.46
120.66
113.90
77.29
77.04
76.78
55.15
f1 (ppm)

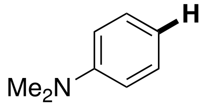
H
$Me_2N$

**2l**

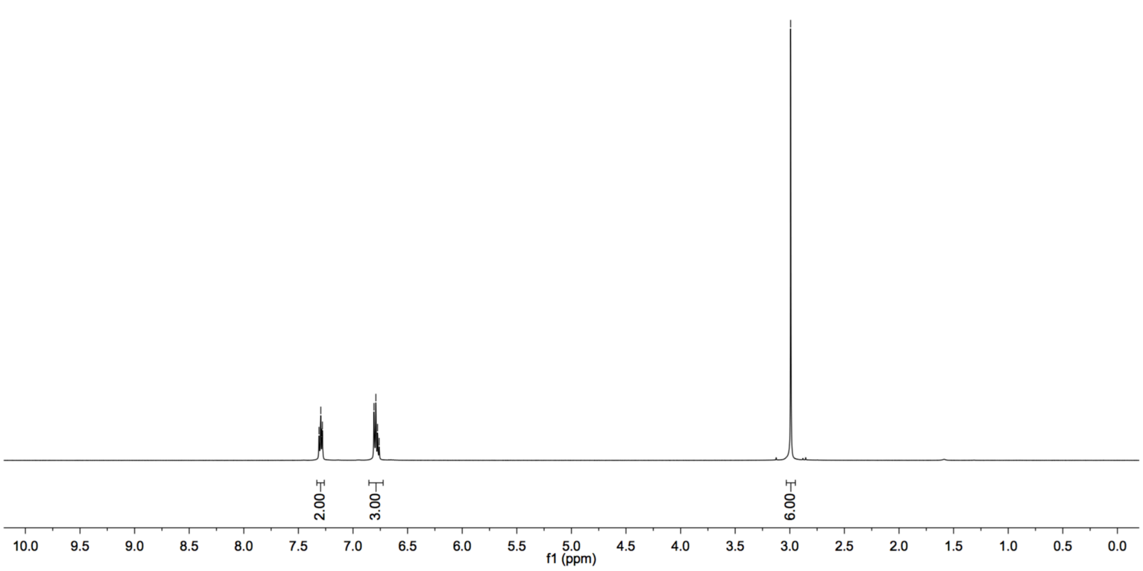
7.31
7.30
7.29
7.28
6.81
6.79
6.78
6.76
2.99
2.00
3.00
6.00
f1 (ppm)

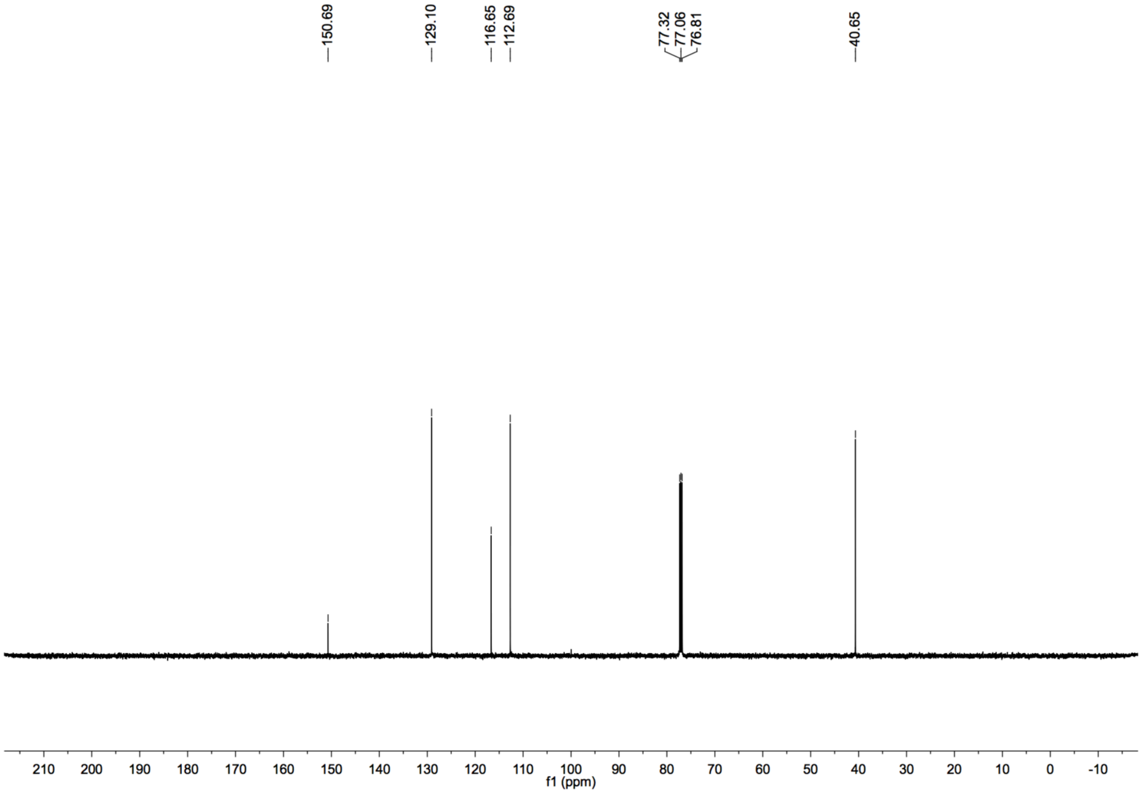
150.69
129.10
116.65
112.69
77.32
77.06
76.81
40.65
f1 (ppm)

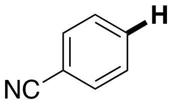
H
NC

**2m**

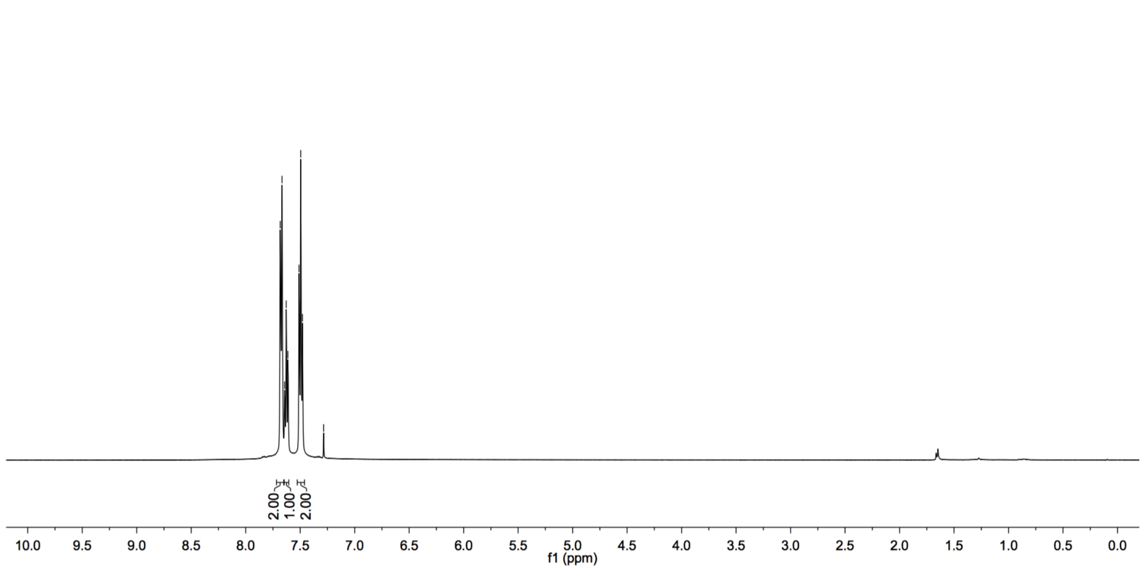
7.68
7.67
7.64
7.63
7.61
7.51
7.50
7.48
7.29
2.00
1.00
2.00
10.0 9.5 9.0 8.5 8.0 7.5 7.0 6.5 6.0 5.5 5.0 4.5 4.0 3.5 3.0 2.5 2.0 1.5 1.0 0.5 0.0
f1 (ppm)

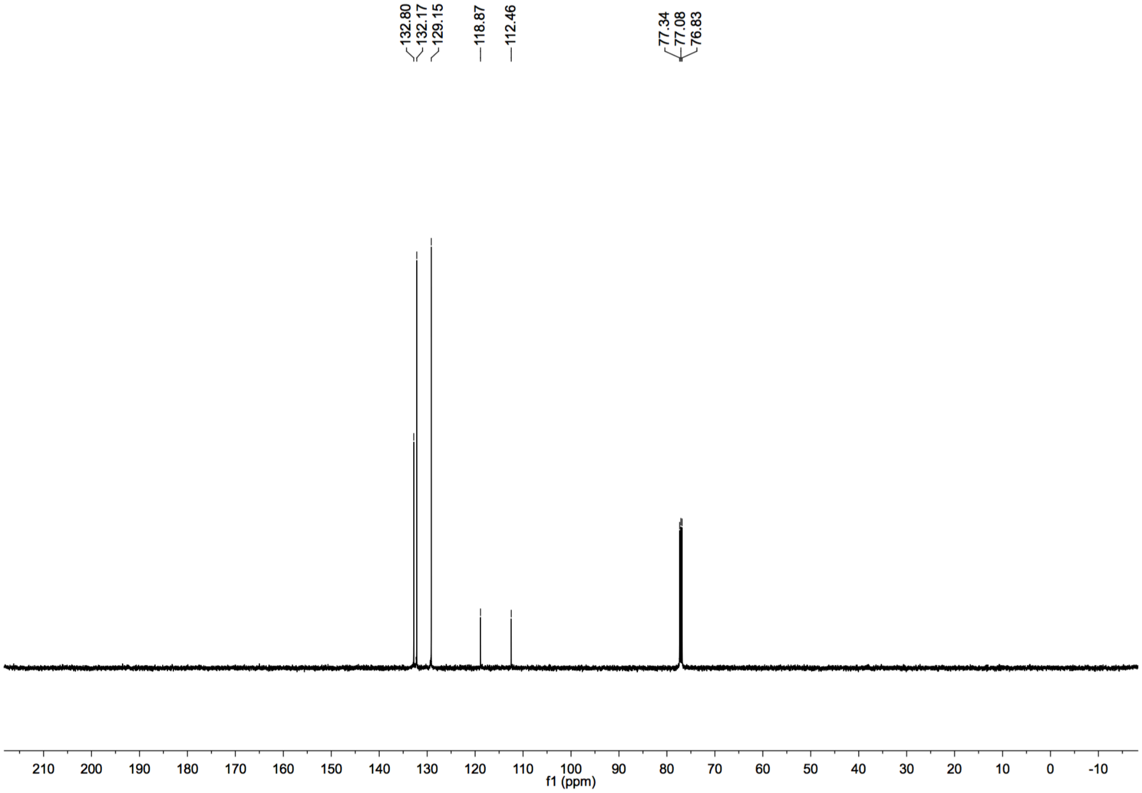
132.80
132.17
129.15
118.87
112.46
77.34
77.08
76.83
210 200 190 180 170 160 150 140 130 120 110 100 90 80 70 60 50 40 30 20 10 0 -10
f1 (ppm)

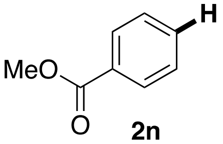

2n

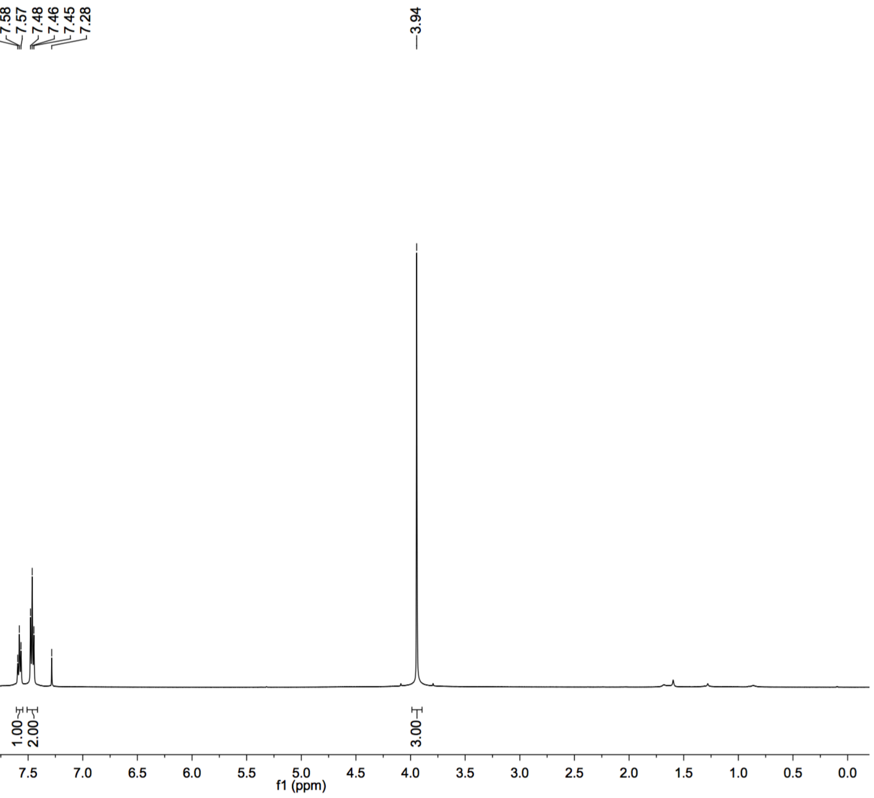

8.08
8.06
7.59
7.58
7.57
7.48
7.46
7.45
7.28
3.94
2.00
1.00
2.00
3.00
10.0
9.5
9.0
8.5
8.0
7.5
7.0
6.5
6.0
5.5
5.0
4.5
4.0
3.5
3.0
2.5
2.0
1.5
1.0
0.5
0.0
f1 (ppm)


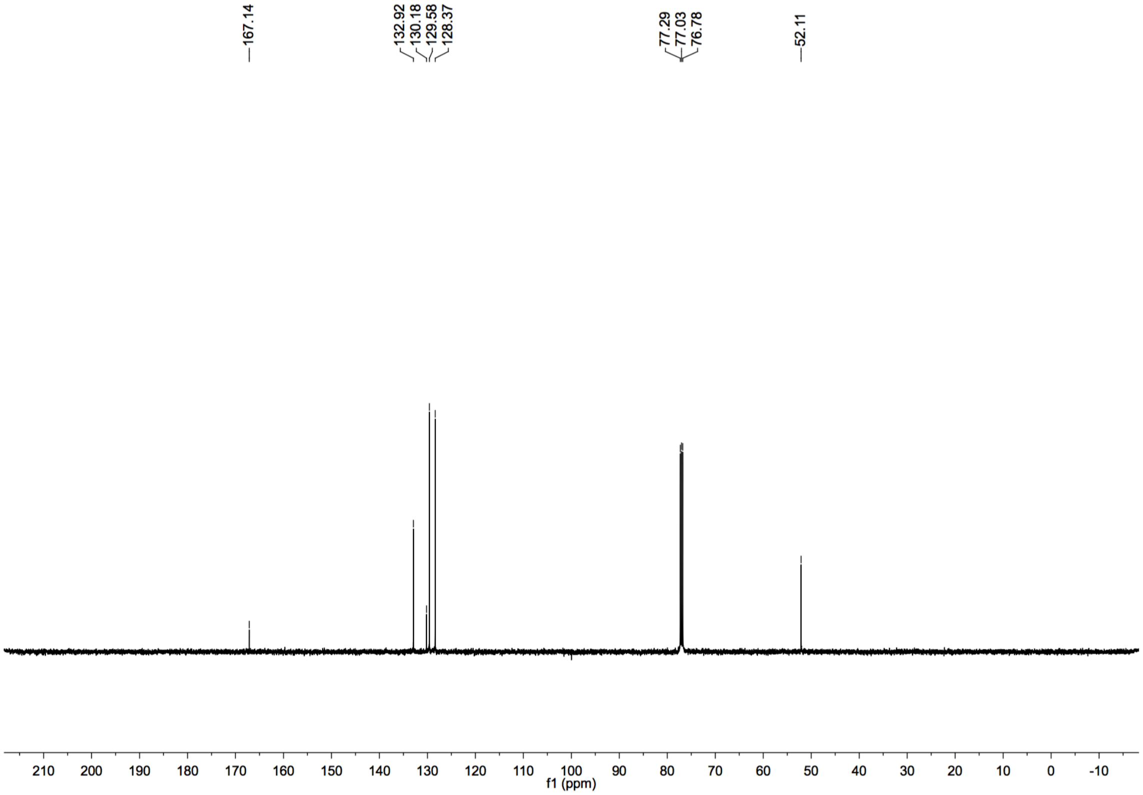

167.14
132.92
130.18
129.58
128.37
77.29
77.03
76.78
52.11
210
200
190
180
170
160
150
140
130
120
110
100
90
80
70
60
50
40
30
20
10
0
-10
f1 (ppm)

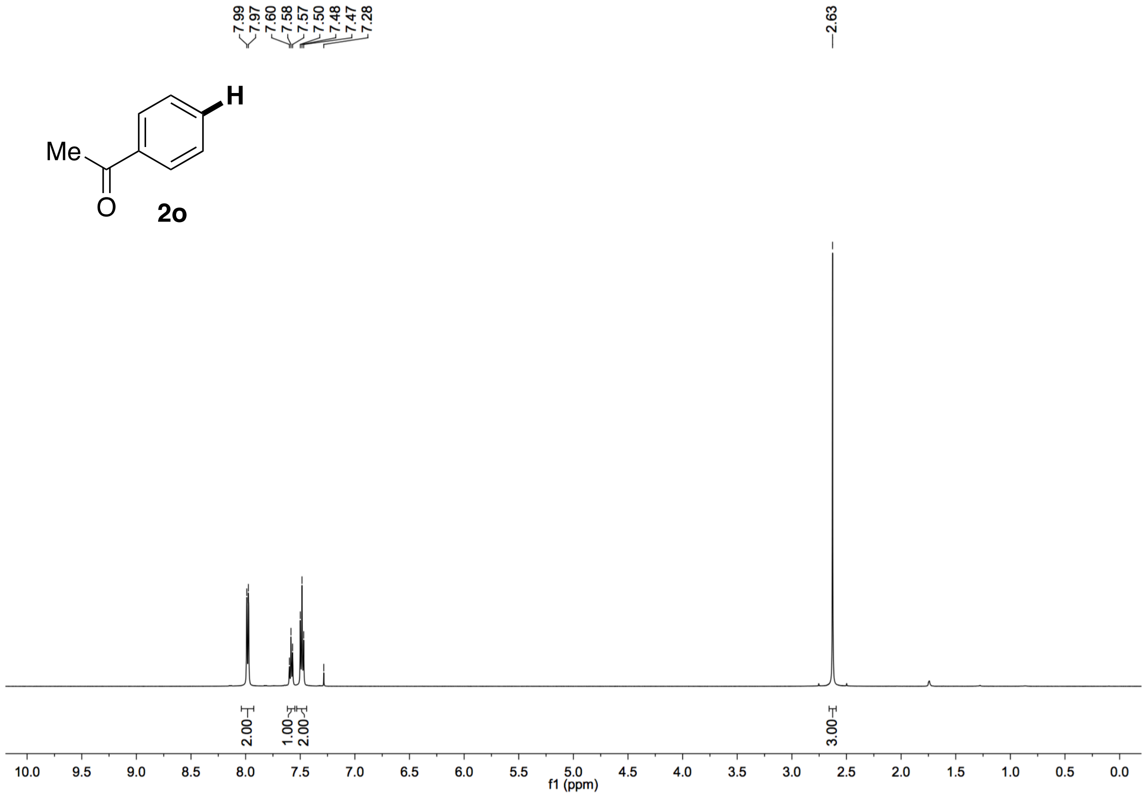
7.99
7.97
7.60
7.58
7.57
7.50
7.48
7.47
7.28
2.63
Me
O
H
2o
2.00
1.00
2.00
3.00
10.0 9.5 9.0 8.5 8.0 7.5 7.0 6.5 6.0 5.5 5.0 4.5 4.0 3.5 3.0 2.5 2.0 1.5 1.0 0.5 0.0
f1 (ppm)

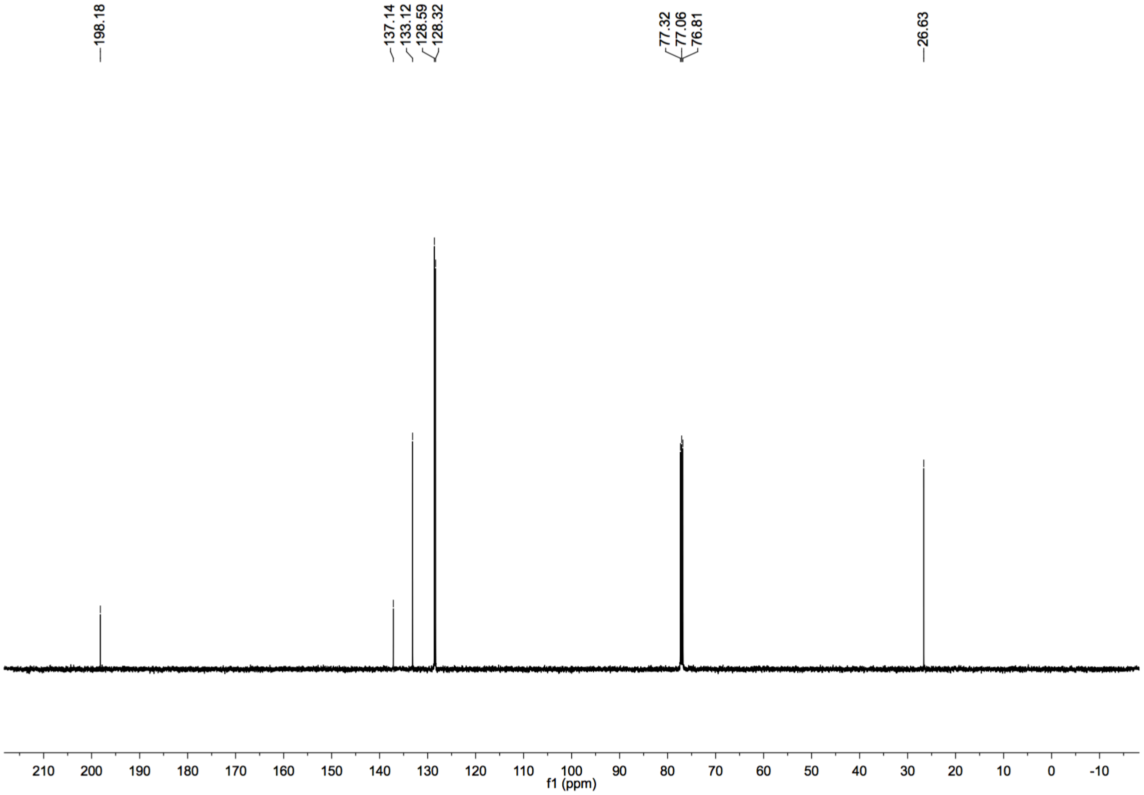
198.18
137.14
133.12
128.59
128.32
77.32
77.06
76.81
26.63
210 200 190 180 170 160 150 140 130 120 110 100 90 80 70 60 50 40 30 20 10 0 -10
f1 (ppm)

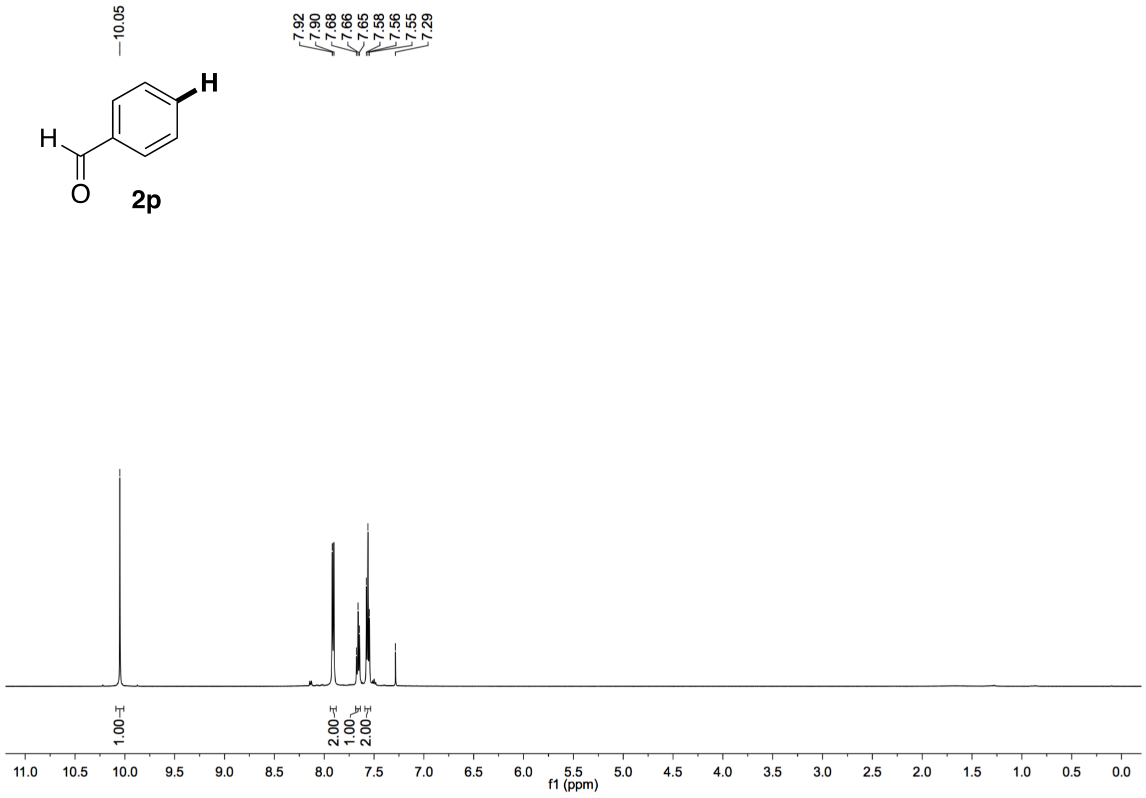
10.05
7.92
7.90
7.68
7.66
7.65
7.58
7.56
7.55
7.29
H
2p
1.00
2.00
1.00
2.00
f1 (ppm)

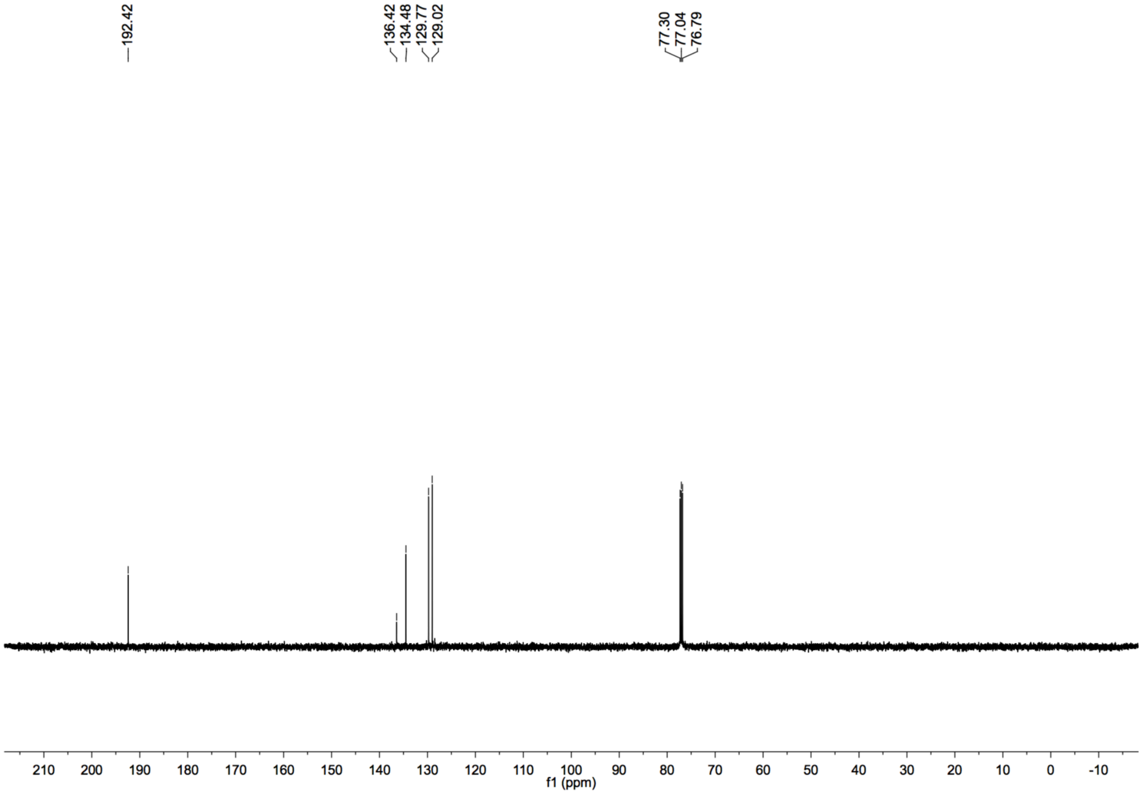
192.42
136.42
134.48
129.77
129.02
77.30
77.04
76.79
f1 (ppm)

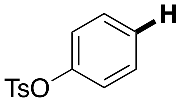
H
TsO

**2q**

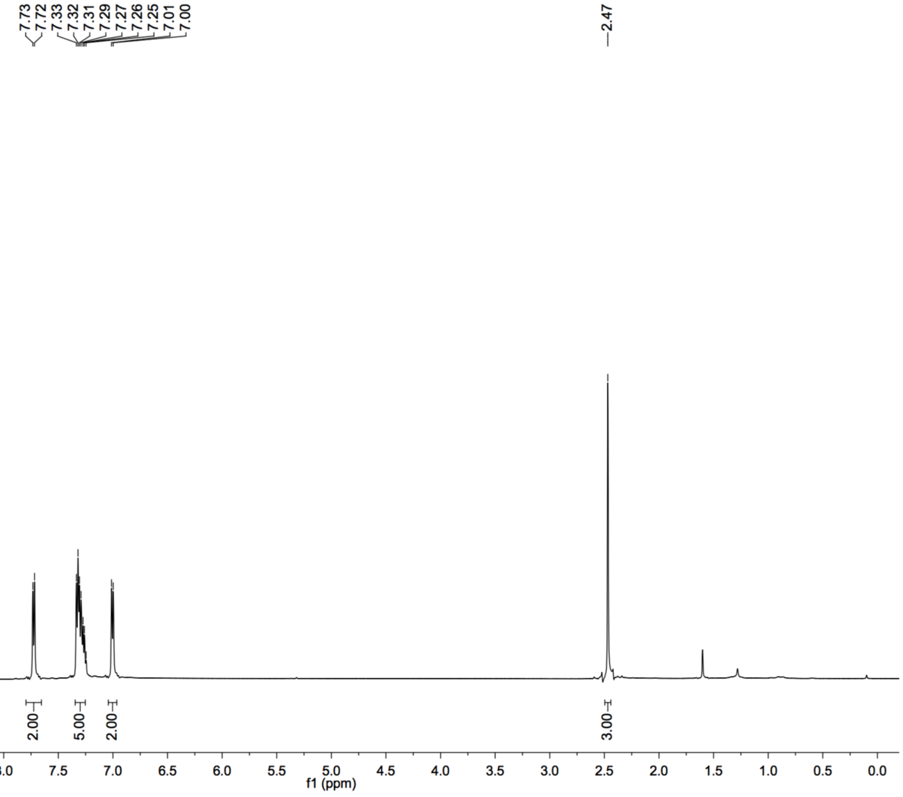
7.73
7.72
7.33
7.32
7.31
7.29
7.27
7.26
7.25
7.01
7.00
2.47
2.00
5.00
2.00
3.00
10.0 9.5 9.0 8.5 8.0 7.5 7.0 6.5 6.0 5.5 5.0 4.5 4.0 3.5 3.0 2.5 2.0 1.5 1.0 0.5 0.0
f1 (ppm)

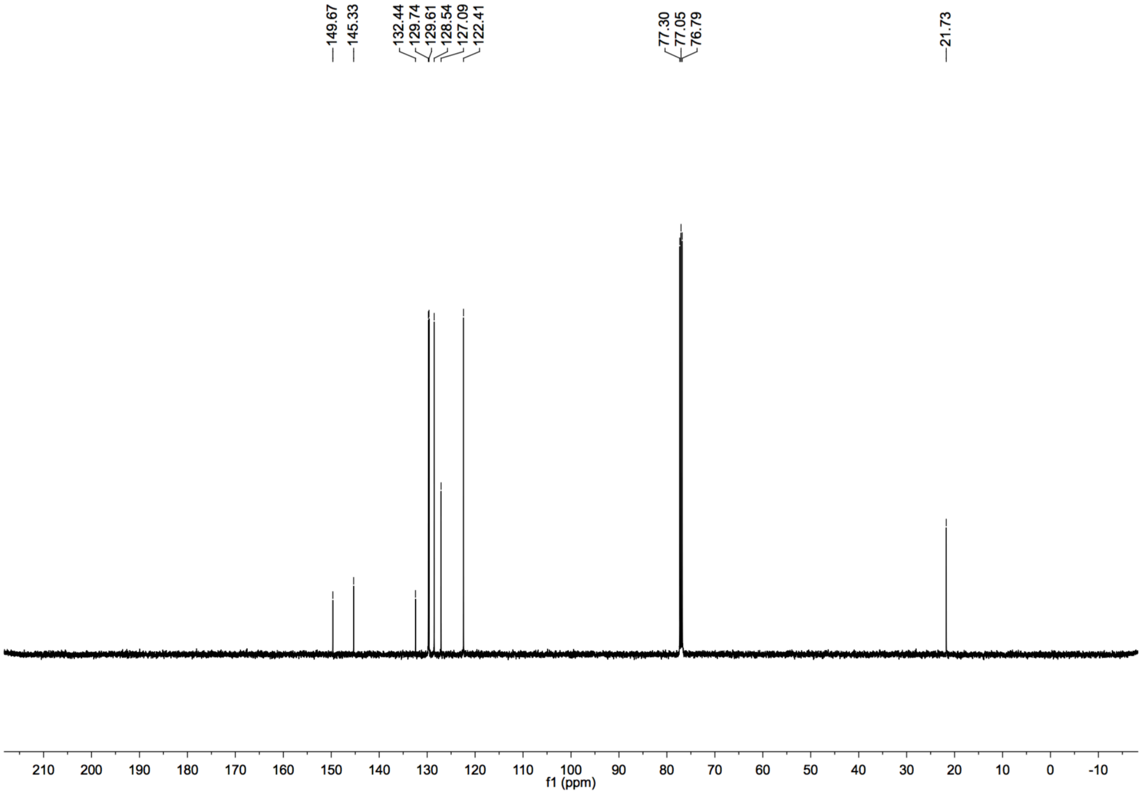
149.67
145.33
132.44
129.74
129.61
128.54
127.09
122.41
77.30
77.05
76.79
21.73
210 200 190 180 170 160 150 140 130 120 110 100 90 80 70 60 50 40 30 20 10 0 -10
f1 (ppm)

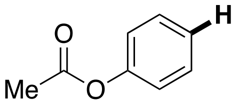

2r

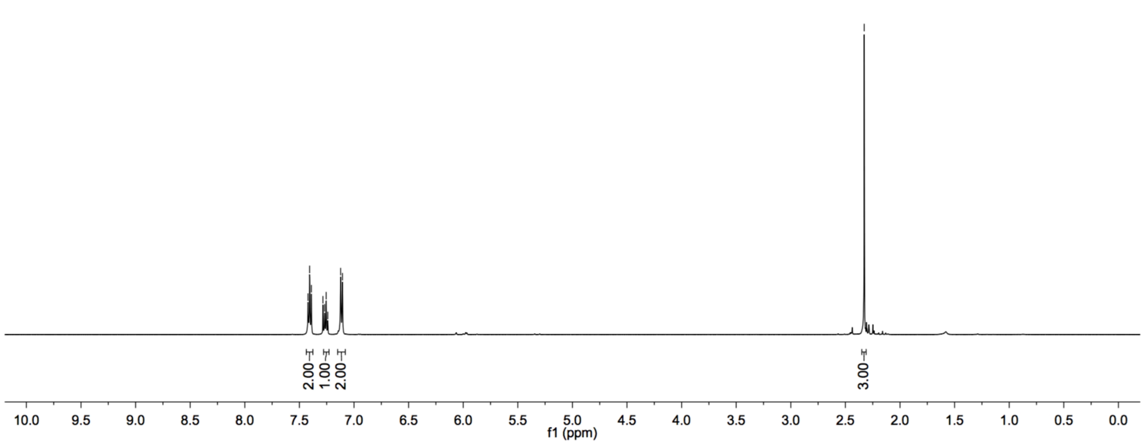
7.42
7.41
7.39
7.28
7.27
7.25
7.24
7.12
7.11
2.33
2.00
1.00
2.00
3.00
10.0 9.5 9.0 8.5 8.0 7.5 7.0 6.5 6.0 5.5 5.0 4.5 4.0 3.5 3.0 2.5 2.0 1.5 1.0 0.5 0.0
f1 (ppm)

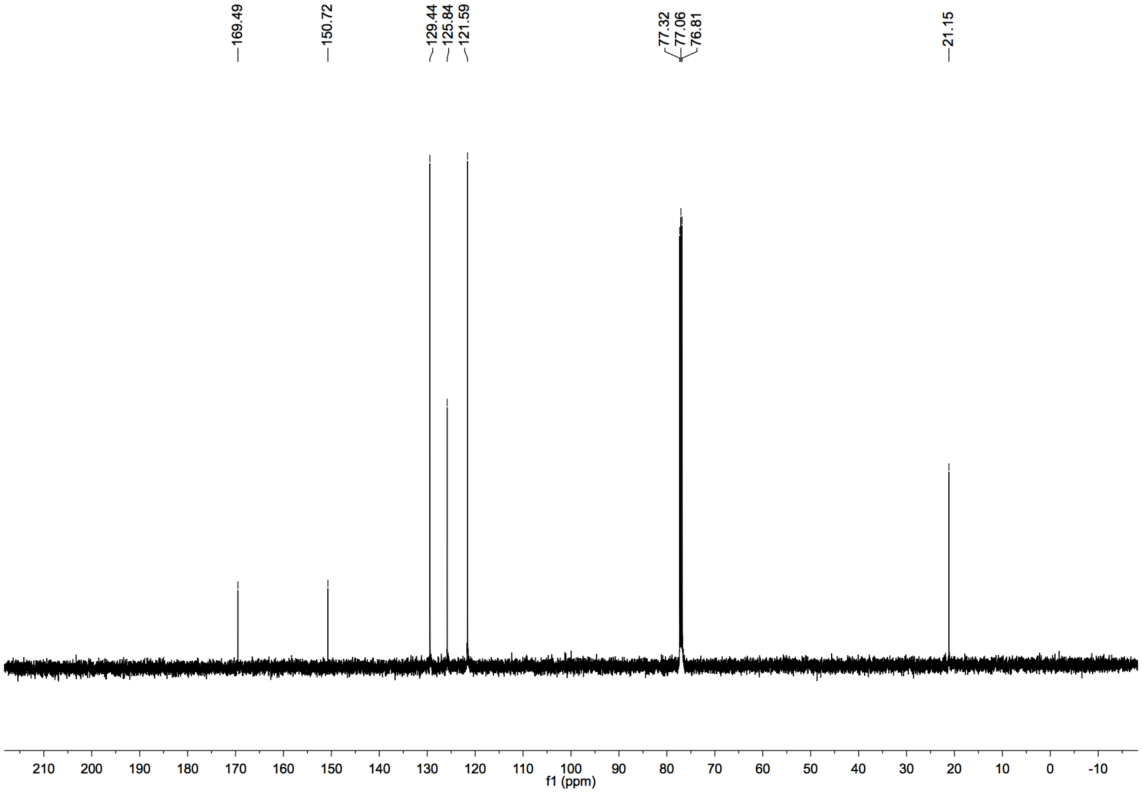
169.49
150.72
129.44
125.84
121.59
77.32
77.06
76.81
21.15
210 200 190 180 170 160 150 140 130 120 110 100 90 80 70 60 50 40 30 20 10 0 -10
f1 (ppm)

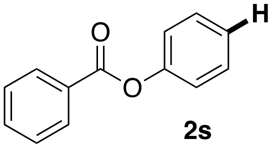

O
O
H


**2s**

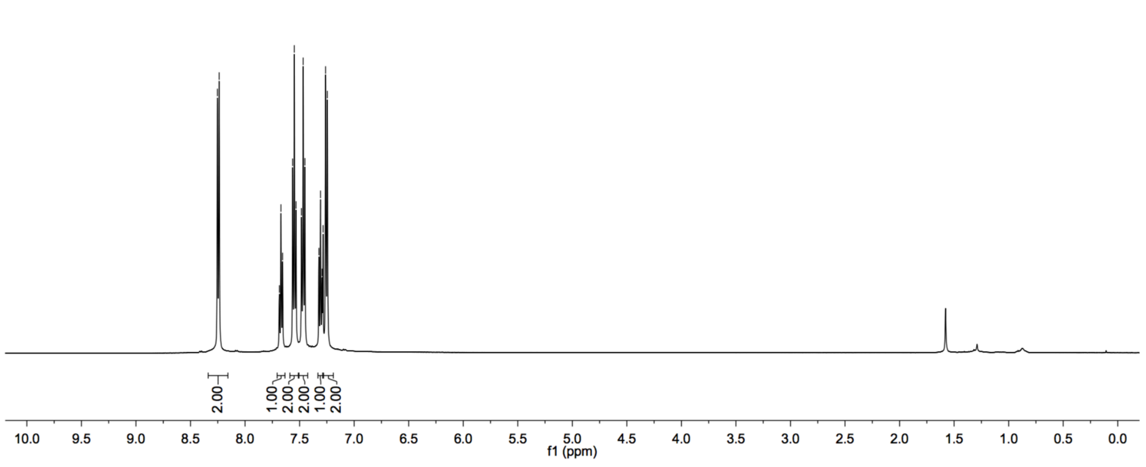

8.25
8.24
7.69
7.67
7.66
7.56
7.55
7.53
7.48
7.47
7.45
7.32
7.31
7.29
7.28
7.26
7.25
2.00
1.00
2.00
2.00
1.00
2.00
10.0 9.5 9.0 8.5 8.0 7.5 7.0 6.5 6.0 5.5 5.0 4.5 4.0 3.5 3.0 2.5 2.0 1.5 1.0 0.5 0.0
f1 (ppm)


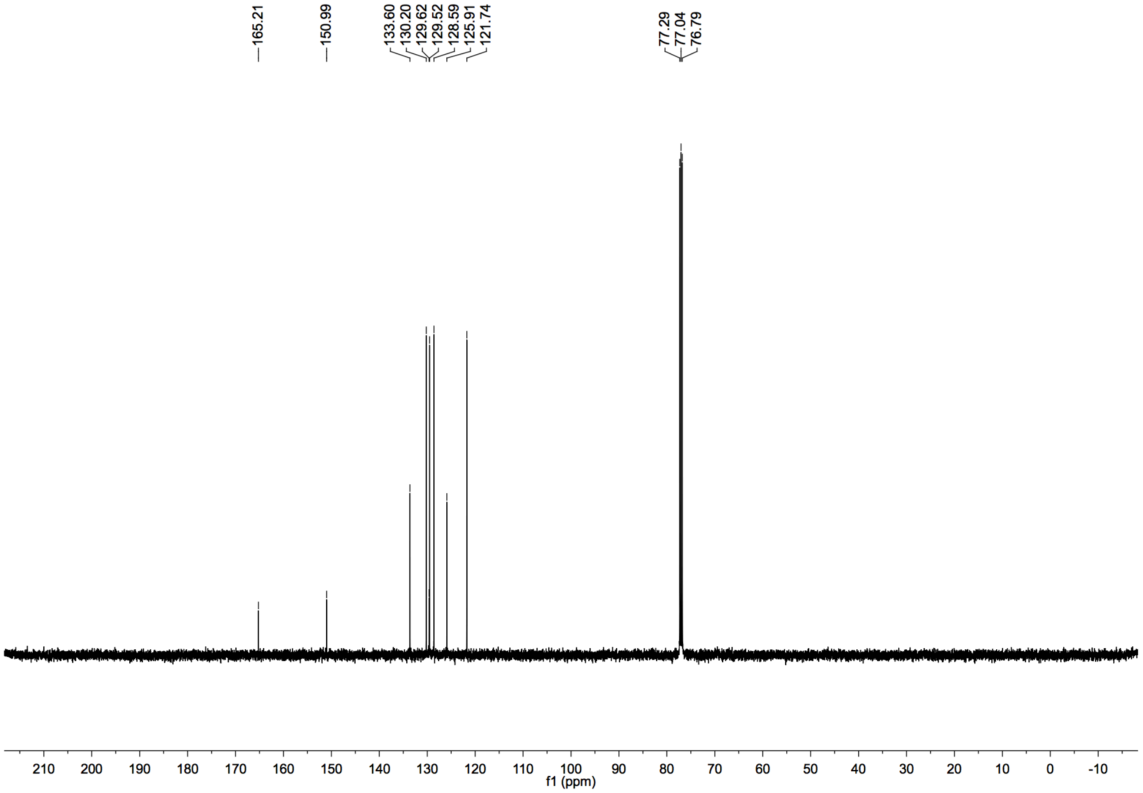

165.21
150.99
133.60
130.20
129.62
129.52
128.59
125.91
121.74
77.29
77.04
76.79
210 200 190 180 170 160 150 140 130 120 110 100 90 80 70 60 50 40 30 20 10 0 -10
f1 (ppm)

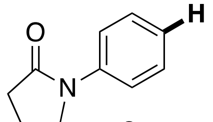
O
N
H

**2t**

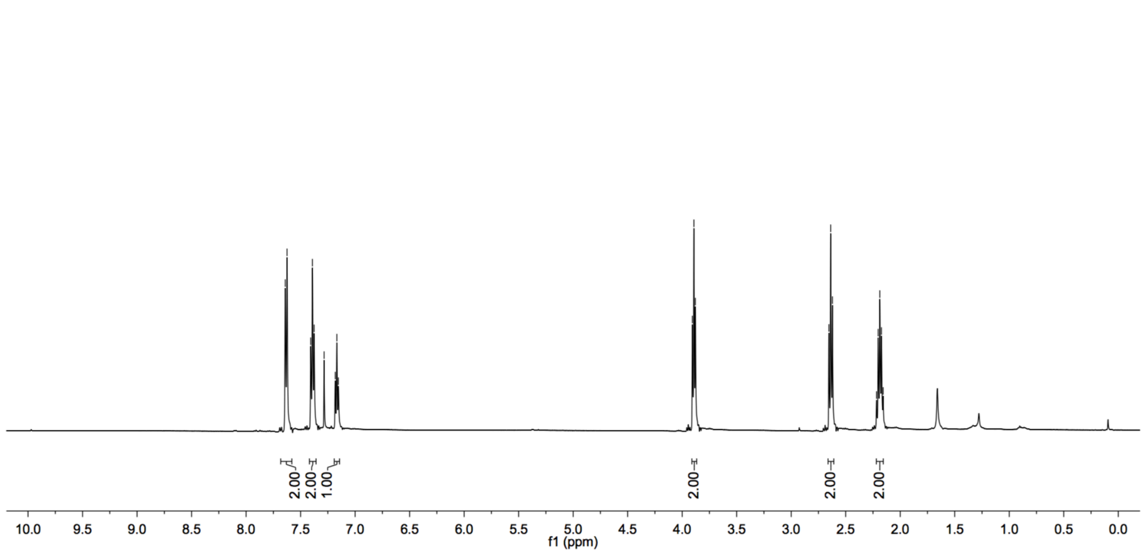
7.64
7.62
7.41
7.39
7.38
7.28
7.18
7.17
7.15
3.91
3.89
3.88
2.65
2.64
2.62
2.22
2.20
2.19
2.17
2.16
2.00
2.00
1.00
2.00
2.00
2.00
f1 (ppm)

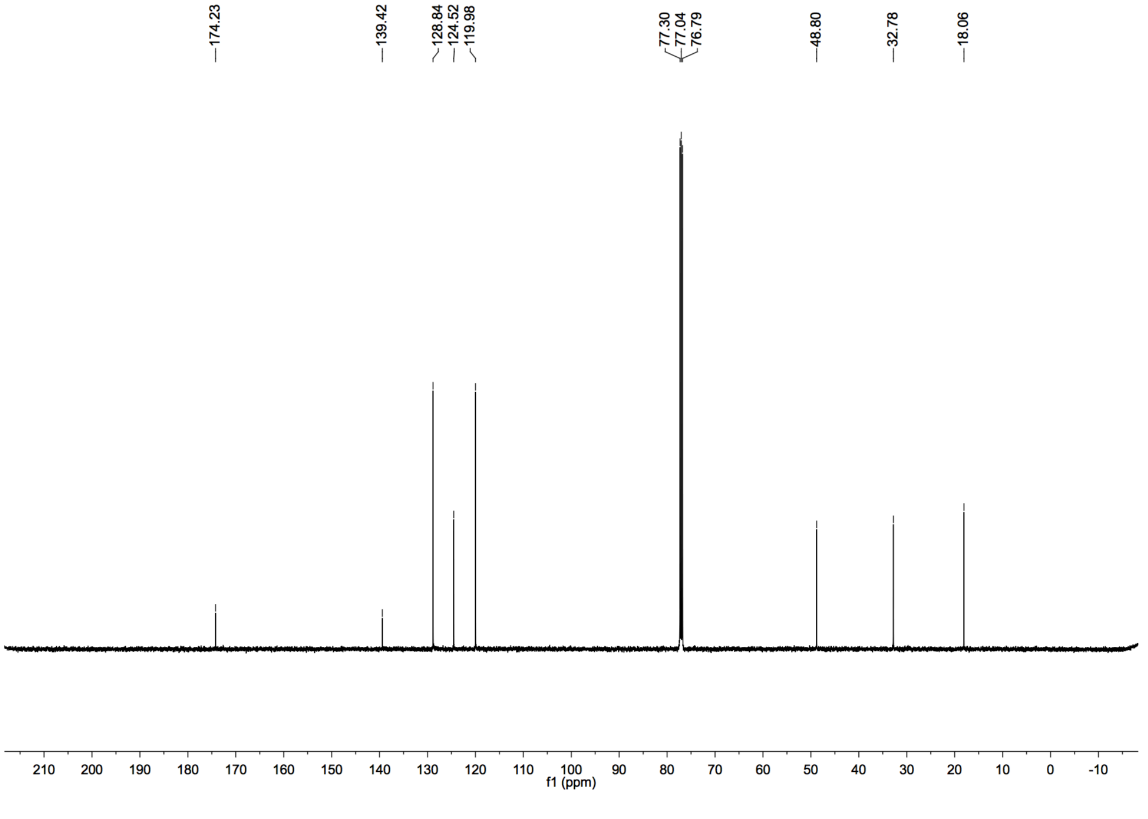
174.23
139.42
128.84
124.52
119.98
77.30
77.04
76.79
48.80
32.78
18.06
f1 (ppm)

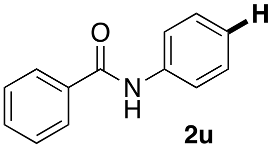

2u

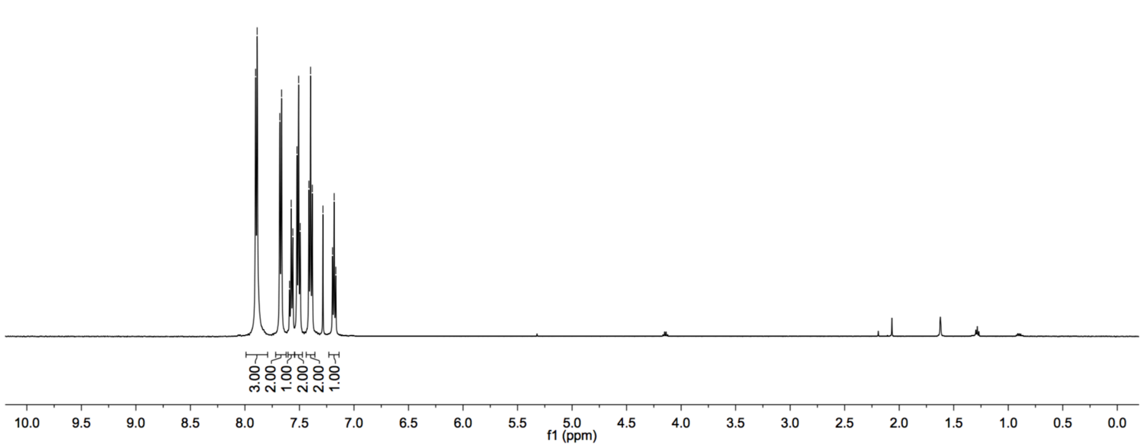

7.90
7.89
7.68
7.66
7.59
7.58
7.56
7.52
7.51
7.49
7.41
7.40
7.38
7.28
7.20
7.18
7.17
3.00
2.00
1.00
2.00
2.00
1.00
f1 (ppm)


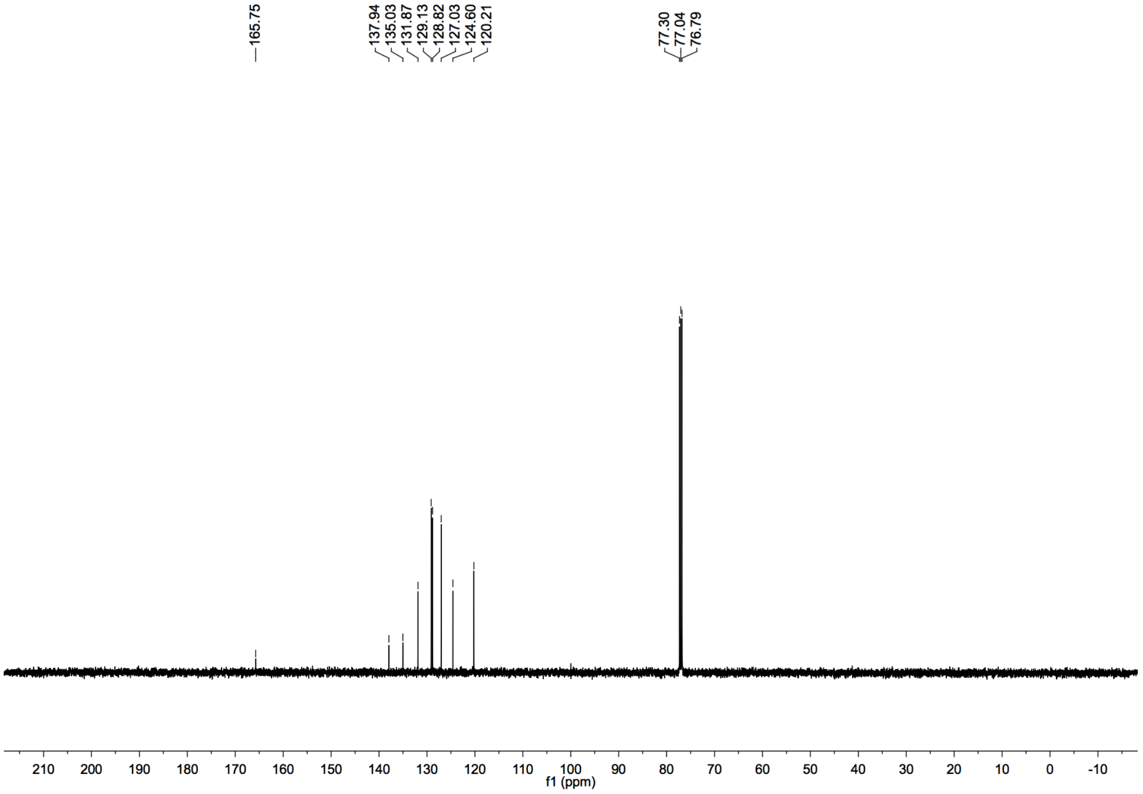

165.75
137.94
135.03
131.87
129.13
128.82
127.03
124.60
120.21
77.30
77.04
76.79
f1 (ppm)

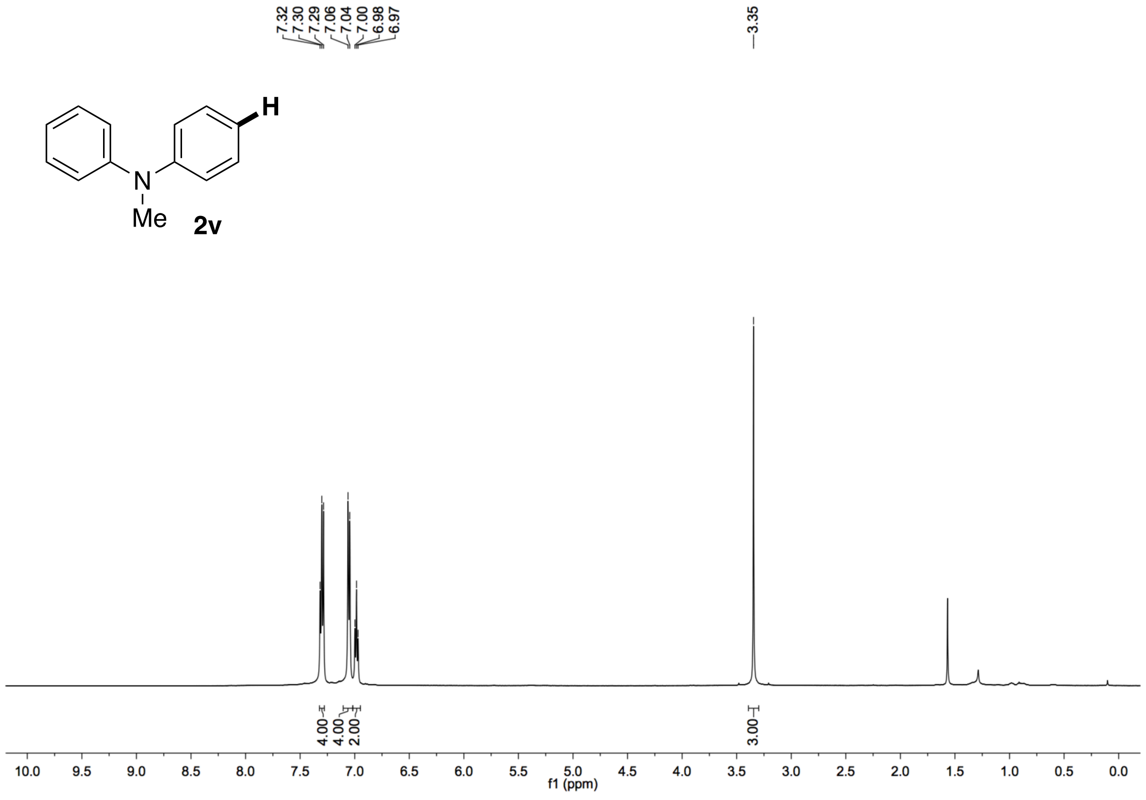
7.32
7.30
7.29
7.06
7.04
7.00
6.98
6.97
3.35
H
N
Me
2v
4.00
4.00
2.00
3.00
10.0 9.5 9.0 8.5 8.0 7.5 7.0 6.5 6.0 5.5 5.0 4.5 4.0 3.5 3.0 2.5 2.0 1.5 1.0 0.5 0.0
f1 (ppm)

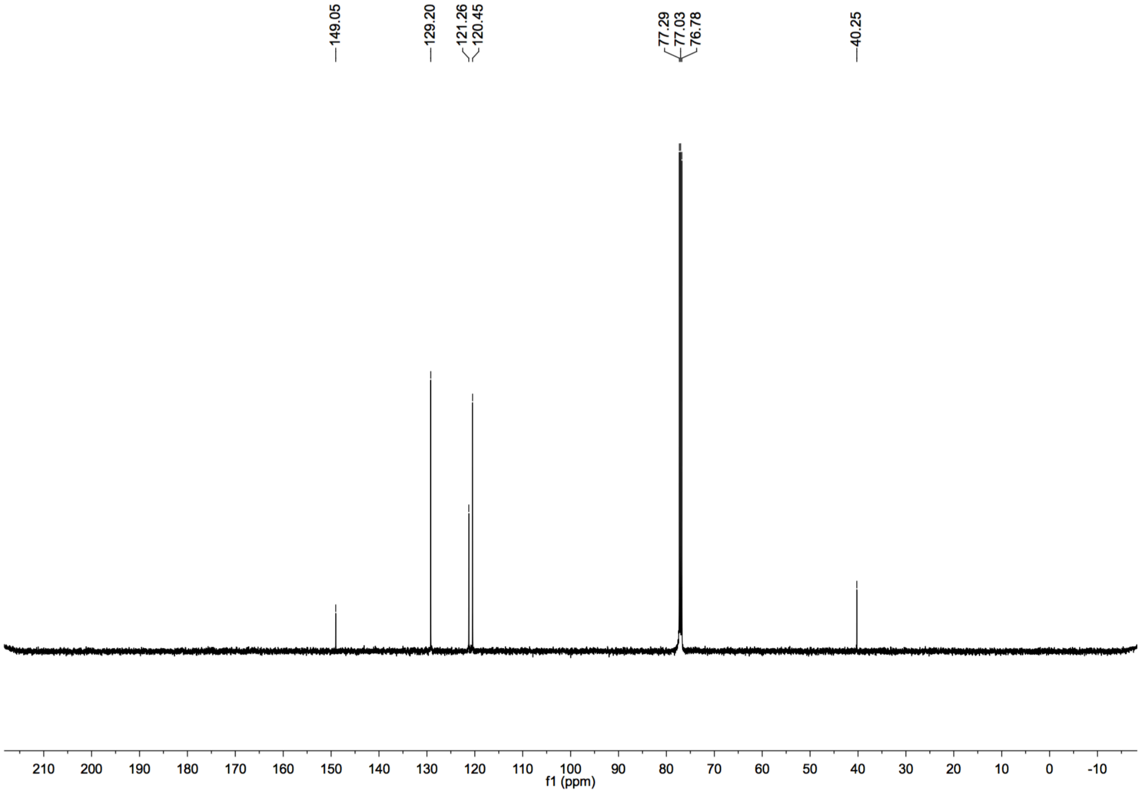
149.05
129.20
121.26
120.45
77.29
77.03
76.78
40.25
210 200 190 180 170 160 150 140 130 120 110 100 90 80 70 60 50 40 30 20 10 0 -10
f1 (ppm)

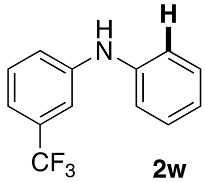

H
N
H
CF3


**2w**

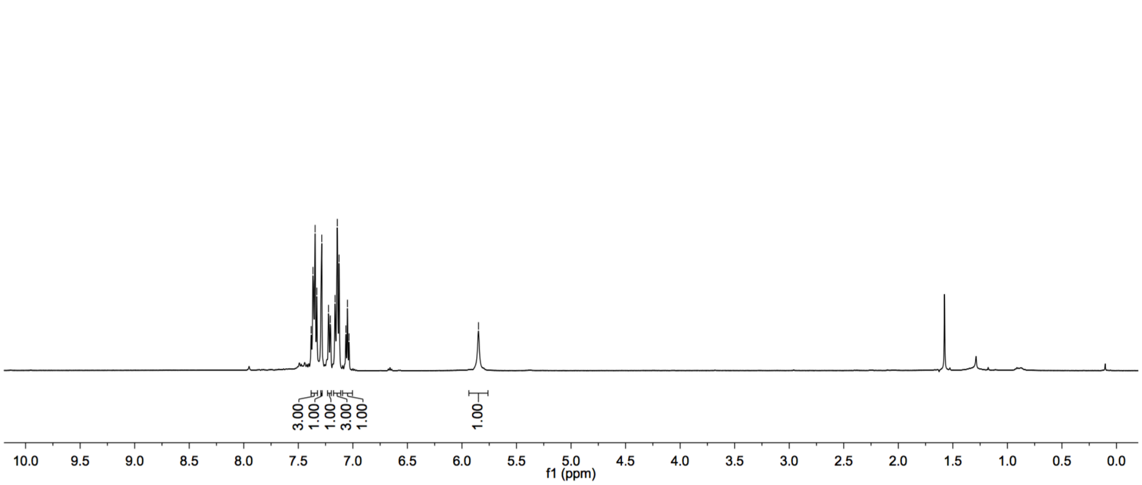

7.38
7.37
7.35
7.33
7.29
7.22
7.21
7.16
7.14
7.13
7.06
7.05
7.03
5.85
3.00
1.00
1.00
3.00
1.00
1.00
10.0 9.5 9.0 8.5 8.0 7.5 7.0 6.5 6.0 5.5 5.0 4.5 4.0 3.5 3.0 2.5 2.0 1.5 1.0 0.5 0.0
f1 (ppm)


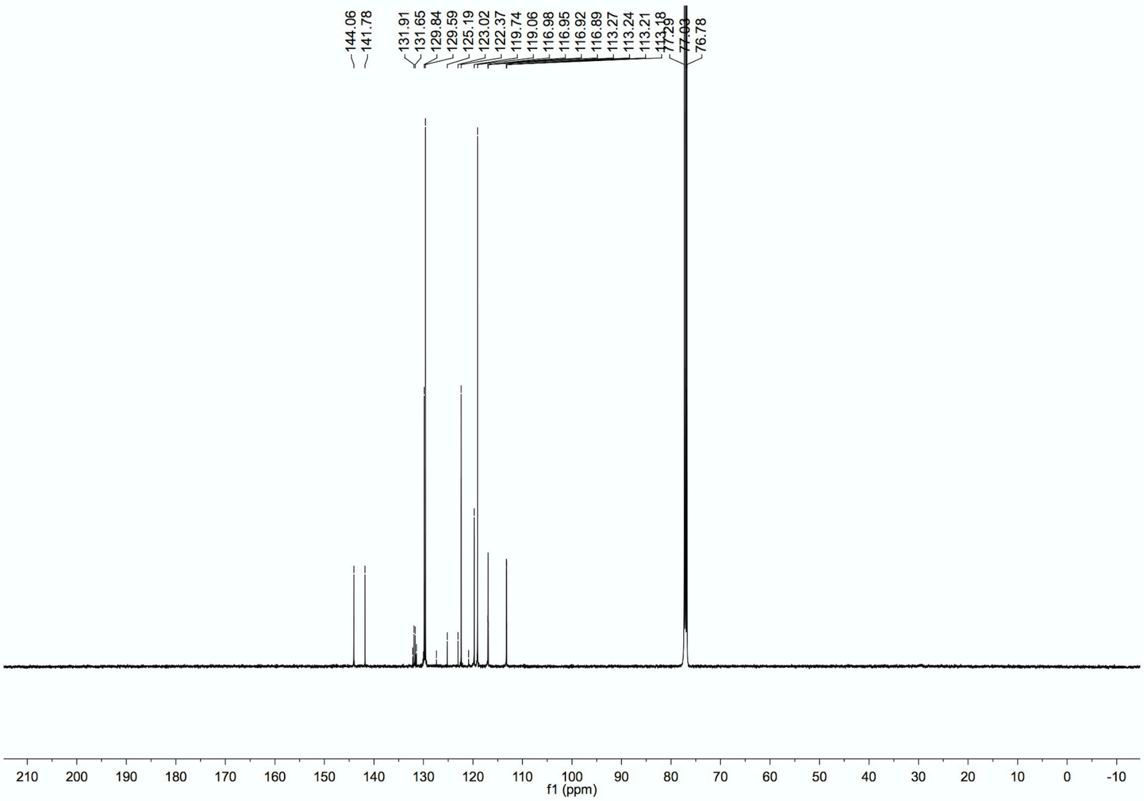

144.06
141.78
131.91
131.65
129.84
129.59
125.19
123.02
122.37
119.74
119.06
116.98
116.95
116.92
116.89
113.27
113.24
113.21
113.18
77.29
77.03
76.78
210 200 190 180 170 160 150 140 130 120 110 100 90 80 70 60 50 40 30 20 10 0 -10
f1 (ppm)

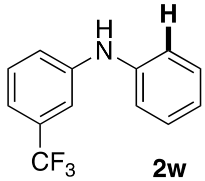

**2w**

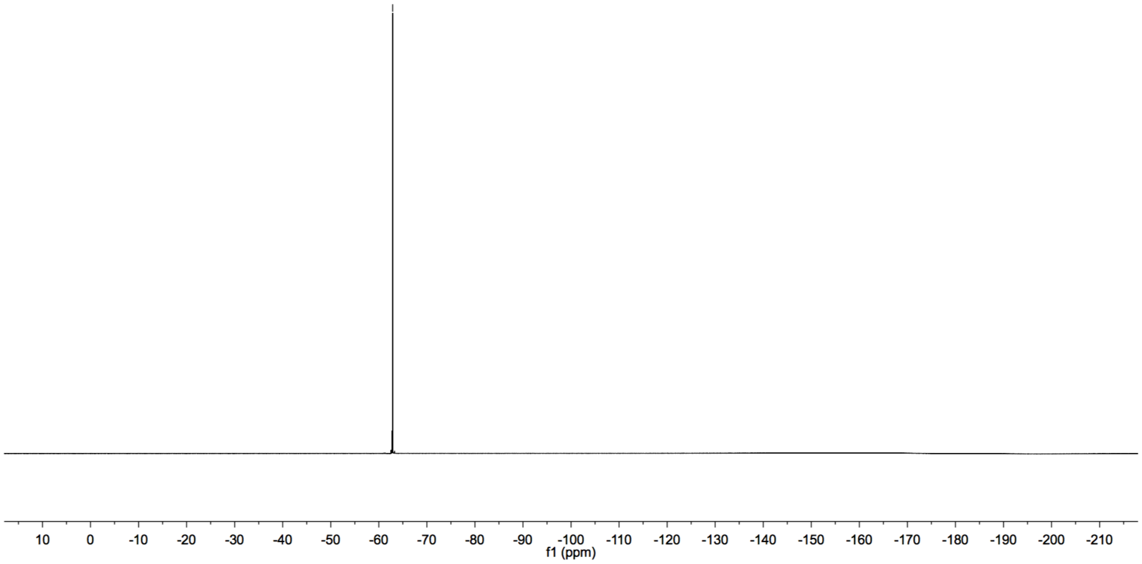

-62.87
10
0
-10
-20
-30
-40
-50
-60
-70
-80
-90
-100
-110
-120
-130
-140
-150
-160
-170
-180
-190
-200
-210
f1 (ppm)

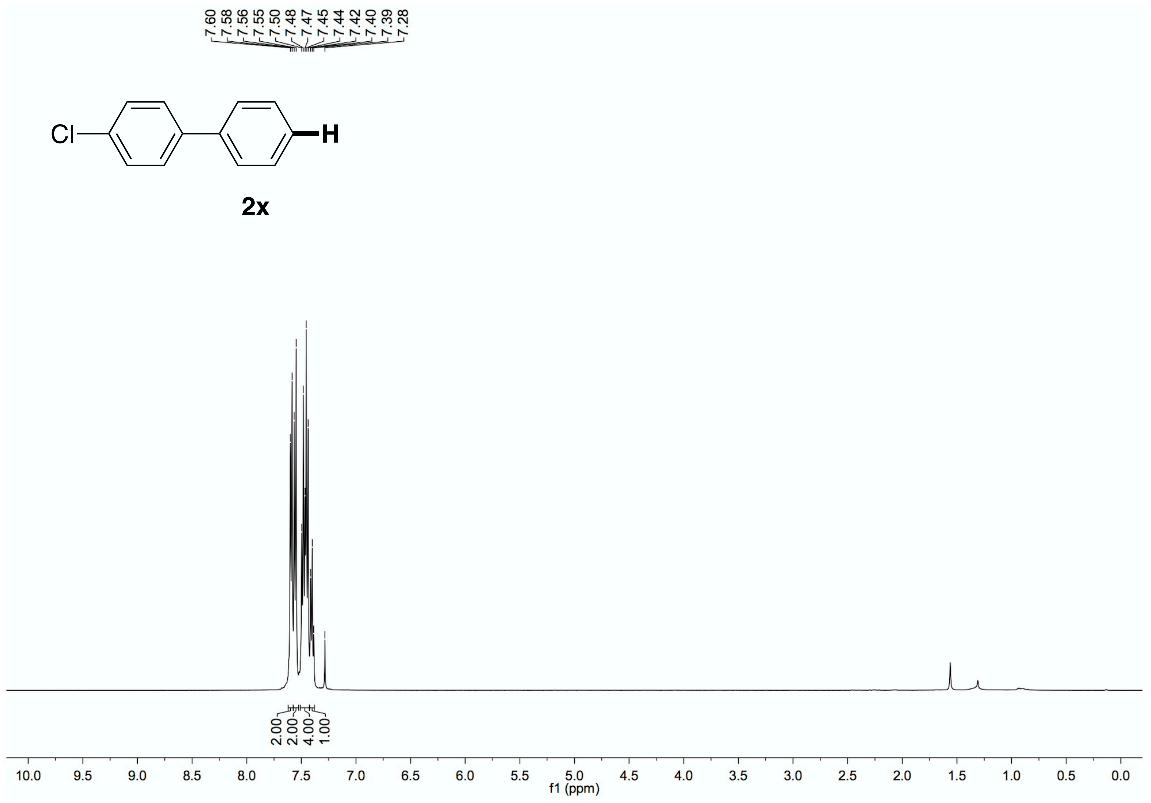
7.60
7.58
7.56
7.55
7.50
7.48
7.47
7.45
7.44
7.42
7.40
7.39
7.28
Cl
H
2x
2.00
2.00
4.00
1.00
f1 (ppm)

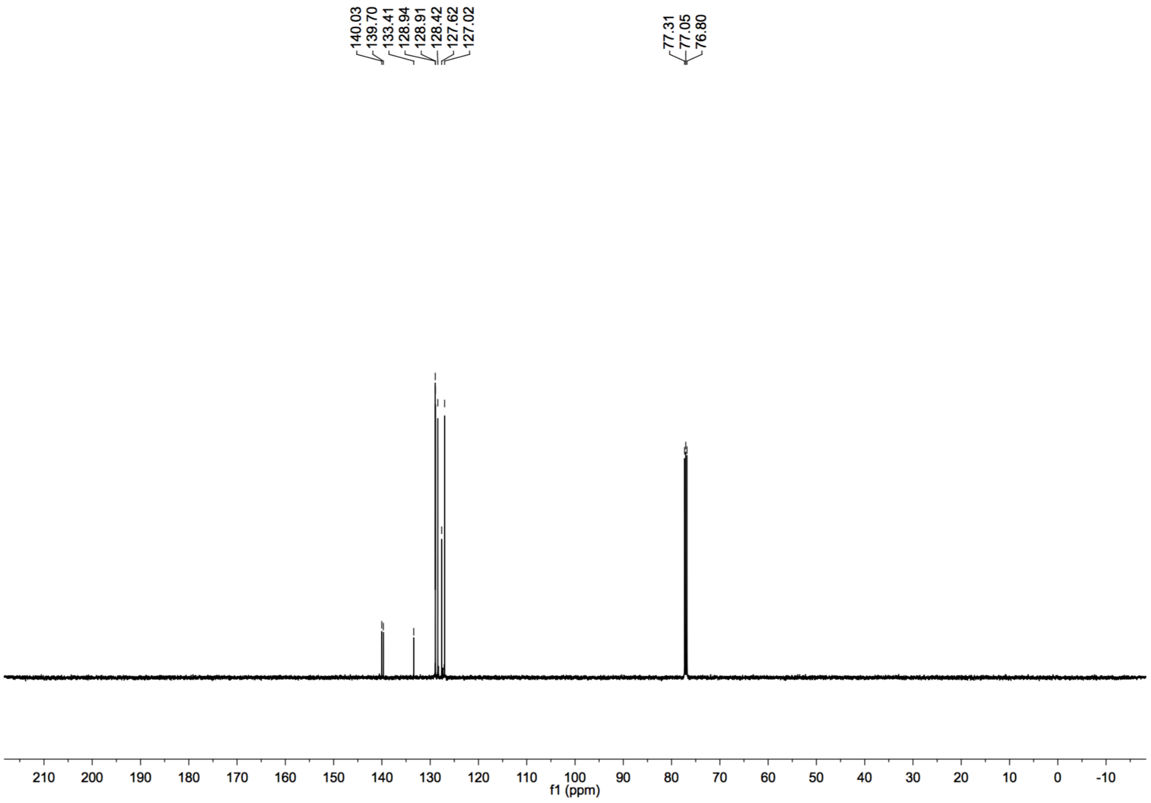
140.03
139.70
133.41
128.94
128.91
128.42
127.62
127.02
77.31
77.05
76.80
f1 (ppm)

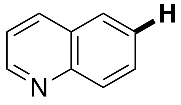
H
N

**2y**

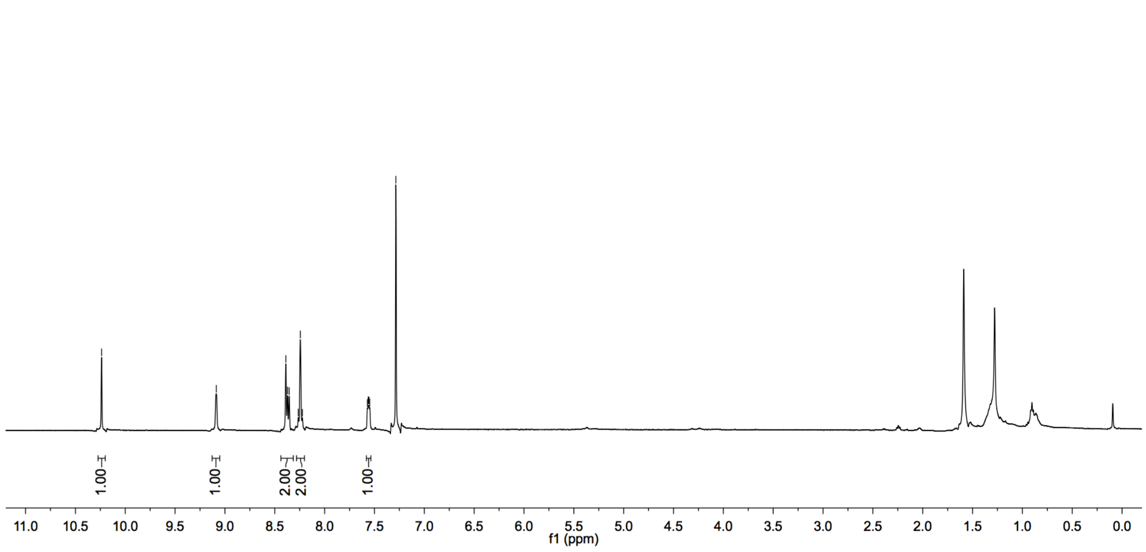
10.24
9.09
8.39
8.37
8.36
8.26
8.24
8.22
7.57
7.56
7.56
7.55
7.29
1.00
1.00
2.00
2.00
1.00
f1 (ppm)

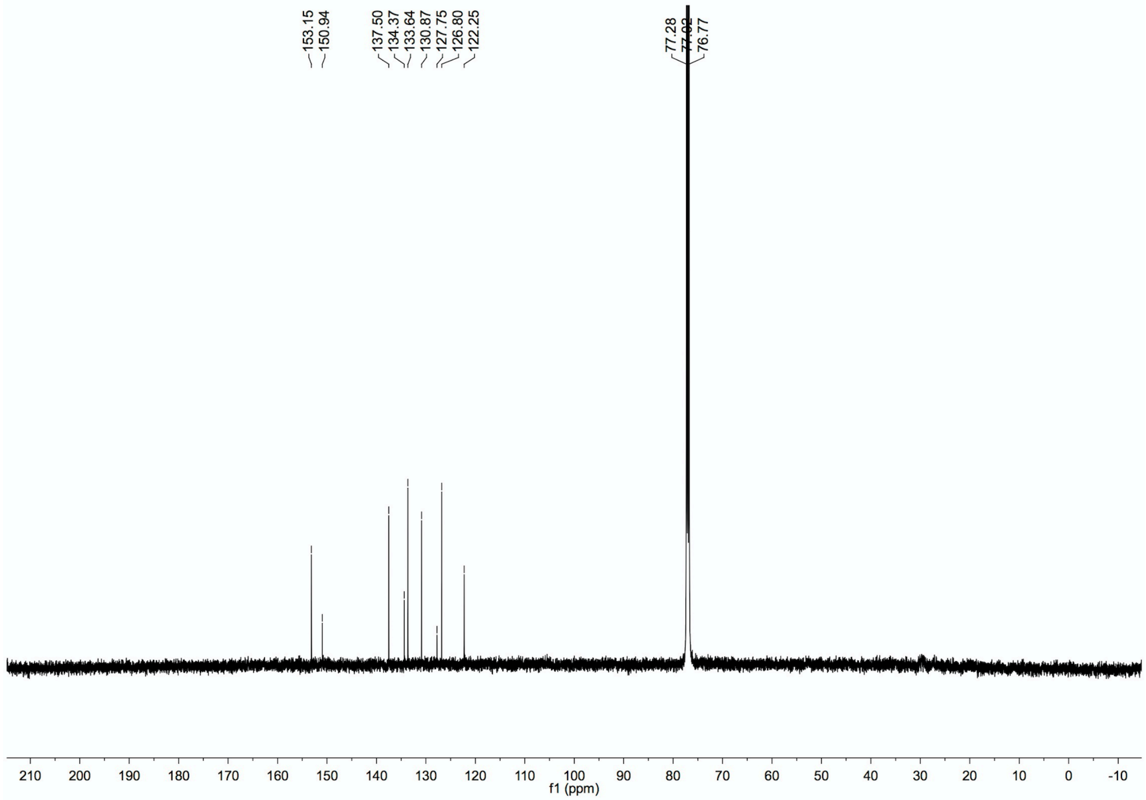
153.15
150.94
137.50
134.37
133.64
130.87
127.75
126.80
122.25
77.28
77.02
76.77
f1 (ppm)

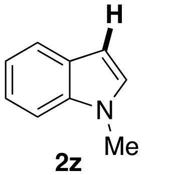
H
N
Me

**2z**

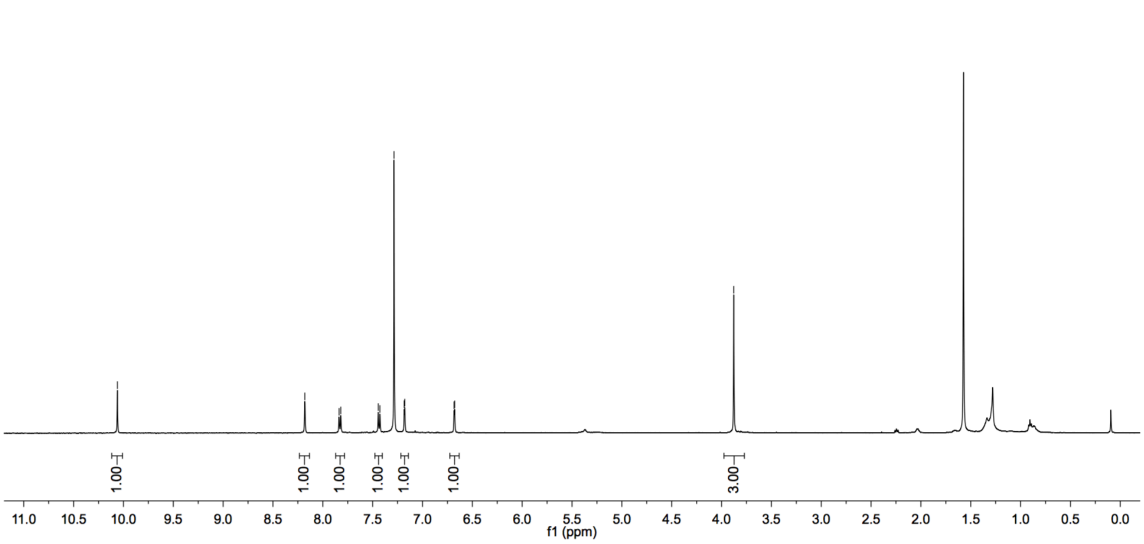
10.06
8.18
7.84
7.82
7.44
7.43
7.29
7.18
7.18
6.68
6.68
3.88
1.00
1.00
1.00
1.00
1.00
1.00
3.00
11.0 10.5 10.0 9.5 9.0 8.5 8.0 7.5 7.0 6.5 6.0 5.5 5.0 4.5 4.0 3.5 3.0 2.5 2.0 1.5 1.0 0.5 0.0
f1 (ppm)

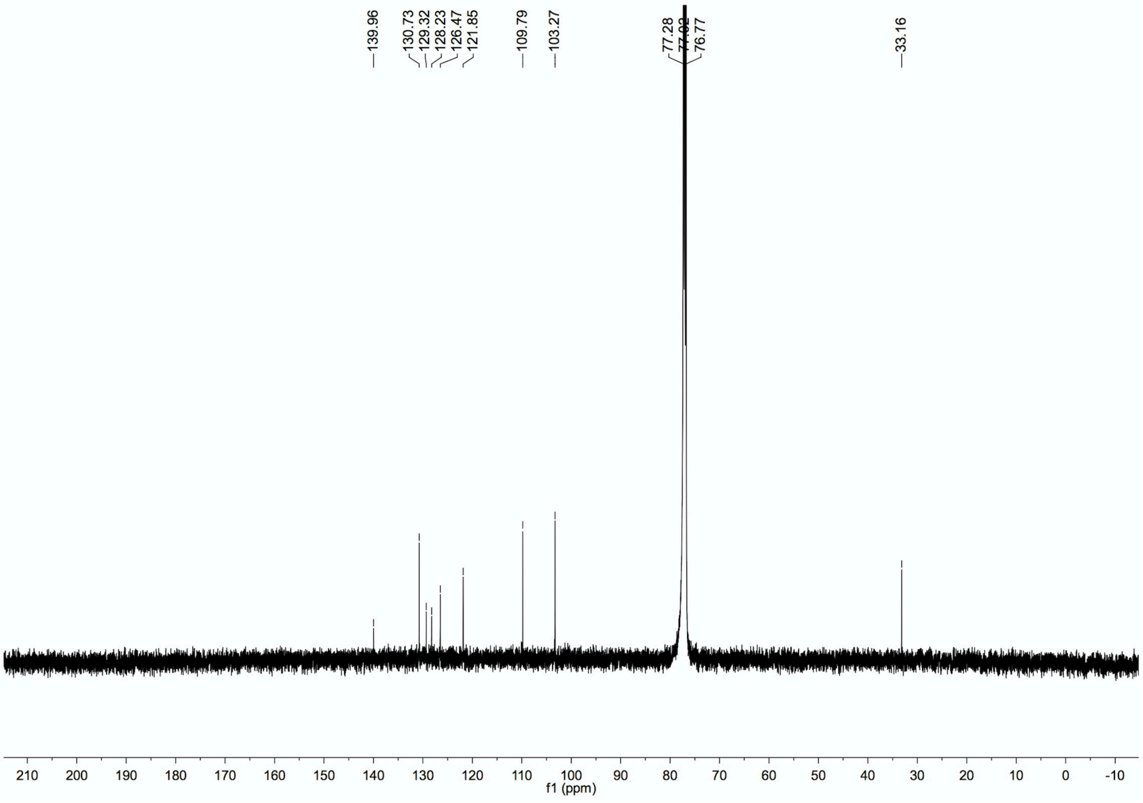
139.96
130.73
129.32
128.23
126.47
121.85
109.79
103.27
77.28
76.77
33.16
210 200 190 180 170 160 150 140 130 120 110 100 90 80 70 60 50 40 30 20 10 0 -10
f1 (ppm)

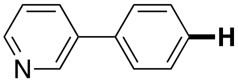
N
H

**2aa**

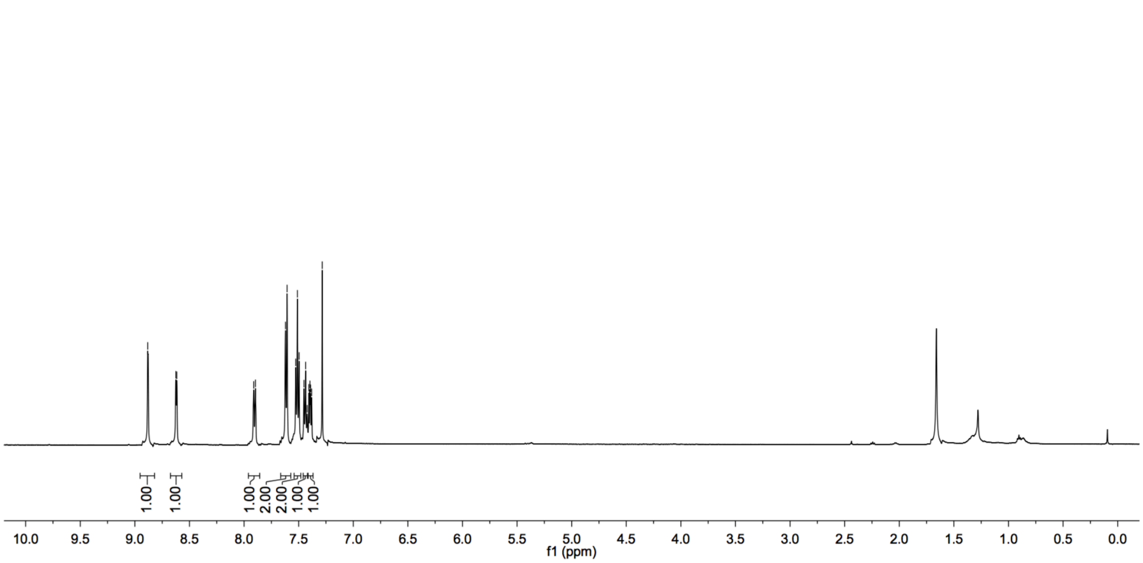
8.88
8.63
8.62
7.91
7.90
7.62
7.61
7.53
7.51
7.50
7.45
7.44
7.42
7.41
7.40
7.39
7.38
7.28
1.00
1.00
1.00
2.00
2.00
1.00
1.00
10.0 9.5 9.0 8.5 8.0 7.5 7.0 6.5 6.0 5.5 5.0 4.5 4.0 3.5 3.0 2.5 2.0 1.5 1.0 0.5 0.0
f1 (ppm)

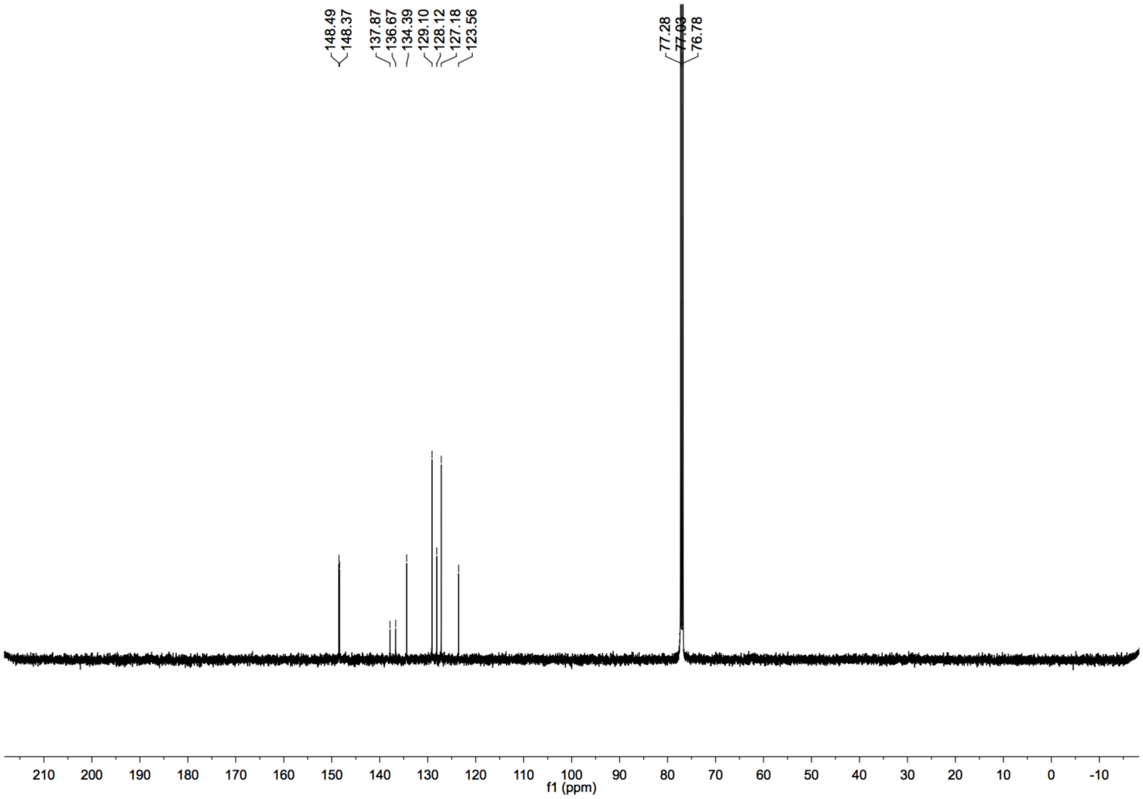
148.49
148.37
137.87
136.67
134.39
129.10
128.12
127.18
123.56
77.28
77.03
76.78
210 200 190 180 170 160 150 140 130 120 110 100 90 80 70 60 50 40 30 20 10 0 -10
f1 (ppm)

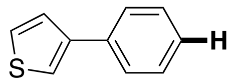

**2ab**

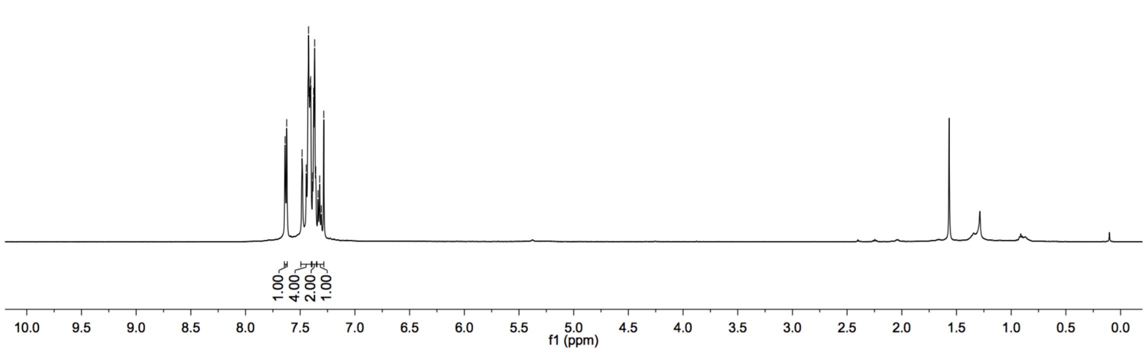

7.64
7.62
7.48
7.44
7.42
7.42
7.41
7.41
7.41
7.40
7.39
7.38
7.37
7.36
7.34
7.32
7.31
7.28
1.00
4.00
2.00
1.00
f1 (ppm)


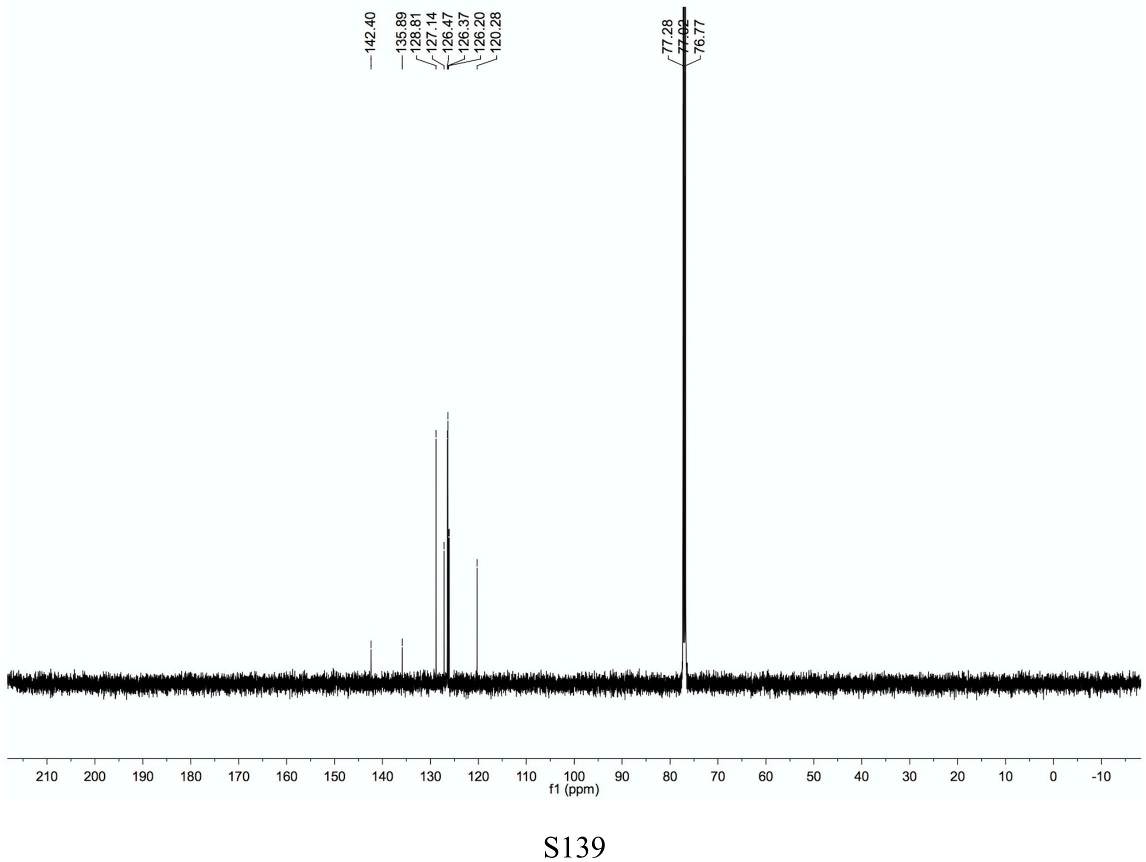

142.40
135.89
128.81
127.14
126.47
126.37
126.20
120.28
77.28
77.02
76.77
f1 (ppm)

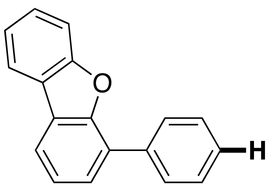

**2ac**

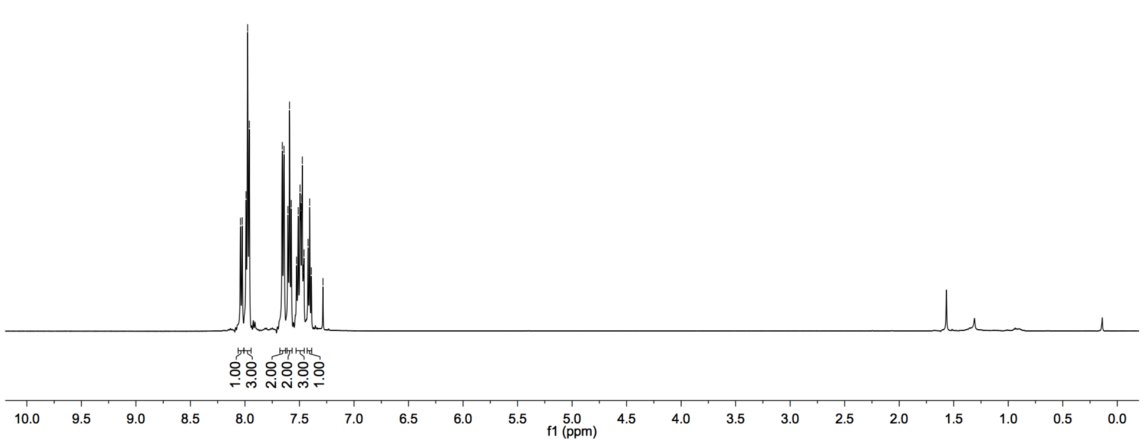
8.04
8.03
7.99
7.97
7.96
7.66
7.64
7.61
7.59
7.58
7.53
7.51
7.49
7.49
7.48
7.47
7.46
7.46
7.42
7.41
7.39
7.28
1.00
3.00
2.00
2.00
3.00
1.00
10.0 9.5 9.0 8.5 8.0 7.5 7.0 6.5 6.0 5.5 5.0 4.5 4.0 3.5 3.0 2.5 2.0 1.5 1.0 0.5 0.0
f1 (ppm)

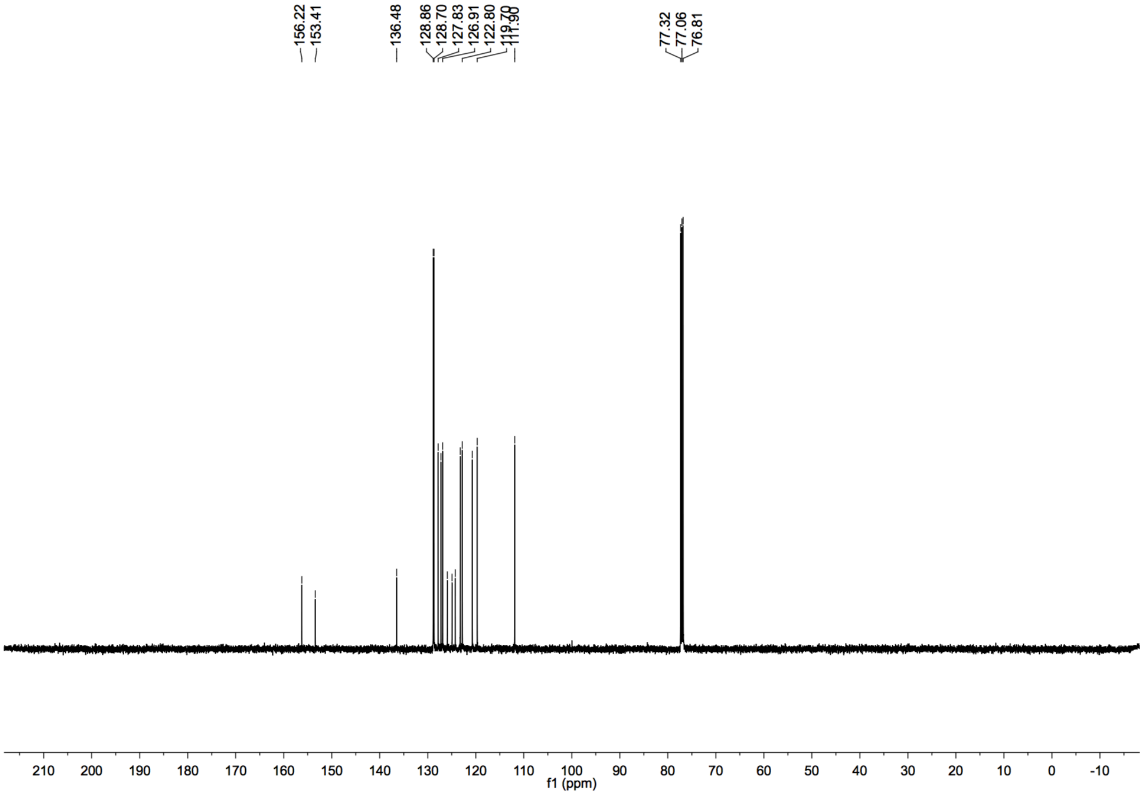
156.22
153.41
136.48
128.86
128.70
127.83
126.91
122.80
119.70
111.90
77.32
77.06
76.81
210 200 190 180 170 160 150 140 130 120 110 100 90 80 70 60 50 40 30 20 10 0 -10
f1 (ppm)

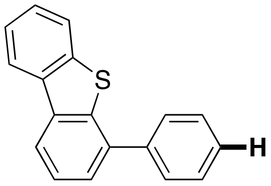
S
H

**2ad**

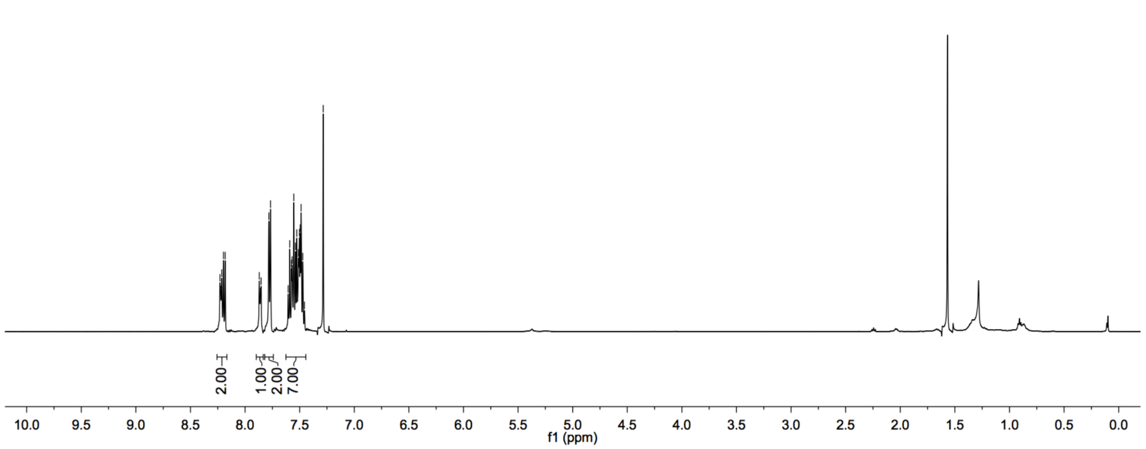
8.23
8.21
8.20
8.18
7.87
7.85
7.78
7.77
7.61
7.59
7.57
7.57
7.55
7.54
7.53
7.51
7.50
7.49
7.49
7.47
7.46
7.28
2.00
1.00
2.00
7.00
10.0 9.5 9.0 8.5 8.0 7.5 7.0 6.5 6.0 5.5 5.0 4.5 4.0 3.5 3.0 2.5 2.0 1.5 1.0 0.5 0.0
f1 (ppm)

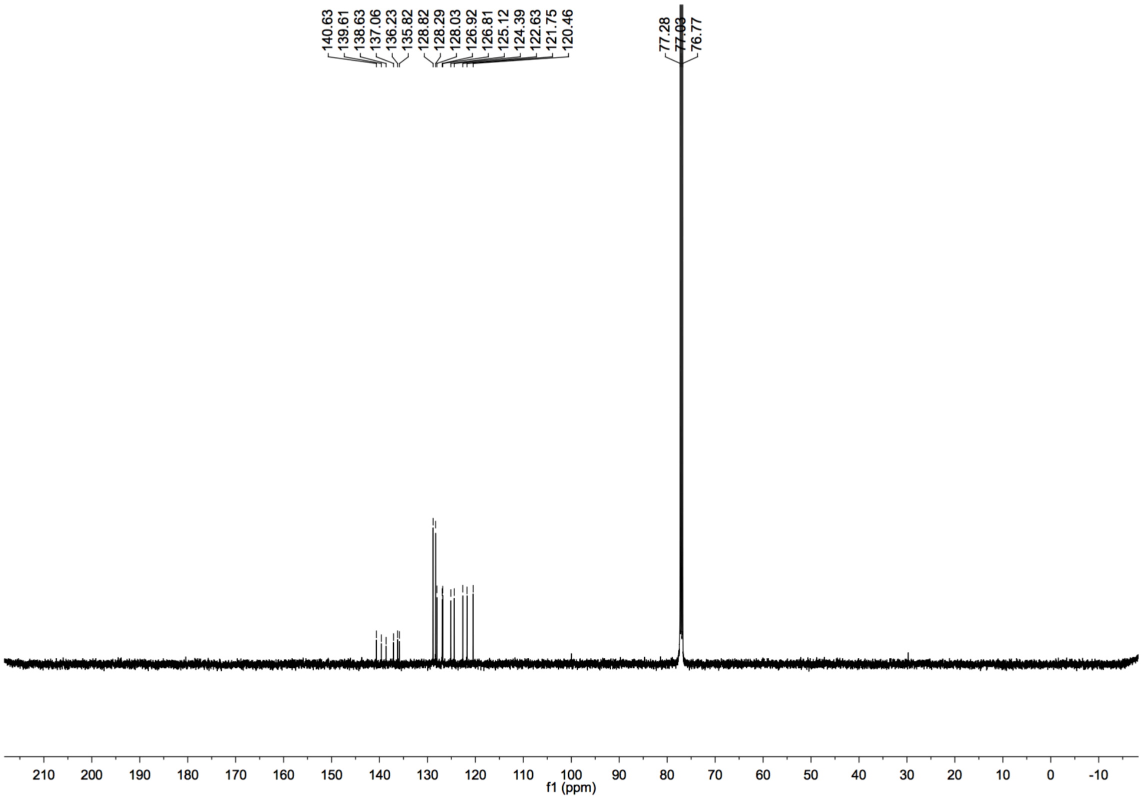
140.63
139.61
138.63
137.06
136.23
135.82
128.82
128.29
128.03
126.92
126.81
125.12
124.39
122.63
121.75
120.46
77.28
77.03
76.77
210 200 190 180 170 160 150 140 130 120 110 100 90 80 70 60 50 40 30 20 10 0 -10
f1 (ppm)

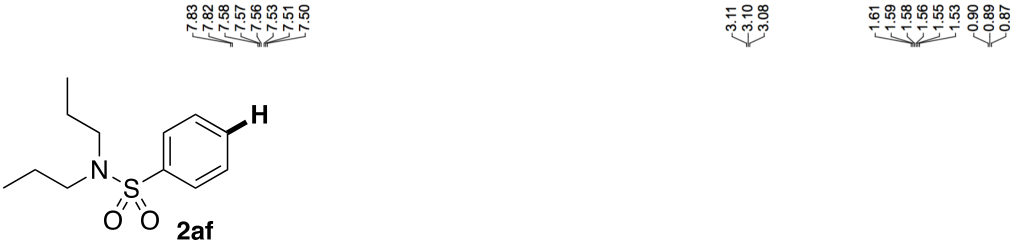
N
S
O
O
H

**2af**

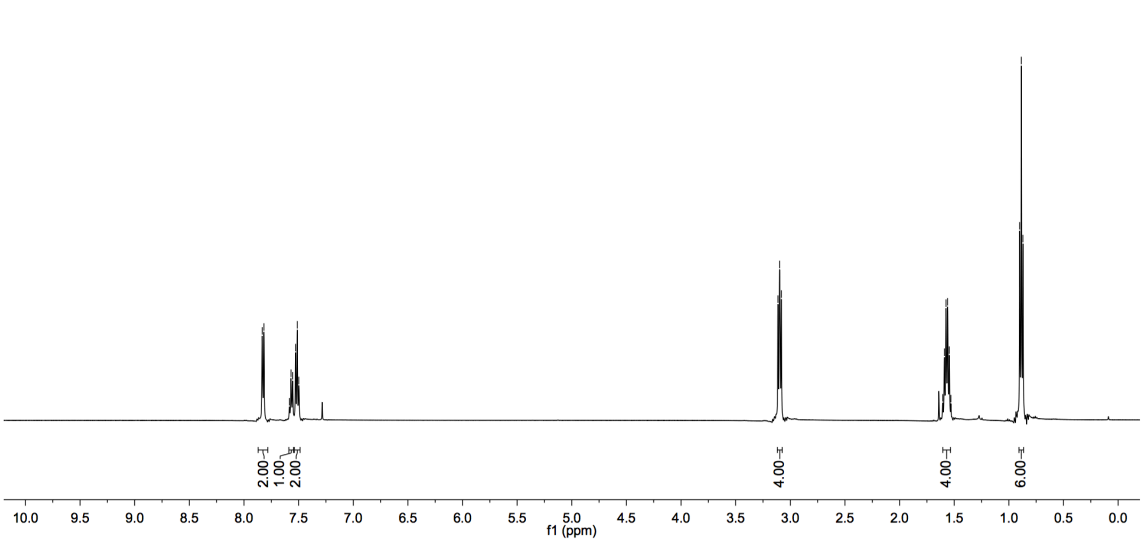
7.83
7.82
7.58
7.57
7.56
7.53
7.51
7.50
3.11
3.10
3.08
1.61
1.59
1.58
1.56
1.55
1.53
0.90
0.89
0.87
2.00
1.00
2.00
4.00
4.00
6.00
f1 (ppm)

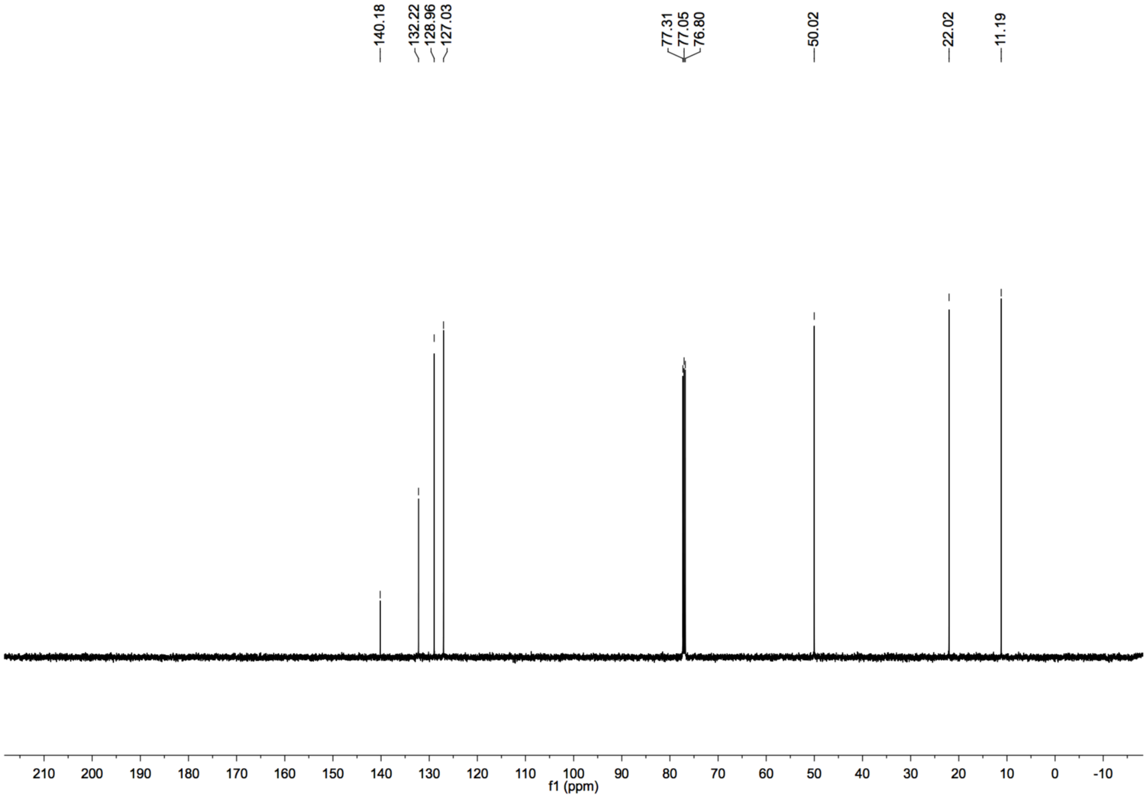
140.18
132.22
128.96
127.03
77.31
77.05
76.80
50.02
22.02
11.19
f1 (ppm)

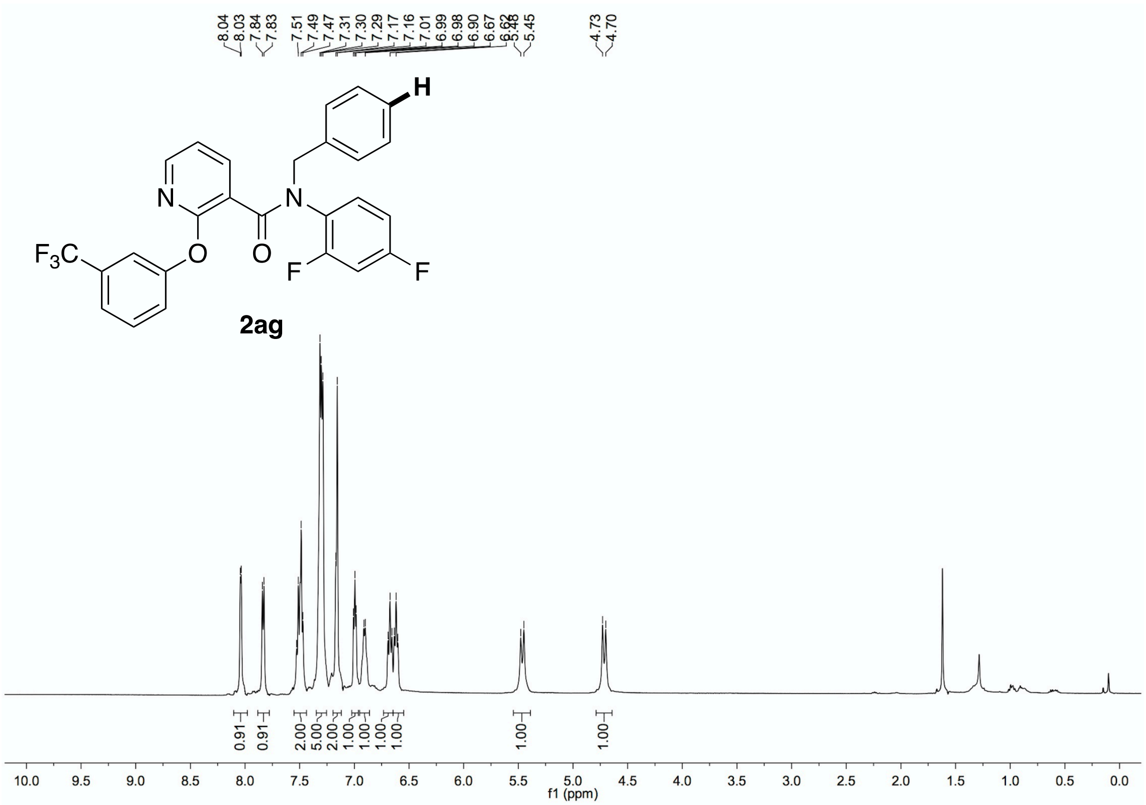
8.04
8.03
7.84
7.83
7.51
7.49
7.47
7.31
7.30
7.29
7.17
7.16
7.01
6.99
6.98
6.90
6.67
6.62
5.48
5.45
4.73
4.70
H
N
N
F3C
O
O
F
F
2ag
0.91
0.91
2.00
5.00
2.00
1.00
1.00
1.00
1.00
1.00
1.00
10.0 9.5 9.0 8.5 8.0 7.5 7.0 6.5 6.0 5.5 5.0 4.5 4.0 3.5 3.0 2.5 2.0 1.5 1.0 0.5 0.0
f1 (ppm)

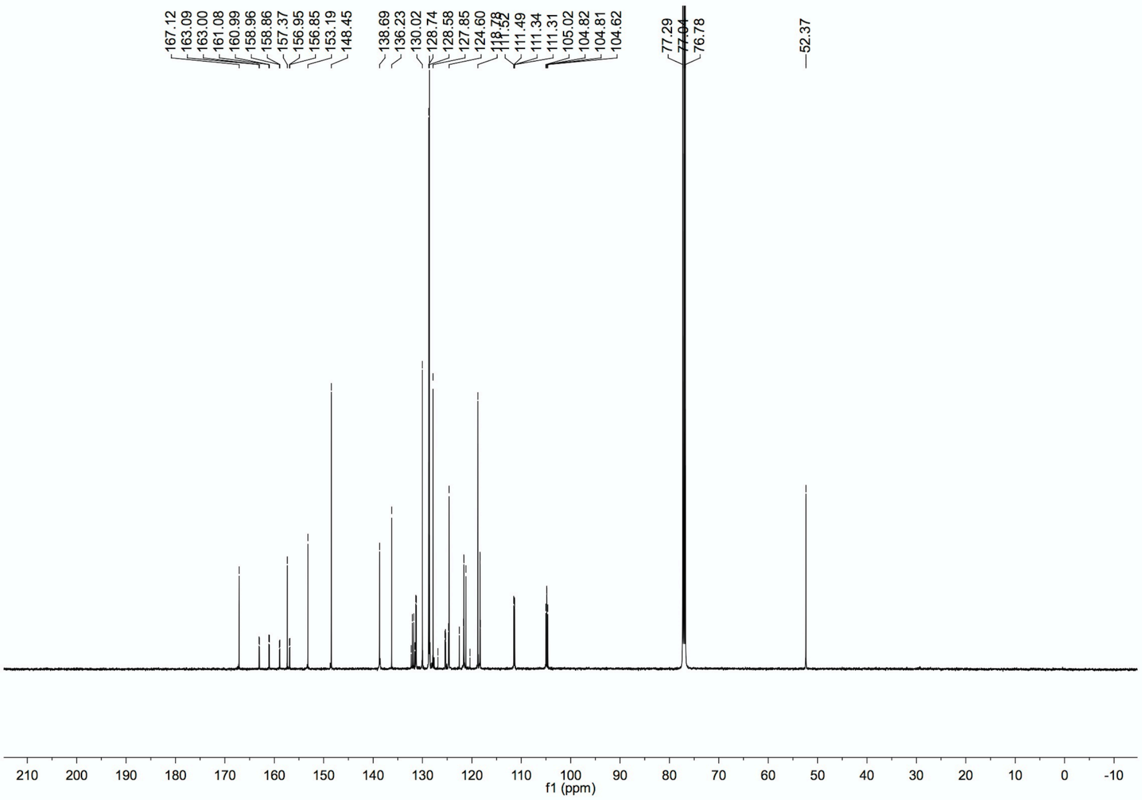
167.12
163.09
163.00
161.08
160.99
158.96
158.86
157.37
156.95
156.85
153.19
148.45
138.69
136.23
130.02
128.74
128.58
127.85
124.60
111.49
111.34
111.31
105.02
104.82
104.81
104.62
77.29
76.78
52.37
210 200 190 180 170 160 150 140 130 120 110 100 90 80 70 60 50 40 30 20 10 0 -10
f1 (ppm)

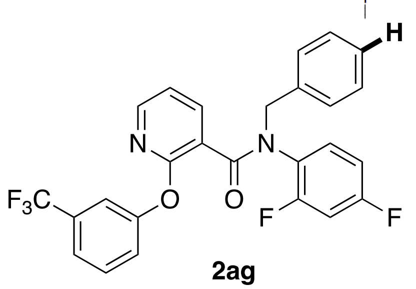

H
N
O
N
O
F₃C
F
F


**2ag**

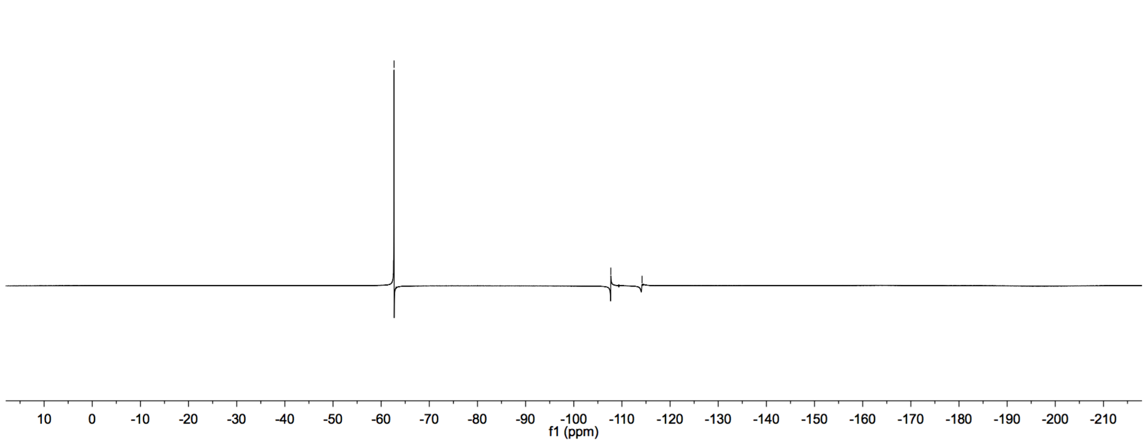

-62.68
-107.69
-114.19
10 0 -10 -20 -30 -40 -50 -60 -70 -80 -90 -100 -110 -120 -130 -140 -150 -160 -170 -180 -190 -200 -210
f1 (ppm)

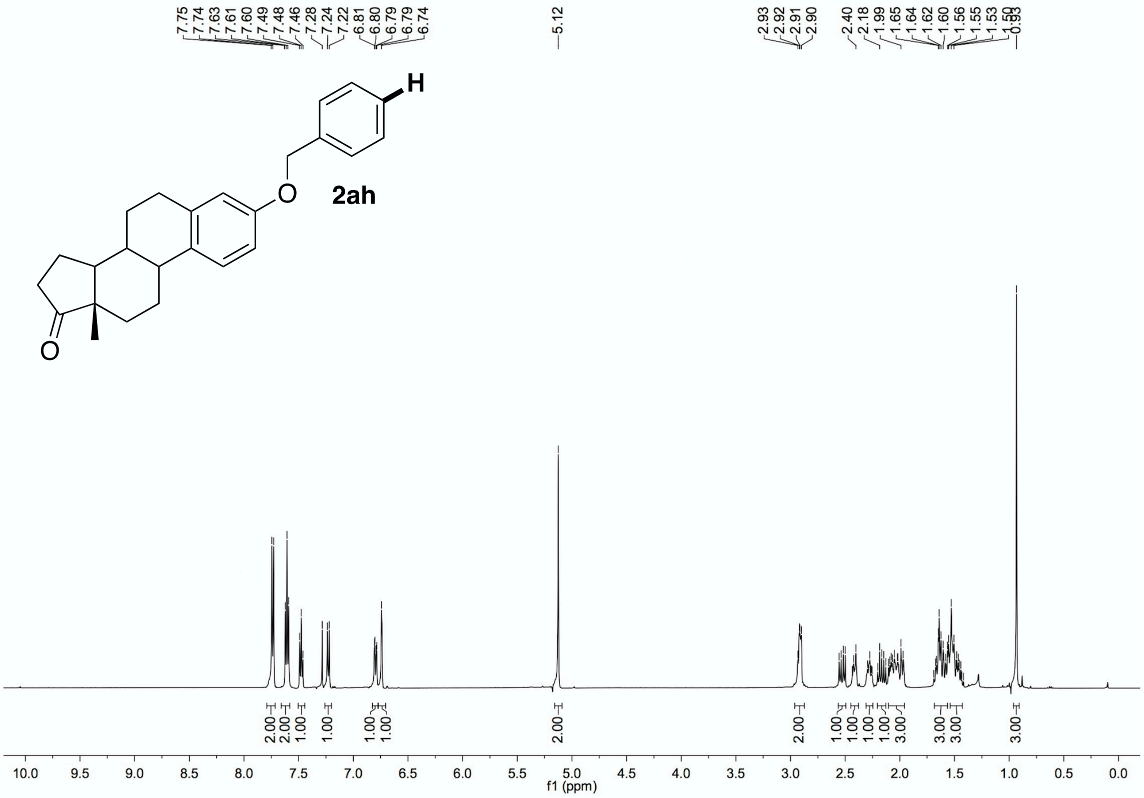
2ah
f1 (ppm)

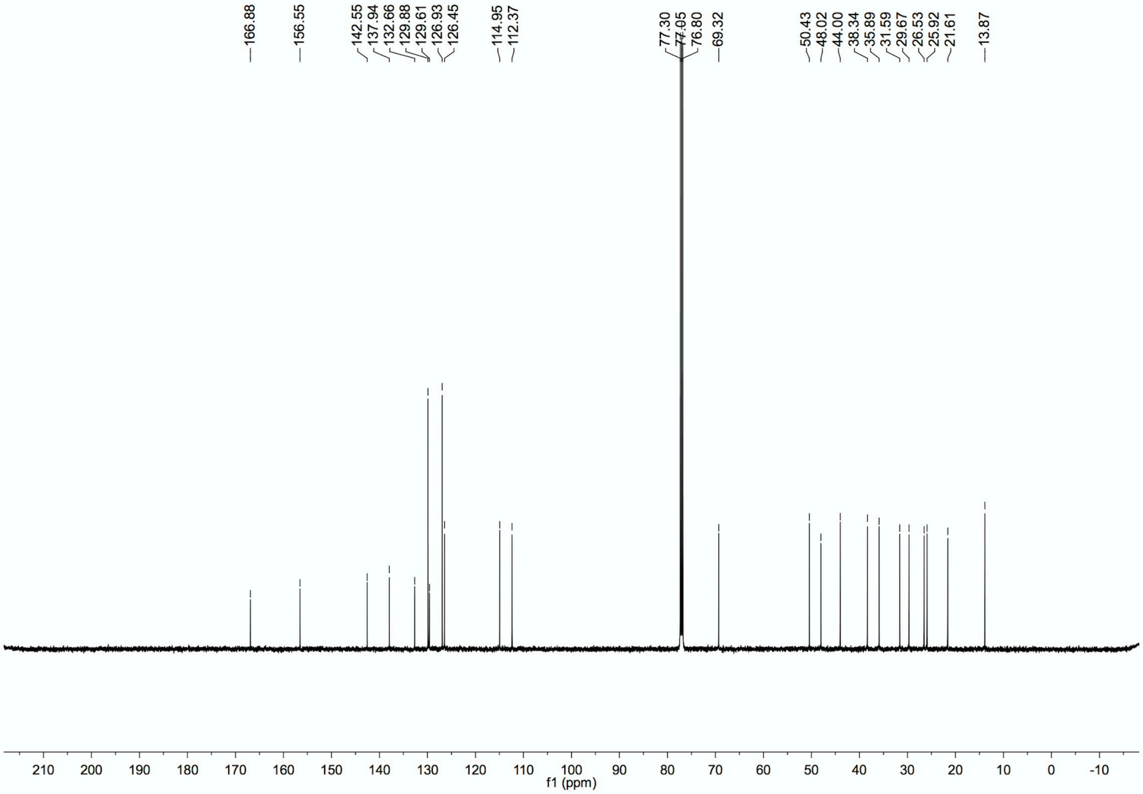
f1 (ppm)

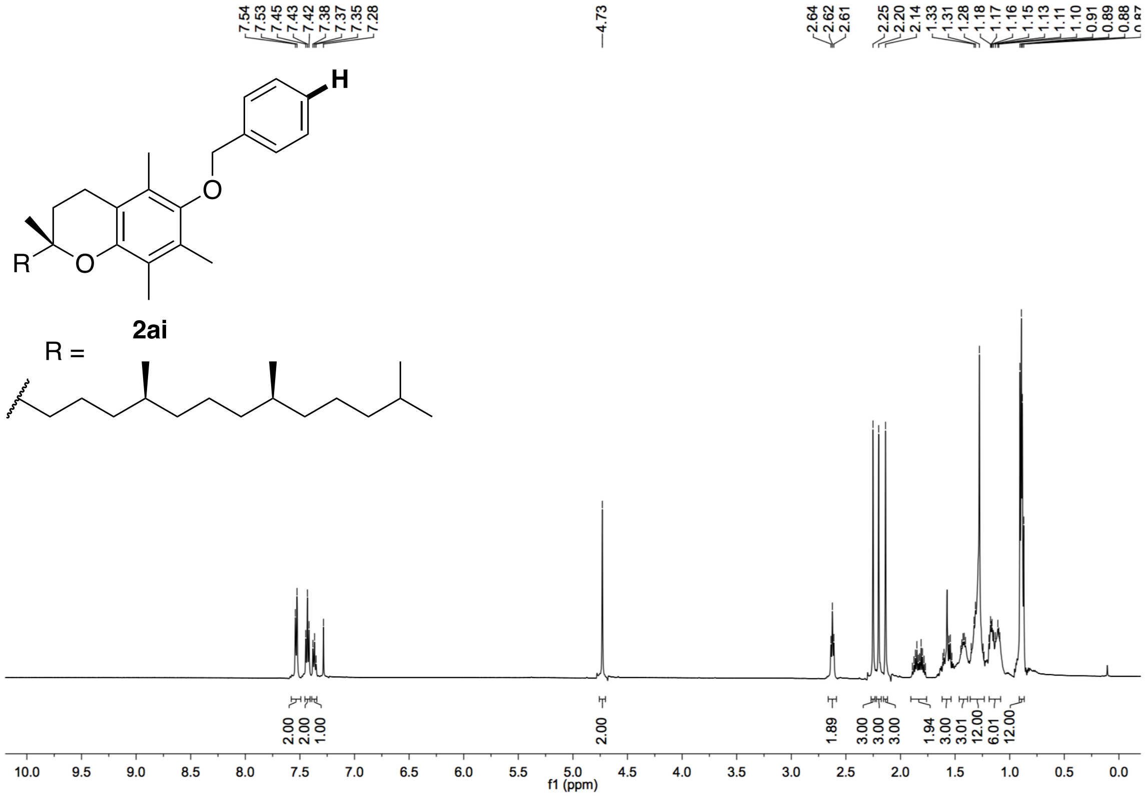

7.54
7.53
7.45
7.43
7.42
7.38
7.37
7.35
7.28
4.73
2.64
2.62
2.61
2.25
2.20
2.14
1.33
1.31
1.28
1.18
1.17
1.16
1.15
1.13
1.11
1.10
0.91
0.89
0.88
H
O
R
O
2ai
R =
2.00
2.00
1.00
2.00
1.89
3.00
3.00
3.00
1.94
3.00
3.01
12.00
6.01
12.00
f1 (ppm)


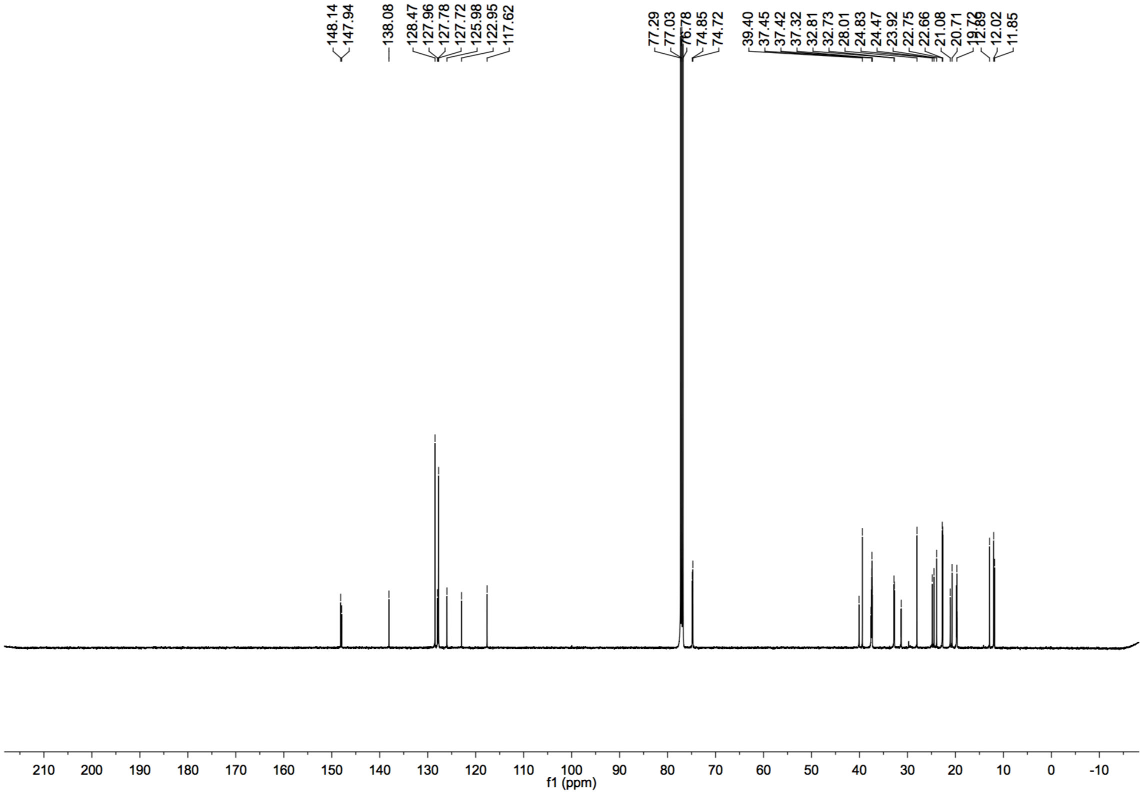

148.14
147.94
138.08
128.47
127.96
127.78
127.72
125.98
122.95
117.62
77.29
77.03
76.78
74.85
74.72
39.40
37.45
37.42
37.32
32.81
32.73
28.01
24.83
24.47
23.92
22.75
22.66
21.08
20.71
12.02
11.85
f1 (ppm)

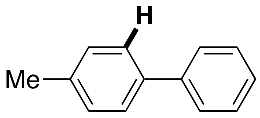
H
Me

**2aj**

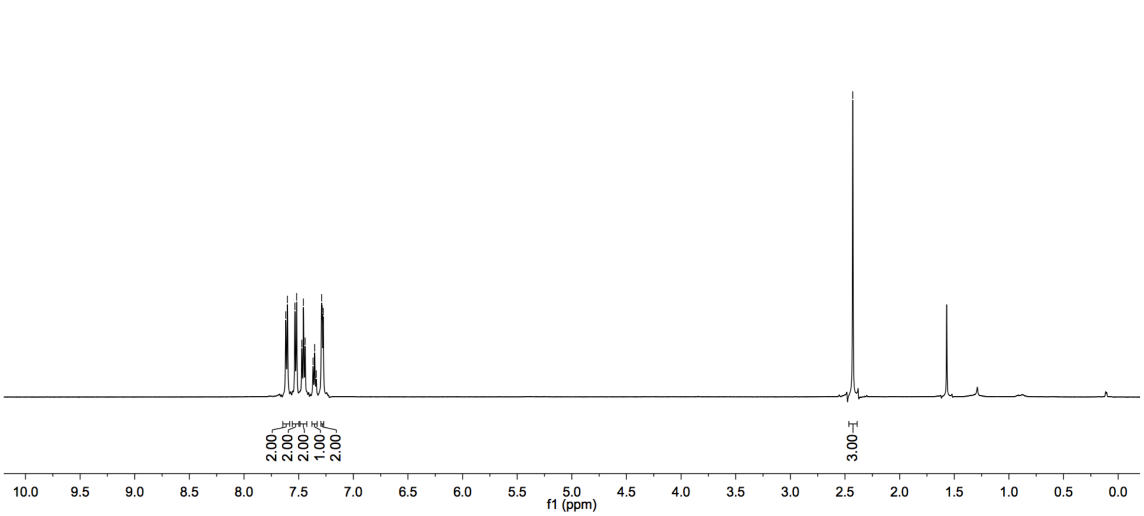
7.62
7.60
7.53
7.52
7.47
7.46
7.44
7.37
7.35
7.34
7.29
7.28
7.28
2.43
2.00
2.00
2.00
1.00
2.00
3.00
f1 (ppm)

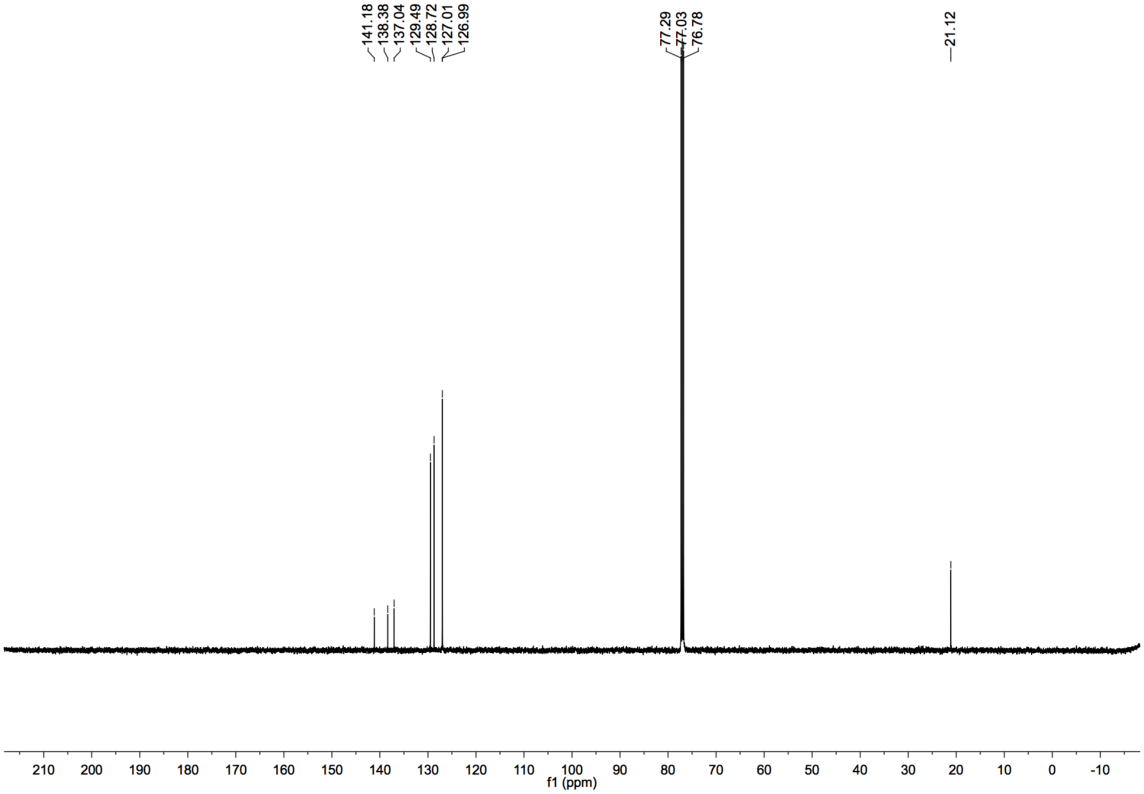
141.18
138.38
137.04
129.49
128.72
127.01
126.99
77.29
77.03
76.78
21.12
f1 (ppm)

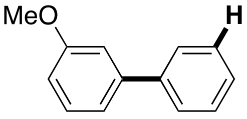
MeO
H

**2ak**

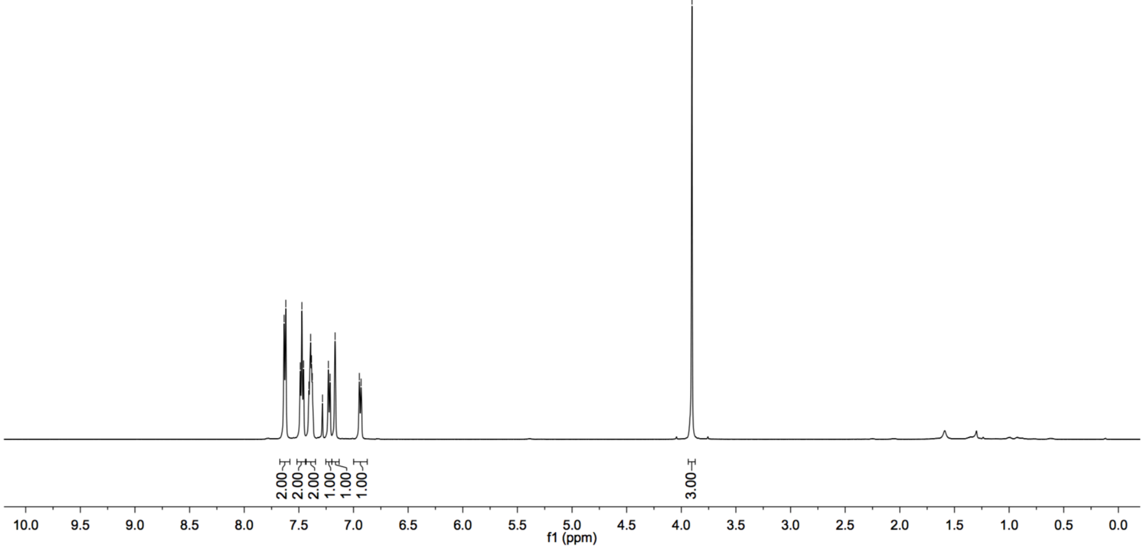
7.63
7.62
7.49
7.47
7.46
7.41
7.39
7.38
7.38
7.28
7.23
7.21
7.17
6.95
6.93
3.90
2.00
2.00
2.00
1.00
1.00
1.00
3.00
10.0 9.5 9.0 8.5 8.0 7.5 7.0 6.5 6.0 5.5 5.0 4.5 4.0 3.5 3.0 2.5 2.0 1.5 1.0 0.5 0.0
f1 (ppm)

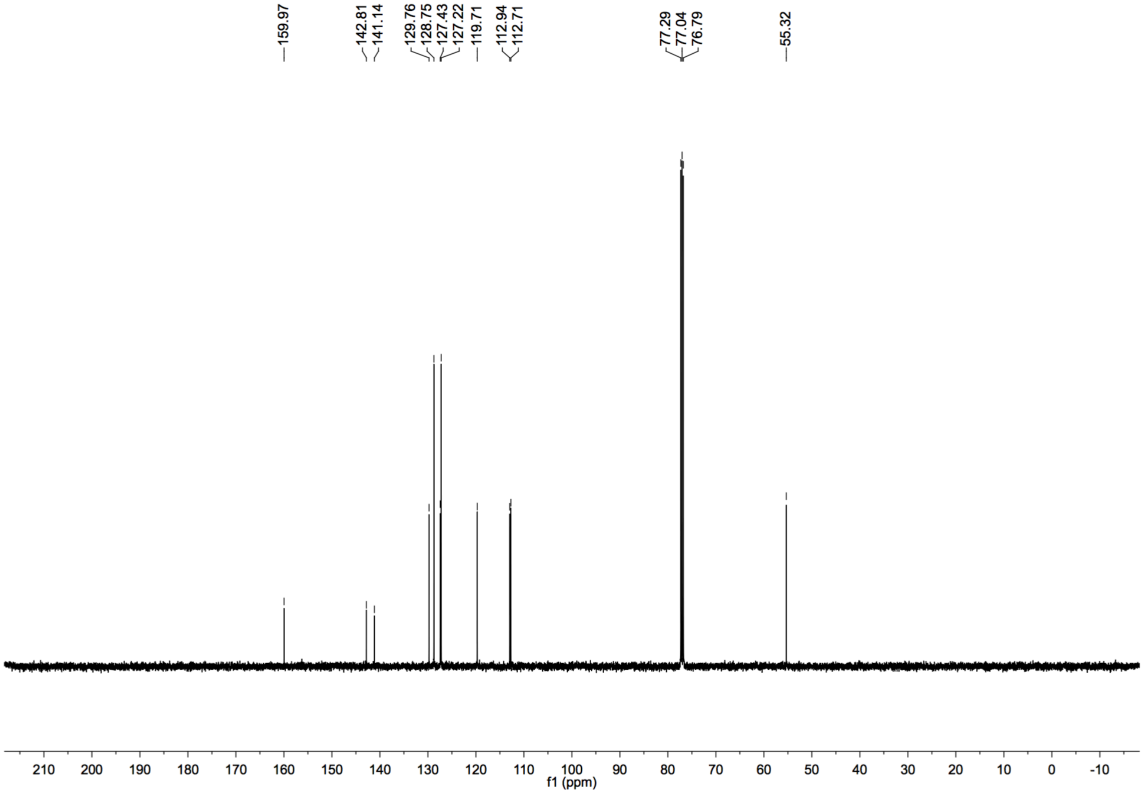
159.97
142.81
141.14
129.76
128.75
127.43
127.22
119.71
112.94
112.71
77.29
77.04
76.79
55.32
210 200 190 180 170 160 150 140 130 120 110 100 90 80 70 60 50 40 30 20 10 0 -10
f1 (ppm)
